# *MLH1* single-nucleotide variant in circulating tumor DNA predicts overall survival of patients with hepatocellular carcinoma

**DOI:** 10.1038/s41598-020-74494-y

**Published:** 2020-10-20

**Authors:** Soon Sun Kim, Jung Woo Eun, Ji-Hye Choi, Hyun Goo Woo, Hyo Jung Cho, Hye Ri Ahn, Chul Won Suh, Geum Ok Baek, Sung Won Cho, Jae Youn Cheong

**Affiliations:** 1 Department of Gastroenterology, Ajou University School of Medicine, Suwon, Republic of Korea; 2 Department of Physiology, Ajou University School of Medicine, Suwon, Republic of Korea; 3 Department of Biomedical Science, Ajou University Graduate School of Medicine, Suwon, Republic of Korea

**Keywords:** Gastroenterology, Molecular medicine, Oncology

## Abstract

Liquid biopsy can provide a strong basis for precision medicine. We aimed to identify novel single-nucleotide variants (SNVs) in circulating tumor DNA (ctDNA) in patients with hepatocellular carcinoma (HCC). Deep sequencing of plasma-derived ctDNA from 59 patients with HCC was performed using a panel of 2924 SNVs in 69 genes. In 55.9% of the patients, at least one somatic mutation was detected. Among 25 SNVs in 12 genes, four frequently observed SNVs, *MLH1* (13%), *STK11* (13%), *PTEN* (9%), and *CTNNB1* (4%), were validated using droplet digital polymerase chain reaction with ctDNA from 62 patients with HCC. Three candidate SNVs were detected in 35.5% of the patients, with a frequency of 19% for *MLH1* chr3:37025749T>A, 11% for *STK11* chr19:1223126C>G, and 8% for *PTEN* chr10:87864461C>G. The *MLH1* and *STK11* SNVs were also confirmed in HCC tissues. The presence of the *MLH1* SNV, in combination with an increased ctDNA level, predicted poor overall survival among 107 patients. *MLH1* chr3:37025749T>A SNV detection in ctDNA is feasible, and thus, ctDNA can be used to detect somatic mutations in HCC. Furthermore, the presence or absence of the *MLH1* SNV in ctDNA, combined with the ctDNA level, can predict the prognosis of patients with HCC.

## Introduction

Liver cancer is the sixth most commonly diagnosed cancer and the fourth most common cause of cancer-related deaths globally^1^. Recently, systemic therapy for advanced hepatocellular carcinoma (HCC) has progressed drastically. In addition to sorafenib, several molecular-targeted agents and an immune checkpoint inhibitor have succeeded in clinical trials and have been used in the clinic^2^. However, the efficacy of these systemic therapies is still clinically insufficient^3–7^, likely due to factors such as the trial design, lack of patient stratification based on tumor mutations, and off-target activity of these agents.

In the current era of precision medicine, the detection of molecular drivers of tumorigenesis and DNA mutations in tumor samples has become a routine clinical practice to evaluate biomarkers that predict the response or resistance to targeted agents^8^. Liquid biopsy is based on convenient and minimally invasive collection of blood or urine samples, which can be acquired at multiple time points over the course of disease. Unlike most solid tumors, HCC can be confidently diagnosed using imaging techniques, which limits the availability of tissue biopsies for retrospective biomarker studies. Therefore, liquid biopsy can potentially provide a strong basis for individualized treatment of patients with HCC^8^.

Circulating tumor DNA (ctDNA) is tumor-derived fragmented DNA that is found in the bloodstream of patients with cancer and varies substantially in composition, from < 0.01 to > 60% of alleles in circulation^9,10^. The ctDNA of a patient contains the information regarding tumor-specific genetic or epigenetic alterations, including point mutations, copy-number variations, chromosomal rearrangements, and DNA methylation patterns of the tumor. Quantitative or qualitative analysis of ctDNA may reveal valuable information for early diagnosis, treatment, and tumor progression monitoring^11^.

The amount of ctDNA may represent a novel tool for screening, detecting, treatment monitoring, and predicting metastatic potential of HCC^12–18^. Studies have reported that tumor-specific mutations in *TP53*^19^, *ITH*^20^, *HCK*^21^, *CTNNB1*^22^, and *TERT*^22^ are commonly detected in ctDNA from the peripheral blood of patients with HCC. However, there is no standard cutoff for the amount of ctDNA or a specific mutation in ctDNA that can be applied in clinical practice for predicting the HCC prognosis. Therefore, we aimed to screen for novel single nucleotide variants (SNVs) in HCC using targeted deep sequencing (TDS) of 69 candidate genes and validate the candidate SNVs using droplet digital polymerase chain reaction (ddPCR) for both blood and tissues. We also aimed to find the clinical correlation between candidate SNVs and the prognosis of patients with HCC.

## Results

### Patients’ demographics

The demographic and clinical parameters of the 107 patients with HCC who were included in the TDS or ddPCR analysis are shown in Supplementary Table S1. In total, 80 patients (74.8%) were older than 50 years, and 88 patients (82.2%) were males. The major etiology of HCC was hepatitis B virus infection, and 89 patients (83.2%) had good liver function (Child–Pugh class A). Serum levels of alpha-fetoprotein were higher than 200 ng/mL in approximately one-third of the patients (36.4%). The proportions of patients with stages 0, A, B, C, and D per the Barcelona Clinic Liver Cancer (BCLC) classification criteria were 5.6%, 42.1%, 15.9%, 32.7%, and 3.7%, respectively.

### Identification of novel SNVs in ctDNA from patients with HCC

**Figure 1:**
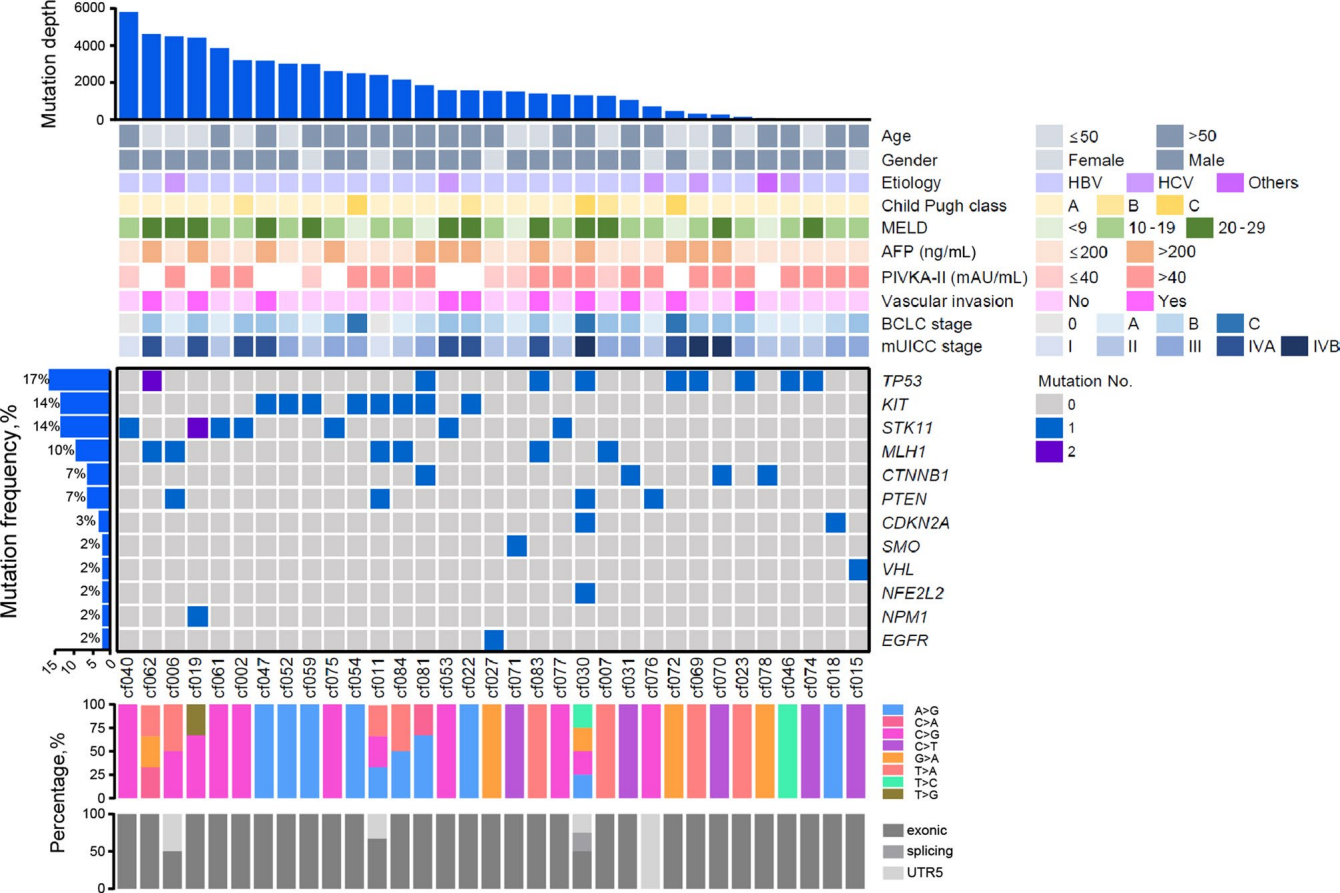
SNV landscape of 33 patients with HCC who tested positive for SNVs using ctDNA sequencing of 69 cancer genes. The patients are sorted in descending order according to the number of genes in which mutations were detected. Genes are sorted in descending order according to the number of patients who had a mutation in a particular gene. The depth of the mutations detected in a patient and the clinical and pathological characteristics of the patient are shown in the upper panel. Genes with SNVs are shown in the middle panel. Mutation frequencies of each gene are shown on the left. The mutation spectrum is shown at the bottom. *ctDNA* circulating tumor DNA, *HCC* hepatocellular carcinoma, *SNV* single-nucleotide variant.

A total of 61 ctDNA sequencing datasets were generated from 59 patients with HCC and two controls (duplicate data using HapMap NA12878 cell line) and analyzed using a panel consisting of 2924 SNVs in 69 genes. Mutations were most frequently detected in *TP53* (17%), followed by *KIT* and *STK11* (14% each), *MLH1* (10%), *CTNNB1* and *PTEN* (7% each), *CDKN2A* (3%), and *SMO*, *VHL*, *NFE2L2*, *NPM1*, and *EGFR* (2% each). Finally, a total of 25 SNVs in 12 genes were identified in 33 patients (55.9%). The SNV landscape of these 33 patients with HCC is shown in Fig. 1. The associations between the presence of mutations in these 12 genes and clinical variables of the patients, including tumor characteristics, were evaluated, and *CDKN2A*, *EGFR*, *MLH1*, *NFE2L2*, *PTEN*, *TP53*, and *VHL* were found to be associated with several clinical parameters (Supplementary Table S2). Overall survival of the patients with *MLH1* or *NPM1* mutations was poor compared with that of the patients without these mutations (both log-rank *P* = 0.02; Supplementary Fig. S1).

**Figure 2:**
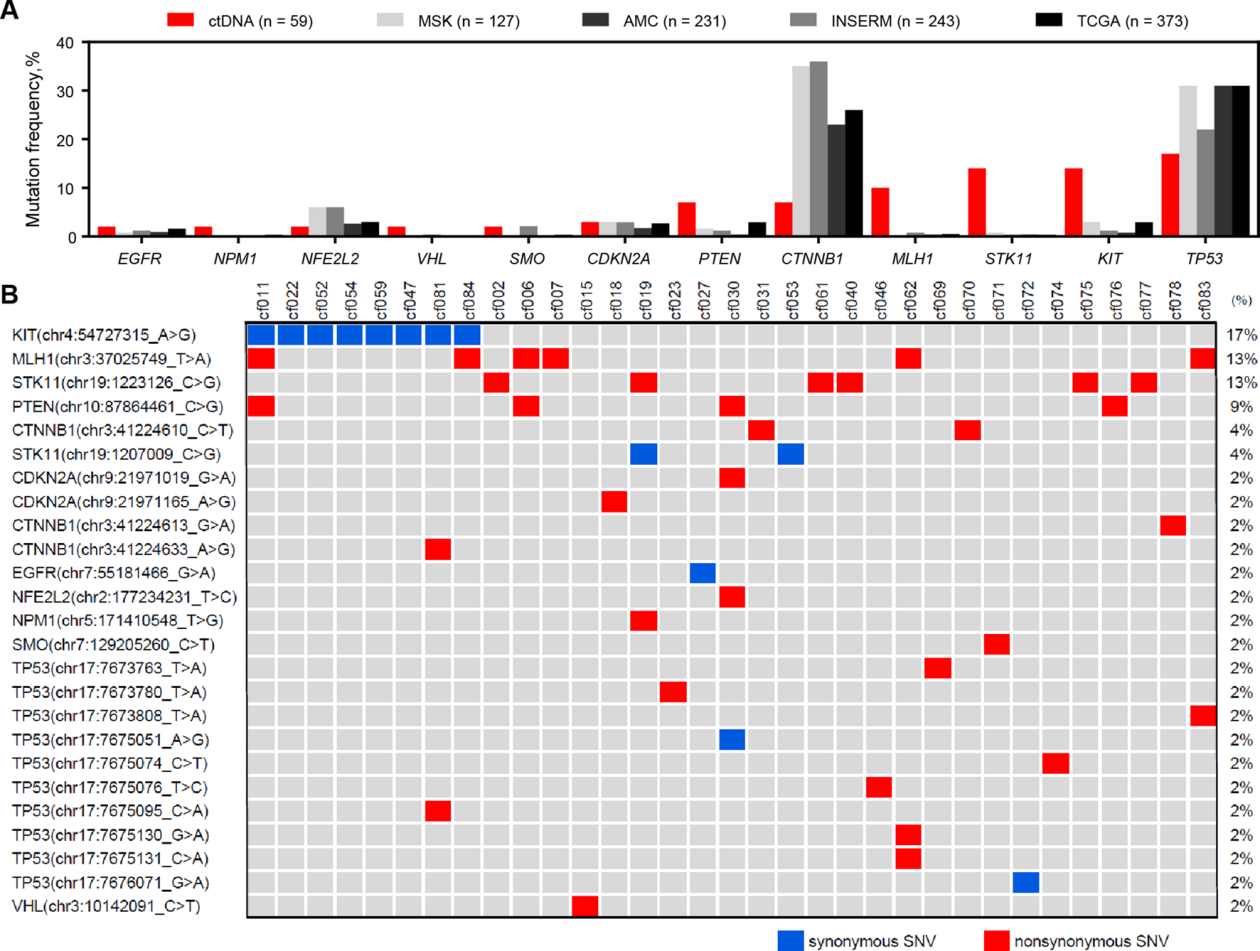
Genetic variations detected in ctDNA of patients with HCC. (**a**) Frequencies of variations in the ctDNA of 59 patients with HCC and frequencies of mutations in tissue genomic DNA in four HCC tissue cohorts. (**b**) ctDNA landscape of 25 variant sites in 12 genes from 33 patients with HCC. Samples are shown on the top, genes with variant sites on the left, and frequencies of these genes on the right. *ctDNA* circulating tumor DNA, *HCC* hepatocellular carcinoma.

The mutation frequencies in these 12 genes were investigated in HCC tissues using published data for four next-generation sequencing cohorts, including The Cancer Genome Atlas, Memorial Sloan Kettering-Integrated Mutation Profiling of Actionable Cancer Targets, French National Institute of Health and Medical Research, and Asan Medical Center ones (Fig. 2a)^23–26^. The most frequently mutated genes in the published data for the HCC tissue cohorts were *CTNNB1* (30%) and *TP53* (28.8%). On the contrary, *KIT*, *STK11*, *MLH1*, *PTEN*, *VHL*, *NPM1*, and *EGFR* variants were detected more frequently in ctDNA than in HCC tissues.

Among the 25 SNVs that were detected 47 times in 33 patients with HCC (Fig. 2b), the most frequently detected were the *KIT* chr4:54727315A>G (rs55986963; 17%), *MLH1* chr3:37025749T>A (rs63750447; 13%), *STK11* chr19:1223126C>G (rs59912467; 13%), *PTEN* chr10:87864461C>G (rs11202592; 9%), *CTNNB1* chr3:41224610C>T (rs121913400; 4%), and *STK11* chr19:1207009C>G (rs79175212; 4%). Considering their high detection frequencies and nonsynonymous substitutions, *MLH1* chr3:37025749T>A, *STK11* chr19:1223126C>G, *PTEN* chr10:87864461C>G, and *CTNNB1* chr3:41224610C>T were selected for further validation using ddPCR analysis.

### Detection of SNVs in blood ctDNA of patients with HCC using ddPCR

**Figure 3:**
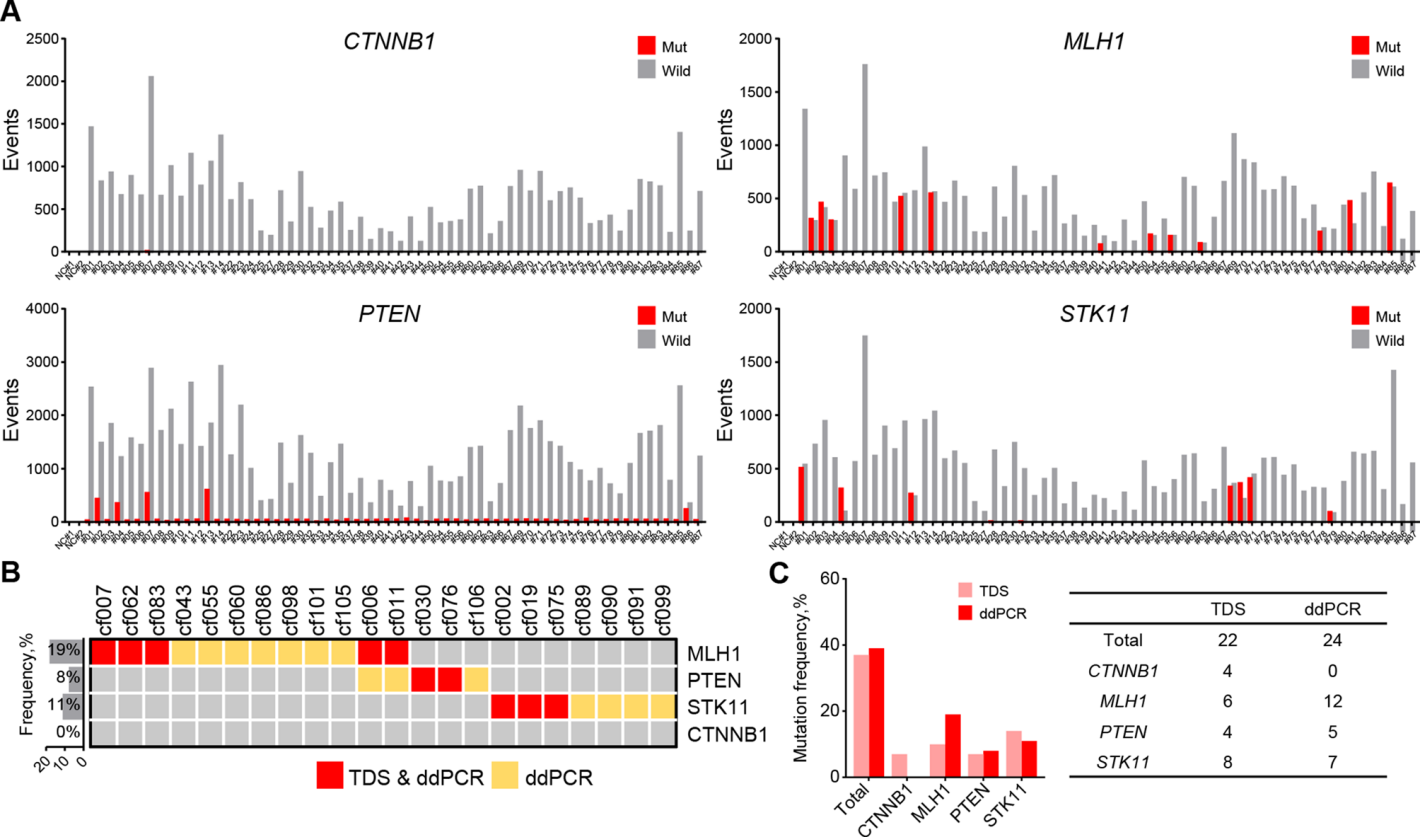
Validation of four genes with SNVs detected in ctDNA in a validation set of 62 patients with HCC using ddPCR. (**a**) Total number of positive droplet events recorded via ddPCR of the four genes. Gray bars represent the number of wild-type-positive droplets, and red bars represent the number of mutant-positive droplets. (**b**) ctDNA landscape of the four genes with SNVs. (**c**) Mutation frequencies in the four genes detected in patients with HCC using TDS and ddPCR. *ctDNA* circulating tumor DNA, *ddPCR* droplet digital polymerase chain reaction, *HCC* hepatocellular carcinoma, *SNV* single-nucleotide variant, *TDS* targeted deep sequencing.

The four selected SNVs were analyzed using ddPCR in ctDNA of 62 patients with HCC. The *MLH1*, *PTEN*, and *STK11* mutation-positive and -negative droplet clusters could be clearly distinguished. However, the *CTNNB1* mutation was not detected, as no positive droplet clusters were visualized (Fig. 3a and Supplementary Fig. S2). The most frequently detected mutation was that in *MLH1* (19%, 12/62 patients), followed by *STK11* (11%, 7/62 patients) and *PTEN* (8%, 5/62 patients) (Fig. 3b). In the 62 patients, the *MLH1* SNV detection rate of ddPCR was twice as high as that of TDS (Fig. 3c).

### Prognostic potential of the *MLH1*, *PTEN*, *STK11*, and *CTNNB1* SNVs in patients with HCC

The associations between clinical parameters and the *MLH1*, *PTEN*, *STK11*, and *CTNNB1* SNVs were evaluated in the 107 patients whose SNVs were analyzed by TDS or ddPCR. Only the *MLH1* SNV was significantly associated with the BCLC stage (*P* = 0.025; Supplementary Table S3), but no SNV was significantly associated with overall survival. However, subgroup analysis of the patients with advanced-stage HCC (BCLC stage C/D or modified Union for International Cancer Control stage IV) showed that the patients with the *MLH1* SNV had a significantly lower survival than did those without this SNV (Supplementary Fig. S3). The *CTNNB1*, *PTEN*, and *STK11* SNVs were not associated with the patient prognosis (Supplementary Fig. S4).

### *MLH1* and *STK11* SNV detection in tissue genomic DNA (tDNA) using ddPCR

**Figure 4:**
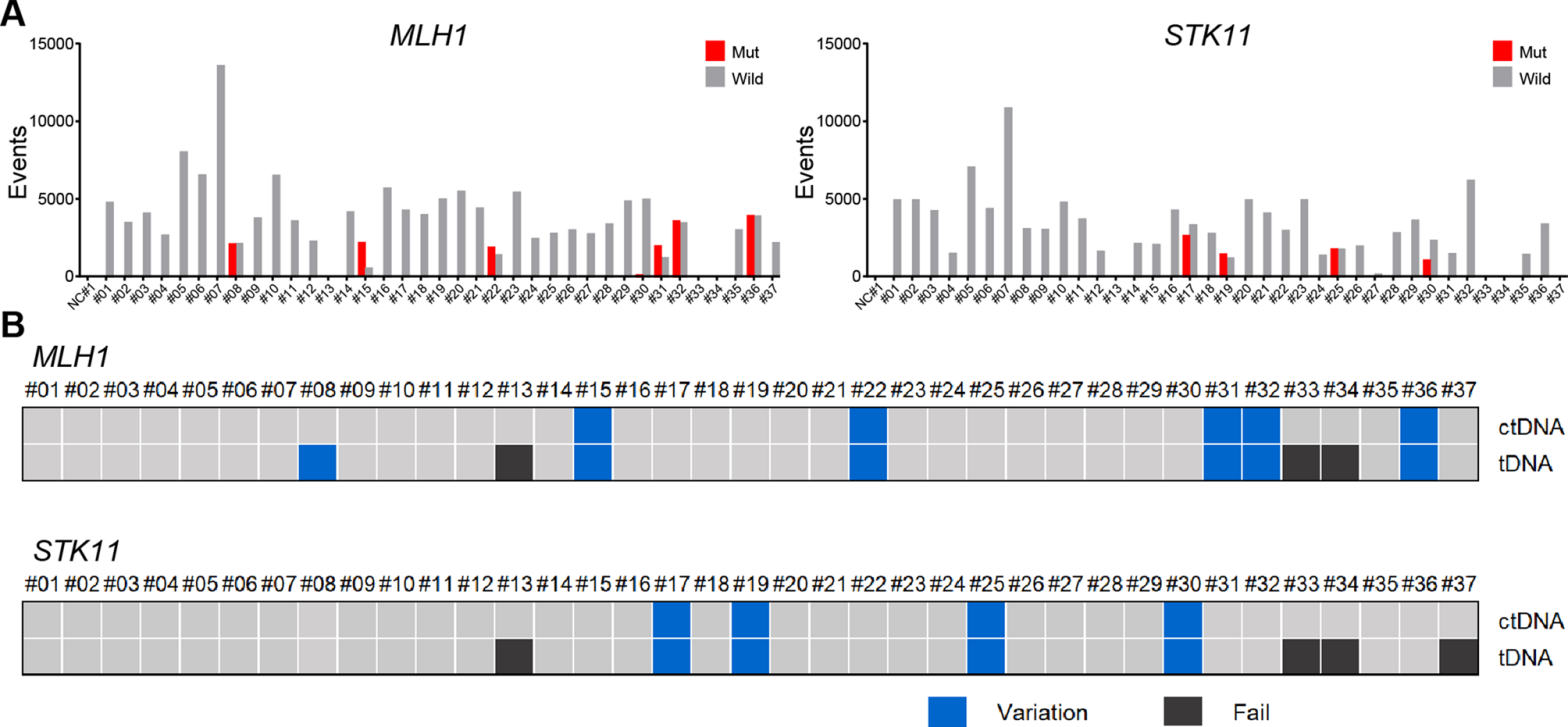
*MLH1* and *STK11* SNV detection in paired ctDNA and tumor DNA samples using ddPCR. (**a**) Total number of positive droplet events in ddPCR analysis of the two genes. Gray bars represent the number of wild-type-positive droplets, and red bars represent the number of mutant-positive droplets. (**b**) Comparison of SNV detection results for the two genes between paired ctDNA and tumor DNA samples. Samples with positive, negative, and failed SNV detection are shown in blue, gray, and black, respectively. *ctDNA* circulating tumor DNA, *ddPCR* droplet digital polymerase chain reaction, *SNV* single-nucleotide variant.

To determine whether the mutations detected in ctDNA were derived from HCC tissue, tDNA was extracted from available HCC tissues of 37 out of the 62 patients who were analyzed using ddPCR. *MLH1* and *STK11*, the most frequently mutated genes detected in ctDNA using ddPCR, were selected for tDNA ddPCR analysis. The results are shown in Fig. 4a. In all cases, both *MLH1* and *STK11* mutations detected in ctDNA were also detected in tDNA. Only one patient, who had the *MLH1* mutation in tDNA, did not show the same mutation in ctDNA (Fig. 4b).

### Association of the ctDNA level and presence of the *MLH1* SNV with patient clinicopathological features and overall survival

**Figure 5:**
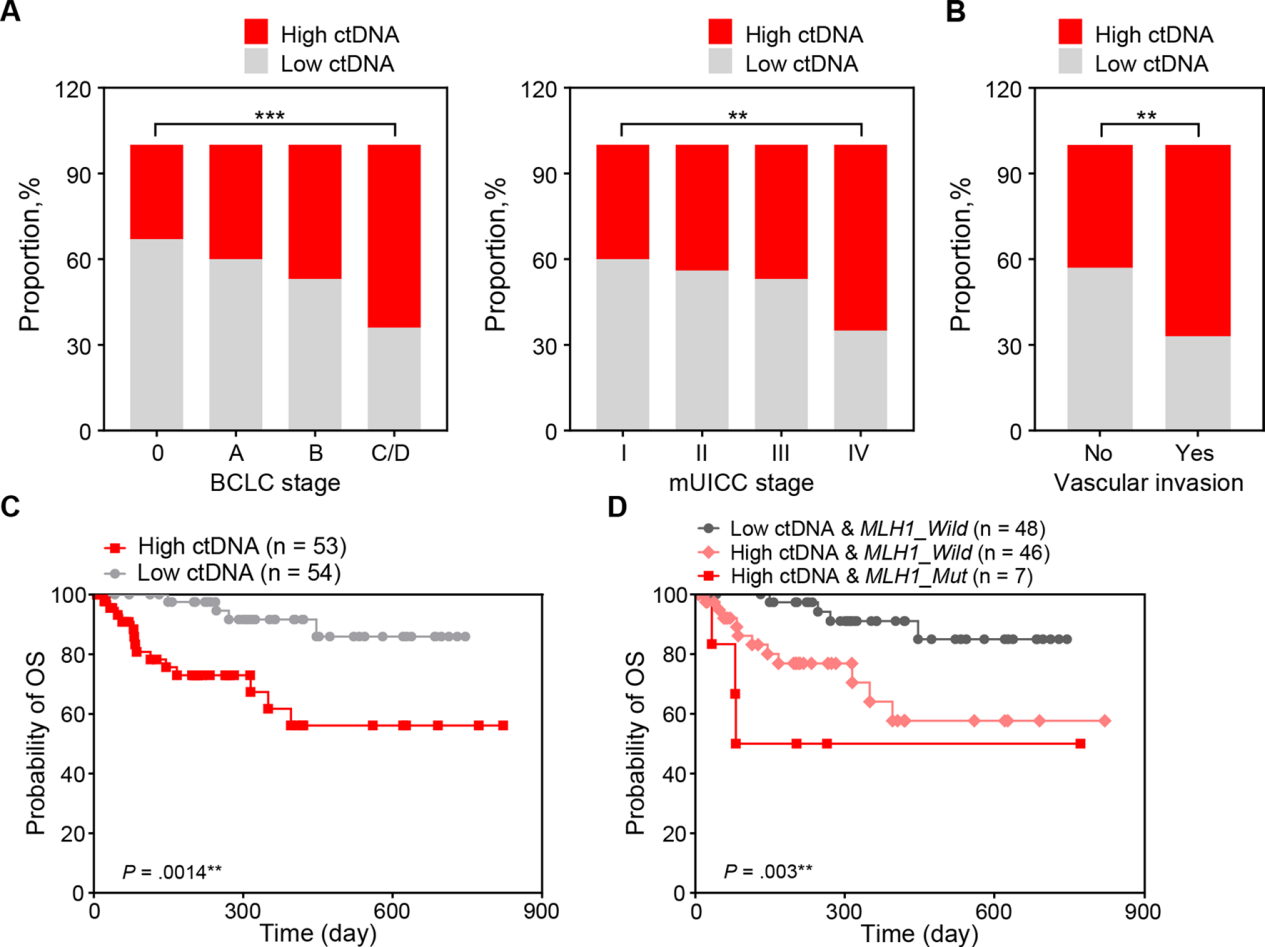
Association of the ctDNA level and *MLH1* SNV with clinicopathological parameters and overall survival of patients with HCC. (**a**) Proportion of patients in the high-versus low-ctDNA group according to the BCLC stage. (**b**) Proportion of patients in the high-versus low-ctDNA group according to the mUICC stage. (**c**) Overall survival curves according to the ctDNA levels in the plasma of patients with HCC. (**d**) Overall survival curves according to the ctDNA levels and presence of the *MLH1* SNV. *BCLC* Barcelona Clinic Liver Cancer, *ctDNA* circulating tumor DNA, *HCC* hepatocellular carcinoma, *mUICC* modified Union for International Cancer Control, *SNV* single-nucleotide variant.

The amount of ctDNA was quantified in each of the 107 patients with HCC. Using the median concentration of recovered ctDNA (5.77 ng/mL), the patients were divided into high (≥ 5.77 ng/mL)- and low (< 5.77 ng/mL)-ctDNA groups. The proportion of patients with high ctDNA levels significantly increased with the HCC stage progression (*P* < 0.001 for BLCL stages; *P* < 0.01 for modified Union for International Cancer Control stages; Fig. 5a). Patients with vascular invasion also had increased ctDNA levels (*P* < 0.01; Fig. 5b). The overall survival of the patients in the high-ctDNA group was lower than that of the patients in the low-ctDNA group (log-rank *P* = 0.0014; Fig. 5c). Moreover, patients with the ctDNA *MLH1* mutation in the high-ctDNA group demonstrated worse overall survival, whereas those with no ctDNA *MLH1* mutation in the low-ctDNA group showed the most favorable overall survival (*P* = 0.003; Fig. 5d). On the other hand, when *MLH*1 SNV was combined with AFP level, the group showing low AFP (< 20 ng/mL) and no ctDNA *MLH*1 mutation had the best overall survival (*P* = 0.005; Supplementary Fig. S5).

## Discussion

The present study identified novel HCC-associated SNVs using a panel of 2924 SNVs in 69 genes to probe ctDNA from 59 patients with HCC. We identified 25 SNVs in 12 genes and validated four (*MLH1*, *STK11*, *PTEN*, and *CTNNB1*) SNV candidates using ddPCR on plasma from other 62 patients with HCC. The *MLH1* SNV (chr3:37025749T>A) was detected in ctDNA and confirmed in tDNA of patients with HCC. Of the four candidate SNVs, only the *MLH1* SNV correlated with the HCC stage and could predict overall survival in patients with advanced HCC. Patients with high levels of ctDNA (≥ 5.77 ng/mL) in the plasma and ctDNA positivity for the *MLH1* SNV exhibited the worst survival rate. To our knowledge, this is the first study to detect the *MLH1* chr3:37025749T>A SNV in the ctDNA of patients with HCC and to show its prognostic value for overall survival.

MLH1 plays a key role in DNA mismatch repair via recognition and repair of mismatched bases during DNA replication. Additionally, MLH1 recruits other mismatch repair proteins to the mismatch site to correct DNA replication errors^27,28^. Defects in repair genes may lead to genomic instability and cancer development, suggesting that polymorphisms in specific repair genes may contribute to individual differences in the risk of developing cancer^29–31^. It has been reported that *MLH1* polymorphisms correlate with the pathogenesis of colorectal cancer, lung cancer, breast cancer, prostate cancer, head and neck squamous cell carcinoma, and oral squamous cell carcinoma^29–36^. However, there are only a few studies on the relationship between *MLH1* polymorphisms and HCC^37,38^. Two previous case–control studies found an increased risk of HCC and reported a poor prognosis in patients with the *MLH1* rs1800734 single-nucleotide polymorphism^37,38^. However, these studies did not include the *MLH1* chr3:37025749T>A (rs63750447) SNV nor did they analyze ctDNA of patients with HCC. Therefore, the present study is the first to clarify the relationship between the *MLH1* chr3:37025749T>A SNV, particularly in ctDNA, and HCC.

The *MLH1* chr3:37025749T>A SNV, which is located in an exon, induces an amino acid substitution of valine to aspartate. The global minor allele frequency of the *MLH1* chr3:37025749T>A SNV is very low (at 0.519%)^39^. We found the incidence of the *MLH1* chr3:37025749T>A SNV to be approximately 13–19% using TDS and ddPCR, which suggests a surprisingly high frequency of this *MLH1* SNV 37025749T>A in patients with HCC. Considering that the reported minor allele frequency of the same polymorphism is 3–8% in Chinese and Japanese patients with other cancers, such as colorectal and lung cancer^40–42^, the incidence of the *MLH1* chr3:37025749T>A SNV in Korean patients with HCC is quite high. Therefore, the *MLH1* chr3:37025749T>A SNV may be more specific to HCC, especially in Korean patients.

Previous studies have reported that the detection of the *MLH1* chr3:37025749T>A polymorphism in the blood is associated with an increased risk of gastric, colorectal, and endometrial cancer in Asian populations^43^. The possible underlying mechanism for the influence of the *MLH1* chr3:37025749T>A SNV on cancer development may be associated with the mismatch repair function of the MLH1 protein. The amino acid affected by the *MLH1* chr3:37025749T>A SNV is located between the NH_2_-terminal ATP-binding domain and the COOH-terminal PMS2 interaction domain of the MLH1 protein. The *MLH1* chr3:37025749T>A SNV alters the charge of the protein and is likely to disrupt its structure and stability. Previous studies have demonstrated decreased in vitro mismatch repair and β-galactosidase activities of the MLH1 protein encoded by the *MLH1* chr3:37025749T>A variant gene^44,45^. Taken together, the *MLH1* chr3:37025749T>A mutation is a partial loss-of-function variant that may increase the risk of cancer in the carriers.

The current study has a few limitations. First, we did not include patients without HCC, such as patients with liver cirrhosis. The HapMap NA12878 cell line was used as a control for the analysis of the TDS data. Second, only one *MLH1* SNV (chr3:37025749T>A) was included in the panel comprising 2924 variants in 69 genes. Therefore, we did not study the correlation between other *MLH1* SNVs and HCC. Third, hepatitis B virus was the main etiology of HCC in the present study. Therefore, the study finding may not be generalizable to population with different etiology and external validation is crucial.

Despite outstanding advances in the precision medicine of various cancers, individualized cancer therapy using companion diagnostics is still challenging in HCC. This drawback may be due to genetic heterogeneity of HCC itself or a difficulty in obtaining liver tissue from patients because this cancer is uniquely diagnosed based on imaging modalities. Liquid biopsy can be considered the most promising tool to overcome these limitations in HCC precision therapy. The current study demonstrated the possibility of using ctDNA to detect HCC mutations and predict the prognosis of patients with HCC.

In conclusion, *MLH1* SNV detection in ctDNA is feasible, and thus, ctDNA can be used to confidently detect somatic mutations in HCC tissue. Furthermore, ctDNA positivity or negativity for the *MLH1* chr3:37025749T>A mutation can provide a prognostic value, with or without measurement of the amount of ctDNA in the plasma of patients with HCC.

## Methods

### Patients and sample collection

A total of 146 consecutive treatment-naïve patients with HCC were enrolled at Ajou University Hospital between May 2016 and April 2019 were enrolled. Patients were diagnosed with HCC if their tumor had a minimum diameter of > 1 cm, and typical features of HCC (arterial phase hyperenhancement, washout in the portal venous or delayed phase, threshold growth, and capsule appearance) were observed using multiphase computed tomography and/or magnetic resonance imaging. If these criteria were met but the diagnosis of HCC was only considered probable, a liver biopsy was performed for final diagnosis^46^. Patients were excluded if they had other concurrent malignancies. A volume of 10 mL of blood was collected from each patient. Sufficient ctDNA (≥ 7 ng/mL) for further analysis using TDS or ddPCR was only obtained from 107 patients. Snap-frozen HCC tissues from 35 patients who underwent hepatectomy and two patients who were diagnosed using liver biopsy were used for validation analysis with the ddPCR technique (Supplementary Fig. S6). All experiments were conducted in accordance with the ethical guidelines of the 1975 Declaration of Helsinki. Written informed consent was obtained from all patients, and the study was approved by the Institutional Review of Board of Ajou University of School of Medicine (approval No: AJIRB-BMR-OBS-16-344).

### Extraction of ctDNA and tDNA

ctDNA was extracted from plasma using the QIAamp circulating nucleic acid kit (Qiagen, Hilden, Germany) following the manufacturer’s instructions. The purity of ctDNA was determined using the Agilent high-sensitivity DNA kit and a Bioanalyzer 2100 instrument (Agilent Technologies, Santa Clara, CA, USA). tDNA was extracted from snap-frozen tissues using the DNeasy blood and tissue kit (Qiagen) according to the manufacturer’s instructions and was eluted in a volume of 200 μL. The purity of the extracted genomic DNA was assessed by electrophoresis on a 1% agarose gel, and the DNA concentration was quantified using a Qubit 2.0 fluorometer and the Agilent high-sensitivity DNA kit.

### Library construction and sequencing

A customized panel was designed that targeted 2924 SNVs in 69 genes (Supplementary Table S4). A sequencing library was constructed from ctDNA using the Celemics library preparation kit (Celemics, Seoul, Republic of Korea). The capture of hybridization targets was performed using Celemics customized capture probes and the Celemics target capture kit. PCR amplification of captured libraries was performed using the KAPA HiFi HotStart PCR kit (Kapa Biosystems, Boston, MA, USA). Captured samples were pooled and sequenced using the Illumina NextSeq 500 platform (Illumina, San Diego, CA, USA) with 2 × 150-bp paired-end runs.

### ctDNA sequencing and variant calling

Sequencing of ctDNA was performed on the Illumina NextSeq 500 platform with 151-bp paired-end reads, with a coverage greater than 2000 reads in the panel regions per sample. The raw image data were transformed to and stored in a FASTQ format. Low-quality sequence reads with a PHRED score of less than 30 and adapter sequence reads were trimmed using the Trim Galore! tool and then mapped to a human reference genome (hg38) using Burrows–Wheeler Aligner with default parameters. Local realignment of indels and the normalization of base quality scores were performed using the Genome Analysis Toolkit. Sequence variations were detected using VarScan2 and annotated using the ANNOVAR software. Somatic variants were filtered as follows: allele frequencies of the variants in the normal population were obtained from the 1000 Genomes Project and Exome Aggregation Consortium dataset, and variants with allele frequencies greater than 5% in either of the normal populations were filtered out. Variants in the control cell line were also filtered out (Supplementary Fig. S7).

### Public omics data analysis

For comparative analysis of ctDNA target variations, we used the data for four large liver cancer tissue cohorts (from The Cancer Genome Atlas, Memorial Sloan Kettering-Integrated Mutation Profiling of Actionable Cancer Targets, French National Institute of Health and Medical Research, and Asan Medical Center), with the whole-exome sequencing data listed on cBioPortal (https://www.cbioportal.org/)^23^.

### ddPCR

ddPCR was performed using a previously reported method^47^. Each 20-μL reaction contained 10 μL of a ddPCR supermix (no dUTP), 6 μL of a primer–probe premix (0.4 μL each of 10 μmol/L upstream and downstream primers, 0.2 μL of 10 μmol/L probe, and 5 μL of deionized water), and 4 μL of a nucleic acid extract. Each sample reaction was added to the middle of a DG8 cartridge. Next, 70 μL of oil was added to the bottom row of each lane to avoid bubble formation, and the wells were covered. The reaction system and droplet-forming cartridge were placed in a droplet generator and subjected to microdroplet treatment. Droplets were produced in the top row of the wells, and the suction volume was adjusted to 40 μL. The samples were then gently moved to 96-well plates, and a preheated PX1 heat sealing device was used with a sealing film (with the red line up) at 180 °C for 5 s. The PCR conditions were as follows: predenaturation at 95 °C for 10 min, 40 cycles of 94 °C for 30 s and 55 °C for 1 min, with a temperature change rate of 2 °C/s. The 96-well plate containing PCR-amplified products was then positioned on a QX200 microdrop reader and analyzed using the QuantaSoft software (Bio-Rad).

### Statistical analysis

The IBM SPSS software version 22.0 (SPSS, Inc., Chicago, IL, USA) and the GraphPad Prism 7.01 software (GraphPad Software, San Diego, CA, USA) were used for statistical analyses. All tests were two-sided and considered statistically significant if *P* was < 0.05. Continuous variables were compared using independent sample *t*-tests. Analysis of variance was used to compare continuous variables between more than three groups. Categorical data were compared using a Pearson χ^2^ test or a Fisher exact test. Survival analysis was performed using the Kaplan–Meier method with a log-rank test.

## Supplementary information

Supplementary information.

## Data Availability

The data used or analyzed in this article is available from the corresponding author upon reasonable request.
