# Supplementary material for: MLH1 single-nucleotide variant in circulating tumor DNA predicts overall survival of patients with hepatocellular carcinoma
Source: Sci Rep. 2020 Oct 20;10:17862. doi: 10.1038/s41598-020-74494-y (PMC7576198; doi:10.1038/s41598-020-74494-y)

**MLH1 single nucleotide variant in circulating tumor DNA predicts overall survival of patients with hepatocellular carcinoma**

**Soon Sun Kim, Jung Woo Eun, Ji-Hye Choi, Hyun Goo Woo, Hyo Jung Cho, Hye Ri Ahn, Chul Won Suh, Geum Ok Baek, Sung Won Cho, Jae Youn Cheong**

**Supplementary Online Content**

**Supplementary Table S1. Patient Demographics and Clinical Characteristics**

**Supplementary Table S2. Association Between the Patients’ Clinical Variables and the Presence of 12 Genes Mutations Using Targeted Deep Sequencing**

**Supplementary Table S3. Association Between clinical parameters and *MLH1, PTEN, STK11*, and *CTNNB1* SNVs**

**Supplementary Table S4. Customized Panel Targeting 2924 SNVs in 69 Genes**

**Supplementary Figure S1. Overall Survival Curves With SNVs of 12 ctDNA Genes in HCC Patients (n = 59)**

**Supplementary Figure S2. 2D–Plots and Amount of Positive Droplets of ddPCR Results of 4 ctDNA Genes in HCC Patients**

**Supplementary Figure S3. Prognostic Potential of the MLH1 SNV for HCC**

**Supplementary Figure S4. Analysis of Overall Survival of Patient Outcome Predicted by the SNV Status of ctDNA Genes in HCC**

**Supplementary Figure S5. Association of the AFP level and MLH1 SNV with overall survival of patients with HCC.**

**Supplementary Figure S6. Strategy Used to Identify Novel Single Nucleotide Variants of ctDNA for HCC**

**Supplementary Figure S7. The Schematic of Data Analyses of Targeted Deep Sequencing**

**Supplementary Table S1.** Patient Demographics and Clinical Characteristics. AFP, alpha-fetoprotein; BCLC, Barcelona Clinic Liver Cancer; HBV, hepatitis B virus; HCV, hepatitis C virus; MELD, model for end-stage liver disease; mUICC, modified Union for International Cancer Control; PIVKA-II, Protein induced by vitamin K absence-II; RFA, Radiofrequency ablation; TACE, Transarterial chemoembolization.

| Characteristics | Count (%) |
| --- | --- |
| Age (>50 years) | 80 (74.8) |
| Gender (male) | 88 (82.2) |
| Etiology |  |
| HBV | 72 (67.3) |
| HCV | 11 (10.3) |
| Others | 24 (22.4) |
| Child Pugh class |  |
| A | 89 (83.2) |
| B | 14 (13.1) |
| C | 4 (3.7) |
| MELD |  |
| <9 | 65 (60.7) |
| 10–19 | 33 (30.8) |
| 20–29 | 2 (1.9) |
| AFP (>200 ng/mL) | 39 (36.4) |
| PIVKA-II (>40 mAU/mL) | 61 (57.0) |
| Vascular invasion | 30 (28.0) |
| Lymph node metastasis | 13 (12.1) |
| Distant metastasis | 10 (8.9) |
| BCLC stage |  |
| 0 | 6 (5.6) |
| A | 45 (42.1) |
| B | 17 (15.9) |
| C | 35 (32.7) |
| D | 4 (3.7) |
| mUICC stage |  |
| I | 10 (9.3) |
| II | 39 (36.4) |
| III | 32 (29.9) |
| IVA | 15 (14.0) |
| IVB | 11 (0.3) |

| Treatment |  |
| --- | --- |
| Hepatectomy | 35 (32.7) |
| RFA | 4 (3.7) |
| TACE | 30 (28.0) |
| Sorafenib | 15 (14.0) |
| Chemotherapy | 6 (5.6) |
| Conservative or unknown | 17 (15.9) |

**Supplementary Table S2.** Association Between the Patients’ Clinical Variables and the Presence of 12 Genes Mutations Using Targeted Deep Sequencing. AFP, alpha-fetoprotein; BCLC, Barcelona Clinic Liver CancerMELD, model for end-stage liver disease; mUICC, modified Union for International Cancer Control; PIVKA-II, Protein induced by vitamin K absence-II. ^a^*P* <.05

| **Total Set (n = 59)** | ***CDKN2A*** | ***CTNNB1*** | ***EGFR*** | ***KIT*** | ***MLH1*** | ***NFE2L2*** | ***NPM1*** | ***PTEN*** | ***SMO*** | ***STK11*** | ***TP53*** | ***VHL*** |
| --- | --- | --- | --- | --- | --- | --- | --- | --- | --- | --- | --- | --- |
| Gender (male) | *P* = .491 | *P* = .321 | *P* = .035^a^ | *P* = .62 | *P* = .896 | *P* = .629 | *P* = .629 | *P* = .095 | *P* = 0.629 | *P* = .403 | *P* = .783 | *P* = .035^a^ |
| Age (>50 years) | *P* = .36 | *P* = .188 | *P* = .521 | *P* = .273 | *P* = .031^a^ | *P* = .521 | *P* = .113 | *P* = .862 | *P* = 0.113 | *P* = .234 | *P* = .018^a^ | *P* = .521 |
| Etiology | *P* = .68 | *P* = .74 | *P* = .001^a^ | *P* = .221 | *P* = .855 | *P* = .828 | *P* = .828 | *P* = .436 | *P* = 0.828 | *P* = .955 | *P* = .444 | *P* = .083 |
| Child Pugh class | *P* = .012^a^ | *P* = .545 | *P* = .866 | *P* = .571 | *P* = .833 | *P* = .00^a^ | *P* = .866 | *P* = .13 | *P* = 0.866 | *P* = .955 | *P* = .059 | *P* = .866 |
| MELD | *P* = .715 | *P* = .715 | *P* = .432 | *P* = .51 | *P* = .121 | *P* = .195 | *P* = .195 | *P* = .599 | - | *P* = .244 | *P* = .332 | *P* = .432 |
| AFP (>200 ng/mL | *P* = .705 | *P* = .586 | *P* = .437 | *P* = .989 | *P* = .833 | *P* = .191 | *P* = .191 | *P* = .599 | *P* = 0.437 | *P* = .42 | *P* = .108 | *P* = .437 |
| PIVKA-II (>40 mAU/mL) | *P* = .331 | *P* = .228 | *P* = .132 | *P* = .569 | *P* = .569 | *P* = .497 | - | *P* = .159 | *P* = 0.132 | *P* = .782 | *P* = .053 | *P* = .497 |
| Vascular invasion | *P* = .584 | *P* = .749 | *P* = .487 | *P* = .639 | *P* = .95 | *P* = .143 | *P* = .143 | *P* = .749 | *P* = 0.487 | *P* = .25 | *P* = .16 | *P* = .487 |
| BCLC stage | *P* = .025* | *P* = .939 | *P* = .289 | *P* = .357 | *P* = .271 | *P* =.001^a^ | *P* = .81 | *P* = .018^a^ | *P* = 0.765 | *P* = .555 | *P* = .299 | *P* = .81 |
| mUICC stage | *P* = .408 | *P* = .66 | *P* = .642 | *P* = .819 | *P* = .679 | *P* = .109 | *P* = .35 | *P* = .363 | *P* = 0.642 | *P* = .543 | *P* = .516 | *P* = .678 |

**Supplementary Table S3.** Association Between clinical parameters and *MLH1, PTEN, STK11*, and *CTNNB1* SNVs. AFP, alpha-fetoprotein; BCLC, Barcelona Clinic Liver CancerMELD, model for end-stage liver disease; mUICC, modified Union for International Cancer Control; PIVKA-II, Protein induced by vitamin K absence-II. ^a^*P* <.05

| Total Set (n = 107) | *MLH1* | *STK11* | *PTEN* | *CTNNB1* | *4 Genes* |
| --- | --- | --- | --- | --- | --- |
| Gender (male) | *P* = .811 | *P* = .088 | *P* = .183 | *P* = .344 | *P* = .138 |
| Age (>50 years) | *P* = .624 | *P* = .493 | *P* = .783 | *P* = .236 | *P* = .971 |
| Etiology | *P* = .731 | *P* = .956 | *P* = .41 | *P* = .788 | *P* = .497 |
| Child Pugh class | *P* = .735 | *P* = .605 | *P* = .111 | *P* = .657 | *P* = .669 |
| MELD | *P* = .794 | *P* = .807 | *P* = .903 | *P* = .865 | *P* = .508 |
| AFP (>200 ng/mL | *P* = .092 | *P* = .301 | *P* = .434 | *P* = .566 | *P* = .771 |
| PIVKA-II (>40 mAU/mL) | *P* = .662 | *P* = .715 | *P* = .594 | *P* = .241 | *P* = .731 |
| Vascular invasion | *P* = .671 | *P* = .352 | *P* = .682 | *P* = .89 | *P* = .354 |
| BCLC stage | *P* = .025^a^ | *P* = .735 | *P* = .072 | *P* = .866 | *P* = .097 |

**Supplementary Table S4. Customized Panel Targeting 2924 SNVs in 69 Genes**

| No. | Gene | Accession_number | COSMIC_id | CDS_mut_syntax | AA_mut_syntax | Strand | HG19_coordinates |
| --- | --- | --- | --- | --- | --- | --- | --- |
| 1 | VHL | NM_000551.2 | 14279 | c.405delA | p.L135fs*24 | + | 3:10188262-10188262 |
| 2 | VHL | NM_000551.2 | 14284 | c.429C>G | p.D143E | + | 3:10188286-10188286 |
| 3 | VHL | NM_000551.2 | 14302 | c.391delA | p.N131fs*28 | + | 3:10188248-10188248 |
| 4 | VHL | NM_000551.2 | 14305 | c.266T>A | p.L89H | + | 3:10183797-10183797 |
| 5 | VHL | NM_000551.2 | 14307 | c.496G>A | p.V166I | + | 3:10191503-10191503 |
| 6 | VHL | NM_000551.2 | 14309 | c.478G>A | p.E160K | + | 3:10191485-10191485 |
| 7 | VHL | NM_000551.2 | 14310 | c.349T>C | p.W117R | + | 3:10188206-10188206 |
| 8 | VHL | NM_000551.2 | 14311 | c.499C>T | p.R167W | + | 3:10191506-10191506 |
| 9 | VHL | NM_000551.2 | 14312 | c.353T>C | p.L118P | + | 3:10188210-10188210 |
| 10 | VHL | NM_000551.2 | 14325 | c.294C>G | p.Y98* | + | 3:10183825-10183825 |
| 11 | VHL | NM_000551.2 | 14328 | c.351G>T | p.W117C | + | 3:10188208-10188208 |
| 12 | VHL | NM_000551.2 | 14346 | c.266T>C | p.L89P | + | 3:10183797-10183797 |
| 13 | VHL | NM_000551.2 | 14348 | c.301C>T | p.L101L | + | 3:10183832-10183832 |
| 14 | VHL | NM_000551.2 | 14349 | c.259_264delGTATGG | p.V87_W88del | + | 3:10183790-10183795 |
| 15 | VHL | NM_000551.2 | 14355 | c.488T>C | p.L163P | + | 3:10191495-10191495 |
| 16 | VHL | NM_000551.2 | 14356 | c.394C>T | p.Q132* | + | 3:10188251-10188251 |
| 17 | VHL | NM_000551.2 | 14362 | c.383T>G | p.L128R | + | 3:10188240-10188240 |
| 18 | VHL | NM_000551.2 | 14368 | c.473T>A | p.L158Q | + | 3:10191480-10191480 |
| 19 | VHL | NM_000551.2 | 14373 | c.400G>T | p.E134* | + | 3:10188257-10188257 |
| 20 | VHL | NM_000551.2 | 14374 | c.513G>C | p.K171N | + | 3:10191520-10191520 |
| 21 | VHL | NM_000551.2 | 14375 | c.343C>T | p.H115Y | + | 3:10188200-10188200 |
| 22 | VHL | NM_000551.2 | 14376 | c.464-1G>A | p.? | + | 3:10191470-10191470 |
| 23 | VHL | NM_000551.2 | 14382 | c.245G>C | p.R82P | + | 3:10183776-10183776 |
| 24 | VHL | NM_000551.2 | 14383 | c.413C>G | p.P138R | + | 3:10188270-10188270 |
| 25 | VHL | NM_000551.2 | 14386 | c.309delT | p.G104fs*55 | + | 3:10183840-10183840 |
| 26 | VHL | NM_000551.2 | 14387 | c.473T>C | p.L158P | + | 3:10191480-10191480 |
| 27 | VHL | NM_000551.2 | 14390 | c.269A>T | p.N90I | + | 3:10183800-10183800 |
| 28 | VHL | NM_000551.2 | 14391 | c.469delA | p.T157fs*2 | + | 3:10191476-10191476 |
| 29 | VHL | NM_000551.2 | 14392 | c.377A>G | p.D126G | + | 3:10188234-10188234 |
| 30 | VHL | NM_000551.2 | 14393 | c.311delG | p.G104fs*55 | + | 3:10183842-10183842 |
| 31 | VHL | NM_000551.2 | 14397 | c.490C>T | p.Q164* | + | 3:10191497-10191497 |
| 32 | VHL | NM_000551.2 | 14399 | c.351G>A | p.W117* | + | 3:10188208-10188208 |
| 33 | VHL | NM_000551.2 | 14404 | c.266T>G | p.L89R | + | 3:10183797-10183797 |
| 34 | VHL | NM_000551.2 | 14405 | c.257C>A | p.P86H | + | 3:10183788-10183788 |
| 35 | VHL | NM_000551.2 | 14406 | c.404delT | p.L135fs*24 | + | 3:10188261-10188261 |
| 36 | VHL | NM_000551.2 | 14407 | c.388G>C | p.V130L | + | 3:10188245-10188245 |
| 37 | VHL | NM_000551.2 | 14408 | c.485G>A | p.C162Y | + | 3:10191492-10191492 |
| 38 | VHL | NM_000551.2 | 14410 | c.440delT | p.F148fs*11 | + | 3:10188297-10188297 |
| 39 | VHL | NM_000551.2 | 14412 | c.431delG | p.G144fs*15 | + | 3:10188288-10188288 |
| 40 | VHL | NM_000551.2 | 14413 | c.426_429delTGAC | p.G144fs*14 | + | 3:10188283-10188286 |
| 41 | VHL | NM_000551.2 | 14414 | c.310delG | p.G104fs*55 | + | 3:10183841-10183841 |
| 42 | VHL | NM_000551.2 | 14415 | c.381delG | p.L128fs*31 | + | 3:10188238-10188238 |
| 43 | VHL | NM_000551.2 | 14419 | c.488delT | p.L163fs*7 | + | 3:10191495-10191495 |
| 44 | VHL | NM_000551.2 | 14437 | c.464-1G>C | p.? | + | 3:10191470-10191470 |
| 45 | VHL | NM_000551.2 | 144971 | c.244C>G | p.R82G | + | 3:10183775-10183775 |
| 46 | VHL | NM_000551.2 | 17612 | c.481C>T | p.R161* | + | 3:10191488-10191488 |
| 47 | VHL | NM_000551.2 | 17644 | c.363T>G | p.D121E | + | 3:10188220-10188220 |
| 48 | VHL | NM_000551.2 | 17647 | c.414delA | p.S139fs*20 | + | 3:10188271-10188271 |
| 49 | VHL | NM_000551.2 | 17648 | c.423delT | p.N141fs*18 | + | 3:10188280-10188280 |
| 50 | VHL | NM_000551.2 | 17649 | c.436delC | p.P146fs*13 | + | 3:10188293-10188293 |
| 51 | VHL | NM_000551.2 | 17657 | c.472C>G | p.L158V | + | 3:10191479-10191479 |
| 52 | VHL | NM_000551.2 | 17658 | c.286C>T | p.Q96* | + | 3:10183817-10183817 |
| 53 | VHL | NM_000551.2 | 17662 | c.492G>T | p.Q164H | + | 3:10191499-10191499 |
| 54 | VHL | NM_000551.2 | 17676 | c.498delC | p.R167fs*3 | + | 3:10191505-10191505 |
| 55 | VHL | NM_000551.2 | 17678 | c.344delA | p.H115fs*44 | + | 3:10188201-10188201 |
| 56 | VHL | NM_000551.2 | 17699 | c.284C>G | p.P95R | + | 3:10183815-10183815 |
| 57 | VHL | NM_000551.2 | 17721 | c.241C>T | p.P81S | + | 3:10183772-10183772 |
| 58 | VHL | NM_000551.2 | 17735 | c.444delT | p.F148fs*11 | + | 3:10188301-10188301 |
| 59 | VHL | NM_000551.2 | 17752 | c.343C>A | p.H115N | + | 3:10188200-10188200 |
| 60 | VHL | NM_000551.2 | 17757 | c.390delT | p.N131fs*28 | + | 3:10188247-10188247 |
| 61 | VHL | NM_000551.2 | 17759 | c.393_396delCCAA | p.N131fs*27 | + | 3:10188250-10188253 |
| 62 | VHL | NM_000551.2 | 17764 | c.408delT | p.F136fs*23 | + | 3:10188265-10188265 |
| 63 | VHL | NM_000551.2 | 17765 | c.409delG | p.V137fs*22 | + | 3:10188266-10188266 |
| 64 | VHL | NM_000551.2 | 17767 | c.417T>A | p.S139S | + | 3:10188274-10188274 |
| 65 | VHL | NM_000551.2 | 17769 | c.437delC | p.P146fs*13 | + | 3:10188294-10188294 |
| 66 | VHL | NM_000551.2 | 17770 | c.501delG | p.S168fs*2 | + | 3:10191508-10191508 |
| 67 | VHL | NM_000551.2 | 17786 | c.497T>G | p.V166G | + | 3:10191504-10191504 |
| 68 | VHL | NM_000551.2 | 17805 | c.471T>A | p.T157T | + | 3:10191478-10191478 |
| 69 | VHL | NM_000551.2 | 17807 | c.264G>C | p.W88C | + | 3:10183795-10183795 |
| 70 | VHL | NM_000551.2 | 17837 | c.506T>C | p.L169P | + | 3:10191513-10191513 |
| 71 | VHL | NM_000551.2 | 17844 | c.464delT | p.V155fs*4 | + | 3:10191471-10191471 |
| 72 | VHL | NM_000551.2 | 17857 | c.395delA | p.T133fs*26 | + | 3:10188252-10188252 |
| 73 | VHL | NM_000551.2 | 17859 | c.254T>C | p.L85P | + | 3:10183785-10183785 |
| 74 | VHL | NM_000551.2 | 17862 | c.350G>A | p.W117* | + | 3:10188207-10188207 |
| 75 | VHL | NM_000551.2 | 17881 | c.262T>A | p.W88R | + | 3:10183793-10183793 |
| 76 | VHL | NM_000551.2 | 17882 | c.269delA | p.N90fs*69 | + | 3:10183800-10183800 |
| 77 | VHL | NM_000551.2 | 17885 | c.293A>T | p.Y98F | + | 3:10183824-10183824 |
| 78 | VHL | NM_000551.2 | 17886 | c.296delC | p.P99fs*60 | + | 3:10183827-10183827 |
| 79 | VHL | NM_000551.2 | 17895 | c.369delG | p.T124fs*35 | + | 3:10188226-10188226 |
| 80 | VHL | NM_000551.2 | 17904 | c.422_423insA | p.N141fs*3 | + | 3:10188279-10188280 |
| 81 | VHL | NM_000551.2 | 17909 | c.482G>C | p.R161P | + | 3:10191489-10191489 |
| 82 | VHL | NM_000551.2 | 17913 | c.497T>A | p.V166D | + | 3:10191504-10191504 |
| 83 | VHL | NM_000551.2 | 17953 | c.262T>C | p.W88R | + | 3:10183793-10183793 |
| 84 | VHL | NM_000551.2 | 17956 | c.291delC | p.Y98fs*61 | + | 3:10183822-10183822 |
| 85 | VHL | NM_000551.2 | 17958 | c.294C>A | p.Y98* | + | 3:10183825-10183825 |
| 86 | VHL | NM_000551.2 | 17967 | c.365C>A | p.A122E | + | 3:10188222-10188222 |
| 87 | VHL | NM_000551.2 | 17974 | c.438T>C | p.P146P | + | 3:10188295-10188295 |
| 88 | VHL | NM_000551.2 | 17982 | c.496G>T | p.V166F | + | 3:10191503-10191503 |
| 89 | VHL | NM_000551.2 | 17983 | c.500G>A | p.R167Q | + | 3:10191507-10191507 |
| 90 | VHL | NM_000551.2 | 17988 | c.341-1G>T | p.? | + | 3:10188197-10188197 |
| 91 | VHL | NM_000551.2 | 18009 | c.362A>G | p.D121G | + | 3:10188219-10188219 |
| 92 | VHL | NM_000551.2 | 18014 | c.405_406insT | p.V137fs*7 | + | 3:10188262-10188263 |
| 93 | VHL | NM_000551.2 | 18022 | c.357C>G | p.F119L | + | 3:10188214-10188214 |
| 94 | VHL | NM_000551.2 | 18023 | c.486C>G | p.C162W | + | 3:10191493-10191493 |
| 95 | VHL | NM_000551.2 | 18025 | c.470C>T | p.T157I | + | 3:10191477-10191477 |
| 96 | VHL | NM_000551.2 | 18028 | c.257C>T | p.P86L | + | 3:10183788-10183788 |
| 97 | VHL | NM_000551.2 | 18051 | c.424delG | p.V142fs*17 | + | 3:10188281-10188281 |
| 98 | VHL | NM_000551.2 | 18059 | c.406delT | p.F136fs*23 | + | 3:10188263-10188263 |
| 99 | VHL | NM_000551.2 | 18070 | c.263G>A | p.W88* | + | 3:10183794-10183794 |
| 100 | VHL | NM_000551.2 | 18073 | c.484T>C | p.C162R | + | 3:10191491-10191491 |
| 101 | VHL | NM_000551.2 | 18075 | c.478G>T | p.E160* | + | 3:10191485-10191485 |
| 102 | VHL | NM_000551.2 | 18080 | c.277G>C | p.G93R | + | 3:10183808-10183808 |
| 103 | VHL | NM_000551.2 | 18192 | c.350G>T | p.W117L | + | 3:10188207-10188207 |
| 104 | VHL | NM_000551.2 | 18212 | c.491A>C | p.Q164P | + | 3:10191498-10191498 |
| 105 | VHL | NM_000551.2 | 18215 | c.426delT | p.V142fs*17 | + | 3:10188283-10188283 |
| 106 | VHL | NM_000551.2 | 18255 | c.302T>C | p.L101P | + | 3:10183833-10183833 |
| 107 | VHL | NM_000551.2 | 18276 | c.406T>G | p.F136V | + | 3:10188263-10188263 |
| 108 | VHL | NM_000551.2 | 18286 | c.390_391insT | p.N131fs*2 | + | 3:10188247-10188248 |
| 109 | VHL | NM_000551.2 | 18290 | c.341-1G>A | p.? | + | 3:10188197-10188197 |
| 110 | VHL | NM_000551.2 | 18346 | c.341G>C | p.G114A | + | 3:10188198-10188198 |
| 111 | VHL | NM_000551.2 | 18350 | c.256C>T | p.P86S | + | 3:10183787-10183787 |
| 112 | VHL | NM_000551.2 | 18379 | c.505C>T | p.L169L | + | 3:10191512-10191512 |
| 113 | VHL | NM_000551.2 | 18415 | c.263G>C | p.W88S | + | 3:10183794-10183794 |
| 114 | VHL | NM_000551.2 | 22469 | c.467delA | p.Y156fs*3 | + | 3:10191474-10191474 |
| 115 | VHL | NM_000551.2 | 249586 | c.412_425delCCATCTCTCAATGT | p.P138fs*1 | + | 3:10188269-10188282 |
| 116 | VHL | NM_000551.2 | 25675 | c.464-1G>T | p.? | + | 3:10191470-10191470 |
| 117 | VHL | NM_000551.2 | 25676 | c.485G>T | p.C162F | + | 3:10191492-10191492 |
| 118 | VHL | NM_000551.2 | 25682 | c.430G>T | p.G144* | + | 3:10188287-10188287 |
| 119 | VHL | NM_000551.2 | 25719 | c.383T>C | p.L128P | + | 3:10188240-10188240 |
| 120 | VHL | NM_000551.2 | 25720 | c.393C>G | p.N131K | + | 3:10188250-10188250 |
| 121 | VHL | NM_000551.2 | 26785 | c.263delG | p.W88fs*71 | + | 3:10183794-10183794 |
| 122 | VHL | NM_000551.2 | 30295 | c.341-2A>G | p.? | + | 3:10188196-10188196 |
| 123 | VHL | NM_000551.2 | 34019 | c.361G>T | p.D121Y | + | 3:10188218-10188218 |
| 124 | VHL | NM_000551.2 | 34030 | c.477delA | p.E160fs*10 | + | 3:10191484-10191484 |
| 125 | TRIM33 | NM_015906 |  | c.14>15 |  | - | 1:114970369-114973534 |
| 126 | TPM3 | NM_153649 |  | c.8>9 |  | - | 1:154148590-154163787 |
| 127 | TP53 | ENST00000545858 | 111497 | c.253delC | p.H85fs*69 | - | 17:7578398-7578398 |
| 128 | TP53 | ENST00000545858 | 111723 | c.293_295delCTC | p.P98delP | - | 17:7578275-7578277 |
| 129 | TP53 | ENST00000545858 | 116673 | c.428A>G | p.Y143C | - | 17:7577574-7577574 |
| 130 | TP53 | ENST00000545858 | 117397 | c.248G>T | p.C83F | - | 17:7578403-7578403 |
| 131 | TP53 | ENST00000545858 | 117948 | c.295C>T | p.Q99* | - | 17:7578275-7578275 |
| 132 | TP53 | ENST00000545858 | 118012 | c.313G>T | p.E105* | - | 17:7578257-7578257 |
| 133 | TP53 | ENST00000545858 | 120006 | c.463C>T | p.R155W | - | 17:7577539-7577539 |
| 134 | TP53 | ENST00000545858 | 121037 | c.454G>A | p.G152S | - | 17:7577548-7577548 |
| 135 | TP53 | ENST00000545858 | 121044 | c.238G>C | p.V80L | - | 17:7578413-7578413 |
| 136 | TP53 | ENST00000545858 | 129850 | c.256C>T | p.H86Y | - | 17:7578395-7578395 |
| 137 | TP53 | ENST00000545858 | 129854 | c.209A>G | p.Y70C | - | 17:7578442-7578442 |
| 138 | TP53 | ENST00000545858 | 129858 | c.176C>T | p.P59L | - | 17:7578475-7578475 |
| 139 | TP53 | ENST00000545858 | 131482 | c.190G>T | p.V64F | - | 17:7578461-7578461 |
| 140 | TP53 | ENST00000545858 | 131536 | c.280+1G>A | p.? | - | 17:7578370-7578370 |
| 141 | TP53 | ENST00000545858 | 165074 | c.422A>G | p.Y141C | - | 17:7577580-7577580 |
| 142 | TP53 | ENST00000545858 | 179807 | c.455G>A | p.G152D | - | 17:7577547-7577547 |
| 143 | TP53 | ENST00000545858 | 179825 | c.249C>A | p.C83* | - | 17:7578402-7578402 |
| 144 | TP53 | ENST00000545858 | 220781 | c.194G>A | p.R65H | - | 17:7578457-7578457 |
| 145 | TP53 | ENST00000545858 | 220784 | c.97T>G | p.Y33D | - | 17:7578554-7578554 |
| 146 | TP53 | ENST00000545858 | 242000 | c.359G>T | p.R120L | - | 17:7578211-7578211 |
| 147 | TP53 | ENST00000545858 | 98966 | c.238G>A | p.V80M | - | 17:7578413-7578413 |
| 148 | TP53 | ENST00000545858 | 99021 | c.464G>A | p.R155Q | - | 17:7577538-7577538 |
| 149 | TP53 | ENST00000545858 | 99024 | c.245G>A | p.R82H | - | 17:7578406-7578406 |
| 150 | TP53 | ENST00000545858 | 99600 | c.125G>A | p.C42Y | - | 17:7578526-7578526 |
| 151 | TP53 | ENST00000545858 | 99617 | c.358C>T | p.R120* | - | 17:7578212-7578212 |
| 152 | TP53 | ENST00000545858 | 99625 | c.434G>T | p.C145F | - | 17:7577568-7577568 |
| 153 | TP53 | ENST00000545858 | 99640 | c.238G>T | p.V80L | - | 17:7578413-7578413 |
| 154 | TP53 | ENST00000545858 | 99647 | c.432G>A | p.M144I | - | 17:7577570-7577570 |
| 155 | TP53 | ENST00000545858 | 99667 | c.307C>T | p.R103* | - | 17:7578263-7578263 |
| 156 | TP53 | ENST00000545858 | 99719 | c.380A>G | p.Y127C | - | 17:7578190-7578190 |
| 157 | TP53 | ENST00000545858 | 99918 | c.299A>T | p.H100L | - | 17:7578271-7578271 |
| 158 | TP53 | ENST00000545858 | 99945 | c.99C>G | p.Y33* | - | 17:7578552-7578552 |
| 159 | TP53 | ENST00000414315 | 111496 | c.136delC | p.H46fs*>45 | - | 17:7578398-7578398 |
| 160 | TP53 | ENST00000414315 | 111722 | c.176_178delCTC | p.P59delP | - | 17:7578275-7578277 |
| 161 | TP53 | ENST00000414315 | 117396 | c.131G>T | p.C44F | - | 17:7578403-7578403 |
| 162 | TP53 | ENST00000414315 | 117947 | c.178C>T | p.Q60* | - | 17:7578275-7578275 |
| 163 | TP53 | ENST00000414315 | 118011 | c.196G>T | p.E66* | - | 17:7578257-7578257 |
| 164 | TP53 | ENST00000414315 | 121045 | c.121G>C | p.V41L | - | 17:7578413-7578413 |
| 165 | TP53 | ENST00000414315 | 129851 | c.139C>T | p.H47Y | - | 17:7578395-7578395 |
| 166 | TP53 | ENST00000414315 | 129855 | c.92A>G | p.Y31C | - | 17:7578442-7578442 |
| 167 | TP53 | ENST00000414315 | 129859 | c.59C>T | p.P20L | - | 17:7578475-7578475 |
| 168 | TP53 | ENST00000414315 | 131483 | c.73G>T | p.V25F | - | 17:7578461-7578461 |
| 169 | TP53 | ENST00000414315 | 131537 | c.163+1G>A | p.? | - | 17:7578370-7578370 |
| 170 | TP53 | ENST00000414315 | 179824 | c.132C>A | p.C44* | - | 17:7578402-7578402 |
| 171 | TP53 | ENST00000414315 | 220780 | c.77G>A | p.R26H | - | 17:7578457-7578457 |
| 172 | TP53 | ENST00000414315 | 241999 | c.242G>T | p.R81L | - | 17:7578211-7578211 |
| 173 | TP53 | ENST00000414315 | 98965 | c.121G>A | p.V41M | - | 17:7578413-7578413 |
| 174 | TP53 | ENST00000414315 | 99023 | c.128G>A | p.R43H | - | 17:7578406-7578406 |
| 175 | TP53 | ENST00000414315 | 99599 | c.8G>A | p.C3Y | - | 17:7578526-7578526 |
| 176 | TP53 | ENST00000414315 | 99616 | c.241C>T | p.R81* | - | 17:7578212-7578212 |
| 177 | TP53 | ENST00000414315 | 99639 | c.121G>T | p.V41L | - | 17:7578413-7578413 |
| 178 | TP53 | ENST00000414315 | 99666 | c.190C>T | p.R64* | - | 17:7578263-7578263 |
| 179 | TP53 | ENST00000414315 | 99917 | c.182A>T | p.H61L | - | 17:7578271-7578271 |
| 180 | TP53 | ENST00000413465 | 111495 | c.532delC | p.H178fs*69 | - | 17:7578398-7578398 |
| 181 | TP53 | ENST00000413465 | 111721 | c.572_574delCTC | p.P191delP | - | 17:7578275-7578277 |
| 182 | TP53 | ENST00000413465 | 116672 | c.707A>G | p.Y236C | - | 17:7577574-7577574 |
| 183 | TP53 | ENST00000413465 | 117395 | c.527G>T | p.C176F | - | 17:7578403-7578403 |
| 184 | TP53 | ENST00000413465 | 117946 | c.574C>T | p.Q192* | - | 17:7578275-7578275 |
| 185 | TP53 | ENST00000413465 | 118010 | c.592G>T | p.E198* | - | 17:7578257-7578257 |
| 186 | TP53 | ENST00000413465 | 120005 | c.742C>T | p.R248W | - | 17:7577539-7577539 |
| 187 | TP53 | ENST00000413465 | 121036 | c.733G>A | p.G245S | - | 17:7577548-7577548 |
| 188 | TP53 | ENST00000413465 | 121043 | c.517G>C | p.V173L | - | 17:7578413-7578413 |
| 189 | TP53 | ENST00000413465 | 129849 | c.535C>T | p.H179Y | - | 17:7578395-7578395 |
| 190 | TP53 | ENST00000413465 | 129853 | c.488A>G | p.Y163C | - | 17:7578442-7578442 |
| 191 | TP53 | ENST00000413465 | 129857 | c.455C>T | p.P152L | - | 17:7578475-7578475 |
| 192 | TP53 | ENST00000413465 | 131479 | c.747G>T | p.R249S | - | 17:7577534-7577534 |
| 193 | TP53 | ENST00000413465 | 131481 | c.469G>T | p.V157F | - | 17:7578461-7578461 |
| 194 | TP53 | ENST00000413465 | 131535 | c.559+1G>A | p.? | - | 17:7578370-7578370 |
| 195 | TP53 | ENST00000413465 | 165072 | c.701A>G | p.Y234C | - | 17:7577580-7577580 |
| 196 | TP53 | ENST00000413465 | 179805 | c.734G>A | p.G245D | - | 17:7577547-7577547 |
| 197 | TP53 | ENST00000413465 | 179822 | c.528C>A | p.C176* | - | 17:7578402-7578402 |
| 198 | TP53 | ENST00000413465 | 220765 | c.319T>G | p.Y107D | - | 17:7579368-7579368 |
| 199 | TP53 | ENST00000413465 | 220778 | c.473G>A | p.R158H | - | 17:7578457-7578457 |
| 200 | TP53 | ENST00000413465 | 220782 | c.376T>G | p.Y126D | - | 17:7578554-7578554 |
| 201 | TP53 | ENST00000413465 | 241997 | c.638G>T | p.R213L | - | 17:7578211-7578211 |
| 202 | TP53 | ENST00000413465 | 98964 | c.517G>A | p.V173M | - | 17:7578413-7578413 |
| 203 | TP53 | ENST00000413465 | 99020 | c.743G>A | p.R248Q | - | 17:7577538-7577538 |
| 204 | TP53 | ENST00000413465 | 99022 | c.524G>A | p.R175H | - | 17:7578406-7578406 |
| 205 | TP53 | ENST00000413465 | 99598 | c.404G>A | p.C135Y | - | 17:7578526-7578526 |
| 206 | TP53 | ENST00000413465 | 99615 | c.637C>T | p.R213* | - | 17:7578212-7578212 |
| 207 | TP53 | ENST00000413465 | 99624 | c.713G>T | p.C238F | - | 17:7577568-7577568 |
| 208 | TP53 | ENST00000413465 | 99638 | c.517G>T | p.V173L | - | 17:7578413-7578413 |
| 209 | TP53 | ENST00000413465 | 99646 | c.711G>A | p.M237I | - | 17:7577570-7577570 |
| 210 | TP53 | ENST00000413465 | 99665 | c.586C>T | p.R196* | - | 17:7578263-7578263 |
| 211 | TP53 | ENST00000413465 | 99718 | c.659A>G | p.Y220C | - | 17:7578190-7578190 |
| 212 | TP53 | ENST00000413465 | 99916 | c.578A>T | p.H193L | - | 17:7578271-7578271 |
| 213 | TP53 | ENST00000413465 | 99928 | c.329G>T | p.R110L | - | 17:7579358-7579358 |
| 214 | TP53 | ENST00000413465 | 99944 | c.378C>G | p.Y126* | - | 17:7578552-7578552 |
| 215 | TP53 | ENST00000269305 | 111498 | c.532delC | p.H178fs*69 | - | 17:7578398-7578398 |
| 216 | TP53 | ENST00000269305 | 111724 | c.572_574delCTC | p.P191delP | - | 17:7578275-7578277 |
| 217 | TP53 | ENST00000269305 | 116674 | c.707A>G | p.Y236C | - | 17:7577574-7577574 |
| 218 | TP53 | ENST00000269305 | 117398 | c.527G>T | p.C176F | - | 17:7578403-7578403 |
| 219 | TP53 | ENST00000269305 | 117949 | c.574C>T | p.Q192* | - | 17:7578275-7578275 |
| 220 | TP53 | ENST00000269305 | 118013 | c.592G>T | p.E198* | - | 17:7578257-7578257 |
| 221 | TP53 | ENST00000269305 | 120007 | c.742C>T | p.R248W | - | 17:7577539-7577539 |
| 222 | TP53 | ENST00000269305 | 121035 | c.733G>A | p.G245S | - | 17:7577548-7577548 |
| 223 | TP53 | ENST00000269305 | 121042 | c.517G>C | p.V173L | - | 17:7578413-7578413 |
| 224 | TP53 | ENST00000269305 | 126981 | c.880G>T | p.E294* | - | 17:7577058-7577058 |
| 225 | TP53 | ENST00000269305 | 129830 | c.839G>A | p.R280K | - | 17:7577099-7577099 |
| 226 | TP53 | ENST00000269305 | 129831 | c.833C>T | p.P278L | - | 17:7577105-7577105 |
| 227 | TP53 | ENST00000269305 | 129848 | c.535C>T | p.H179Y | - | 17:7578395-7578395 |
| 228 | TP53 | ENST00000269305 | 129852 | c.488A>G | p.Y163C | - | 17:7578442-7578442 |
| 229 | TP53 | ENST00000269305 | 129856 | c.455C>T | p.P152L | - | 17:7578475-7578475 |
| 230 | TP53 | ENST00000269305 | 131478 | c.747G>T | p.R249S | - | 17:7577534-7577534 |
| 231 | TP53 | ENST00000269305 | 131480 | c.469G>T | p.V157F | - | 17:7578461-7578461 |
| 232 | TP53 | ENST00000269305 | 131534 | c.559+1G>A | p.? | - | 17:7578370-7578370 |
| 233 | TP53 | ENST00000269305 | 137087 | c.853G>A | p.E285K | - | 17:7577085-7577085 |
| 234 | TP53 | ENST00000269305 | 139044 | c.832C>T | p.P278S | - | 17:7577106-7577106 |
| 235 | TP53 | ENST00000269305 | 165073 | c.701A>G | p.Y234C | - | 17:7577580-7577580 |
| 236 | TP53 | ENST00000269305 | 165075 | c.820G>T | p.V274F | - | 17:7577118-7577118 |
| 237 | TP53 | ENST00000269305 | 165084 | c.824G>A | p.C275Y | - | 17:7577114-7577114 |
| 238 | TP53 | ENST00000269305 | 179804 | c.799C>T | p.R267W | - | 17:7577139-7577139 |
| 239 | TP53 | ENST00000269305 | 179806 | c.734G>A | p.G245D | - | 17:7577547-7577547 |
| 240 | TP53 | ENST00000269305 | 179823 | c.528C>A | p.C176* | - | 17:7578402-7578402 |
| 241 | TP53 | ENST00000269305 | 220766 | c.319T>G | p.Y107D | - | 17:7579368-7579368 |
| 242 | TP53 | ENST00000269305 | 220779 | c.473G>A | p.R158H | - | 17:7578457-7578457 |
| 243 | TP53 | ENST00000269305 | 220783 | c.376T>G | p.Y126D | - | 17:7578554-7578554 |
| 244 | TP53 | ENST00000269305 | 241998 | c.638G>T | p.R213L | - | 17:7578211-7578211 |
| 245 | TP53 | ENST00000269305 | 99601 | c.404G>A | p.C135Y | - | 17:7578526-7578526 |
| 246 | TP53 | ENST00000269305 | 99602 | c.743G>A | p.R248Q | - | 17:7577538-7577538 |
| 247 | TP53 | ENST00000269305 | 99618 | c.637C>T | p.R213* | - | 17:7578212-7578212 |
| 248 | TP53 | ENST00000269305 | 99626 | c.713G>T | p.C238F | - | 17:7577568-7577568 |
| 249 | TP53 | ENST00000269305 | 99641 | c.517G>T | p.V173L | - | 17:7578413-7578413 |
| 250 | TP53 | ENST00000269305 | 99648 | c.711G>A | p.M237I | - | 17:7577570-7577570 |
| 251 | TP53 | ENST00000269305 | 99668 | c.586C>T | p.R196* | - | 17:7578263-7578263 |
| 252 | TP53 | ENST00000269305 | 99720 | c.659A>G | p.Y220C | - | 17:7578190-7578190 |
| 253 | TP53 | ENST00000269305 | 99721 | c.1024C>T | p.R342* | - | 17:7574003-7574003 |
| 254 | TP53 | ENST00000269305 | 99725 | c.832C>G | p.P278A | - | 17:7577106-7577106 |
| 255 | TP53 | ENST00000269305 | 99729 | c.818G>A | p.R273H | - | 17:7577120-7577120 |
| 256 | TP53 | ENST00000269305 | 99914 | c.524G>A | p.R175H | - | 17:7578406-7578406 |
| 257 | TP53 | ENST00000269305 | 99919 | c.578A>T | p.H193L | - | 17:7578271-7578271 |
| 258 | TP53 | ENST00000269305 | 99924 | c.856G>A | p.E286K | - | 17:7577082-7577082 |
| 259 | TP53 | ENST00000269305 | 99925 | c.844C>T | p.R282W | - | 17:7577094-7577094 |
| 260 | TP53 | ENST00000269305 | 99929 | c.329G>T | p.R110L | - | 17:7579358-7579358 |
| 261 | TP53 | ENST00000269305 | 99932 | c.824G>T | p.C275F | - | 17:7577114-7577114 |
| 262 | TP53 | ENST00000269305 | 99933 | c.817C>T | p.R273C | - | 17:7577121-7577121 |
| 263 | TP53 | ENST00000269305 | 99946 | c.378C>G | p.Y126* | - | 17:7578552-7578552 |
| 264 | TP53 | ENST00000269305 | 99947 | c.916C>T | p.R306* | - | 17:7577022-7577022 |
| 265 | TP53 | ENST00000269305 | 99950 | c.814G>A | p.V272M | - | 17:7577124-7577124 |
| 266 | TP53 | ENST00000269305 | 99952 | c.797G>T | p.G266V | - | 17:7577141-7577141 |
| 267 | TP53 | NM_000546 | 10645 | c.527G>T | p.C176F | - | 17:7578403-7578403 |
| 268 | TP53 | NM_000546 | 10646 | c.725G>A | p.C242Y | - | 17:7577556-7577556 |
| 269 | TP53 | NM_000546 | 10647 | c.404G>T | p.C135F | - | 17:7578526-7578526 |
| 270 | TP53 | NM_000546 | 10648 | c.524G>A | p.R175H | - | 17:7578406-7578406 |
| 271 | TP53 | NM_000546 | 10650 | c.529C>T | p.P177S | - | 17:7578401-7578401 |
| 272 | TP53 | NM_000546 | 10651 | c.530C>G | p.P177R | - | 17:7578400-7578400 |
| 273 | TP53 | NM_000546 | 10654 | c.637C>T | p.R213* | - | 17:7578212-7578212 |
| 274 | TP53 | NM_000546 | 10656 | c.742C>T | p.R248W | - | 17:7577539-7577539 |
| 275 | TP53 | NM_000546 | 10659 | c.817C>T | p.R273C | - | 17:7577121-7577121 |
| 276 | TP53 | NM_000546 | 10660 | c.818G>A | p.R273H | - | 17:7577120-7577120 |
| 277 | TP53 | NM_000546 | 10662 | c.743G>A | p.R248Q | - | 17:7577538-7577538 |
| 278 | TP53 | NM_000546 | 10663 | c.916C>T | p.R306* | - | 17:7577022-7577022 |
| 279 | TP53 | NM_000546 | 10667 | c.646G>A | p.V216M | - | 17:7578203-7578203 |
| 280 | TP53 | NM_000546 | 10668 | c.745A>G | p.R249G | - | 17:7577536-7577536 |
| 281 | TP53 | NM_000546 | 10670 | c.469G>T | p.V157F | - | 17:7578461-7578461 |
| 282 | TP53 | NM_000546 | 10672 | c.577C>T | p.H193Y | - | 17:7578272-7578272 |
| 283 | TP53 | NM_000546 | 10684 | c.403T>C | p.C135R | - | 17:7578527-7578527 |
| 284 | TP53 | NM_000546 | 10687 | c.527G>A | p.C176Y | - | 17:7578403-7578403 |
| 285 | TP53 | NM_000546 | 10690 | c.473G>A | p.R158H | - | 17:7578457-7578457 |
| 286 | TP53 | NM_000546 | 10701 | c.824G>T | p.C275F | - | 17:7577114-7577114 |
| 287 | TP53 | NM_000546 | 10704 | c.844C>T | p.R282W | - | 17:7577094-7577094 |
| 288 | TP53 | NM_000546 | 10705 | c.586C>T | p.R196* | - | 17:7578263-7578263 |
| 289 | TP53 | NM_000546 | 10706 | c.548C>G | p.S183* | - | 17:7578382-7578382 |
| 290 | TP53 | NM_000546 | 10709 | c.722C>G | p.S241C | - | 17:7577559-7577559 |
| 291 | TP53 | NM_000546 | 10710 | c.892G>T | p.E298* | - | 17:7577046-7577046 |
| 292 | TP53 | NM_000546 | 10714 | c.473G>T | p.R158L | - | 17:7578457-7578457 |
| 293 | TP53 | NM_000546 | 10715 | c.695T>A | p.I232N | - | 17:7577586-7577586 |
| 294 | TP53 | NM_000546 | 10716 | c.329G>T | p.R110L | - | 17:7579358-7579358 |
| 295 | TP53 | NM_000546 | 10718 | c.524G>T | p.R175L | - | 17:7578406-7578406 |
| 296 | TP53 | NM_000546 | 10719 | c.811G>A | p.E271K | - | 17:7577127-7577127 |
| 297 | TP53 | NM_000546 | 10722 | c.853G>A | p.E285K | - | 17:7577085-7577085 |
| 298 | TP53 | NM_000546 | 10724 | c.839G>C | p.R280T | - | 17:7577099-7577099 |
| 299 | TP53 | NM_000546 | 10725 | c.701A>G | p.Y234C | - | 17:7577580-7577580 |
| 300 | TP53 | NM_000546 | 10726 | c.856G>A | p.E286K | - | 17:7577082-7577082 |
| 301 | TP53 | NM_000546 | 10728 | c.839G>A | p.R280K | - | 17:7577099-7577099 |
| 302 | TP53 | NM_000546 | 10731 | c.707A>G | p.Y236C | - | 17:7577574-7577574 |
| 303 | TP53 | NM_000546 | 10733 | c.574C>T | p.Q192* | - | 17:7578275-7578275 |
| 304 | TP53 | NM_000546 | 10735 | c.638G>A | p.R213Q | - | 17:7578211-7578211 |
| 305 | TP53 | NM_000546 | 10738 | c.542G>A | p.R181H | - | 17:7578388-7578388 |
| 306 | TP53 | NM_000546 | 10739 | c.481G>A | p.A161T | - | 17:7578449-7578449 |
| 307 | TP53 | NM_000546 | 10742 | c.578A>G | p.H193R | - | 17:7578271-7578271 |
| 308 | TP53 | NM_000546 | 10743 | c.848G>C | p.R283P | - | 17:7577090-7577090 |
| 309 | TP53 | NM_000546 | 10749 | c.830G>T | p.C277F | - | 17:7577108-7577108 |
| 310 | TP53 | NM_000546 | 10750 | c.490A>T | p.K164* | - | 17:7578440-7578440 |
| 311 | TP53 | NM_000546 | 10756 | c.827C>T | p.A276V | - | 17:7577111-7577111 |
| 312 | TP53 | NM_000546 | 10757 | c.738G>C | p.M246I | - | 17:7577543-7577543 |
| 313 | TP53 | NM_000546 | 10758 | c.659A>G | p.Y220C | - | 17:7578190-7578190 |
| 314 | TP53 | NM_000546 | 10760 | c.467G>C | p.R156P | - | 17:7578463-7578463 |
| 315 | TP53 | NM_000546 | 10762 | c.490A>G | p.K164E | - | 17:7578440-7578440 |
| 316 | TP53 | NM_000546 | 10768 | c.535C>T | p.H179Y | - | 17:7578395-7578395 |
| 317 | TP53 | NM_000546 | 10769 | c.820G>T | p.V274F | - | 17:7577118-7577118 |
| 318 | TP53 | NM_000546 | 10770 | c.1045G>T | p.E349* | - | 17:7573982-7573982 |
| 319 | TP53 | NM_000546 | 10771 | c.749C>T | p.P250L | - | 17:7577532-7577532 |
| 320 | TP53 | NM_000546 | 10777 | c.715A>G | p.N239D | - | 17:7577566-7577566 |
| 321 | TP53 | NM_000546 | 10779 | c.818G>T | p.R273L | - | 17:7577120-7577120 |
| 322 | TP53 | NM_000546 | 10785 | c.747G>C | p.R249S | - | 17:7577534-7577534 |
| 323 | TP53 | NM_000546 | 10788 | c.764T>G | p.I255S | - | 17:7577517-7577517 |
| 324 | TP53 | NM_000546 | 10790 | c.455C>T | p.P152L | - | 17:7578475-7578475 |
| 325 | TP53 | NM_000546 | 10794 | c.796G>A | p.G266R | - | 17:7577142-7577142 |
| 326 | TP53 | NM_000546 | 10801 | c.404G>A | p.C135Y | - | 17:7578526-7578526 |
| 327 | TP53 | NM_000546 | 10804 | c.610G>T | p.E204* | - | 17:7578239-7578239 |
| 328 | TP53 | NM_000546 | 10808 | c.488A>G | p.Y163C | - | 17:7578442-7578442 |
| 329 | TP53 | NM_000546 | 10810 | c.725G>T | p.C242F | - | 17:7577556-7577556 |
| 330 | TP53 | NM_000546 | 10812 | c.722C>T | p.S241F | - | 17:7577559-7577559 |
| 331 | TP53 | NM_000546 | 10813 | c.394A>G | p.K132E | - | 17:7578536-7578536 |
| 332 | TP53 | NM_000546 | 10814 | c.832C>G | p.P278A | - | 17:7577106-7577106 |
| 333 | TP53 | NM_000546 | 10817 | c.747G>T | p.R249S | - | 17:7577534-7577534 |
| 334 | TP53 | NM_000546 | 10834 | c.711G>A | p.M237I | - | 17:7577570-7577570 |
| 335 | TP53 | NM_000546 | 10856 | c.880G>T | p.E294* | - | 17:7577058-7577058 |
| 336 | TP53 | NM_000546 | 10859 | c.814G>T | p.V272L | - | 17:7577124-7577124 |
| 337 | TP53 | NM_000546 | 10862 | c.378C>G | p.Y126* | - | 17:7578552-7578552 |
| 338 | TP53 | NM_000546 | 10863 | c.833C>T | p.P278L | - | 17:7577105-7577105 |
| 339 | TP53 | NM_000546 | 10867 | c.797G>A | p.G266E | - | 17:7577141-7577141 |
| 340 | TP53 | NM_000546 | 10870 | c.523C>G | p.R175G | - | 17:7578407-7578407 |
| 341 | TP53 | NM_000546 | 10883 | c.731G>A | p.G244D | - | 17:7577550-7577550 |
| 342 | TP53 | NM_000546 | 10886 | c.310C>T | p.Q104* | - | 17:7579377-7579377 |
| 343 | TP53 | NM_000546 | 10887 | c.833C>G | p.P278R | - | 17:7577105-7577105 |
| 344 | TP53 | NM_000546 | 10888 | c.378C>A | p.Y126* | - | 17:7578552-7578552 |
| 345 | TP53 | NM_000546 | 10889 | c.536A>G | p.H179R | - | 17:7578394-7578394 |
| 346 | TP53 | NM_000546 | 10891 | c.814G>A | p.V272M | - | 17:7577124-7577124 |
| 347 | TP53 | NM_000546 | 10893 | c.824G>A | p.C275Y | - | 17:7577114-7577114 |
| 348 | TP53 | NM_000546 | 10911 | c.847C>T | p.R283C | - | 17:7577091-7577091 |
| 349 | TP53 | NM_000546 | 10912 | c.463A>C | p.T155P | - | 17:7578467-7578467 |
| 350 | TP53 | NM_000546 | 10931 | c.751A>C | p.I251L | - | 17:7577530-7577530 |
| 351 | TP53 | NM_000546 | 10935 | c.722C>A | p.S241Y | - | 17:7577559-7577559 |
| 352 | TP53 | NM_000546 | 10939 | c.832C>T | p.P278S | - | 17:7577106-7577106 |
| 353 | TP53 | NM_000546 | 10941 | c.730G>A | p.G244S | - | 17:7577551-7577551 |
| 354 | TP53 | NM_000546 | 10943 | c.841G>C | p.D281H | - | 17:7577097-7577097 |
| 355 | TP53 | NM_000546 | 10957 | c.733G>C | p.G245R | - | 17:7577548-7577548 |
| 356 | TP53 | NM_000546 | 10958 | c.797G>T | p.G266V | - | 17:7577141-7577141 |
| 357 | TP53 | NM_000546 | 10991 | c.396G>T | p.K132N | - | 17:7578534-7578534 |
| 358 | TP53 | NM_000546 | 10992 | c.844C>G | p.R282G | - | 17:7577094-7577094 |
| 359 | TP53 | NM_000546 | 10995 | c.580C>T | p.L194F | - | 17:7578269-7578269 |
| 360 | TP53 | NM_000546 | 10996 | c.511G>T | p.E171* | - | 17:7578419-7578419 |
| 361 | TP53 | NM_000546 | 11011 | c.794T>C | p.L265P | - | 17:7577144-7577144 |
| 362 | TP53 | NM_000546 | 11059 | c.713G>A | p.C238Y | - | 17:7577568-7577568 |
| 363 | TP53 | NM_000546 | 11063 | c.711G>T | p.M237I | - | 17:7577570-7577570 |
| 364 | TP53 | NM_000546 | 11066 | c.578A>T | p.H193L | - | 17:7578271-7578271 |
| 365 | TP53 | NM_000546 | 11071 | c.1009C>T | p.R337C | - | 17:7574018-7574018 |
| 366 | TP53 | NM_000546 | 11073 | c.1024C>T | p.R342* | - | 17:7574003-7574003 |
| 367 | TP53 | NM_000546 | 11078 | c.1027G>T | p.E343* | - | 17:7574000-7574000 |
| 368 | TP53 | NM_000546 | 11081 | c.733G>T | p.G245C | - | 17:7577548-7577548 |
| 369 | TP53 | NM_000546 | 11084 | c.517G>A | p.V173M | - | 17:7578413-7578413 |
| 370 | TP53 | NM_000546 | 11087 | c.472C>G | p.R158G | - | 17:7578458-7578458 |
| 371 | TP53 | NM_000546 | 11089 | c.584T>C | p.I195T | - | 17:7578265-7578265 |
| 372 | TP53 | NM_000546 | 11090 | c.541C>T | p.R181C | - | 17:7578389-7578389 |
| 373 | TP53 | NM_000546 | 11114 | c.528C>G | p.C176W | - | 17:7578402-7578402 |
| 374 | TP53 | NM_000546 | 11123 | c.838A>G | p.R280G | - | 17:7577100-7577100 |
| 375 | TP53 | NM_000546 | 11133 | c.725G>C | p.C242S | - | 17:7577556-7577556 |
| 376 | TP53 | NM_000546 | 11148 | c.476C>T | p.A159V | - | 17:7578454-7578454 |
| 377 | TP53 | NM_000546 | 11152 | c.700T>C | p.Y234H | - | 17:7577581-7577581 |
| 378 | TP53 | NM_000546 | 11166 | c.406C>T | p.Q136* | - | 17:7578524-7578524 |
| 379 | TP53 | NM_000546 | 11181 | c.764T>C | p.I255T | - | 17:7577517-7577517 |
| 380 | TP53 | NM_000546 | 11183 | c.799C>T | p.R267W | - | 17:7577139-7577139 |
| 381 | TP53 | NM_000546 | 11196 | c.734G>T | p.G245V | - | 17:7577547-7577547 |
| 382 | TP53 | NM_000546 | 11205 | c.796G>C | p.G266R | - | 17:7577142-7577142 |
| 383 | TP53 | NM_000546 | 11210 | c.646G>T | p.V216L | - | 17:7578203-7578203 |
| 384 | TP53 | NM_000546 | 11213 | c.752T>C | p.I251T | - | 17:7577529-7577529 |
| 385 | TP53 | NM_000546 | 11218 | c.464C>A | p.T155N | - | 17:7578466-7578466 |
| 386 | TP53 | NM_000546 | 11224 | c.394A>C | p.K132Q | - | 17:7578536-7578536 |
| 387 | TP53 | NM_000546 | 11232 | c.842A>G | p.D281G | - | 17:7577096-7577096 |
| 388 | TP53 | NM_000546 | 11244 | c.764T>A | p.I255N | - | 17:7577517-7577517 |
| 389 | TP53 | NM_000546 | 11249 | c.537T>G | p.H179Q | - | 17:7578393-7578393 |
| 390 | TP53 | NM_000546 | 11250 | c.329G>C | p.R110P | - | 17:7579358-7579358 |
| 391 | TP53 | NM_000546 | 11286 | c.1015G>T | p.E339* | - | 17:7574012-7574012 |
| 392 | TP53 | NM_000546 | 11287 | c.839G>T | p.R280I | - | 17:7577099-7577099 |
| 393 | TP53 | NM_000546 | 11290 | c.625A>T | p.R209* | - | 17:7578224-7578224 |
| 394 | TP53 | NM_000546 | 11291 | c.1006G>T | p.E336* | - | 17:7574021-7574021 |
| 395 | TP53 | NM_000546 | 11305 | c.809T>C | p.F270S | - | 17:7577129-7577129 |
| 396 | TP53 | NM_000546 | 11307 | c.643A>T | p.S215C | - | 17:7578206-7578206 |
| 397 | TP53 | NM_000546 | 11319 | c.402T>G | p.F134L | - | 17:7578528-7578528 |
| 398 | TP53 | NM_000546 | 11323 | c.482C>A | p.A161D | - | 17:7578448-7578448 |
| 399 | TP53 | NM_000546 | 11333 | c.499C>T | p.Q167* | - | 17:7578431-7578431 |
| 400 | TP53 | NM_000546 | 11351 | c.614A>T | p.Y205F | - | 17:7578235-7578235 |
| 401 | TP53 | NM_000546 | 11355 | c.737T>C | p.M246T | - | 17:7577544-7577544 |
| 402 | TP53 | NM_000546 | 11356 | c.726C>G | p.C242W | - | 17:7577555-7577555 |
| 403 | TP53 | NM_000546 | 11369 | c.492G>T | p.K164N | - | 17:7578438-7578438 |
| 404 | TP53 | NM_000546 | 11374 | c.752T>A | p.I251N | - | 17:7577529-7577529 |
| 405 | TP53 | NM_000546 | 11376 | c.737T>G | p.M246R | - | 17:7577544-7577544 |
| 406 | TP53 | NM_000546 | 11392 | c.800G>C | p.R267P | - | 17:7577138-7577138 |
| 407 | TP53 | NM_000546 | 11411 | c.1010G>T | p.R337L | - | 17:7574017-7574017 |
| 408 | TP53 | NM_000546 | 11448 | c.321C>G | p.Y107* | - | 17:7579366-7579366 |
| 409 | TP53 | NM_000546 | 11449 | c.388C>T | p.L130F | - | 17:7578542-7578542 |
| 410 | TP53 | NM_000546 | 11450 | c.644G>T | p.S215I | - | 17:7578205-7578205 |
| 411 | TP53 | NM_000546 | 11462 | c.388C>G | p.L130V | - | 17:7578542-7578542 |
| 412 | TP53 | NM_000546 | 11483 | c.848G>A | p.R283H | - | 17:7577090-7577090 |
| 413 | TP53 | NM_000546 | 11491 | c.743G>C | p.R248P | - | 17:7577538-7577538 |
| 414 | TP53 | NM_000546 | 11496 | c.476C>A | p.A159D | - | 17:7578454-7578454 |
| 415 | TP53 | NM_000546 | 11501 | c.823T>G | p.C275G | - | 17:7577115-7577115 |
| 416 | TP53 | NM_000546 | 11508 | c.497C>A | p.S166* | - | 17:7578433-7578433 |
| 417 | TP53 | NM_000546 | 11514 | c.1001G>T | p.G334V | - | 17:7574026-7574026 |
| 418 | TP53 | NM_000546 | 11516 | c.841G>T | p.D281Y | - | 17:7577097-7577097 |
| 419 | TP53 | NM_000546 | 11517 | c.377A>G | p.Y126C | - | 17:7578553-7578553 |
| 420 | TP53 | NM_000546 | 11524 | c.730G>T | p.G244C | - | 17:7577551-7577551 |
| 421 | TP53 | NM_000546 | 11542 | c.703A>G | p.N235D | - | 17:7577578-7577578 |
| 422 | TP53 | NM_000546 | 11564 | c.742C>G | p.R248G | - | 17:7577539-7577539 |
| 423 | TP53 | NM_000546 | 11582 | c.395A>G | p.K132R | - | 17:7578535-7578535 |
| 424 | TP53 | NM_000546 | 11606 | c.31G>C | p.E11Q | - | 17:7579882-7579882 |
| 425 | TP53 | NM_000546 | 11665 | c.842A>C | p.D281A | - | 17:7577096-7577096 |
| 426 | TP53 | NM_000546 | 11717 | c.548C>A | p.S183* | - | 17:7578382-7578382 |
| 427 | TP53 | NM_000546 | 11738 | c.724T>C | p.C242R | - | 17:7577557-7577557 |
| 428 | TP53 | NM_000546 | 11781 | c.398T>A | p.M133K | - | 17:7578532-7578532 |
| 429 | TP53 | NM_000546 | 11847 | c.658T>G | p.Y220D | - | 17:7578191-7578191 |
| 430 | TP53 | NM_000546 | 11860 | c.638G>C | p.R213P | - | 17:7578211-7578211 |
| 431 | TP53 | NM_000546 | 11929 | c.760_761AT>GA | p.I254D | - | 17:7577520-7577521 |
| 432 | TP53 | NM_000546 | 11966 | c.485T>A | p.I162N | - | 17:7578445-7578445 |
| 433 | TP53 | NM_000546 | 11998 | c.534C>A | p.H178Q | - | 17:7578396-7578396 |
| 434 | TP53 | NM_000546 | 12013 | c.731G>C | p.G244A | - | 17:7577550-7577550 |
| 435 | TP53 | NM_000546 | 12296 | c.292C>T | p.P98S | - | 17:7579395-7579395 |
| 436 | TP53 | NM_000546 | 13119 | c.322_324delGGT | p.G108del | - | 17:7579363-7579365 |
| 437 | TP53 | NM_000546 | 13120 | c.626_627delGA | p.R209fs*6 | - | 17:7578222-7578223 |
| 438 | TP53 | NM_000546 | 13421 | c.814delG | p.V272fs*73 | - | 17:7577124-7577124 |
| 439 | TP53 | NM_000546 | 146240 | c.806_808delGCT | p.S269_F270>I | - | 17:7577130-7577132 |
| 440 | TP53 | NM_000546 | 18597 | c.1024delC | p.R342fs*3 | - | 17:7574003-7574003 |
| 441 | TP53 | NM_000546 | 18610 | c.267delC | p.S90fs*33 | - | 17:7579424-7579424 |
| 442 | TP53 | NM_000546 | 18657 | c.560-2A>G | p.? | - | 17:7578291-7578291 |
| 443 | TP53 | NM_000546 | 21572 | c.376-1G>A | p.? | - | 17:7578555-7578555 |
| 444 | TP53 | NM_000546 | 22908 | c.376-1G>T | p.? | - | 17:7578555-7578555 |
| 445 | TP53 | NM_000546 | 249845 | c.377_377delA | p.Y126fs*44 | - | 17:7578553-7578553 |
| 446 | TP53 | NM_000546 | 39293 | c.734G>A | p.G245D | - | 17:7577547-7577547 |
| 447 | TP53 | NM_000546 | 39455 | c.569delC | p.P190fs*57 | - | 17:7578280-7578280 |
| 448 | TP53 | NM_000546 | 40942 | c.380C>T | p.S127F | - | 17:7578550-7578550 |
| 449 | TP53 | NM_000546 | 42811 | c.641A>G | p.H214R | - | 17:7578208-7578208 |
| 450 | TP53 | NM_000546 | 42813 | c.313G>T | p.G105C | - | 17:7579374-7579374 |
| 451 | TP53 | NM_000546 | 43054 | c.518T>G | p.V173G | - | 17:7578412-7578412 |
| 452 | TP53 | NM_000546 | 43533 | c.391A>T | p.N131Y | - | 17:7578539-7578539 |
| 453 | TP53 | NM_000546 | 43535 | c.391A>C | p.N131H | - | 17:7578539-7578539 |
| 454 | TP53 | NM_000546 | 43537 | c.565G>A | p.A189T | - | 17:7578284-7578284 |
| 455 | TP53 | NM_000546 | 43541 | c.559+3G>C | p.? | - | 17:7578368-7578368 |
| 456 | TP53 | NM_000546 | 43544 | c.260C>A | p.P87Q | - | 17:7579427-7579427 |
| 457 | TP53 | NM_000546 | 43545 | c.503A>G | p.H168R | - | 17:7578427-7578427 |
| 458 | TP53 | NM_000546 | 43548 | c.467G>T | p.R156L | - | 17:7578463-7578463 |
| 459 | TP53 | NM_000546 | 43550 | c.694A>T | p.I232F | - | 17:7577587-7577587 |
| 460 | TP53 | NM_000546 | 43555 | c.736A>G | p.M246V | - | 17:7577545-7577545 |
| 461 | TP53 | NM_000546 | 43559 | c.517G>T | p.V173L | - | 17:7578413-7578413 |
| 462 | TP53 | NM_000546 | 43564 | c.708C>A | p.Y236* | - | 17:7577573-7577573 |
| 463 | TP53 | NM_000546 | 43565 | c.857A>G | p.E286G | - | 17:7577081-7577081 |
| 464 | TP53 | NM_000546 | 43570 | c.529_546del18 | p.P177_C182delPHHERC | - | 17:7578384-7578401 |
| 465 | TP53 | NM_000546 | 43582 | c.454C>T | p.P152S | - | 17:7578476-7578476 |
| 466 | TP53 | NM_000546 | 43584 | c.534_535CC>TT | p.H179Y | - | 17:7578395-7578396 |
| 467 | TP53 | NM_000546 | 43585 | c.843_844CC>TT | p.R282W | - | 17:7577094-7577095 |
| 468 | TP53 | NM_000546 | 43587 | c.832_833CC>TT | p.P278F | - | 17:7577105-7577106 |
| 469 | TP53 | NM_000546 | 43588 | c.740A>C | p.N247T | - | 17:7577541-7577541 |
| 470 | TP53 | NM_000546 | 43592 | c.395A>T | p.K132M | - | 17:7578535-7578535 |
| 471 | TP53 | NM_000546 | 43594 | c.605G>A | p.R202H | - | 17:7578244-7578244 |
| 472 | TP53 | NM_000546 | 43596 | c.841G>A | p.D281N | - | 17:7577097-7577097 |
| 473 | TP53 | NM_000546 | 43597 | c.538G>T | p.E180* | - | 17:7578392-7578392 |
| 474 | TP53 | NM_000546 | 43599 | c.607G>A | p.V203M | - | 17:7578242-7578242 |
| 475 | TP53 | NM_000546 | 43602 | c.706T>G | p.Y236D | - | 17:7577575-7577575 |
| 476 | TP53 | NM_000546 | 43606 | c.734G>A | p.G245D | - | 17:7577547-7577547 |
| 477 | TP53 | NM_000546 | 43608 | c.605G>C | p.R202P | - | 17:7578244-7578244 |
| 478 | TP53 | NM_000546 | 43614 | c.854A>C | p.E285A | - | 17:7577084-7577084 |
| 479 | TP53 | NM_000546 | 43615 | c.473G>C | p.R158P | - | 17:7578457-7578457 |
| 480 | TP53 | NM_000546 | 43616 | c.704A>G | p.N235S | - | 17:7577577-7577577 |
| 481 | TP53 | NM_000546 | 43621 | c.809T>G | p.F270C | - | 17:7577129-7577129 |
| 482 | TP53 | NM_000546 | 43623 | c.581T>A | p.L194H | - | 17:7578268-7578268 |
| 483 | TP53 | NM_000546 | 43624 | c.875A>G | p.K292R | - | 17:7577063-7577063 |
| 484 | TP53 | NM_000546 | 43625 | c.469G>A | p.V157I | - | 17:7578461-7578461 |
| 485 | TP53 | NM_000546 | 43626 | c.475G>A | p.A159T | - | 17:7578455-7578455 |
| 486 | TP53 | NM_000546 | 43629 | c.745A>T | p.R249W | - | 17:7577536-7577536 |
| 487 | TP53 | NM_000546 | 43632 | c.493C>T | p.Q165* | - | 17:7578437-7578437 |
| 488 | TP53 | NM_000546 | 43635 | c.536A>T | p.H179L | - | 17:7578394-7578394 |
| 489 | TP53 | NM_000546 | 43641 | c.628delA | p.N210fs*37 | - | 17:7578221-7578221 |
| 490 | TP53 | NM_000546 | 43642 | c.613T>C | p.Y205H | - | 17:7578236-7578236 |
| 491 | TP53 | NM_000546 | 43645 | c.721delT | p.S241fs*6 | - | 17:7577560-7577560 |
| 492 | TP53 | NM_000546 | 43648 | c.685_686delTG | p.C229fs*10 | - | 17:7577595-7577596 |
| 493 | TP53 | NM_000546 | 43650 | c.638G>T | p.R213L | - | 17:7578211-7578211 |
| 494 | TP53 | NM_000546 | 43651 | c.763A>T | p.I255F | - | 17:7577518-7577518 |
| 495 | TP53 | NM_000546 | 43652 | c.731G>T | p.G244V | - | 17:7577550-7577550 |
| 496 | TP53 | NM_000546 | 43656 | c.732C>G | p.G244G | - | 17:7577549-7577549 |
| 497 | TP53 | NM_000546 | 43657 | c.569C>T | p.P190L | - | 17:7578280-7578280 |
| 498 | TP53 | NM_000546 | 43660 | c.719G>T | p.S240I | - | 17:7577562-7577562 |
| 499 | TP53 | NM_000546 | 43661 | c.394delA | p.K132fs*38 | - | 17:7578536-7578536 |
| 500 | TP53 | NM_000546 | 43663 | c.826G>C | p.A276P | - | 17:7577112-7577112 |
| 501 | TP53 | NM_000546 | 43665 | c.746G>C | p.R249T | - | 17:7577535-7577535 |
| 502 | TP53 | NM_000546 | 43666 | c.462C>T | p.G154G | - | 17:7578468-7578468 |
| 503 | TP53 | NM_000546 | 43667 | c.820G>A | p.V274I | - | 17:7577118-7577118 |
| 504 | TP53 | NM_000546 | 43670 | c.465C>T | p.T155T | - | 17:7578465-7578465 |
| 505 | TP53 | NM_000546 | 43674 | c.835G>T | p.G279W | - | 17:7577103-7577103 |
| 506 | TP53 | NM_000546 | 43675 | c.457C>T | p.P153S | - | 17:7578473-7578473 |
| 507 | TP53 | NM_000546 | 43678 | c.305C>T | p.T102I | - | 17:7579382-7579382 |
| 508 | TP53 | NM_000546 | 43679 | c.531C>T | p.P177P | - | 17:7578399-7578399 |
| 509 | TP53 | NM_000546 | 43680 | c.523C>T | p.R175C | - | 17:7578407-7578407 |
| 510 | TP53 | NM_000546 | 43681 | c.647T>G | p.V216G | - | 17:7578202-7578202 |
| 511 | TP53 | NM_000546 | 43682 | c.328C>T | p.R110C | - | 17:7579359-7579359 |
| 512 | TP53 | NM_000546 | 43683 | c.758C>T | p.T253I | - | 17:7577523-7577523 |
| 513 | TP53 | NM_000546 | 43684 | c.720T>G | p.S240R | - | 17:7577561-7577561 |
| 514 | TP53 | NM_000546 | 43687 | c.641A>G | p.H214R | - | 17:7578208-7578208 |
| 515 | TP53 | NM_000546 | 43688 | c.265C>T | p.P89S | - | 17:7579422-7579422 |
| 516 | TP53 | NM_000546 | 43689 | c.482C>T | p.A161V | - | 17:7578448-7578448 |
| 517 | TP53 | NM_000546 | 43690 | c.592G>A | p.E198K | - | 17:7578257-7578257 |
| 518 | TP53 | NM_000546 | 43692 | c.460G>A | p.G154S | - | 17:7578470-7578470 |
| 519 | TP53 | NM_000546 | 43695 | c.748C>T | p.P250S | - | 17:7577533-7577533 |
| 520 | TP53 | NM_000546 | 43697 | c.832C>A | p.P278T | - | 17:7577106-7577106 |
| 521 | TP53 | NM_000546 | 43698 | c.566C>G | p.A189G | - | 17:7578283-7578283 |
| 522 | TP53 | NM_000546 | 43700 | c.712T>A | p.C238S | - | 17:7577569-7577569 |
| 523 | TP53 | NM_000546 | 43702 | c.571C>T | p.P191S | - | 17:7578278-7578278 |
| 524 | TP53 | NM_000546 | 43704 | c.405C>T | p.C135C | - | 17:7578525-7578525 |
| 525 | TP53 | NM_000546 | 43706 | c.811G>C | p.E271Q | - | 17:7577127-7577127 |
| 526 | TP53 | NM_000546 | 43709 | c.500A>G | p.Q167R | - | 17:7578430-7578430 |
| 527 | TP53 | NM_000546 | 43710 | c.468delC | p.V157fs*13 | - | 17:7578462-7578462 |
| 528 | TP53 | NM_000546 | 43714 | c.836G>A | p.G279E | - | 17:7577102-7577102 |
| 529 | TP53 | NM_000546 | 43723 | c.398T>C | p.M133T | - | 17:7578532-7578532 |
| 530 | TP53 | NM_000546 | 43725 | c.862A>T | p.N288Y | - | 17:7577076-7577076 |
| 531 | TP53 | NM_000546 | 43726 | c.727A>T | p.M243L | - | 17:7577554-7577554 |
| 532 | TP53 | NM_000546 | 43728 | c.543C>T | p.R181R | - | 17:7578387-7578387 |
| 533 | TP53 | NM_000546 | 43730 | c.398T>G | p.M133R | - | 17:7578532-7578532 |
| 534 | TP53 | NM_000546 | 43732 | c.517delG | p.V173fs*1 | - | 17:7578413-7578413 |
| 535 | TP53 | NM_000546 | 43734 | c.528C>A | p.C176* | - | 17:7578402-7578402 |
| 536 | TP53 | NM_000546 | 43737 | c.830G>A | p.C277Y | - | 17:7577108-7577108 |
| 537 | TP53 | NM_000546 | 43739 | c.467G>A | p.R156H | - | 17:7578463-7578463 |
| 538 | TP53 | NM_000546 | 43743 | c.914A>G | p.K305R | - | 17:7577024-7577024 |
| 539 | TP53 | NM_000546 | 43744 | c.466C>A | p.R156S | - | 17:7578464-7578464 |
| 540 | TP53 | NM_000546 | 43746 | c.881A>G | p.E294G | - | 17:7577057-7577057 |
| 541 | TP53 | NM_000546 | 43747 | c.872A>G | p.K291R | - | 17:7577066-7577066 |
| 542 | TP53 | NM_000546 | 43749 | c.595G>A | p.G199R | - | 17:7578254-7578254 |
| 543 | TP53 | NM_000546 | 43750 | c.811G>T | p.E271* | - | 17:7577127-7577127 |
| 544 | TP53 | NM_000546 | 43753 | c.560-1G>A | p.? | - | 17:7578290-7578290 |
| 545 | TP53 | NM_000546 | 43755 | c.833C>A | p.P278H | - | 17:7577105-7577105 |
| 546 | TP53 | NM_000546 | 43761 | c.612G>A | p.E204E | - | 17:7578237-7578237 |
| 547 | TP53 | NM_000546 | 43765 | c.727A>C | p.M243L | - | 17:7577554-7577554 |
| 548 | TP53 | NM_000546 | 43766 | c.899C>T | p.P300L | - | 17:7577039-7577039 |
| 549 | TP53 | NM_000546 | 43767 | c.406C>G | p.Q136E | - | 17:7578524-7578524 |
| 550 | TP53 | NM_000546 | 43768 | c.700T>G | p.Y234D | - | 17:7577581-7577581 |
| 551 | TP53 | NM_000546 | 43772 | c.538G>A | p.E180K | - | 17:7578392-7578392 |
| 552 | TP53 | NM_000546 | 43773 | c.913A>T | p.K305* | - | 17:7577025-7577025 |
| 553 | TP53 | NM_000546 | 43776 | c.861G>A | p.E287E | - | 17:7577077-7577077 |
| 554 | TP53 | NM_000546 | 43777 | c.603G>C | p.L201F | - | 17:7578246-7578246 |
| 555 | TP53 | NM_000546 | 43778 | c.713G>T | p.C238F | - | 17:7577568-7577568 |
| 556 | TP53 | NM_000546 | 43779 | c.589G>A | p.V197M | - | 17:7578260-7578260 |
| 557 | TP53 | NM_000546 | 43781 | c.472delC | p.R158fs*12 | - | 17:7578458-7578458 |
| 558 | TP53 | NM_000546 | 43782 | c.576G>A | p.Q192Q | - | 17:7578273-7578273 |
| 559 | TP53 | NM_000546 | 43787 | c.217G>A | p.V73M | - | 17:7579470-7579470 |
| 560 | TP53 | NM_000546 | 43793 | c.617T>A | p.L206* | - | 17:7578232-7578232 |
| 561 | TP53 | NM_000546 | 43795 | c.1023delC | p.R342fs*3 | - | 17:7574004-7574004 |
| 562 | TP53 | NM_000546 | 43797 | c.550G>C | p.D184H | - | 17:7578380-7578380 |
| 563 | TP53 | NM_000546 | 43801 | c.716A>C | p.N239T | - | 17:7577565-7577565 |
| 564 | TP53 | NM_000546 | 43806 | c.689C>A | p.T230N | - | 17:7577592-7577592 |
| 565 | TP53 | NM_000546 | 43807 | c.637delC | p.R213fs*34 | - | 17:7578212-7578212 |
| 566 | TP53 | NM_000546 | 43809 | c.808T>A | p.F270I | - | 17:7577130-7577130 |
| 567 | TP53 | NM_000546 | 43814 | c.587G>C | p.R196P | - | 17:7578262-7578262 |
| 568 | TP53 | NM_000546 | 43820 | c.489C>G | p.Y163* | - | 17:7578441-7578441 |
| 569 | TP53 | NM_000546 | 43823 | c.825T>G | p.C275W | - | 17:7577113-7577113 |
| 570 | TP53 | NM_000546 | 43826 | c.706T>A | p.Y236N | - | 17:7577575-7577575 |
| 571 | TP53 | NM_000546 | 43827 | c.581T>C | p.L194P | - | 17:7578268-7578268 |
| 572 | TP53 | NM_000546 | 43828 | c.544T>A | p.C182S | - | 17:7578386-7578386 |
| 573 | TP53 | NM_000546 | 43829 | c.752T>G | p.I251S | - | 17:7577529-7577529 |
| 574 | TP53 | NM_000546 | 43831 | c.472_475delCGCG | p.R158fs*11 | - | 17:7578455-7578458 |
| 575 | TP53 | NM_000546 | 43833 | c.578A>C | p.H193P | - | 17:7578271-7578271 |
| 576 | TP53 | NM_000546 | 43836 | c.475G>C | p.A159P | - | 17:7578455-7578455 |
| 577 | TP53 | NM_000546 | 43837 | c.843C>G | p.D281E | - | 17:7577095-7577095 |
| 578 | TP53 | NM_000546 | 43841 | c.560-1G>T | p.? | - | 17:7578290-7578290 |
| 579 | TP53 | NM_000546 | 43843 | c.817C>G | p.R273G | - | 17:7577121-7577121 |
| 580 | TP53 | NM_000546 | 43844 | c.613T>G | p.Y205D | - | 17:7578236-7578236 |
| 581 | TP53 | NM_000546 | 43846 | c.487T>C | p.Y163H | - | 17:7578443-7578443 |
| 582 | TP53 | NM_000546 | 43848 | c.472C>T | p.R158C | - | 17:7578458-7578458 |
| 583 | TP53 | NM_000546 | 43850 | c.659A>C | p.Y220S | - | 17:7578190-7578190 |
| 584 | TP53 | NM_000546 | 43851 | c.506T>C | p.M169T | - | 17:7578424-7578424 |
| 585 | TP53 | NM_000546 | 43853 | c.684C>G | p.D228E | - | 17:7577597-7577597 |
| 586 | TP53 | NM_000546 | 43859 | c.598delA | p.N200fs*47 | - | 17:7578251-7578251 |
| 587 | TP53 | NM_000546 | 43860 | c.704A>T | p.N235I | - | 17:7577577-7577577 |
| 588 | TP53 | NM_000546 | 43861 | c.502C>T | p.H168Y | - | 17:7578428-7578428 |
| 589 | TP53 | NM_000546 | 43862 | c.683A>C | p.D228A | - | 17:7577598-7577598 |
| 590 | TP53 | NM_000546 | 43864 | c.739A>T | p.N247Y | - | 17:7577542-7577542 |
| 591 | TP53 | NM_000546 | 43865 | c.701A>C | p.Y234S | - | 17:7577580-7577580 |
| 592 | TP53 | NM_000546 | 43868 | c.689C>T | p.T230I | - | 17:7577592-7577592 |
| 593 | TP53 | NM_000546 | 43871 | c.746G>T | p.R249M | - | 17:7577535-7577535 |
| 594 | TP53 | NM_000546 | 43872 | c.560-1G>C | p.? | - | 17:7578290-7578290 |
| 595 | TP53 | NM_000546 | 43879 | c.812A>G | p.E271G | - | 17:7577126-7577126 |
| 596 | TP53 | NM_000546 | 43881 | c.757A>T | p.T253S | - | 17:7577524-7577524 |
| 597 | TP53 | NM_000546 | 43882 | c.1010G>A | p.R337H | - | 17:7574017-7574017 |
| 598 | TP53 | NM_000546 | 43889 | c.691A>T | p.T231S | - | 17:7577590-7577590 |
| 599 | TP53 | NM_000546 | 43891 | c.480G>A | p.M160I | - | 17:7578450-7578450 |
| 600 | TP53 | NM_000546 | 43896 | c.818G>C | p.R273P | - | 17:7577120-7577120 |
| 601 | TP53 | NM_000546 | 43898 | c.485T>G | p.I162S | - | 17:7578445-7578445 |
| 602 | TP53 | NM_000546 | 43900 | c.376T>G | p.Y126D | - | 17:7578554-7578554 |
| 603 | TP53 | NM_000546 | 43902 | c.823T>C | p.C275R | - | 17:7577115-7577115 |
| 604 | TP53 | NM_000546 | 43903 | c.470T>G | p.V157G | - | 17:7578460-7578460 |
| 605 | TP53 | NM_000546 | 43905 | c.590T>G | p.V197G | - | 17:7578259-7578259 |
| 606 | TP53 | NM_000546 | 43906 | c.843C>A | p.D281E | - | 17:7577095-7577095 |
| 607 | TP53 | NM_000546 | 43909 | c.817C>A | p.R273S | - | 17:7577121-7577121 |
| 608 | TP53 | NM_000546 | 43910 | c.245C>T | p.P82L | - | 17:7579442-7579442 |
| 609 | TP53 | NM_000546 | 43912 | c.395A>C | p.K132T | - | 17:7578535-7578535 |
| 610 | TP53 | NM_000546 | 43915 | c.886C>T | p.H296Y | - | 17:7577052-7577052 |
| 611 | TP53 | NM_000546 | 43918 | c.809T>A | p.F270Y | - | 17:7577129-7577129 |
| 612 | TP53 | NM_000546 | 43919 | c.856G>T | p.E286* | - | 17:7577082-7577082 |
| 613 | TP53 | NM_000546 | 43920 | c.680C>T | p.S227F | - | 17:7577601-7577601 |
| 614 | TP53 | NM_000546 | 43923 | c.800G>A | p.R267Q | - | 17:7577138-7577138 |
| 615 | TP53 | NM_000546 | 43927 | c.559+9C>T | p.? | - | 17:7578362-7578362 |
| 616 | TP53 | NM_000546 | 43928 | c.615T>A | p.Y205* | - | 17:7578234-7578234 |
| 617 | TP53 | NM_000546 | 43929 | c.582T>C | p.L194L | - | 17:7578267-7578267 |
| 618 | TP53 | NM_000546 | 43931 | c.523C>A | p.R175S | - | 17:7578407-7578407 |
| 619 | TP53 | NM_000546 | 43934 | c.471C>A | p.V157V | - | 17:7578459-7578459 |
| 620 | TP53 | NM_000546 | 43935 | c.577C>A | p.H193N | - | 17:7578272-7578272 |
| 621 | TP53 | NM_000546 | 43936 | c.857A>T | p.E286V | - | 17:7577081-7577081 |
| 622 | TP53 | NM_000546 | 43939 | c.632C>T | p.T211I | - | 17:7578217-7578217 |
| 623 | TP53 | NM_000546 | 43940 | c.474C>T | p.R158R | - | 17:7578456-7578456 |
| 624 | TP53 | NM_000546 | 43941 | c.400T>G | p.F134V | - | 17:7578530-7578530 |
| 625 | TP53 | NM_000546 | 43945 | c.821T>G | p.V274G | - | 17:7577117-7577117 |
| 626 | TP53 | NM_000546 | 43947 | c.614A>G | p.Y205C | - | 17:7578235-7578235 |
| 627 | TP53 | NM_000546 | 43949 | c.401T>G | p.F134C | - | 17:7578529-7578529 |
| 628 | TP53 | NM_000546 | 43951 | c.643A>G | p.S215G | - | 17:7578206-7578206 |
| 629 | TP53 | NM_000546 | 43952 | c.710T>A | p.M237K | - | 17:7577571-7577571 |
| 630 | TP53 | NM_000546 | 43955 | c.514G>A | p.V172I | - | 17:7578416-7578416 |
| 631 | TP53 | NM_000546 | 43956 | c.700T>A | p.Y234N | - | 17:7577581-7577581 |
| 632 | TP53 | NM_000546 | 43957 | c.750C>T | p.P250P | - | 17:7577531-7577531 |
| 633 | TP53 | NM_000546 | 43958 | c.843C>T | p.D281D | - | 17:7577095-7577095 |
| 634 | TP53 | NM_000546 | 43960 | c.683A>G | p.D228G | - | 17:7577598-7577598 |
| 635 | TP53 | NM_000546 | 43962 | c.805A>G | p.S269G | - | 17:7577133-7577133 |
| 636 | TP53 | NM_000546 | 43963 | c.396G>C | p.K132N | - | 17:7578534-7578534 |
| 637 | TP53 | NM_000546 | 43964 | c.459C>T | p.P153P | - | 17:7578471-7578471 |
| 638 | TP53 | NM_000546 | 43965 | c.734G>C | p.G245A | - | 17:7577547-7577547 |
| 639 | TP53 | NM_000546 | 43967 | c.751A>T | p.I251F | - | 17:7577530-7577530 |
| 640 | TP53 | NM_000546 | 43968 | c.866T>C | p.L289P | - | 17:7577072-7577072 |
| 641 | TP53 | NM_000546 | 43970 | c.380C>A | p.S127Y | - | 17:7578550-7578550 |
| 642 | TP53 | NM_000546 | 43973 | c.718A>G | p.S240G | - | 17:7577563-7577563 |
| 643 | TP53 | NM_000546 | 43977 | c.849C>T | p.R283R | - | 17:7577089-7577089 |
| 644 | TP53 | NM_000546 | 43978 | c.529delC | p.H178fs*69 | - | 17:7578401-7578401 |
| 645 | TP53 | NM_000546 | 43979 | c.802A>C | p.N268H | - | 17:7577136-7577136 |
| 646 | TP53 | NM_000546 | 43980 | c.691A>G | p.T231A | - | 17:7577590-7577590 |
| 647 | TP53 | NM_000546 | 43986 | c.908G>A | p.S303N | - | 17:7577030-7577030 |
| 648 | TP53 | NM_000546 | 43987 | c.622G>A | p.D208N | - | 17:7578227-7578227 |
| 649 | TP53 | NM_000546 | 43988 | c.905G>A | p.G302E | - | 17:7577033-7577033 |
| 650 | TP53 | NM_000546 | 43989 | c.596G>A | p.G199E | - | 17:7578253-7578253 |
| 651 | TP53 | NM_000546 | 43990 | c.610G>A | p.E204K | - | 17:7578239-7578239 |
| 652 | TP53 | NM_000546 | 43995 | c.740A>T | p.N247I | - | 17:7577541-7577541 |
| 653 | TP53 | NM_000546 | 44002 | c.577C>G | p.H193D | - | 17:7578272-7578272 |
| 654 | TP53 | NM_000546 | 44004 | c.569C>G | p.P190R | - | 17:7578280-7578280 |
| 655 | TP53 | NM_000546 | 44005 | c.835delG | p.R280fs*65 | - | 17:7577103-7577103 |
| 656 | TP53 | NM_000546 | 44009 | c.463_470delACCCGCGT | p.T155fs*23 | - | 17:7578460-7578467 |
| 657 | TP53 | NM_000546 | 44011 | c.610delG | p.E204fs*43 | - | 17:7578239-7578239 |
| 658 | TP53 | NM_000546 | 44017 | c.869G>A | p.R290H | - | 17:7577069-7577069 |
| 659 | TP53 | NM_000546 | 44018 | c.214C>T | p.R72C | - | 17:7579473-7579473 |
| 660 | TP53 | NM_000546 | 44019 | c.226_270del45 | p.A76_S90del15 | - | 17:7579417-7579461 |
| 661 | TP53 | NM_000546 | 44023 | c.560G>A | p.G187D | - | 17:7578289-7578289 |
| 662 | TP53 | NM_000546 | 44026 | c.559G>A | p.G187S | - | 17:7578371-7578371 |
| 663 | TP53 | NM_000546 | 44029 | c.550G>A | p.D184N | - | 17:7578380-7578380 |
| 664 | TP53 | NM_000546 | 44030 | c.760A>G | p.I254V | - | 17:7577521-7577521 |
| 665 | TP53 | NM_000546 | 44032 | c.298C>T | p.Q100* | - | 17:7579389-7579389 |
| 666 | TP53 | NM_000546 | 44033 | c.464C>T | p.T155I | - | 17:7578466-7578466 |
| 667 | TP53 | NM_000546 | 44035 | c.496T>C | p.S166P | - | 17:7578434-7578434 |
| 668 | TP53 | NM_000546 | 44036 | c.296C>T | p.S99F | - | 17:7579391-7579391 |
| 669 | TP53 | NM_000546 | 44037 | c.322G>A | p.G108S | - | 17:7579365-7579365 |
| 670 | TP53 | NM_000546 | 44048 | c.280T>A | p.S94T | - | 17:7579407-7579407 |
| 671 | TP53 | NM_000546 | 44054 | c.754C>T | p.L252F | - | 17:7577527-7577527 |
| 672 | TP53 | NM_000546 | 44057 | c.517G>C | p.V173L | - | 17:7578413-7578413 |
| 673 | TP53 | NM_000546 | 44058 | c.761T>C | p.I254T | - | 17:7577520-7577520 |
| 674 | TP53 | NM_000546 | 44061 | c.456G>A | p.P152P | - | 17:7578474-7578474 |
| 675 | TP53 | NM_000546 | 44063 | c.389T>G | p.L130R | - | 17:7578541-7578541 |
| 676 | TP53 | NM_000546 | 44064 | c.748delC | p.I251fs*94 | - | 17:7577533-7577533 |
| 677 | TP53 | NM_000546 | 44067 | c.721T>A | p.S241T | - | 17:7577560-7577560 |
| 678 | TP53 | NM_000546 | 44068 | c.532C>A | p.H178N | - | 17:7578398-7578398 |
| 679 | TP53 | NM_000546 | 44070 | c.1031T>C | p.L344P | - | 17:7573996-7573996 |
| 680 | TP53 | NM_000546 | 44072 | c.706_708delTAC | p.Y236del | - | 17:7577573-7577575 |
| 681 | TP53 | NM_000546 | 44074 | c.605_606GT>CG | p.R202P | - | 17:7578243-7578244 |
| 682 | TP53 | NM_000546 | 44075 | c.251C>G | p.A84G | - | 17:7579436-7579436 |
| 683 | TP53 | NM_000546 | 44076 | c.655C>T | p.P219S | - | 17:7578194-7578194 |
| 684 | TP53 | NM_000546 | 44091 | c.746G>A | p.R249K | - | 17:7577535-7577535 |
| 685 | TP53 | NM_000546 | 44092 | c.794T>G | p.L265R | - | 17:7577144-7577144 |
| 686 | TP53 | NM_000546 | 44093 | c.644G>A | p.S215N | - | 17:7578205-7578205 |
| 687 | TP53 | NM_000546 | 44094 | c.716A>G | p.N239S | - | 17:7577565-7577565 |
| 688 | TP53 | NM_000546 | 44096 | c.748C>G | p.P250A | - | 17:7577533-7577533 |
| 689 | TP53 | NM_000546 | 44097 | c.530C>T | p.P177L | - | 17:7578400-7578400 |
| 690 | TP53 | NM_000546 | 44102 | c.637C>G | p.R213G | - | 17:7578212-7578212 |
| 691 | TP53 | NM_000546 | 44103 | c.737T>A | p.M246K | - | 17:7577544-7577544 |
| 692 | TP53 | NM_000546 | 44112 | c.640C>T | p.H214Y | - | 17:7578209-7578209 |
| 693 | TP53 | NM_000546 | 44113 | c.693C>T | p.T231T | - | 17:7577588-7577588 |
| 694 | TP53 | NM_000546 | 44114 | c.826G>A | p.A276T | - | 17:7577112-7577112 |
| 695 | TP53 | NM_000546 | 44119 | c.483C>T | p.A161A | - | 17:7578447-7578447 |
| 696 | TP53 | NM_000546 | 44120 | c.532C>T | p.H178Y | - | 17:7578398-7578398 |
| 697 | TP53 | NM_000546 | 44124 | c.751delA | p.I251fs*94 | - | 17:7577530-7577530 |
| 698 | TP53 | NM_000546 | 44125 | c.486C>G | p.I162M | - | 17:7578444-7578444 |
| 699 | TP53 | NM_000546 | 44126 | c.507G>A | p.M169I | - | 17:7578423-7578423 |
| 700 | TP53 | NM_000546 | 44127 | c.880G>A | p.E294K | - | 17:7577058-7577058 |
| 701 | TP53 | NM_000546 | 44128 | c.879G>A | p.G293G | - | 17:7577059-7577059 |
| 702 | TP53 | NM_000546 | 44129 | c.729G>A | p.M243I | - | 17:7577552-7577552 |
| 703 | TP53 | NM_000546 | 44130 | c.477C>T | p.A159A | - | 17:7578453-7578453 |
| 704 | TP53 | NM_000546 | 44131 | c.879G>C | p.G293G | - | 17:7577059-7577059 |
| 705 | TP53 | NM_000546 | 44132 | c.708C>T | p.Y236Y | - | 17:7577573-7577573 |
| 706 | TP53 | NM_000546 | 44133 | c.859G>T | p.E287* | - | 17:7577079-7577079 |
| 707 | TP53 | NM_000546 | 44134 | c.528delC | p.H178fs*69 | - | 17:7578402-7578402 |
| 708 | TP53 | NM_000546 | 44135 | c.724T>G | p.C242G | - | 17:7577557-7577557 |
| 709 | TP53 | NM_000546 | 44140 | c.596G>T | p.G199V | - | 17:7578253-7578253 |
| 710 | TP53 | NM_000546 | 44142 | c.377A>C | p.Y126S | - | 17:7578553-7578553 |
| 711 | TP53 | NM_000546 | 44146 | c.526T>A | p.C176S | - | 17:7578404-7578404 |
| 712 | TP53 | NM_000546 | 44151 | c.535C>A | p.H179N | - | 17:7578395-7578395 |
| 713 | TP53 | NM_000546 | 44152 | c.542G>T | p.R181L | - | 17:7578388-7578388 |
| 714 | TP53 | NM_000546 | 44156 | c.810T>A | p.F270L | - | 17:7577128-7577128 |
| 715 | TP53 | NM_000546 | 44157 | c.601delT | p.L201fs*46 | - | 17:7578248-7578248 |
| 716 | TP53 | NM_000546 | 44162 | c.635_636delTT | p.F212fs*3 | - | 17:7578213-7578214 |
| 717 | TP53 | NM_000546 | 44165 | c.903A>G | p.P301P | - | 17:7577035-7577035 |
| 718 | TP53 | NM_000546 | 44167 | c.908G>C | p.S303T | - | 17:7577030-7577030 |
| 719 | TP53 | NM_000546 | 44169 | c.614A>C | p.Y205S | - | 17:7578235-7578235 |
| 720 | TP53 | NM_000546 | 44171 | c.840A>T | p.R280S | - | 17:7577098-7577098 |
| 721 | TP53 | NM_000546 | 44172 | c.572C>G | p.P191R | - | 17:7578277-7578277 |
| 722 | TP53 | NM_000546 | 44174 | c.604C>A | p.R202S | - | 17:7578245-7578245 |
| 723 | TP53 | NM_000546 | 44175 | c.644G>C | p.S215T | - | 17:7578205-7578205 |
| 724 | TP53 | NM_000546 | 44183 | c.715delA | p.N239fs*8 | - | 17:7577566-7577566 |
| 725 | TP53 | NM_000546 | 44185 | c.555C>A | p.S185R | - | 17:7578375-7578375 |
| 726 | TP53 | NM_000546 | 44192 | c.272G>A | p.W91* | - | 17:7579415-7579415 |
| 727 | TP53 | NM_000546 | 44194 | c.251C>T | p.A84V | - | 17:7579436-7579436 |
| 728 | TP53 | NM_000546 | 44198 | c.653T>G | p.V218G | - | 17:7578196-7578196 |
| 729 | TP53 | NM_000546 | 44200 | c.242C>T | p.T81I | - | 17:7579445-7579445 |
| 730 | TP53 | NM_000546 | 44202 | c.550G>T | p.D184Y | - | 17:7578380-7578380 |
| 731 | TP53 | NM_000546 | 44206 | c.399G>T | p.M133I | - | 17:7578531-7578531 |
| 732 | TP53 | NM_000546 | 44207 | c.874delA | p.E294fs*51 | - | 17:7577064-7577064 |
| 733 | TP53 | NM_000546 | 44212 | c.391_393delAAC | p.N131del | - | 17:7578537-7578539 |
| 734 | TP53 | NM_000546 | 44214 | c.537T>A | p.H179Q | - | 17:7578393-7578393 |
| 735 | TP53 | NM_000546 | 44215 | c.533A>C | p.H178P | - | 17:7578397-7578397 |
| 736 | TP53 | NM_000546 | 44216 | c.487T>G | p.Y163D | - | 17:7578443-7578443 |
| 737 | TP53 | NM_000546 | 44217 | c.718delA | p.S240fs*7 | - | 17:7577563-7577563 |
| 738 | TP53 | NM_000546 | 44218 | c.536A>C | p.H179P | - | 17:7578394-7578394 |
| 739 | TP53 | NM_000546 | 44219 | c.405C>G | p.C135W | - | 17:7578525-7578525 |
| 740 | TP53 | NM_000546 | 44221 | c.730G>C | p.G244R | - | 17:7577551-7577551 |
| 741 | TP53 | NM_000546 | 44224 | c.721T>G | p.S241A | - | 17:7577560-7577560 |
| 742 | TP53 | NM_000546 | 44225 | c.859G>A | p.E287K | - | 17:7577079-7577079 |
| 743 | TP53 | NM_000546 | 44226 | c.380C>T | p.S127F | - | 17:7578550-7578550 |
| 744 | TP53 | NM_000546 | 44227 | c.854A>T | p.E285V | - | 17:7577084-7577084 |
| 745 | TP53 | NM_000546 | 44229 | c.515T>A | p.V172D | - | 17:7578415-7578415 |
| 746 | TP53 | NM_000546 | 44230 | c.481delG | p.A161fs*9 | - | 17:7578449-7578449 |
| 747 | TP53 | NM_000546 | 44231 | c.262delG | p.A88fs*35 | - | 17:7579425-7579425 |
| 748 | TP53 | NM_000546 | 44233 | c.840A>C | p.R280S | - | 17:7577098-7577098 |
| 749 | TP53 | NM_000546 | 44234 | c.571_573delCCT | p.P191del | - | 17:7578276-7578278 |
| 750 | TP53 | NM_000546 | 44236 | c.806G>A | p.S269N | - | 17:7577132-7577132 |
| 751 | TP53 | NM_000546 | 44237 | c.904delG | p.S303fs*42 | - | 17:7577034-7577034 |
| 752 | TP53 | NM_000546 | 44238 | c.631A>G | p.T211A | - | 17:7578218-7578218 |
| 753 | TP53 | NM_000546 | 44239 | c.647delT | p.V216fs*31 | - | 17:7578202-7578202 |
| 754 | TP53 | NM_000546 | 44240 | c.514G>T | p.V172F | - | 17:7578416-7578416 |
| 755 | TP53 | NM_000546 | 44241 | c.592G>T | p.E198* | - | 17:7578257-7578257 |
| 756 | TP53 | NM_000546 | 44247 | c.754_756delCTC | p.L252del | - | 17:7577525-7577527 |
| 757 | TP53 | NM_000546 | 44249 | c.623A>T | p.D208V | - | 17:7578226-7578226 |
| 758 | TP53 | NM_000546 | 44250 | c.856G>C | p.E286Q | - | 17:7577082-7577082 |
| 759 | TP53 | NM_000546 | 44257 | c.301delA | p.T102fs*21 | - | 17:7579386-7579386 |
| 760 | TP53 | NM_000546 | 44262 | c.808T>C | p.F270L | - | 17:7577130-7577130 |
| 761 | TP53 | NM_000546 | 44267 | c.472_477delCGCGCC | p.R158_A159delRA | - | 17:7578453-7578458 |
| 762 | TP53 | NM_000546 | 44268 | c.559+1G>T | p.? | - | 17:7578370-7578370 |
| 763 | TP53 | NM_000546 | 44271 | c.688A>C | p.T230P | - | 17:7577593-7577593 |
| 764 | TP53 | NM_000546 | 44274 | c.647T>A | p.V216E | - | 17:7578202-7578202 |
| 765 | TP53 | NM_000546 | 44275 | c.499_500delCA | p.Q167fs*13 | - | 17:7578430-7578431 |
| 766 | TP53 | NM_000546 | 44282 | c.496T>G | p.S166A | - | 17:7578434-7578434 |
| 767 | TP53 | NM_000546 | 44287 | c.229C>G | p.P77A | - | 17:7579458-7579458 |
| 768 | TP53 | NM_000546 | 44289 | c.497C>T | p.S166L | - | 17:7578433-7578433 |
| 769 | TP53 | NM_000546 | 44290 | c.763A>G | p.I255V | - | 17:7577518-7577518 |
| 770 | TP53 | NM_000546 | 44292 | c.858A>G | p.E286E | - | 17:7577080-7577080 |
| 771 | TP53 | NM_000546 | 44294 | c.815T>C | p.V272A | - | 17:7577123-7577123 |
| 772 | TP53 | NM_000546 | 44297 | c.376-3C>T | p.? | - | 17:7578557-7578557 |
| 773 | TP53 | NM_000546 | 44298 | c.462C>A | p.G154G | - | 17:7578468-7578468 |
| 774 | TP53 | NM_000546 | 44299 | c.501G>A | p.Q167Q | - | 17:7578429-7578429 |
| 775 | TP53 | NM_000546 | 44300 | c.548C>T | p.S183L | - | 17:7578382-7578382 |
| 776 | TP53 | NM_000546 | 44301 | c.468C>G | p.R156R | - | 17:7578462-7578462 |
| 777 | TP53 | NM_000546 | 44303 | c.463A>G | p.T155A | - | 17:7578467-7578467 |
| 778 | TP53 | NM_000546 | 44305 | c.479T>A | p.M160K | - | 17:7578451-7578451 |
| 779 | TP53 | NM_000546 | 44306 | c.845G>C | p.R282P | - | 17:7577093-7577093 |
| 780 | TP53 | NM_000546 | 44308 | c.494A>G | p.Q165R | - | 17:7578436-7578436 |
| 781 | TP53 | NM_000546 | 44310 | c.738G>A | p.M246I | - | 17:7577543-7577543 |
| 782 | TP53 | NM_000546 | 44312 | c.511G>A | p.E171K | - | 17:7578419-7578419 |
| 783 | TP53 | NM_000546 | 44313 | c.686G>A | p.C229Y | - | 17:7577595-7577595 |
| 784 | TP53 | NM_000546 | 44317 | c.653T>A | p.V218E | - | 17:7578196-7578196 |
| 785 | TP53 | NM_000546 | 44319 | c.405C>A | p.C135* | - | 17:7578525-7578525 |
| 786 | TP53 | NM_000546 | 44320 | c.484A>T | p.I162F | - | 17:7578446-7578446 |
| 787 | TP53 | NM_000546 | 44321 | c.712T>C | p.C238R | - | 17:7577569-7577569 |
| 788 | TP53 | NM_000546 | 44322 | c.728T>A | p.M243K | - | 17:7577553-7577553 |
| 789 | TP53 | NM_000546 | 44326 | c.706T>C | p.Y236H | - | 17:7577575-7577575 |
| 790 | TP53 | NM_000546 | 44327 | c.518T>C | p.V173A | - | 17:7578412-7578412 |
| 791 | TP53 | NM_000546 | 44328 | c.478A>G | p.M160V | - | 17:7578452-7578452 |
| 792 | TP53 | NM_000546 | 44329 | c.470T>A | p.V157D | - | 17:7578460-7578460 |
| 793 | TP53 | NM_000546 | 44334 | c.649G>T | p.V217L | - | 17:7578200-7578200 |
| 794 | TP53 | NM_000546 | 44336 | c.499delC | p.Q167fs*3 | - | 17:7578431-7578431 |
| 795 | TP53 | NM_000546 | 44338 | c.845G>A | p.R282Q | - | 17:7577093-7577093 |
| 796 | TP53 | NM_000546 | 44343 | c.547T>C | p.S183P | - | 17:7578383-7578383 |
| 797 | TP53 | NM_000546 | 44345 | c.915G>T | p.K305N | - | 17:7577023-7577023 |
| 798 | TP53 | NM_000546 | 44346 | c.875A>C | p.K292T | - | 17:7577063-7577063 |
| 799 | TP53 | NM_000546 | 44349 | c.566C>T | p.A189V | - | 17:7578283-7578283 |
| 800 | TP53 | NM_000546 | 44350 | c.699C>G | p.H233Q | - | 17:7577582-7577582 |
| 801 | TP53 | NM_000546 | 44351 | c.572C>T | p.P191L | - | 17:7578277-7578277 |
| 802 | TP53 | NM_000546 | 44352 | c.850A>C | p.T284P | - | 17:7577088-7577088 |
| 803 | TP53 | NM_000546 | 44358 | c.634delT | p.R213fs*34 | - | 17:7578215-7578215 |
| 804 | TP53 | NM_000546 | 44360 | c.686_687delGT | p.C229fs*10 | - | 17:7577594-7577595 |
| 805 | TP53 | NM_000546 | 44365 | c.607G>T | p.V203L | - | 17:7578242-7578242 |
| 806 | TP53 | NM_000546 | 44367 | c.458C>T | p.P153L | - | 17:7578472-7578472 |
| 807 | TP53 | NM_000546 | 44371 | c.631delA | p.T211fs*36 | - | 17:7578218-7578218 |
| 808 | TP53 | NM_000546 | 44372 | c.640delC | p.H214fs*33 | - | 17:7578209-7578209 |
| 809 | TP53 | NM_000546 | 44375 | c.650T>G | p.V217G | - | 17:7578199-7578199 |
| 810 | TP53 | NM_000546 | 44378 | c.726C>A | p.C242* | - | 17:7577555-7577555 |
| 811 | TP53 | NM_000546 | 44380 | c.376T>A | p.Y126N | - | 17:7578554-7578554 |
| 812 | TP53 | NM_000546 | 44383 | c.518T>G | p.V173G | - | 17:7578412-7578412 |
| 813 | TP53 | NM_000546 | 44384 | c.510G>A | p.T170T | - | 17:7578420-7578420 |
| 814 | TP53 | NM_000546 | 44387 | c.491A>C | p.K164T | - | 17:7578439-7578439 |
| 815 | TP53 | NM_000546 | 44388 | c.853G>T | p.E285* | - | 17:7577085-7577085 |
| 816 | TP53 | NM_000546 | 44390 | c.838A>T | p.R280* | - | 17:7577100-7577100 |
| 817 | TP53 | NM_000546 | 44391 | c.489C>T | p.Y163Y | - | 17:7578441-7578441 |
| 818 | TP53 | NM_000546 | 44393 | c.821T>C | p.V274A | - | 17:7577117-7577117 |
| 819 | TP53 | NM_000546 | 44396 | c.382delC | p.P128fs*42 | - | 17:7578548-7578548 |
| 820 | TP53 | NM_000546 | 44397 | c.382C>T | p.P128S | - | 17:7578548-7578548 |
| 821 | TP53 | NM_000546 | 44398 | c.682G>A | p.D228N | - | 17:7577599-7577599 |
| 822 | TP53 | NM_000546 | 44399 | c.677G>T | p.G226V | - | 17:7577604-7577604 |
| 823 | TP53 | NM_000546 | 44405 | c.376_396del21 | p.Y126_K132delYSPALNK | - | 17:7578534-7578554 |
| 824 | TP53 | NM_000546 | 44407 | c.642T>G | p.H214Q | - | 17:7578207-7578207 |
| 825 | TP53 | NM_000546 | 44411 | c.608T>A | p.V203E | - | 17:7578241-7578241 |
| 826 | TP53 | NM_000546 | 44412 | c.882G>A | p.E294E | - | 17:7577056-7577056 |
| 827 | TP53 | NM_000546 | 44413 | c.484A>G | p.I162V | - | 17:7578446-7578446 |
| 828 | TP53 | NM_000546 | 44415 | c.711G>C | p.M237I | - | 17:7577570-7577570 |
| 829 | TP53 | NM_000546 | 44416 | c.459C>A | p.P153P | - | 17:7578471-7578471 |
| 830 | TP53 | NM_000546 | 44417 | c.910delA | p.T304fs*41 | - | 17:7577028-7577028 |
| 831 | TP53 | NM_000546 | 44424 | c.590T>A | p.V197E | - | 17:7578259-7578259 |
| 832 | TP53 | NM_000546 | 44426 | c.568C>G | p.P190A | - | 17:7578281-7578281 |
| 833 | TP53 | NM_000546 | 44428 | c.741C>T | p.N247N | - | 17:7577540-7577540 |
| 834 | TP53 | NM_000546 | 44431 | c.505A>G | p.M169V | - | 17:7578425-7578425 |
| 835 | TP53 | NM_000546 | 44433 | c.872A>C | p.K291T | - | 17:7577066-7577066 |
| 836 | TP53 | NM_000546 | 44438 | c.568C>A | p.P190T | - | 17:7578281-7578281 |
| 837 | TP53 | NM_000546 | 44439 | c.656C>T | p.P219L | - | 17:7578193-7578193 |
| 838 | TP53 | NM_000546 | 44441 | c.813G>C | p.E271D | - | 17:7577125-7577125 |
| 839 | TP53 | NM_000546 | 44443 | c.820G>C | p.V274L | - | 17:7577118-7577118 |
| 840 | TP53 | NM_000546 | 44446 | c.873G>C | p.K291N | - | 17:7577065-7577065 |
| 841 | TP53 | NM_000546 | 44447 | c.287C>T | p.S96F | - | 17:7579400-7579400 |
| 842 | TP53 | NM_000546 | 44448 | c.821T>A | p.V274D | - | 17:7577117-7577117 |
| 843 | TP53 | NM_000546 | 44451 | c.874A>G | p.K292E | - | 17:7577064-7577064 |
| 844 | TP53 | NM_000546 | 44453 | c.309C>G | p.Y103* | - | 17:7579378-7579378 |
| 845 | TP53 | NM_000546 | 44457 | c.751_759delATCCTCACC | p.I251_T253delILT | - | 17:7577522-7577530 |
| 846 | TP53 | NM_000546 | 44458 | c.688_698del11 | p.T230fs*6 | - | 17:7577583-7577593 |
| 847 | TP53 | NM_000546 | 44460 | c.757_760delACCA | p.T253fs*91 | - | 17:7577521-7577524 |
| 848 | TP53 | NM_000546 | 44463 | c.848G>T | p.R283L | - | 17:7577090-7577090 |
| 849 | TP53 | NM_000546 | 44464 | c.749_750CC>AG | p.P250Q | - | 17:7577531-7577532 |
| 850 | TP53 | NM_000546 | 44467 | c.497C>G | p.S166* | - | 17:7578433-7578433 |
| 851 | TP53 | NM_000546 | 44469 | c.812A>T | p.E271V | - | 17:7577126-7577126 |
| 852 | TP53 | NM_000546 | 44470 | c.845G>T | p.R282L | - | 17:7577093-7577093 |
| 853 | TP53 | NM_000546 | 44474 | c.392A>G | p.N131S | - | 17:7578538-7578538 |
| 854 | TP53 | NM_000546 | 44475 | c.871A>T | p.K291* | - | 17:7577067-7577067 |
| 855 | TP53 | NM_000546 | 44476 | c.749C>A | p.P250H | - | 17:7577532-7577532 |
| 856 | TP53 | NM_000546 | 44481 | c.313G>T | p.G105C | - | 17:7579374-7579374 |
| 857 | TP53 | NM_000546 | 44492 | c.273G>A | p.W91* | - | 17:7579414-7579414 |
| 858 | TP53 | NM_000546 | 44495 | c.559+2T>A | p.? | - | 17:7578369-7578369 |
| 859 | TP53 | NM_000546 | 44502 | c.599A>G | p.N200S | - | 17:7578250-7578250 |
| 860 | TP53 | NM_000546 | 44505 | c.660T>G | p.Y220* | - | 17:7578189-7578189 |
| 861 | TP53 | NM_000546 | 44506 | c.401T>C | p.F134S | - | 17:7578529-7578529 |
| 862 | TP53 | NM_000546 | 44510 | c.717C>G | p.N239K | - | 17:7577564-7577564 |
| 863 | TP53 | NM_000546 | 44511 | c.753C>A | p.I251I | - | 17:7577528-7577528 |
| 864 | TP53 | NM_000546 | 44512 | c.740A>G | p.N247S | - | 17:7577541-7577541 |
| 865 | TP53 | NM_000546 | 44513 | c.732C>A | p.G244G | - | 17:7577549-7577549 |
| 866 | TP53 | NM_000546 | 44514 | c.728T>G | p.M243R | - | 17:7577553-7577553 |
| 867 | TP53 | NM_000546 | 44517 | c.519G>A | p.V173V | - | 17:7578411-7578411 |
| 868 | TP53 | NM_000546 | 44518 | c.522G>A | p.R174R | - | 17:7578408-7578408 |
| 869 | TP53 | NM_000546 | 44521 | c.490A>C | p.K164Q | - | 17:7578440-7578440 |
| 870 | TP53 | NM_000546 | 44522 | c.887A>T | p.H296L | - | 17:7577051-7577051 |
| 871 | TP53 | NM_000546 | 44523 | c.863A>G | p.N288S | - | 17:7577075-7577075 |
| 872 | TP53 | NM_000546 | 44524 | c.521G>A | p.R174K | - | 17:7578409-7578409 |
| 873 | TP53 | NM_000546 | 44525 | c.709A>G | p.M237V | - | 17:7577572-7577572 |
| 874 | TP53 | NM_000546 | 44526 | c.471C>T | p.V157V | - | 17:7578459-7578459 |
| 875 | TP53 | NM_000546 | 44535 | c.761T>A | p.I254N | - | 17:7577520-7577520 |
| 876 | TP53 | NM_000546 | 44536 | c.728T>C | p.M243T | - | 17:7577553-7577553 |
| 877 | TP53 | NM_000546 | 44537 | c.595G>T | p.G199* | - | 17:7578254-7578254 |
| 878 | TP53 | NM_000546 | 44539 | c.584T>G | p.I195S | - | 17:7578265-7578265 |
| 879 | TP53 | NM_000546 | 44541 | c.754delC | p.L252fs*93 | - | 17:7577527-7577527 |
| 880 | TP53 | NM_000546 | 44544 | c.766A>G | p.T256A | - | 17:7577515-7577515 |
| 881 | TP53 | NM_000546 | 44546 | c.545G>A | p.C182Y | - | 17:7578385-7578385 |
| 882 | TP53 | NM_000546 | 44547 | c.677G>A | p.G226D | - | 17:7577604-7577604 |
| 883 | TP53 | NM_000546 | 44550 | c.386C>T | p.A129V | - | 17:7578544-7578544 |
| 884 | TP53 | NM_000546 | 44552 | c.509C>T | p.T170M | - | 17:7578421-7578421 |
| 885 | TP53 | NM_000546 | 44561 | c.454C>A | p.P152T | - | 17:7578476-7578476 |
| 886 | TP53 | NM_000546 | 44563 | c.544T>C | p.C182R | - | 17:7578386-7578386 |
| 887 | TP53 | NM_000546 | 44565 | c.907A>T | p.S303C | - | 17:7577031-7577031 |
| 888 | TP53 | NM_000546 | 44566 | c.525C>G | p.R175R | - | 17:7578405-7578405 |
| 889 | TP53 | NM_000546 | 44567 | c.647T>C | p.V216A | - | 17:7578202-7578202 |
| 890 | TP53 | NM_000546 | 44568 | c.840A>G | p.R280R | - | 17:7577098-7577098 |
| 891 | TP53 | NM_000546 | 44569 | c.588A>G | p.R196R | - | 17:7578261-7578261 |
| 892 | TP53 | NM_000546 | 44571 | c.581T>G | p.L194R | - | 17:7578268-7578268 |
| 893 | TP53 | NM_000546 | 44578 | c.721T>C | p.S241P | - | 17:7577560-7577560 |
| 894 | TP53 | NM_000546 | 44580 | c.815T>A | p.V272E | - | 17:7577123-7577123 |
| 895 | TP53 | NM_000546 | 44585 | c.655delC | p.Y220fs*27 | - | 17:7578194-7578194 |
| 896 | TP53 | NM_000546 | 44589 | c.393_395delCAA | p.N131del | - | 17:7578535-7578537 |
| 897 | TP53 | NM_000546 | 44599 | c.587G>A | p.R196Q | - | 17:7578262-7578262 |
| 898 | TP53 | NM_000546 | 44601 | c.695T>C | p.I232T | - | 17:7577586-7577586 |
| 899 | TP53 | NM_000546 | 44603 | c.835G>A | p.G279R | - | 17:7577103-7577103 |
| 900 | TP53 | NM_000546 | 44607 | c.646_648delGTG | p.V216del | - | 17:7578201-7578203 |
| 901 | TP53 | NM_000546 | 44609 | c.748_749CC>TT | p.P250F | - | 17:7577532-7577533 |
| 902 | TP53 | NM_000546 | 44613 | c.455C>A | p.P152Q | - | 17:7578475-7578475 |
| 903 | TP53 | NM_000546 | 44615 | c.586C>A | p.R196R | - | 17:7578263-7578263 |
| 904 | TP53 | NM_000546 | 44621 | c.718A>T | p.S240C | - | 17:7577563-7577563 |
| 905 | TP53 | NM_000546 | 44622 | c.694A>G | p.I232V | - | 17:7577587-7577587 |
| 906 | TP53 | NM_000546 | 44623 | c.487T>A | p.Y163N | - | 17:7578443-7578443 |
| 907 | TP53 | NM_000546 | 44625 | c.747G>A | p.R249R | - | 17:7577534-7577534 |
| 908 | TP53 | NM_000546 | 44633 | c.583A>T | p.I195F | - | 17:7578266-7578266 |
| 909 | TP53 | NM_000546 | 44637 | c.658T>C | p.Y220H | - | 17:7578191-7578191 |
| 910 | TP53 | NM_000546 | 44638 | c.640_647delCATAGTGT | p.H214fs*5 | - | 17:7578202-7578209 |
| 911 | TP53 | NM_000546 | 44639 | c.869G>T | p.R290L | - | 17:7577069-7577069 |
| 912 | TP53 | NM_000546 | 44641 | c.394A>T | p.K132* | - | 17:7578536-7578536 |
| 913 | TP53 | NM_000546 | 44642 | c.733delG | p.G245fs*2 | - | 17:7577548-7577548 |
| 914 | TP53 | NM_000546 | 44643 | c.404G>C | p.C135S | - | 17:7578526-7578526 |
| 915 | TP53 | NM_000546 | 44645 | c.527G>C | p.C176S | - | 17:7578403-7578403 |
| 916 | TP53 | NM_000546 | 44647 | c.717C>A | p.N239K | - | 17:7577564-7577564 |
| 917 | TP53 | NM_000546 | 44650 | c.751_753delATC | p.I251del | - | 17:7577528-7577530 |
| 918 | TP53 | NM_000546 | 44651 | c.856_863delGAAGAGAA | p.E286fs*17 | - | 17:7577075-7577082 |
| 919 | TP53 | NM_000546 | 44653 | c.713G>C | p.C238S | - | 17:7577568-7577568 |
| 920 | TP53 | NM_000546 | 44654 | c.400T>C | p.F134L | - | 17:7578530-7578530 |
| 921 | TP53 | NM_000546 | 44657 | c.722delC | p.C242fs*5 | - | 17:7577559-7577559 |
| 922 | TP53 | NM_000546 | 44659 | c.532delC | p.H178fs*69 | - | 17:7578398-7578398 |
| 923 | TP53 | NM_000546 | 44661 | c.632C>A | p.T211N | - | 17:7578217-7578217 |
| 924 | TP53 | NM_000546 | 44662 | c.766A>T | p.T256S | - | 17:7577515-7577515 |
| 925 | TP53 | NM_000546 | 44664 | c.736_750del15 | p.M246_P250delMNRRP | - | 17:7577531-7577545 |
| 926 | TP53 | NM_000546 | 44665 | c.568_570delCCT | p.P190del | - | 17:7578279-7578281 |
| 927 | TP53 | NM_000546 | 44670 | c.400delT | p.C135fs*35 | - | 17:7578530-7578530 |
| 928 | TP53 | NM_000546 | 44672 | c.658T>A | p.Y220N | - | 17:7578191-7578191 |
| 929 | TP53 | NM_000546 | 44673 | c.284C>T | p.S95F | - | 17:7579403-7579403 |
| 930 | TP53 | NM_000546 | 44676 | c.714T>G | p.C238W | - | 17:7577567-7577567 |
| 931 | TP53 | NM_000546 | 44681 | c.293C>T | p.P98L | - | 17:7579394-7579394 |
| 932 | TP53 | NM_000546 | 44682 | c.568C>T | p.P190S | - | 17:7578281-7578281 |
| 933 | TP53 | NM_000546 | 44683 | c.652G>A | p.V218M | - | 17:7578197-7578197 |
| 934 | TP53 | NM_000546 | 44687 | c.379T>C | p.S127P | - | 17:7578551-7578551 |
| 935 | TP53 | NM_000546 | 44689 | c.657C>T | p.P219P | - | 17:7578192-7578192 |
| 936 | TP53 | NM_000546 | 44692 | c.526T>G | p.C176G | - | 17:7578404-7578404 |
| 937 | TP53 | NM_000546 | 44693 | c.707A>C | p.Y236S | - | 17:7577574-7577574 |
| 938 | TP53 | NM_000546 | 44694 | c.486C>A | p.I162I | - | 17:7578444-7578444 |
| 939 | TP53 | NM_000546 | 44695 | c.634_635delTT | p.F212fs*3 | - | 17:7578214-7578215 |
| 940 | TP53 | NM_000546 | 44700 | c.556G>A | p.D186N | - | 17:7578374-7578374 |
| 941 | TP53 | NM_000546 | 44701 | c.817delC | p.R273fs*72 | - | 17:7577121-7577121 |
| 942 | TP53 | NM_000546 | 44705 | c.697C>T | p.H233Y | - | 17:7577584-7577584 |
| 943 | TP53 | NM_000546 | 44707 | c.609G>A | p.V203V | - | 17:7578240-7578240 |
| 944 | TP53 | NM_000546 | 44709 | c.855G>A | p.E285E | - | 17:7577083-7577083 |
| 945 | TP53 | NM_000546 | 44714 | c.553A>G | p.S185G | - | 17:7578377-7578377 |
| 946 | TP53 | NM_000546 | 44715 | c.460G>T | p.G154C | - | 17:7578470-7578470 |
| 947 | TP53 | NM_000546 | 44724 | c.846G>A | p.R282R | - | 17:7577092-7577092 |
| 948 | TP53 | NM_000546 | 44725 | c.522delG | p.R174fs*73 | - | 17:7578408-7578408 |
| 949 | TP53 | NM_000546 | 44726 | c.460_466delGGCACCC | p.G154fs*14 | - | 17:7578464-7578470 |
| 950 | TP53 | NM_000546 | 44729 | c.898C>T | p.P300S | - | 17:7577040-7577040 |
| 951 | TP53 | NM_000546 | 44730 | c.529_545del17 | p.P177fs*3 | - | 17:7578385-7578401 |
| 952 | TP53 | NM_000546 | 44732 | c.512A>G | p.E171G | - | 17:7578418-7578418 |
| 953 | TP53 | NM_000546 | 44735 | c.825T>C | p.C275C | - | 17:7577113-7577113 |
| 954 | TP53 | NM_000546 | 44737 | c.860A>G | p.E287G | - | 17:7577078-7577078 |
| 955 | TP53 | NM_000546 | 44742 | c.523_540del18 | p.R175_E180delRCPHHE | - | 17:7578390-7578407 |
| 956 | TP53 | NM_000546 | 44749 | c.453C>T | p.P151P | - | 17:7578477-7578477 |
| 957 | TP53 | NM_000546 | 44750 | c.883C>T | p.P295S | - | 17:7577055-7577055 |
| 958 | TP53 | NM_000546 | 44753 | c.901C>T | p.P301S | - | 17:7577037-7577037 |
| 959 | TP53 | NM_000546 | 44757 | c.586delC | p.R196fs*51 | - | 17:7578263-7578263 |
| 960 | TP53 | NM_000546 | 44759 | c.526delT | p.C176fs*71 | - | 17:7578404-7578404 |
| 961 | TP53 | NM_000546 | 44769 | c.755T>C | p.L252P | - | 17:7577526-7577526 |
| 962 | TP53 | NM_000546 | 44774 | c.376_393del18 | p.Y126_N131delYSPALN | - | 17:7578537-7578554 |
| 963 | TP53 | NM_000546 | 44776 | c.535C>G | p.H179D | - | 17:7578395-7578395 |
| 964 | TP53 | NM_000546 | 44782 | c.520A>T | p.R174W | - | 17:7578410-7578410 |
| 965 | TP53 | NM_000546 | 44784 | c.703_705delAAC | p.N235del | - | 17:7577576-7577578 |
| 966 | TP53 | NM_000546 | 44787 | c.732C>T | p.G244G | - | 17:7577549-7577549 |
| 967 | TP53 | NM_000546 | 44788 | c.454C>G | p.P152A | - | 17:7578476-7578476 |
| 968 | TP53 | NM_000546 | 44793 | c.537T>C | p.H179H | - | 17:7578393-7578393 |
| 969 | TP53 | NM_000546 | 44794 | c.392A>T | p.N131I | - | 17:7578538-7578538 |
| 970 | TP53 | NM_000546 | 44801 | c.503A>T | p.H168L | - | 17:7578427-7578427 |
| 971 | TP53 | NM_000546 | 44808 | c.503A>C | p.H168P | - | 17:7578427-7578427 |
| 972 | TP53 | NM_000546 | 44817 | c.661G>T | p.E221* | - | 17:7578188-7578188 |
| 973 | TP53 | NM_000546 | 44818 | c.531C>G | p.P177P | - | 17:7578399-7578399 |
| 974 | TP53 | NM_000546 | 44820 | c.692C>T | p.T231I | - | 17:7577589-7577589 |
| 975 | TP53 | NM_000546 | 44829 | c.403T>G | p.C135G | - | 17:7578527-7578527 |
| 976 | TP53 | NM_000546 | 44830 | c.1066G>T | p.G356W | - | 17:7573961-7573961 |
| 977 | TP53 | NM_000546 | 44832 | c.1096T>G | p.S366A | - | 17:7573931-7573931 |
| 978 | TP53 | NM_000546 | 44835 | c.852A>T | p.T284T | - | 17:7577086-7577086 |
| 979 | TP53 | NM_000546 | 44837 | c.556G>C | p.D186H | - | 17:7578374-7578374 |
| 980 | TP53 | NM_000546 | 44838 | c.720T>C | p.S240S | - | 17:7577561-7577561 |
| 981 | TP53 | NM_000546 | 44841 | c.491A>T | p.K164M | - | 17:7578439-7578439 |
| 982 | TP53 | NM_000546 | 44842 | c.478A>C | p.M160L | - | 17:7578452-7578452 |
| 983 | TP53 | NM_000546 | 44843 | c.759C>T | p.T253T | - | 17:7577522-7577522 |
| 984 | TP53 | NM_000546 | 44844 | c.727A>G | p.M243V | - | 17:7577554-7577554 |
| 985 | TP53 | NM_000546 | 44845 | c.591G>A | p.V197V | - | 17:7578258-7578258 |
| 986 | TP53 | NM_000546 | 44846 | c.636T>A | p.F212L | - | 17:7578213-7578213 |
| 987 | TP53 | NM_000546 | 44848 | c.579T>C | p.H193H | - | 17:7578270-7578270 |
| 988 | TP53 | NM_000546 | 44849 | c.575A>G | p.Q192R | - | 17:7578274-7578274 |
| 989 | TP53 | NM_000546 | 44850 | c.494A>T | p.Q165L | - | 17:7578436-7578436 |
| 990 | TP53 | NM_000546 | 44851 | c.494A>C | p.Q165P | - | 17:7578436-7578436 |
| 991 | TP53 | NM_000546 | 44852 | c.617delT | p.L206fs*41 | - | 17:7578232-7578232 |
| 992 | TP53 | NM_000546 | 44853 | c.661G>A | p.E221K | - | 17:7578188-7578188 |
| 993 | TP53 | NM_000546 | 44854 | c.655C>A | p.P219T | - | 17:7578194-7578194 |
| 994 | TP53 | NM_000546 | 44861 | c.490delA | p.K164fs*6 | - | 17:7578440-7578440 |
| 995 | TP53 | NM_000546 | 44868 | c.804C>T | p.N268N | - | 17:7577134-7577134 |
| 996 | TP53 | NM_000546 | 44870 | c.815T>G | p.V272G | - | 17:7577123-7577123 |
| 997 | TP53 | NM_000546 | 44871 | c.833delC | p.P278fs*67 | - | 17:7577105-7577105 |
| 998 | TP53 | NM_000546 | 44877 | c.584T>A | p.I195N | - | 17:7578265-7578265 |
| 999 | TP53 | NM_000546 | 44879 | c.1033delA | p.N345fs*25 | - | 17:7573994-7573994 |
| 1000 | TP53 | NM_000546 | 44886 | c.807C>T | p.S269S | - | 17:7577131-7577131 |
| 1001 | TP53 | NM_000546 | 44887 | c.644delG | p.S215fs*32 | - | 17:7578205-7578205 |
| 1002 | TP53 | NM_000546 | 44891 | c.796G>T | p.G266* | - | 17:7577142-7577142 |
| 1003 | TP53 | NM_000546 | 44896 | c.835_838delGGGA | p.G279fs*65 | - | 17:7577100-7577103 |
| 1004 | TP53 | NM_000546 | 44897 | c.871_889del19 | p.K291fs*48 | - | 17:7577049-7577067 |
| 1005 | TP53 | NM_000546 | 44900 | c.735C>T | p.G245G | - | 17:7577546-7577546 |
| 1006 | TP53 | NM_000546 | 44901 | c.532C>G | p.H178D | - | 17:7578398-7578398 |
| 1007 | TP53 | NM_000546 | 44903 | c.736delA | p.M246fs*1 | - | 17:7577545-7577545 |
| 1008 | TP53 | NM_000546 | 44908 | c.743_744GG>AA | p.R248Q | - | 17:7577537-7577538 |
| 1009 | TP53 | NM_000546 | 44910 | c.403T>A | p.C135S | - | 17:7578527-7578527 |
| 1010 | TP53 | NM_000546 | 44916 | c.746delG | p.R249fs*96 | - | 17:7577535-7577535 |
| 1011 | TP53 | NM_000546 | 44918 | c.844C>A | p.R282R | - | 17:7577094-7577094 |
| 1012 | TP53 | NM_000546 | 44920 | c.742C>A | p.R248R | - | 17:7577539-7577539 |
| 1013 | TP53 | NM_000546 | 44921 | c.748_756delCCCATCCTC | p.P250_L252delPIL | - | 17:7577525-7577533 |
| 1014 | TP53 | NM_000546 | 44923 | c.565G>C | p.A189P | - | 17:7578284-7578284 |
| 1015 | TP53 | NM_000546 | 44924 | c.615T>G | p.Y205* | - | 17:7578234-7578234 |
| 1016 | TP53 | NM_000546 | 44925 | c.605G>T | p.R202L | - | 17:7578244-7578244 |
| 1017 | TP53 | NM_000546 | 44929 | c.650T>A | p.V217E | - | 17:7578199-7578199 |
| 1018 | TP53 | NM_000546 | 44930 | c.653T>C | p.V218A | - | 17:7578196-7578196 |
| 1019 | TP53 | NM_000546 | 44933 | c.376-4A>G | p.? | - | 17:7578558-7578558 |
| 1020 | TP53 | NM_000546 | 44935 | c.724T>A | p.C242S | - | 17:7577557-7577557 |
| 1021 | TP53 | NM_000546 | 44940 | c.730delG | p.G244fs*3 | - | 17:7577551-7577551 |
| 1022 | TP53 | NM_000546 | 44948 | c.526T>C | p.C176R | - | 17:7578404-7578404 |
| 1023 | TP53 | NM_000546 | 44953 | c.700_702delTAC | p.Y234del | - | 17:7577579-7577581 |
| 1024 | TP53 | NM_000546 | 44956 | c.808T>G | p.F270V | - | 17:7577130-7577130 |
| 1025 | TP53 | NM_000546 | 44960 | c.708C>G | p.Y236* | - | 17:7577573-7577573 |
| 1026 | TP53 | NM_000546 | 44964 | c.719G>C | p.S240T | - | 17:7577562-7577562 |
| 1027 | TP53 | NM_000546 | 44965 | c.709A>T | p.M237L | - | 17:7577572-7577572 |
| 1028 | TP53 | NM_000546 | 44966 | c.385G>A | p.A129T | - | 17:7578545-7578545 |
| 1029 | TP53 | NM_000546 | 44971 | c.534C>T | p.H178H | - | 17:7578396-7578396 |
| 1030 | TP53 | NM_000546 | 44972 | c.831T>A | p.C277* | - | 17:7577107-7577107 |
| 1031 | TP53 | NM_000546 | 44973 | c.516T>C | p.V172V | - | 17:7578414-7578414 |
| 1032 | TP53 | NM_000546 | 44979 | c.645T>G | p.S215R | - | 17:7578204-7578204 |
| 1033 | TP53 | NM_000546 | 44986 | c.302A>G | p.K101R | - | 17:7579385-7579385 |
| 1034 | TP53 | NM_000546 | 44996 | c.515T>C | p.V172A | - | 17:7578415-7578415 |
| 1035 | TP53 | NM_000546 | 45005 | c.739A>G | p.N247D | - | 17:7577542-7577542 |
| 1036 | TP53 | NM_000546 | 45015 | c.380_381CC>TT | p.S127F | - | 17:7578549-7578550 |
| 1037 | TP53 | NM_000546 | 45017 | c.722_723CC>TT | p.S241F | - | 17:7577558-7577559 |
| 1038 | TP53 | NM_000546 | 45019 | c.471_472CC>TT | p.R158C | - | 17:7578458-7578459 |
| 1039 | TP53 | NM_000546 | 45021 | c.585_586CC>TT | p.R196* | - | 17:7578263-7578264 |
| 1040 | TP53 | NM_000546 | 45025 | c.488A>C | p.Y163S | - | 17:7578442-7578442 |
| 1041 | TP53 | NM_000546 | 45026 | c.560-2A>T | p.? | - | 17:7578291-7578291 |
| 1042 | TP53 | NM_000546 | 45028 | c.684C>T | p.D228D | - | 17:7577597-7577597 |
| 1043 | TP53 | NM_000546 | 45029 | c.565_591del27 | p.A189_V197delAPPQHLIRV | - | 17:7578258-7578284 |
| 1044 | TP53 | NM_000546 | 45032 | c.710T>G | p.M237R | - | 17:7577571-7577571 |
| 1045 | TP53 | NM_000546 | 45034 | c.748_749CC>AA | p.P250N | - | 17:7577532-7577533 |
| 1046 | TP53 | NM_000546 | 45035 | c.761T>G | p.I254S | - | 17:7577520-7577520 |
| 1047 | TP53 | NM_000546 | 45040 | c.321C>A | p.Y107* | - | 17:7579366-7579366 |
| 1048 | TP53 | NM_000546 | 45044 | c.576G>T | p.Q192H | - | 17:7578273-7578273 |
| 1049 | TP53 | NM_000546 | 45045 | c.695T>G | p.I232S | - | 17:7577586-7577586 |
| 1050 | TP53 | NM_000546 | 45046 | c.542G>C | p.R181P | - | 17:7578388-7578388 |
| 1051 | TP53 | NM_000546 | 45047 | c.515T>G | p.V172G | - | 17:7578415-7578415 |
| 1052 | TP53 | NM_000546 | 45050 | c.871A>G | p.K291E | - | 17:7577067-7577067 |
| 1053 | TP53 | NM_000546 | 45051 | c.597A>G | p.G199G | - | 17:7578252-7578252 |
| 1054 | TP53 | NM_000546 | 45055 | c.715_720delAACAGT | p.N239_S240delNS | - | 17:7577561-7577566 |
| 1055 | TP53 | NM_000546 | 45057 | c.475delG | p.A159fs*11 | - | 17:7578455-7578455 |
| 1056 | TP53 | NM_000546 | 45069 | c.886delC | p.H296fs*49 | - | 17:7577052-7577052 |
| 1057 | TP53 | NM_000546 | 45074 | c.829T>G | p.C277G | - | 17:7577109-7577109 |
| 1058 | TP53 | NM_000546 | 45077 | c.390C>T | p.L130L | - | 17:7578540-7578540 |
| 1059 | TP53 | NM_000546 | 45084 | c.744G>A | p.R248R | - | 17:7577537-7577537 |
| 1060 | TP53 | NM_000546 | 45089 | c.407A>C | p.Q136P | - | 17:7578523-7578523 |
| 1061 | TP53 | NM_000546 | 45091 | c.755T>A | p.L252H | - | 17:7577526-7577526 |
| 1062 | TP53 | NM_000546 | 45101 | c.913_915AAG>TAA | p.K305* | - | 17:7577023-7577025 |
| 1063 | TP53 | NM_000546 | 45103 | c.492G>A | p.K164K | - | 17:7578438-7578438 |
| 1064 | TP53 | NM_000546 | 45109 | c.831T>C | p.C277C | - | 17:7577107-7577107 |
| 1065 | TP53 | NM_000546 | 45110 | c.650T>C | p.V217A | - | 17:7578199-7578199 |
| 1066 | TP53 | NM_000546 | 45111 | c.469_473delGTCCG | p.V157fs*22 | - | 17:7578457-7578461 |
| 1067 | TP53 | NM_000546 | 45114 | c.702C>A | p.Y234* | - | 17:7577579-7577579 |
| 1068 | TP53 | NM_000546 | 45115 | c.640C>G | p.H214D | - | 17:7578209-7578209 |
| 1069 | TP53 | NM_000546 | 45116 | c.742delC | p.R248fs*97 | - | 17:7577539-7577539 |
| 1070 | TP53 | NM_000546 | 45120 | c.469G>C | p.V157L | - | 17:7578461-7578461 |
| 1071 | TP53 | NM_000546 | 45122 | c.645T>A | p.S215R | - | 17:7578204-7578204 |
| 1072 | TP53 | NM_000546 | 45128 | c.911C>T | p.T304I | - | 17:7577027-7577027 |
| 1073 | TP53 | NM_000546 | 45131 | c.383C>T | p.P128L | - | 17:7578547-7578547 |
| 1074 | TP53 | NM_000546 | 45134 | c.715_726del12 | p.N239_C242delNSSC | - | 17:7577555-7577566 |
| 1075 | TP53 | NM_000546 | 45138 | c.853G>C | p.E285Q | - | 17:7577085-7577085 |
| 1076 | TP53 | NM_000546 | 45140 | c.572_574delCTC | p.P191del | - | 17:7578275-7578277 |
| 1077 | TP53 | NM_000546 | 45154 | c.466C>G | p.R156G | - | 17:7578464-7578464 |
| 1078 | TP53 | NM_000546 | 45157 | c.688delA | p.T230fs*17 | - | 17:7577593-7577593 |
| 1079 | TP53 | NM_000546 | 45162 | c.709delA | p.M237fs*10 | - | 17:7577572-7577572 |
| 1080 | TP53 | NM_000546 | 45168 | c.722_724delCCT | p.S241del | - | 17:7577557-7577559 |
| 1081 | TP53 | NM_000546 | 45169 | c.326T>C | p.F109S | - | 17:7579361-7579361 |
| 1082 | TP53 | NM_000546 | 45172 | c.703A>T | p.N235Y | - | 17:7577578-7577578 |
| 1083 | TP53 | NM_000546 | 45178 | c.832delC | p.P278fs*67 | - | 17:7577106-7577106 |
| 1084 | TP53 | NM_000546 | 45179 | c.313G>C | p.G105R | - | 17:7579374-7579374 |
| 1085 | TP53 | NM_000546 | 45184 | c.902delC | p.P301fs*44 | - | 17:7577036-7577036 |
| 1086 | TP53 | NM_000546 | 45187 | c.490_499del10 | p.K164fs*3 | - | 17:7578431-7578440 |
| 1087 | TP53 | NM_000546 | 45188 | c.847delC | p.R283fs*62 | - | 17:7577091-7577091 |
| 1088 | TP53 | NM_000546 | 45194 | c.487delT | p.Y163fs*7 | - | 17:7578443-7578443 |
| 1089 | TP53 | NM_000546 | 45198 | c.555C>T | p.S185S | - | 17:7578375-7578375 |
| 1090 | TP53 | NM_000546 | 45200 | c.233C>T | p.A78V | - | 17:7579454-7579454 |
| 1091 | TP53 | NM_000546 | 45233 | c.884C>T | p.P295L | - | 17:7577054-7577054 |
| 1092 | TP53 | NM_000546 | 45240 | c.560G>T | p.G187V | - | 17:7578289-7578289 |
| 1093 | TP53 | NM_000546 | 45248 | c.805A>T | p.S269C | - | 17:7577133-7577133 |
| 1094 | TP53 | NM_000546 | 45253 | c.611A>G | p.E204G | - | 17:7578238-7578238 |
| 1095 | TP53 | NM_000546 | 45257 | c.626G>C | p.R209T | - | 17:7578223-7578223 |
| 1096 | TP53 | NM_000546 | 45261 | c.720T>A | p.S240R | - | 17:7577561-7577561 |
| 1097 | TP53 | NM_000546 | 45268 | c.827C>A | p.A276D | - | 17:7577111-7577111 |
| 1098 | TP53 | NM_000546 | 45275 | c.559G>T | p.G187C | - | 17:7578371-7578371 |
| 1099 | TP53 | NM_000546 | 45276 | c.1025G>C | p.R342P | - | 17:7574002-7574002 |
| 1100 | TP53 | NM_000546 | 45277 | c.856delG | p.E286fs*59 | - | 17:7577082-7577082 |
| 1101 | TP53 | NM_000546 | 45278 | c.1025G>A | p.R342Q | - | 17:7574002-7574002 |
| 1102 | TP53 | NM_000546 | 45284 | c.813G>A | p.E271E | - | 17:7577125-7577125 |
| 1103 | TP53 | NM_000546 | 45286 | c.475G>T | p.A159S | - | 17:7578455-7578455 |
| 1104 | TP53 | NM_000546 | 45288 | c.217G>C | p.V73L | - | 17:7579470-7579470 |
| 1105 | TP53 | NM_000546 | 45293 | c.407A>G | p.Q136R | - | 17:7578523-7578523 |
| 1106 | TP53 | NM_000546 | 45297 | c.810T>G | p.F270L | - | 17:7577128-7577128 |
| 1107 | TP53 | NM_000546 | 45299 | c.831T>G | p.C277W | - | 17:7577107-7577107 |
| 1108 | TP53 | NM_000546 | 45306 | c.886C>A | p.H296N | - | 17:7577052-7577052 |
| 1109 | TP53 | NM_000546 | 45307 | c.309C>A | p.Y103* | - | 17:7579378-7579378 |
| 1110 | TP53 | NM_000546 | 45308 | c.607delG | p.V203fs*44 | - | 17:7578242-7578242 |
| 1111 | TP53 | NM_000546 | 45311 | c.898C>G | p.P300A | - | 17:7577040-7577040 |
| 1112 | TP53 | NM_000546 | 45314 | c.553delA | p.S185fs*62 | - | 17:7578377-7578377 |
| 1113 | TP53 | NM_000546 | 45320 | c.569delC | p.P190fs*57 | - | 17:7578280-7578280 |
| 1114 | TP53 | NM_000546 | 45322 | c.757A>G | p.T253A | - | 17:7577524-7577524 |
| 1115 | TP53 | NM_000546 | 45326 | c.530C>A | p.P177H | - | 17:7578400-7578400 |
| 1116 | TP53 | NM_000546 | 45329 | c.710T>C | p.M237T | - | 17:7577571-7577571 |
| 1117 | TP53 | NM_000546 | 45332 | c.885T>C | p.P295P | - | 17:7577053-7577053 |
| 1118 | TP53 | NM_000546 | 45338 | c.552T>C | p.D184D | - | 17:7578378-7578378 |
| 1119 | TP53 | NM_000546 | 45341 | c.571delC | p.P191fs*56 | - | 17:7578278-7578278 |
| 1120 | TP53 | NM_000546 | 45342 | c.500A>T | p.Q167L | - | 17:7578430-7578430 |
| 1121 | TP53 | NM_000546 | 45364 | c.376-1delG | p.? | - | 17:7578555-7578555 |
| 1122 | TP53 | NM_000546 | 45372 | c.540G>T | p.E180D | - | 17:7578390-7578390 |
| 1123 | TP53 | NM_000546 | 45393 | c.793C>A | p.L265M | - | 17:7577145-7577145 |
| 1124 | TP53 | NM_000546 | 45394 | c.687T>A | p.C229* | - | 17:7577594-7577594 |
| 1125 | TP53 | NM_000546 | 45399 | c.526_543del18 | p.C176_R181delCPHHER | - | 17:7578387-7578404 |
| 1126 | TP53 | NM_000546 | 45407 | c.751A>G | p.I251V | - | 17:7577530-7577530 |
| 1127 | TP53 | NM_000546 | 45410 | c.733_734GG>AA | p.G245N | - | 17:7577547-7577548 |
| 1128 | TP53 | NM_000546 | 45411 | c.489C>A | p.Y163* | - | 17:7578441-7578441 |
| 1129 | TP53 | NM_000546 | 45413 | c.824G>C | p.C275S | - | 17:7577114-7577114 |
| 1130 | TP53 | NM_000546 | 45416 | c.524G>C | p.R175P | - | 17:7578406-7578406 |
| 1131 | TP53 | NM_000546 | 45417 | c.877G>A | p.G293R | - | 17:7577061-7577061 |
| 1132 | TP53 | NM_000546 | 45438 | c.626delG | p.R209fs*38 | - | 17:7578223-7578223 |
| 1133 | TP53 | NM_000546 | 45440 | c.567C>T | p.A189A | - | 17:7578282-7578282 |
| 1134 | TP53 | NM_000546 | 45441 | c.629A>G | p.N210S | - | 17:7578220-7578220 |
| 1135 | TP53 | NM_000546 | 45446 | c.865C>T | p.L289F | - | 17:7577073-7577073 |
| 1136 | TP53 | NM_000546 | 45449 | c.592G>C | p.E198Q | - | 17:7578257-7578257 |
| 1137 | TP53 | NM_000546 | 45459 | c.862delA | p.N288fs*57 | - | 17:7577076-7577076 |
| 1138 | TP53 | NM_000546 | 45467 | c.826G>T | p.A276S | - | 17:7577112-7577112 |
| 1139 | TP53 | NM_000546 | 45479 | c.504C>T | p.H168H | - | 17:7578426-7578426 |
| 1140 | TP53 | NM_000546 | 45487 | c.898delC | p.P301fs*44 | - | 17:7577040-7577040 |
| 1141 | TP53 | NM_000546 | 45488 | c.797G>C | p.G266A | - | 17:7577141-7577141 |
| 1142 | TP53 | NM_000546 | 45489 | c.603G>T | p.L201F | - | 17:7578246-7578246 |
| 1143 | TP53 | NM_000546 | 45490 | c.507G>T | p.M169I | - | 17:7578423-7578423 |
| 1144 | TP53 | NM_000546 | 45491 | c.822T>G | p.V274V | - | 17:7577116-7577116 |
| 1145 | TP53 | NM_000546 | 45494 | c.888_889CC>TT | p.H297Y | - | 17:7577049-7577050 |
| 1146 | TP53 | NM_000546 | 45500 | c.281C>A | p.S94* | - | 17:7579406-7579406 |
| 1147 | TP53 | NM_000546 | 45505 | c.455C>G | p.P152R | - | 17:7578475-7578475 |
| 1148 | TP53 | NM_000546 | 45506 | c.460_461GG>AT | p.G154I | - | 17:7578469-7578470 |
| 1149 | TP53 | NM_000546 | 45507 | c.806G>C | p.S269T | - | 17:7577132-7577132 |
| 1150 | TP53 | NM_000546 | 45509 | c.321C>T | p.Y107Y | - | 17:7579366-7579366 |
| 1151 | TP53 | NM_000546 | 45511 | c.645T>C | p.S215S | - | 17:7578204-7578204 |
| 1152 | TP53 | NM_000546 | 45515 | c.525C>T | p.R175R | - | 17:7578405-7578405 |
| 1153 | TP53 | NM_000546 | 45516 | c.662A>G | p.E221G | - | 17:7578187-7578187 |
| 1154 | TP53 | NM_000546 | 45519 | c.620A>G | p.D207G | - | 17:7578229-7578229 |
| 1155 | TP53 | NM_000546 | 45529 | c.683A>T | p.D228V | - | 17:7577598-7577598 |
| 1156 | TP53 | NM_000546 | 45534 | c.882G>T | p.E294D | - | 17:7577056-7577056 |
| 1157 | TP53 | NM_000546 | 45536 | c.1061A>G | p.Q354R | - | 17:7573966-7573966 |
| 1158 | TP53 | NM_000546 | 45541 | c.510G>T | p.T170T | - | 17:7578420-7578420 |
| 1159 | TP53 | NM_000546 | 45543 | c.743_744GG>TT | p.R248L | - | 17:7577537-7577538 |
| 1160 | TP53 | NM_000546 | 45546 | c.901delC | p.P301fs*44 | - | 17:7577037-7577037 |
| 1161 | TP53 | NM_000546 | 45548 | c.721_723delTCC | p.S241del | - | 17:7577558-7577560 |
| 1162 | TP53 | NM_000546 | 45551 | c.469_471delGTC | p.V157del | - | 17:7578459-7578461 |
| 1163 | TP53 | NM_000546 | 45562 | c.546C>A | p.C182* | - | 17:7578384-7578384 |
| 1164 | TP53 | NM_000546 | 45583 | c.514_559del46 | p.V173fs*59 | - | 17:7578371-7578416 |
| 1165 | TP53 | NM_000546 | 45586 | c.397delA | p.M133fs*37 | - | 17:7578533-7578533 |
| 1166 | TP53 | NM_000546 | 45594 | c.453C>G | p.P151P | - | 17:7578477-7578477 |
| 1167 | TP53 | NM_000546 | 45607 | c.676G>A | p.G226S | - | 17:7577605-7577605 |
| 1168 | TP53 | NM_000546 | 45611 | c.876A>C | p.K292N | - | 17:7577062-7577062 |
| 1169 | TP53 | NM_000546 | 45612 | c.685T>C | p.C229R | - | 17:7577596-7577596 |
| 1170 | TP53 | NM_000546 | 45620 | c.704A>C | p.N235T | - | 17:7577577-7577577 |
| 1171 | TP53 | NM_000546 | 45622 | c.461G>A | p.G154D | - | 17:7578469-7578469 |
| 1172 | TP53 | NM_000546 | 45626 | c.501G>T | p.Q167H | - | 17:7578429-7578429 |
| 1173 | TP53 | NM_000546 | 45627 | c.486C>T | p.I162I | - | 17:7578444-7578444 |
| 1174 | TP53 | NM_000546 | 45631 | c.688A>T | p.T230S | - | 17:7577593-7577593 |
| 1175 | TP53 | NM_000546 | 45632 | c.741C>A | p.N247K | - | 17:7577540-7577540 |
| 1176 | TP53 | NM_000546 | 45639 | c.1024delC | p.R342fs*3 | - | 17:7574003-7574003 |
| 1177 | TP53 | NM_000546 | 45647 | c.760A>T | p.I254F | - | 17:7577521-7577521 |
| 1178 | TP53 | NM_000546 | 45649 | c.854A>G | p.E285G | - | 17:7577084-7577084 |
| 1179 | TP53 | NM_000546 | 45654 | c.685_699del15 | p.C229_H233delCTTIH | - | 17:7577582-7577596 |
| 1180 | TP53 | NM_000546 | 45660 | c.457C>A | p.P153T | - | 17:7578473-7578473 |
| 1181 | TP53 | NM_000546 | 45670 | c.877delG | p.E294fs*51 | - | 17:7577061-7577061 |
| 1182 | TP53 | NM_000546 | 45671 | c.521G>T | p.R174M | - | 17:7578409-7578409 |
| 1183 | TP53 | NM_000546 | 45672 | c.376-2A>G | p.? | - | 17:7578556-7578556 |
| 1184 | TP53 | NM_000546 | 45674 | c.480G>T | p.M160I | - | 17:7578450-7578450 |
| 1185 | TP53 | NM_000546 | 45677 | c.714T>A | p.C238* | - | 17:7577567-7577567 |
| 1186 | TP53 | NM_000546 | 45679 | c.868C>T | p.R290C | - | 17:7577070-7577070 |
| 1187 | TP53 | NM_000546 | 45685 | c.613T>A | p.Y205N | - | 17:7578236-7578236 |
| 1188 | TP53 | NM_000546 | 45688 | c.867C>T | p.L289L | - | 17:7577071-7577071 |
| 1189 | TP53 | NM_000546 | 45691 | c.726C>T | p.C242C | - | 17:7577555-7577555 |
| 1190 | TP53 | NM_000546 | 45695 | c.827C>G | p.A276G | - | 17:7577111-7577111 |
| 1191 | TP53 | NM_000546 | 45703 | c.634T>A | p.F212I | - | 17:7578215-7578215 |
| 1192 | TP53 | NM_000546 | 45706 | c.801G>T | p.R267R | - | 17:7577137-7577137 |
| 1193 | TP53 | NM_000546 | 45707 | c.624C>A | p.D208E | - | 17:7578225-7578225 |
| 1194 | TP53 | NM_000546 | 45711 | c.559+2T>G | p.? | - | 17:7578369-7578369 |
| 1195 | TP53 | NM_000546 | 45728 | c.850A>G | p.T284A | - | 17:7577088-7577088 |
| 1196 | TP53 | NM_000546 | 45729 | c.842A>T | p.D281V | - | 17:7577096-7577096 |
| 1197 | TP53 | NM_000546 | 45735 | c.744G>C | p.R248R | - | 17:7577537-7577537 |
| 1198 | TP53 | NM_000546 | 45739 | c.677G>C | p.G226A | - | 17:7577604-7577604 |
| 1199 | TP53 | NM_000546 | 45751 | c.511G>C | p.E171Q | - | 17:7578419-7578419 |
| 1200 | TP53 | NM_000546 | 45777 | c.633delT | p.R213fs*34 | - | 17:7578216-7578216 |
| 1201 | TP53 | NM_000546 | 45784 | c.691delA | p.T231fs*16 | - | 17:7577590-7577590 |
| 1202 | TP53 | NM_000546 | 45786 | c.682G>T | p.D228Y | - | 17:7577599-7577599 |
| 1203 | TP53 | NM_000546 | 45796 | c.623A>G | p.D208G | - | 17:7578226-7578226 |
| 1204 | TP53 | NM_000546 | 45801 | c.312delG | p.G105fs*18 | - | 17:7579375-7579375 |
| 1205 | TP53 | NM_000546 | 45803 | c.889C>T | p.H297Y | - | 17:7577049-7577049 |
| 1206 | TP53 | NM_000546 | 45809 | c.376-1G>C | p.? | - | 17:7578555-7578555 |
| 1207 | TP53 | NM_000546 | 45820 | c.893A>T | p.E298V | - | 17:7577045-7577045 |
| 1208 | TP53 | NM_000546 | 45823 | c.558T>C | p.D186D | - | 17:7578372-7578372 |
| 1209 | TP53 | NM_000546 | 45824 | c.880G>C | p.E294Q | - | 17:7577058-7577058 |
| 1210 | TP53 | NM_000546 | 45838 | c.556delG | p.D186fs*61 | - | 17:7578374-7578374 |
| 1211 | TP53 | NM_000546 | 45843 | c.838_843delAGAGAC | p.R280_D281delRD | - | 17:7577095-7577100 |
| 1212 | TP53 | NM_000546 | 45851 | c.624C>G | p.D208E | - | 17:7578225-7578225 |
| 1213 | TP53 | NM_000546 | 45862 | c.710delT | p.M237fs*10 | - | 17:7577571-7577571 |
| 1214 | TP53 | NM_000546 | 45868 | c.678C>T | p.G226G | - | 17:7577603-7577603 |
| 1215 | TP53 | NM_000546 | 45870 | c.715A>T | p.N239Y | - | 17:7577566-7577566 |
| 1216 | TP53 | NM_000546 | 45882 | c.391delA | p.N131fs*39 | - | 17:7578539-7578539 |
| 1217 | TP53 | NM_000546 | 45891 | c.847_866del20 | p.R283fs*16 | - | 17:7577072-7577091 |
| 1218 | TP53 | NM_000546 | 45896 | c.466delC | p.R156fs*14 | - | 17:7578464-7578464 |
| 1219 | TP53 | NM_000546 | 45898 | c.814G>C | p.V272L | - | 17:7577124-7577124 |
| 1220 | TP53 | NM_000546 | 45906 | c.514delG | p.V172fs*2 | - | 17:7578416-7578416 |
| 1221 | TP53 | NM_000546 | 45918 | c.253C>T | p.P85S | - | 17:7579434-7579434 |
| 1222 | TP53 | NM_000546 | 45944 | c.318C>G | p.S106R | - | 17:7579369-7579369 |
| 1223 | TP53 | NM_000546 | 45959 | c.698A>T | p.H233L | - | 17:7577583-7577583 |
| 1224 | TP53 | NM_000546 | 45980 | c.757A>C | p.T253P | - | 17:7577524-7577524 |
| 1225 | TP53 | NM_000546 | 45985 | c.215G>A | p.R72H | - | 17:7579472-7579472 |
| 1226 | TP53 | NM_000546 | 45992 | c.736A>T | p.M246L | - | 17:7577545-7577545 |
| 1227 | TP53 | NM_000546 | 45995 | c.626G>A | p.R209K | - | 17:7578223-7578223 |
| 1228 | TP53 | NM_000546 | 45998 | c.906G>C | p.G302G | - | 17:7577032-7577032 |
| 1229 | TP53 | NM_000546 | 46000 | c.643A>C | p.S215R | - | 17:7578206-7578206 |
| 1230 | TP53 | NM_000546 | 46001 | c.466_486del21 | p.R156_I162delRVRAMAI | - | 17:7578444-7578464 |
| 1231 | TP53 | NM_000546 | 46015 | c.1043T>A | p.L348* | - | 17:7573984-7573984 |
| 1232 | TP53 | NM_000546 | 46031 | c.697C>G | p.H233D | - | 17:7577584-7577584 |
| 1233 | TP53 | NM_000546 | 46032 | c.836G>T | p.G279V | - | 17:7577102-7577102 |
| 1234 | TP53 | NM_000546 | 46035 | c.847C>G | p.R283G | - | 17:7577091-7577091 |
| 1235 | TP53 | NM_000546 | 46049 | c.376-2A>C | p.? | - | 17:7578556-7578556 |
| 1236 | TP53 | NM_000546 | 46059 | c.560-3T>G | p.? | - | 17:7578292-7578292 |
| 1237 | TP53 | NM_000546 | 46074 | c.604C>T | p.R202C | - | 17:7578245-7578245 |
| 1238 | TP53 | NM_000546 | 46095 | c.511delG | p.E171fs*3 | - | 17:7578419-7578419 |
| 1239 | TP53 | NM_000546 | 46103 | c.319T>G | p.Y107D | - | 17:7579368-7579368 |
| 1240 | TP53 | NM_000546 | 46107 | c.599A>T | p.N200I | - | 17:7578250-7578250 |
| 1241 | TP53 | NM_000546 | 46114 | c.389T>A | p.L130H | - | 17:7578541-7578541 |
| 1242 | TP53 | NM_000546 | 46115 | c.329G>A | p.R110H | - | 17:7579358-7579358 |
| 1243 | TP53 | NM_000546 | 46124 | c.466C>T | p.R156C | - | 17:7578464-7578464 |
| 1244 | TP53 | NM_000546 | 46131 | c.380delC | p.P128fs*42 | - | 17:7578550-7578550 |
| 1245 | TP53 | NM_000546 | 46136 | c.738G>T | p.M246I | - | 17:7577543-7577543 |
| 1246 | TP53 | NM_000546 | 46163 | c.534C>G | p.H178Q | - | 17:7578396-7578396 |
| 1247 | TP53 | NM_000546 | 46207 | c.910A>G | p.T304A | - | 17:7577028-7577028 |
| 1248 | TP53 | NM_000546 | 46208 | c.859_872del14 | p.N288fs*13 | - | 17:7577066-7577079 |
| 1249 | TP53 | NM_000546 | 46211 | c.633T>C | p.T211T | - | 17:7578216-7578216 |
| 1250 | TP53 | NM_000546 | 46212 | c.589G>T | p.V197L | - | 17:7578260-7578260 |
| 1251 | TP53 | NM_000546 | 46214 | c.635T>C | p.F212S | - | 17:7578214-7578214 |
| 1252 | TP53 | NM_000546 | 46224 | c.873G>A | p.K291K | - | 17:7577065-7577065 |
| 1253 | TP53 | NM_000546 | 46228 | c.729G>C | p.M243I | - | 17:7577552-7577552 |
| 1254 | TP53 | NM_000546 | 46265 | c.224C>G | p.P75R | - | 17:7579463-7579463 |
| 1255 | TP53 | NM_000546 | 46284 | c.837G>A | p.G279G | - | 17:7577101-7577101 |
| 1256 | TP53 | NM_000546 | 46288 | c.546C>T | p.C182C | - | 17:7578384-7578384 |
| 1257 | TP53 | NM_000546 | 46336 | c.712T>G | p.C238G | - | 17:7577569-7577569 |
| 1258 | TP53 | NM_000546 | 46348 | c.1044G>T | p.L348F | - | 17:7573983-7573983 |
| 1259 | TP53 | NM_000546 | 46393 | c.520_536del17 | p.R174fs*1 | - | 17:7578394-7578410 |
| 1260 | TP53 | NM_000546 | 51646 | c.498_499insC | p.Q167fs*14 | - | 17:7578432-7578433 |
| 1261 | TP53 | NM_000546 | 53285 | c.379T>A | p.S127T | - | 17:7578551-7578551 |
| 1262 | TP53 | NM_000546 | 6482 | c.625_626delAG | p.R209fs*6 | - | 17:7578223-7578224 |
| 1263 | TP53 | NM_000546 | 6496 | c.652_654delGTG | p.V218del | - | 17:7578195-7578197 |
| 1264 | TP53 | NM_000546 | 6530 | c.723delC | p.C242fs*5 | - | 17:7577558-7577558 |
| 1265 | TP53 | NM_000546 | 6545 | c.741_742CC>TT | p.R248W | - | 17:7577539-7577540 |
| 1266 | TP53 | NM_000546 | 6546 | c.741_742CC>AT | p.N247_R248>KW | - | 17:7577539-7577540 |
| 1267 | TP53 | NM_000546 | 6549 | c.743G>T | p.R248L | - | 17:7577538-7577538 |
| 1268 | TP53 | NM_000546 | 6621 | c.880delG | p.E294fs*51 | - | 17:7577058-7577058 |
| 1269 | TP53 | NM_000546 | 6815 | c.461G>T | p.G154V | - | 17:7578469-7578469 |
| 1270 | TP53 | NM_000546 | 6900 | c.376-1G>A | p.? | - | 17:7578555-7578555 |
| 1271 | TP53 | NM_000546 | 6901 | c.559+1G>A | p.? | - | 17:7578370-7578370 |
| 1272 | TP53 | NM_000546 | 69195 | c.714_715insT | p.N239fs*1 | - | 17:7577566-7577567 |
| 1273 | TP53 | NM_000546 | 6932 | c.733G>A | p.G245S | - | 17:7577548-7577548 |
| 1274 | TP53 | NM_000546 | 85574 | c.291_295delCCCTT | p.S99fs*48 | - | 17:7579392-7579396 |
| 1275 | TP53 | NM_000546 | 87513 | c.902_903insC | p.G302fs*4 | - | 17:7577035-7577036 |
| 1276 | TP53 | NM_000546 | 96575 | c.625_634del10 | p.R209fs*35 | - | 17:7578215-7578224 |
| 1277 | TACC3 | NM_006342 |  | c.10>11 |  | + | 4:1739324-1741505 |
| 1278 | STK11 | NM_000455 | 12924 | c.842delC | p.P281fs*6 | + | 19:1221319-1221319 |
| 1279 | STK11 | NM_000455 | 12925 | c.109C>T | p.Q37* | + | 19:1207021-1207021 |
| 1280 | STK11 | NM_000455 | 18652 | c.996G>A | p.W332* | + | 19:1223059-1223059 |
| 1281 | STK11 | NM_000455 | 20857 | c.787_790delTTGT | p.F264fs*22 | + | 19:1221264-1221267 |
| 1282 | STK11 | NM_000455 | 20871 | c.837delC | p.P281fs*6 | + | 19:1221314-1221314 |
| 1283 | STK11 | NM_000455 | 20874 | c.180C>G | p.Y60* | + | 19:1207092-1207092 |
| 1284 | STK11 | NM_000455 | 20943 | c.508C>T | p.Q170* | + | 19:1220415-1220415 |
| 1285 | STK11 | NM_000455 | 20944 | c.580G>T | p.D194Y | + | 19:1220487-1220487 |
| 1286 | STK11 | NM_000455 | 20957 | c.581A>T | p.D194V | + | 19:1220488-1220488 |
| 1287 | STK11 | NM_000455 | 21212 | c.169delG | p.E57fs*7 | + | 19:1207077-1207077 |
| 1288 | STK11 | NM_000455 | 21354 | c.511G>A | p.G171S | + | 19:1220418-1220418 |
| 1289 | STK11 | NM_000455 | 21355 | c.842C>T | p.P281L | + | 19:1221319-1221319 |
| 1290 | STK11 | NM_000455 | 21359 | c.595G>A | p.E199K | + | 19:1220502-1220502 |
| 1291 | STK11 | NM_000455 | 21360 | c.1062C>G | p.F354L | + | 19:1223125-1223125 |
| 1292 | STK11 | NM_000455 | 21378 | c.96C>G | p.T32T | + | 19:1207008-1207008 |
| 1293 | STK11 | NM_000455 | 25229 | c.595G>T | p.E199* | + | 19:1220502-1220502 |
| 1294 | STK11 | NM_000455 | 25847 | c.580G>A | p.D194N | + | 19:1220487-1220487 |
| 1295 | STK11 | NM_000455 | 25851 | c.842_843insC | p.L282fs*3 | + | 19:1221319-1221320 |
| 1296 | STK11 | NM_000455 | 27316 | c.475C>T | p.Q159* | + | 19:1220382-1220382 |
| 1297 | STK11 | NM_000455 | 27322 | c.180delC | p.Y60fs*1 | + | 19:1207092-1207092 |
| 1298 | STK11 | NM_000455 | 28298 | c.841_842>T | p.P281fs*6 | + | 19:1221318-1221319 |
| 1299 | STK11 | NM_000455 | 29005 | c.816C>T | p.Y272Y | + | 19:1221293-1221293 |
| 1300 | STK11 | NM_000455 | 48786 | c.587G>T | p.G196V | + | 19:1220494-1220494 |
| 1301 | SRC | NM_005417 | 1369 | c.1591C>T | p.Q531* | + | 20:36031762-36031762 |
| 1302 | SMO | NM_005631.3 | 13145 | c.595C>T | p.R199W | + | 7:128845101-128845101 |
| 1303 | SMO | NM_005631.3 | 13146 | c.1604G>T | p.W535L | + | 7:128850341-128850341 |
| 1304 | SMO | NM_005631.3 | 13147 | c.970G>A | p.A324T | + | 7:128846040-128846040 |
| 1305 | SMO | NM_005631.3 | 13148 | c.1210G>A | p.V404M | + | 7:128846374-128846374 |
| 1306 | SMO | NM_005631.3 | 13150 | c.1918A>G | p.T640A | + | 7:128851593-128851593 |
| 1307 | SMARCB1 | NM_003073.2 | 1002 | c.118C>T | p.R40* | + | 22:24133967-24133967 |
| 1308 | SMARCB1 | NM_003073.2 | 1057 | c.1148delC | p.P383fs | + | 22:24176357-24176357 |
| 1309 | SMARCB1 | NM_003073.2 | 1060 | c.1144delG | p.A382fs*4 | + | 22:24176353-24176353 |
| 1310 | SMARCB1 | NM_003073.2 | 1090 | c.1119-41G>A | p.? | + | 22:24176287-24176287 |
| 1311 | SMARCB1 | NM_003073.2 | 24595 | c.157C>T | p.R53* | + | 22:24134006-24134006 |
| 1312 | SMARCB1 | NM_003073.2 | 29382 | c.1143delG | p.A382fs*5 | + | 22:24176352-24176352 |
| 1313 | SMARCB1 | NM_003073.2 | 29495 | c.1145delC | p.P383fs*4 | + | 22:24176354-24176354 |
| 1314 | SMARCB1 | NM_003073.2 | 989 | c.1130G>A | p.R377H | + | 22:24176339-24176339 |
| 1315 | SMARCB1 | NM_003073.2 | 991 | c.141C>A | p.Y47* | + | 22:24133990-24133990 |
| 1316 | SMARCB1 | NM_003073.2 | 992 | c.472C>T | p.R158* | + | 22:24143240-24143240 |
| 1317 | SMARCB1 | NM_003073.2 | 993 | c.601C>T | p.R201* | + | 22:24145582-24145582 |
| 1318 | SMAD4 | NM_005359.3 | 13115 | c.431C>G | p.S144* | + | 18:48575671-48575671 |
| 1319 | SMAD4 | NM_005359.3 | 14057 | c.733C>T | p.Q245* | + | 18:48584560-48584560 |
| 1320 | SMAD4 | NM_005359.3 | 14096 | c.1333C>T | p.R445* | + | 18:48603032-48603032 |
| 1321 | SMAD4 | NM_005359.3 | 14105 | c.1394_1395insT | p.A466fs*28 | + | 18:48603093-48603094 |
| 1322 | SMAD4 | NM_005359.3 | 14110 | c.989A>C | p.E330A | + | 18:48591826-48591826 |
| 1323 | SMAD4 | NM_005359.3 | 14111 | c.1028C>G | p.S343* | + | 18:48591865-48591865 |
| 1324 | SMAD4 | NM_005359.3 | 14113 | c.1490G>A | p.R497H | + | 18:48604668-48604668 |
| 1325 | SMAD4 | NM_005359.3 | 14115 | c.1569C>G | p.C523W | + | 18:48604747-48604747 |
| 1326 | SMAD4 | NM_005359.3 | 14118 | c.502G>T | p.G168* | + | 18:48581198-48581198 |
| 1327 | SMAD4 | NM_005359.3 | 14121 | c.1015_1029del15 | p.F339_S343del | + | 18:48591852-48591866 |
| 1328 | SMAD4 | NM_005359.3 | 14122 | c.1082G>A | p.R361H | + | 18:48591919-48591919 |
| 1329 | SMAD4 | NM_005359.3 | 14124 | c.1341_1365del25 | p.Q448fs*20 | + | 18:48603040-48603064 |
| 1330 | SMAD4 | NM_005359.3 | 14126 | c.1519A>C | p.K507Q | + | 18:48604697-48604697 |
| 1331 | SMAD4 | NM_005359.3 | 14129 | c.1543A>T | p.R515* | + | 18:48604721-48604721 |
| 1332 | SMAD4 | NM_005359.3 | 14134 | c.1576G>T | p.E526* | + | 18:48604754-48604754 |
| 1333 | SMAD4 | NM_005359.3 | 14135 | c.1051G>C | p.D351H | + | 18:48591888-48591888 |
| 1334 | SMAD4 | NM_005359.3 | 14140 | c.1081C>T | p.R361C | + | 18:48591918-48591918 |
| 1335 | SMAD4 | NM_005359.3 | 14163 | c.931C>T | p.Q311* | + | 18:48586262-48586262 |
| 1336 | SMAD4 | NM_005359.3 | 14167 | c.955+5G>C | p.? | + | 18:48586291-48586291 |
| 1337 | SMAD4 | NM_005359.3 | 14174 | c.1072G>T | p.G358* | + | 18:48591909-48591909 |
| 1338 | SMAD4 | NM_005359.3 | 14175 | c.1236C>G | p.Y412* | + | 18:48593485-48593485 |
| 1339 | SMAD4 | NM_005359.3 | 14177 | c.1546_1553delCAGAGCAT | p.S517fs*7 | + | 18:48604724-48604731 |
| 1340 | SMAD4 | NM_005359.3 | 14215 | c.353C>T | p.A118V | + | 18:48575159-48575159 |
| 1341 | SMAD4 | NM_005359.3 | 14216 | c.363_364insA | p.C123fs*2 | + | 18:48575169-48575170 |
| 1342 | SMAD4 | NM_005359.3 | 14217 | c.776_777delCT | p.T259fs*4 | + | 18:48584603-48584604 |
| 1343 | SMAD4 | NM_005359.3 | 14220 | c.1058A>G | p.Y353C | + | 18:48591895-48591895 |
| 1344 | SMAD4 | NM_005359.3 | 14221 | c.1496G>A | p.C499Y | + | 18:48604674-48604674 |
| 1345 | SMAD4 | NM_005359.3 | 14223 | c.1229_1230insCA | p.Q410fs*6 | + | 18:48593478-48593479 |
| 1346 | SMAD4 | NM_005359.3 | 14232 | c.1064A>G | p.D355G | + | 18:48591901-48591901 |
| 1347 | SMAD4 | NM_005359.3 | 14249 | c.1156G>C | p.G386R | + | 18:48593405-48593405 |
| 1348 | SMAD4 | NM_005359.3 | 25274 | c.366_367insA | p.C123fs*2 | + | 18:48575172-48575173 |
| 1349 | SLC34A2 | NM_006424 |  | c.4>5 |  | + | 4:25665823-25667893 |
| 1350 | SDC4 | NM_002999 |  | c.2>3 |  | - | 20:43959005-43961709 |
| 1351 | ROS1 | NM_002944 |  | c.31>35 |  | - | 6:117708942-117715901 |
| 1352 | RET | NM_020975 | 133167 | c.2647G>T | p.A883S | + | 10:43615568-43615568 |
| 1353 | RET | NM_020975 | 21338 | c.2304G>C | p.E768D | + | 10:43613840-43613840 |
| 1354 | RET | NM_020975 | 27040 | c.1886_1891delTGTGCG | p.L629_D631>H | + | 10:43609934-43609939 |
| 1355 | RET | NM_020975 | 29803 | c.1852T>C | p.C618R | + | 10:43609096-43609096 |
| 1356 | RET | NM_020975 | 29804 | c.1858T>C | p.C620R | + | 10:43609102-43609102 |
| 1357 | RET | NM_020975 | 964 | c.1888T>C | p.C630R | + | 10:43609936-43609936 |
| 1358 | RET | NM_020975 | 965 | c.2753T>C | p.M918T | + | 10:43617416-43617416 |
| 1359 | RET | NM_020975 | 966 | c.1900T>C | p.C634R | + | 10:43609948-43609948 |
| 1360 | RET | NM_020975 | 968 | c.1894_1899delGAGCTG | p.E632_L633del | + | 10:43609942-43609947 |
| 1361 | RET | NM_020975 | 974 | c.1901G>A | p.C634Y | + | 10:43609949-43609949 |
| 1362 | RET | NM_020975 | 975 | c.1902C>G | p.C634W | + | 10:43609950-43609950 |
| 1363 | RET | NM_020975 | 977 | c.2647_2648GC>TT | p.A883F | + | 10:43615568-43615569 |
| 1364 | RET | NM_020975 | 978 | c.1892A>G | p.D631G | + | 10:43609940-43609940 |
| 1365 | RET | NM_020975 | 981 | c.2646_2648AGC>TTT | p.A883F | + | 10:43615567-43615569 |
| 1366 | RET | NM_020975 | 982 | c.1895_1897delAGC | p.E632_L633>V | + | 10:43609943-43609945 |
| 1367 | RET | NM_020975 | 983 | c.1893_1898delCGAGCT | p.D631_L633>E | + | 10:43609941-43609946 |
| 1368 | RET | NM_020975 | 984 | c.1834_1860del27 | p.F612_C620del | + | 10:43609078-43609104 |
| 1369 | RET | NM_020630 |  | c.10>12 |  | + | 10:43609003-43612179 |
| 1370 | RB1 | NM_000321 | 1042 | c.2107-2A>G | p.? | + | 13:49037865-49037865 |
| 1371 | RB1 | NM_000321 | 13117 | c.2053C>T | p.Q685* | + | 13:49033916-49033916 |
| 1372 | RB1 | NM_000321 | 28816 | c.940-2A>T | p.? | + | 13:48941628-48941628 |
| 1373 | RB1 | NM_000321 | 861 | c.1981C>T | p.R661W | + | 13:49033844-49033844 |
| 1374 | RB1 | NM_000321 | 868 | c.2242G>T | p.E748* | + | 13:49039164-49039164 |
| 1375 | RB1 | NM_000321 | 869 | c.1980_1983delCCGG | p.L660fs*2 | + | 13:49033843-49033846 |
| 1376 | RB1 | NM_000321 | 870 | c.2028_2040del13 | p.L676fs*16 | + | 13:49033891-49033903 |
| 1377 | RB1 | NM_000321 | 879 | c.1072C>T | p.R358* | + | 13:48942685-48942685 |
| 1378 | RB1 | NM_000321 | 883 | c.2117G>T | p.C706F | + | 13:49037877-49037877 |
| 1379 | RB1 | NM_000321 | 887 | c.1654C>T | p.R552* | + | 13:48955538-48955538 |
| 1380 | RB1 | NM_000321 | 888 | c.1666C>T | p.R556* | + | 13:48955550-48955550 |
| 1381 | RB1 | NM_000321 | 890 | c.409G>T | p.E137* | + | 13:48919244-48919244 |
| 1382 | RB1 | NM_000321 | 891 | c.958C>T | p.R320* | + | 13:48941648-48941648 |
| 1383 | RB1 | NM_000321 | 892 | c.1735C>T | p.R579* | + | 13:49027168-49027168 |
| 1384 | RB1 | NM_000321 | 895 | c.1363C>T | p.R455* | + | 13:48953760-48953760 |
| 1385 | RB1 | NM_000321 | 915 | c.596T>A | p.L199* | + | 13:48923148-48923148 |
| 1386 | RB1 | NM_000321 | 916 | c.2261T>G | p.V754G | + | 13:49039183-49039183 |
| 1387 | RB1 | NM_000321 | 940 | c.2143A>T | p.K715* | + | 13:49037903-49037903 |
| 1388 | PTPN11 | NM_002834.3 | 13000 | c.226G>A | p.E76K | + | 12:112888210-112888210 |
| 1389 | PTPN11 | NM_002834.3 | 13010 | c.178G>C | p.G60R | + | 12:112888162-112888162 |
| 1390 | PTPN11 | NM_002834.3 | 13011 | c.181G>T | p.D61Y | + | 12:112888165-112888165 |
| 1391 | PTPN11 | NM_002834.3 | 13012 | c.181G>A | p.D61N | + | 12:112888165-112888165 |
| 1392 | PTPN11 | NM_002834.3 | 13013 | c.205G>A | p.E69K | + | 12:112888189-112888189 |
| 1393 | PTPN11 | NM_002834.3 | 13014 | c.214G>A | p.A72T | + | 12:112888198-112888198 |
| 1394 | PTPN11 | NM_002834.3 | 13015 | c.215C>T | p.A72V | + | 12:112888199-112888199 |
| 1395 | PTPN11 | NM_002834.3 | 13016 | c.226G>C | p.E76Q | + | 12:112888210-112888210 |
| 1396 | PTPN11 | NM_002834.3 | 13017 | c.227A>G | p.E76G | + | 12:112888211-112888211 |
| 1397 | PTPN11 | NM_002834.3 | 13019 | c.218C>T | p.T73I | + | 12:112888202-112888202 |
| 1398 | PTPN11 | NM_002834.3 | 13020 | c.1504T>C | p.S502P | + | 12:112926884-112926884 |
| 1399 | PTPN11 | NM_002834.3 | 13021 | c.1508G>A | p.G503E | + | 12:112926888-112926888 |
| 1400 | PTPN11 | NM_002834.3 | 13022 | c.182A>T | p.D61V | + | 12:112888166-112888166 |
| 1401 | PTPN11 | NM_002834.3 | 13023 | c.1505C>T | p.S502L | + | 12:112926885-112926885 |
| 1402 | PTPN11 | NM_002834.3 | 13025 | c.227A>T | p.E76V | + | 12:112888211-112888211 |
| 1403 | PTPN11 | NM_002834.3 | 13026 | c.227A>C | p.E76A | + | 12:112888211-112888211 |
| 1404 | PTPN11 | NM_002834.3 | 13027 | c.1508G>C | p.G503A | + | 12:112926888-112926888 |
| 1405 | PTPN11 | NM_002834.3 | 13028 | c.179G>T | p.G60V | + | 12:112888163-112888163 |
| 1406 | PTPN11 | NM_002834.3 | 13029 | c.213T>A | p.F71L | + | 12:112888197-112888197 |
| 1407 | PTPN11 | NM_002834.3 | 13031 | c.1528C>A | p.Q510K | + | 12:112926908-112926908 |
| 1408 | PTPN11 | NM_002834.3 | 13032 | c.172A>T | p.N58Y | + | 12:112888156-112888156 |
| 1409 | PTPN11 | NM_002834.3 | 13034 | c.1472C>T | p.P491L | + | 12:112926852-112926852 |
| 1410 | PTPN11 | NM_002834.3 | 13035 | c.215C>A | p.A72D | + | 12:112888199-112888199 |
| 1411 | PTPN11 | NM_002834.3 | 13039 | c.211T>C | p.F71L | + | 12:112888195-112888195 |
| 1412 | PTPN11 | NM_002834.3 | 13993 | c.155C>G | p.T52S | + | 12:112888139-112888139 |
| 1413 | PTPN11 | NM_002834.3 | 14269 | c.182A>G | p.D61G | + | 12:112888166-112888166 |
| 1414 | PTPN11 | NM_002834.3 | 14271 | c.1508G>T | p.G503V | + | 12:112926888-112926888 |
| 1415 | PTPN11 | NM_002834.3 | 20900 | c.181G>C | p.D61H | + | 12:112888165-112888165 |
| 1416 | PTEN | NM_000314.4 | 13135 | c.323T>G | p.L108R | + | 10:89692839-89692839 |
| 1417 | PTEN | NM_000314.4 | 133713 | c.780_780delA | p.K260fs*6 | + | 10:89717755-89717755 |
| 1418 | PTEN | NM_000314.4 | 13452 | c.863delA | p.E288fs*3 | + | 10:89720712-89720712 |
| 1419 | PTEN | NM_000314.4 | 13981 | c.752G>A | p.G251D | + | 10:89717727-89717727 |
| 1420 | PTEN | NM_000314.4 | 14087 | c.165_209del45 | p.? | + | 10:89685270-89685314 |
| 1421 | PTEN | NM_000314.4 | 17564 | c.766G>A | p.E256K | + | 10:89717741-89717741 |
| 1422 | PTEN | NM_000314.4 | 18663 | c.385G>T | p.G129* | + | 10:89692901-89692901 |
| 1423 | PTEN | NM_000314.4 | 19564 | c.1026+1G>T | p.? | + | 10:89720876-89720876 |
| 1424 | PTEN | NM_000314.4 | 23626 | c.962_963insA | p.N323fs*2 | + | 10:89720811-89720812 |
| 1425 | PTEN | NM_000314.4 | 23643 | c.315T>G | p.C105W | + | 10:89692831-89692831 |
| 1426 | PTEN | NM_000314.4 | 23644 | c.743C>G | p.P248? | + | 10:89717718-89717718 |
| 1427 | PTEN | NM_000314.4 | 23657 | c.1015C>T | p.P339S | + | 10:89720864-89720864 |
| 1428 | PTEN | NM_000314.4 | 26404 | c.723_724insT | p.E242fs*1 | + | 10:89717698-89717699 |
| 1429 | PTEN | NM_000314.4 | 28884 | c.377C>A | p.A126D | + | 10:89692893-89692893 |
| 1430 | PTEN | NM_000314.4 | 28897 | c.520T>G | p.Y174D | + | 10:89711902-89711902 |
| 1431 | PTEN | NM_000314.4 | 28906 | c.871G>T | p.E291* | + | 10:89720720-89720720 |
| 1432 | PTEN | NM_000314.4 | 28914 | c.878delG | p.G293fs*14 | + | 10:89720727-89720727 |
| 1433 | PTEN | NM_000314.4 | 30622 | c.795delA | p.K267fs*9 | + | 10:89717770-89717770 |
| 1434 | PTEN | NM_000314.4 | 33702 | c.530A>G | p.Y177C | + | 10:89711912-89711912 |
| 1435 | PTEN | NM_000314.4 | 39615 | c.950_953delTACT | p.L318fs*2 | + | 10:89720799-89720802 |
| 1436 | PTEN | NM_000314.4 | 41768 | c.179_179delA | p.K60fs*39 | + | 10:89685284-89685284 |
| 1437 | PTEN | NM_000314.4 | 43075 | c.787A>T | p.K263* | + | 10:89717762-89717762 |
| 1438 | PTEN | NM_000314.4 | 43077 | c.203A>G | p.Y68C | + | 10:89685308-89685308 |
| 1439 | PTEN | NM_000314.4 | 43098 | c.969delT | p.N323fs*21 | + | 10:89720818-89720818 |
| 1440 | PTEN | NM_000314.4 | 4885 | c.1011_1014delTTCT | p.F337fs*6 | + | 10:89720860-89720863 |
| 1441 | PTEN | NM_000314.4 | 4889 | c.202_203delTA | p.Y68fs*5 | + | 10:89685307-89685308 |
| 1442 | PTEN | NM_000314.4 | 4894 | c.952_955delCTTA | p.L318fs*2 | + | 10:89720801-89720804 |
| 1443 | PTEN | NM_000314.4 | 4896 | c.956_959delCTTT | p.T319fs*24 | + | 10:89720805-89720808 |
| 1444 | PTEN | NM_000314.4 | 4898 | c.950_953delTACT | p.V317fs*3 | + | 10:89720799-89720802 |
| 1445 | PTEN | NM_000314.4 | 4899 | c.951_954delACTT | p.V317fs*3 | + | 10:89720800-89720803 |
| 1446 | PTEN | NM_000314.4 | 4903 | c.954_957delTACT | p.L318fs*2 | + | 10:89720803-89720806 |
| 1447 | PTEN | NM_000314.4 | 4907 | c.364_368delATTCA | p.I122fs*2 | + | 10:89692880-89692884 |
| 1448 | PTEN | NM_000314.4 | 4908 | c.760_764delAAAGT | p.K254fs*42 | + | 10:89717735-89717739 |
| 1449 | PTEN | NM_000314.4 | 4912 | c.750_751delTG | p.C250fs*2 | + | 10:89717725-89717726 |
| 1450 | PTEN | NM_000314.4 | 4916 | c.953_956delTTAC | p.L318fs*2 | + | 10:89720802-89720805 |
| 1451 | PTEN | NM_000314.4 | 4929 | c.17_18delAA | p.K6fs*4 | + | 10:89624243-89624244 |
| 1452 | PTEN | NM_000314.4 | 4931 | c.881_885delGTCTA | p.S294fs*2 | + | 10:89720730-89720734 |
| 1453 | PTEN | NM_000314.4 | 4932 | c.987_990delTAAA | p.N329fs*14 | + | 10:89720836-89720839 |
| 1454 | PTEN | NM_000314.4 | 4937 | c.16_17delAA | p.K6fs*4 | + | 10:89624242-89624243 |
| 1455 | PTEN | NM_000314.4 | 4942 | c.187_188delAA | p.N63fs*10 | + | 10:89685292-89685293 |
| 1456 | PTEN | NM_000314.4 | 4943 | c.950_954delTACTT | p.V317fs*6 | + | 10:89720799-89720803 |
| 1457 | PTEN | NM_000314.4 | 4958 | c.955_958delACTT | p.T319fs*1 | + | 10:89720804-89720807 |
| 1458 | PTEN | NM_000314.4 | 4969 | c.526_528delTAT | p.Y176del | + | 10:89711908-89711910 |
| 1459 | PTEN | NM_000314.4 | 4976 | c.49_51delCAA | p.Q17del | + | 10:89624275-89624277 |
| 1460 | PTEN | NM_000314.4 | 4982 | c.955_957delACT | p.T319del | + | 10:89720804-89720806 |
| 1461 | PTEN | NM_000314.4 | 4986 | c.741_742insA | p.P248fs*5 | + | 10:89717716-89717717 |
| 1462 | PTEN | NM_000314.4 | 4990 | c.968_969insA | p.N323fs*2 | + | 10:89720817-89720818 |
| 1463 | PTEN | NM_000314.4 | 4994 | c.963_964insA | p.T321fs*3 | + | 10:89720812-89720813 |
| 1464 | PTEN | NM_000314.4 | 5000 | c.170_171insT | p.L57fs*6 | + | 10:89685275-89685276 |
| 1465 | PTEN | NM_000314.4 | 5008 | c.955_956insA | p.T319fs*6 | + | 10:89720804-89720805 |
| 1466 | PTEN | NM_000314.4 | 5025 | c.742_743insC | p.P248fs*5 | + | 10:89717717-89717718 |
| 1467 | PTEN | NM_000314.4 | 5026 | c.742_743insA | p.P248fs*5 | + | 10:89717717-89717718 |
| 1468 | PTEN | NM_000314.4 | 5032 | c.394G>A | p.G132S | + | 10:89692910-89692910 |
| 1469 | PTEN | NM_000314.4 | 5033 | c.389G>A | p.R130Q | + | 10:89692905-89692905 |
| 1470 | PTEN | NM_000314.4 | 5036 | c.202T>C | p.Y68H | + | 10:89685307-89685307 |
| 1471 | PTEN | NM_000314.4 | 5037 | c.37A>G | p.K13E | + | 10:89624263-89624263 |
| 1472 | PTEN | NM_000314.4 | 5039 | c.518G>A | p.R173H | + | 10:89711900-89711900 |
| 1473 | PTEN | NM_000314.4 | 5041 | c.377C>T | p.A126V | + | 10:89692893-89692893 |
| 1474 | PTEN | NM_000314.4 | 5042 | c.182A>G | p.H61R | + | 10:89685287-89685287 |
| 1475 | PTEN | NM_000314.4 | 5044 | c.397G>A | p.V133I | + | 10:89692913-89692913 |
| 1476 | PTEN | NM_000314.4 | 5045 | c.509G>A | p.S170N | + | 10:89711891-89711891 |
| 1477 | PTEN | NM_000314.4 | 5048 | c.196A>G | p.K66E | + | 10:89685301-89685301 |
| 1478 | PTEN | NM_000314.4 | 5049 | c.29G>A | p.S10N | + | 10:89624255-89624255 |
| 1479 | PTEN | NM_000314.4 | 5051 | c.376G>A | p.A126T | + | 10:89692892-89692892 |
| 1480 | PTEN | NM_000314.4 | 5052 | c.499A>G | p.T167A | + | 10:89711881-89711881 |
| 1481 | PTEN | NM_000314.4 | 5078 | c.367C>T | p.H123Y | + | 10:89692883-89692883 |
| 1482 | PTEN | NM_000314.4 | 5082 | c.373A>G | p.K125E | + | 10:89692889-89692889 |
| 1483 | PTEN | NM_000314.4 | 5089 | c.517C>T | p.R173C | + | 10:89711899-89711899 |
| 1484 | PTEN | NM_000314.4 | 5091 | c.493G>A | p.G165R | + | 10:89711875-89711875 |
| 1485 | PTEN | NM_000314.4 | 5092 | c.385G>A | p.G129R | + | 10:89692901-89692901 |
| 1486 | PTEN | NM_000314.4 | 5093 | c.992A>G | p.D331G | + | 10:89720841-89720841 |
| 1487 | PTEN | NM_000314.4 | 5101 | c.40A>G | p.R14G | + | 10:89624266-89624266 |
| 1488 | PTEN | NM_000314.4 | 5106 | c.335T>C | p.L112P | + | 10:89692851-89692851 |
| 1489 | PTEN | NM_000314.4 | 5111 | c.737C>T | p.P246L | + | 10:89717712-89717712 |
| 1490 | PTEN | NM_000314.4 | 5113 | c.314G>A | p.C105Y | + | 10:89692830-89692830 |
| 1491 | PTEN | NM_000314.4 | 5114 | c.494G>A | p.G165E | + | 10:89711876-89711876 |
| 1492 | PTEN | NM_000314.4 | 5121 | c.331T>C | p.W111R | + | 10:89692847-89692847 |
| 1493 | PTEN | NM_000314.4 | 5123 | c.395G>A | p.G132D | + | 10:89692911-89692911 |
| 1494 | PTEN | NM_000314.4 | 5125 | c.755A>G | p.D252G | + | 10:89717730-89717730 |
| 1495 | PTEN | NM_000314.4 | 5127 | c.170T>C | p.L57S | + | 10:89685275-89685275 |
| 1496 | PTEN | NM_000314.4 | 5133 | c.47A>G | p.Y16C | + | 10:89624273-89624273 |
| 1497 | PTEN | NM_000314.4 | 5143 | c.380G>A | p.G127E | + | 10:89692896-89692896 |
| 1498 | PTEN | NM_000314.4 | 5149 | c.511C>T | p.Q171* | + | 10:89711893-89711893 |
| 1499 | PTEN | NM_000314.4 | 5150 | c.640C>T | p.Q214* | + | 10:89717615-89717615 |
| 1500 | PTEN | NM_000314.4 | 5151 | c.1003C>T | p.R335* | + | 10:89720852-89720852 |
| 1501 | PTEN | NM_000314.4 | 5152 | c.388C>T | p.R130* | + | 10:89692904-89692904 |
| 1502 | PTEN | NM_000314.4 | 5153 | c.49C>T | p.Q17* | + | 10:89624275-89624275 |
| 1503 | PTEN | NM_000314.4 | 5156 | c.892C>T | p.Q298* | + | 10:89720741-89720741 |
| 1504 | PTEN | NM_000314.4 | 5157 | c.332G>A | p.W111* | + | 10:89692848-89692848 |
| 1505 | PTEN | NM_000314.4 | 5159 | c.733C>T | p.Q245* | + | 10:89717708-89717708 |
| 1506 | PTEN | NM_000314.4 | 5160 | c.781C>T | p.Q261* | + | 10:89717756-89717756 |
| 1507 | PTEN | NM_000314.4 | 5161 | c.328C>T | p.Q110* | + | 10:89692844-89692844 |
| 1508 | PTEN | NM_000314.4 | 5191 | c.198G>T | p.K66N | + | 10:89685303-89685303 |
| 1509 | PTEN | NM_000314.4 | 5193 | c.384G>T | p.K128N | + | 10:89692900-89692900 |
| 1510 | PTEN | NM_000314.4 | 5199 | c.334C>G | p.L112V | + | 10:89692850-89692850 |
| 1511 | PTEN | NM_000314.4 | 5200 | c.511C>G | p.Q171E | + | 10:89711893-89711893 |
| 1512 | PTEN | NM_000314.4 | 5211 | c.376G>C | p.A126P | + | 10:89692892-89692892 |
| 1513 | PTEN | NM_000314.4 | 5212 | c.319G>T | p.D107Y | + | 10:89692835-89692835 |
| 1514 | PTEN | NM_000314.4 | 5214 | c.361G>C | p.A121P | + | 10:89692877-89692877 |
| 1515 | PTEN | NM_000314.4 | 5216 | c.389G>T | p.R130L | + | 10:89692905-89692905 |
| 1516 | PTEN | NM_000314.4 | 5218 | c.509G>T | p.S170I | + | 10:89711891-89711891 |
| 1517 | PTEN | NM_000314.4 | 5219 | c.388C>G | p.R130G | + | 10:89692904-89692904 |
| 1518 | PTEN | NM_000314.4 | 5220 | c.751G>T | p.G251C | + | 10:89717726-89717726 |
| 1519 | PTEN | NM_000314.4 | 5224 | c.370T>A | p.C124S | + | 10:89692886-89692886 |
| 1520 | PTEN | NM_000314.4 | 5230 | c.758T>A | p.I253N | + | 10:89717733-89717733 |
| 1521 | PTEN | NM_000314.4 | 5232 | c.44G>T | p.R15I | + | 10:89624270-89624270 |
| 1522 | PTEN | NM_000314.4 | 5244 | c.512A>C | p.Q171P | + | 10:89711894-89711894 |
| 1523 | PTEN | NM_000314.4 | 5246 | c.754G>T | p.D252Y | + | 10:89717729-89717729 |
| 1524 | PTEN | NM_000314.4 | 5253 | c.170T>G | p.L57W | + | 10:89685275-89685275 |
| 1525 | PTEN | NM_000314.4 | 5255 | c.1021T>G | p.F341V | + | 10:89720870-89720870 |
| 1526 | PTEN | NM_000314.4 | 5257 | c.166T>G | p.F56V | + | 10:89685271-89685271 |
| 1527 | PTEN | NM_000314.4 | 5266 | c.314G>T | p.C105F | + | 10:89692830-89692830 |
| 1528 | PTEN | NM_000314.4 | 5270 | c.45A>T | p.R15S | + | 10:89624271-89624271 |
| 1529 | PTEN | NM_000314.4 | 5271 | c.371G>C | p.C124S | + | 10:89692887-89692887 |
| 1530 | PTEN | NM_000314.4 | 5273 | c.362C>A | p.A121E | + | 10:89692878-89692878 |
| 1531 | PTEN | NM_000314.4 | 5277 | c.389G>C | p.R130P | + | 10:89692905-89692905 |
| 1532 | PTEN | NM_000314.4 | 5290 | c.1008C>G | p.Y336* | + | 10:89720857-89720857 |
| 1533 | PTEN | NM_000314.4 | 5292 | c.703G>T | p.E235* | + | 10:89717678-89717678 |
| 1534 | PTEN | NM_000314.4 | 5296 | c.195C>A | p.Y65* | + | 10:89685300-89685300 |
| 1535 | PTEN | NM_000314.4 | 5298 | c.19G>T | p.E7* | + | 10:89624245-89624245 |
| 1536 | PTEN | NM_000314.4 | 5313 | c.176C>A | p.S59* | + | 10:89685281-89685281 |
| 1537 | PTEN | NM_000314.4 | 5314 | c.862G>T | p.E288* | + | 10:89720711-89720711 |
| 1538 | PTEN | NM_000314.4 | 5317 | c.195C>G | p.Y65* | + | 10:89685300-89685300 |
| 1539 | PTEN | NM_000314.4 | 53243 | c.964_964delA | p.N323fs*21 | + | 10:89720813-89720813 |
| 1540 | PTEN | NM_000314.4 | 5775 | c.1002_1003CC>TT | p.R335* | + | 10:89720851-89720852 |
| 1541 | PTEN | NM_000314.4 | 5801 | c.968delA | p.N323fs*21 | + | 10:89720817-89720817 |
| 1542 | PTEN | NM_000314.4 | 5811 | c.188delA | p.N63fs*36 | + | 10:89685293-89685293 |
| 1543 | PTEN | NM_000314.4 | 5812 | c.391delA | p.T131fs*3 | + | 10:89692907-89692907 |
| 1544 | PTEN | NM_000314.4 | 5814 | c.993delC | p.D331fs*13 | + | 10:89720842-89720842 |
| 1545 | PTEN | NM_000314.4 | 5816 | c.867delA | p.V290fs*1 | + | 10:89720716-89720716 |
| 1546 | PTEN | NM_000314.4 | 5817 | c.389delG | p.R130fs*4 | + | 10:89692905-89692905 |
| 1547 | PTEN | NM_000314.4 | 5822 | c.738delG | p.P246fs*10 | + | 10:89717713-89717713 |
| 1548 | PTEN | NM_000314.4 | 5823 | c.963delA | p.T321fs*23 | + | 10:89720812-89720812 |
| 1549 | PTEN | NM_000314.4 | 5824 | c.370delT | p.C124fs*10 | + | 10:89692886-89692886 |
| 1550 | PTEN | NM_000314.4 | 5825 | c.517delC | p.R173fs*10 | + | 10:89711899-89711899 |
| 1551 | PTEN | NM_000314.4 | 5840 | c.321_326delTCTTGA | p.L108_D109del | + | 10:89692837-89692842 |
| 1552 | PTEN | NM_000314.4 | 5841 | c.348delC | p.D116fs*18 | + | 10:89692864-89692864 |
| 1553 | PTEN | NM_000314.4 | 5842 | c.357delT | p.V119fs*15 | + | 10:89692873-89692873 |
| 1554 | PTEN | NM_000314.4 | 5844 | c.383_391del9 | p.K128_R130del | + | 10:89692899-89692907 |
| 1555 | PTEN | NM_000314.4 | 5869 | c.1009delT | p.F337fs*7 | + | 10:89720858-89720858 |
| 1556 | PTEN | NM_000314.4 | 5878 | c.46_47insT | p.Y16fs*28 | + | 10:89624272-89624273 |
| 1557 | PTEN | NM_000314.4 | 5885 | c.353_354insA | p.H118fs*8 | + | 10:89692869-89692870 |
| 1558 | PTEN | NM_000314.4 | 5887 | c.711_712insAA | p.K237fs*19 | + | 10:89717686-89717687 |
| 1559 | PTEN | NM_000314.4 | 5888 | c.723_724insTT | p.E242fs*15 | + | 10:89717698-89717699 |
| 1560 | PTEN | NM_000314.4 | 5907 | c.493-12delT | p.? | + | 10:89711863-89711863 |
| 1561 | PTEN | NM_000314.4 | 5915 | c.1-9C>G | p.? | + | 10:89624218-89624218 |
| 1562 | PTEN | NM_000314.4 | 5916 | c.209+5G>A | p.? | + | 10:89685319-89685319 |
| 1563 | PTEN | NM_000314.4 | 5957 | c.1026+1G>T | p.? | + | 10:89720876-89720876 |
| 1564 | PTEN | NM_000314.4 | 5958 | c.165-1G>T | p.? | + | 10:89685269-89685269 |
| 1565 | PTEN | NM_000314.4 | 5959 | c.165-2A>C | p.? | + | 10:89685268-89685268 |
| 1566 | PTEN | NM_000314.4 | 5960 | c.165-1G>A | p.? | + | 10:89685269-89685269 |
| 1567 | PTEN | NM_000314.4 | 5961 | c.493-1G>A | p.? | + | 10:89711874-89711874 |
| 1568 | PTEN | NM_000314.4 | 5974 | c.209+1G>C | p.? | + | 10:89685315-89685315 |
| 1569 | PTEN | NM_000314.4 | 5975 | c.209+1delGT | p.? | + | 10:89685315-89685316 |
| 1570 | PTEN | NM_000314.4 | 5976 | c.209+1G>T | p.? | + | 10:89685315-89685315 |
| 1571 | PTEN | NM_000314.4 | 5979 | c.209+1delGTAA | p.? | + | 10:89685315-89685318 |
| 1572 | PTEN | NM_000314.4 | 6206 | c.314G>C | p.C105S | + | 10:89692830-89692830 |
| 1573 | PTEN | NM_000314.4 | 87314 | c.797_797delA | p.K267fs*9 | + | 10:89717772-89717772 |
| 1574 | PTEN | NM_000314.4 | 88109 | c.724G>T | p.E242* | + | 10:89717699-89717699 |
| 1575 | PTEN | NM_000314.4 | 5033 | c.389G>A | p.R130Q | + | 10:89692905-89692905 |
| 1576 | PTEN | NM_000314.4 | 5216 | c.389G>T | p.R130L | + | 10:89692905-89692905 |
| 1577 | PTEN | NM_000314.4 | 5219 | c.388C>G | p.R130G | + | 10:89692904-89692904 |
| 1578 | PTEN | NM_000314.4 | 5154 | c.697C>T | p.R233* | + | 10:89717672-89717672 |
| 1579 | PTEN | NM_000314.4 | 5089 | c.517C>T | p.R173C | + | 10:89711899-89711899 |
| 1580 | PIK3CA | ENST00000263967 | 125370 | c.1633G>A | p.E545K | + | 3:178936091-178936091 |
| 1581 | PIK3CA | ENST00000263967 | 94985 | c.3129G>C | p.M1043I | + | 3:178952074-178952074 |
| 1582 | PIK3CA | ENST00000263967 | 94986 | c.3140A>G | p.H1047R | + | 3:178952085-178952085 |
| 1583 | PIK3CA | ENST00000263967 | 94987 | c.3140A>T | p.H1047L | + | 3:178952085-178952085 |
| 1584 | PIK3CA | NM_006218.1 | 12458 | c.1634A>C | p.E545A | + | 3:178936092-178936092 |
| 1585 | PIK3CA | NM_006218.1 | 12459 | c.1637A>G | p.Q546R | + | 3:178936095-178936095 |
| 1586 | PIK3CA | NM_006218.1 | 12461 | c.3062A>G | p.Y1021C | + | 3:178952007-178952007 |
| 1587 | PIK3CA | NM_006218.1 | 12463 | c.3128T>C | p.M1043T | + | 3:178952073-178952073 |
| 1588 | PIK3CA | NM_006218.1 | 12464 | c.3204_3205insA | p.N1068fs*4 | + | 3:178952149-178952150 |
| 1589 | PIK3CA | NM_006218.1 | 12580 | c.333G>C | p.K111N | + | 3:178916946-178916946 |
| 1590 | PIK3CA | NM_006218.1 | 12582 | c.1252G>A | p.E418K | + | 3:178927974-178927974 |
| 1591 | PIK3CA | NM_006218.1 | 12584 | c.1357G>A | p.E453K | + | 3:178928079-178928079 |
| 1592 | PIK3CA | NM_006218.1 | 12590 | c.3073A>T | p.T1025S | + | 3:178952018-178952018 |
| 1593 | PIK3CA | NM_006218.1 | 12591 | c.3127A>G | p.M1043V | + | 3:178952072-178952072 |
| 1594 | PIK3CA | NM_006218.1 | 12592 | c.3132T>A | p.N1044K | + | 3:178952077-178952077 |
| 1595 | PIK3CA | NM_006218.1 | 12597 | c.3145G>C | p.G1049R | + | 3:178952090-178952090 |
| 1596 | PIK3CA | NM_006218.1 | 13570 | c.331A>G | p.K111E | + | 3:178916944-178916944 |
| 1597 | PIK3CA | NM_006218.1 | 13594 | c.3068G>A | p.R1023Q | + | 3:178952013-178952013 |
| 1598 | PIK3CA | NM_006218.1 | 17442 | c.1624G>C | p.E542Q | + | 3:178936082-178936082 |
| 1599 | PIK3CA | NM_006218.1 | 17444 | c.3061T>C | p.Y1021H | + | 3:178952006-178952006 |
| 1600 | PIK3CA | NM_006218.1 | 17445 | c.3104C>T | p.A1035V | + | 3:178952049-178952049 |
| 1601 | PIK3CA | NM_006218.1 | 17449 | c.3207A>G | p.*1069_*1069insWKDN* | + | 3:178952152-178952152 |
| 1602 | PIK3CA | NM_006218.1 | 21451 | c.3075C>T | p.T1025T | + | 3:178952020-178952020 |
| 1603 | PIK3CA | NM_006218.1 | 21462 | c.971C>T | p.T324I | + | 3:178921489-178921489 |
| 1604 | PIK3CA | NM_006218.1 | 22540 | c.1031T>G | p.V344G | + | 3:178921549-178921549 |
| 1605 | PIK3CA | NM_006218.1 | 24712 | c.1638G>T | p.Q546H | + | 3:178936096-178936096 |
| 1606 | PIK3CA | NM_006218.1 | 24714 | c.3141T>G | p.H1047Q | + | 3:178952086-178952086 |
| 1607 | PIK3CA | NM_006218.1 | 249872 | c.1631C>A | p.T544N | + | 3:178936089-178936089 |
| 1608 | PIK3CA | NM_006218.1 | 249908 | c.3207+29T>C | p.? | + | 3:178952181-178952181 |
| 1609 | PIK3CA | NM_006218.1 | 25041 | c.1637A>T | p.Q546L | + | 3:178936095-178936095 |
| 1610 | PIK3CA | NM_006218.1 | 25085 | c.3120G>A | p.M1040I | + | 3:178952065-178952065 |
| 1611 | PIK3CA | NM_006218.1 | 25086 | c.3133G>A | p.D1045N | + | 3:178952078-178952078 |
| 1612 | PIK3CA | NM_006218.1 | 27133 | c.1633G>C | p.E545Q | + | 3:178936091-178936091 |
| 1613 | PIK3CA | NM_006218.1 | 27134 | c.3130A>G | p.N1044D | + | 3:178952075-178952075 |
| 1614 | PIK3CA | NM_006218.1 | 27155 | c.1634A>T | p.E545V | + | 3:178936092-178936092 |
| 1615 | PIK3CA | NM_006218.1 | 27156 | c.3137C>A | p.A1046E | + | 3:178952082-178952082 |
| 1616 | PIK3CA | NM_006218.1 | 27158 | c.3146G>C | p.G1049A | + | 3:178952091-178952091 |
| 1617 | PIK3CA | NM_006218.1 | 27273 | c.3136G>A | p.A1046T | + | 3:178952081-178952081 |
| 1618 | PIK3CA | NM_006218.1 | 27374 | c.1635G>C | p.E545D | + | 3:178936093-178936093 |
| 1619 | PIK3CA | NM_006218.1 | 27502 | c.241G>A | p.E81K | + | 3:178916854-178916854 |
| 1620 | PIK3CA | NM_006218.1 | 27505 | c.333G>T | p.K111N | + | 3:178916946-178916946 |
| 1621 | PIK3CA | NM_006218.1 | 28938 | c.3059C>T | p.A1020V | + | 3:178952004-178952004 |
| 1622 | PIK3CA | NM_006218.1 | 29110 | c.3115T>C | p.F1039L | + | 3:178952060-178952060 |
| 1623 | PIK3CA | NM_006218.1 | 29313 | c.3129G>A | p.M1043I | + | 3:178952074-178952074 |
| 1624 | PIK3CA | NM_006218.1 | 36285 | c.3074C>T | p.T1025I | + | 3:178952019-178952019 |
| 1625 | PIK3CA | NM_006218.1 | 36286 | c.3085G>C | p.D1029H | + | 3:178952030-178952030 |
| 1626 | PIK3CA | NM_006218.1 | 36289 | c.3143A>G | p.H1048R | + | 3:178952088-178952088 |
| 1627 | PIK3CA | NM_006218.1 | 6147 | c.1636C>G | p.Q546E | + | 3:178936094-178936094 |
| 1628 | PIK3CA | NM_006218.1 | 746 | c.263G>A | p.R88Q | + | 3:178916876-178916876 |
| 1629 | PIK3CA | NM_006218.1 | 754 | c.1035T>A | p.N345K | + | 3:178921553-178921553 |
| 1630 | PIK3CA | NM_006218.1 | 757 | c.1258T>C | p.C420R | + | 3:178927980-178927980 |
| 1631 | PIK3CA | NM_006218.1 | 759 | c.1616C>G | p.P539R | + | 3:178936074-178936074 |
| 1632 | PIK3CA | NM_006218.1 | 760 | c.1624G>A | p.E542K | + | 3:178936082-178936082 |
| 1633 | PIK3CA | NM_006218.1 | 762 | c.1625A>T | p.E542V | + | 3:178936083-178936083 |
| 1634 | PIK3CA | NM_006218.1 | 763 | c.1633G>A | p.E545K | + | 3:178936091-178936091 |
| 1635 | PIK3CA | NM_006218.1 | 764 | c.1634A>G | p.E545G | + | 3:178936092-178936092 |
| 1636 | PIK3CA | NM_006218.1 | 765 | c.1635G>T | p.E545D | + | 3:178936093-178936093 |
| 1637 | PIK3CA | NM_006218.1 | 766 | c.1636C>A | p.Q546K | + | 3:178936094-178936094 |
| 1638 | PIK3CA | NM_006218.1 | 767 | c.1637A>C | p.Q546P | + | 3:178936095-178936095 |
| 1639 | PIK3CA | NM_006218.1 | 769 | c.2702G>T | p.C901F | + | 3:178947827-178947827 |
| 1640 | PIK3CA | NM_006218.1 | 770 | c.2725T>C | p.F909L | + | 3:178947850-178947850 |
| 1641 | PIK3CA | NM_006218.1 | 771 | c.3073A>G | p.T1025A | + | 3:178952018-178952018 |
| 1642 | PIK3CA | NM_006218.1 | 772 | c.3074C>A | p.T1025N | + | 3:178952019-178952019 |
| 1643 | PIK3CA | NM_006218.1 | 773 | c.3129G>T | p.M1043I | + | 3:178952074-178952074 |
| 1644 | PIK3CA | NM_006218.1 | 774 | c.3139C>T | p.H1047Y | + | 3:178952084-178952084 |
| 1645 | PIK3CA | NM_006218.1 | 775 | c.3140A>G | p.H1047R | + | 3:178952085-178952085 |
| 1646 | PIK3CA | NM_006218.1 | 776 | c.3140A>T | p.H1047L | + | 3:178952085-178952085 |
| 1647 | PIK3CA | NM_006218.1 | 777 | c.3145G>A | p.G1049S | + | 3:178952090-178952090 |
| 1648 | PIK3CA | NM_006218.1 | 778 | c.2102A>C | p.H701P | + | 3:178938860-178938860 |
| 1649 | PIK3CA | NM_006218.1 | 94984 | c.3129G>C | p.M1043I | + | 3:178952074-178952074 |
| 1650 | PIK3CA | NM_006218.1 | 125368 | c.344G>T | p.R115L | + | 3:178916957-178916957 |
| 1651 | PIK3CA | NM_006218.1 | 12579 | c.178C>A | p.Q60K | + | 3:178916791-178916791 |
| 1652 | PIK3CA | NM_006218.1 | 12589 | c.2727C>G | p.F909L | + | 3:178947852-178947852 |
| 1653 | PIK3CA | NM_006218.1 | 14052 | c.332A>G | p.K111R | + | 3:178916945-178916945 |
| 1654 | PIK3CA | NM_006218.1 | 163484 | c.238G>A | p.E80K | + | 3:178916851-178916851 |
| 1655 | PIK3CA | NM_006218.1 | 166154 | c.337_342delCTCAAT | p.L113_N114delLN | + | 3:178916950-178916955 |
| 1656 | PIK3CA | NM_006218.1 | 21448 | c.337_339delCTC | p.L113del | + | 3:178916950-178916952 |
| 1657 | PIK3CA | NM_006218.1 | 230020 | c.1396C>T | p.P466S | + | 3:178928118-178928118 |
| 1658 | PIK3CA | NM_006218.1 | 24710 | c.325_327delGAA | p.E109del | + | 3:178916938-178916940 |
| 1659 | PIK3CA | NM_006218.1 | 24711 | c.335T>A | p.I112N | + | 3:178916948-178916948 |
| 1660 | PIK3CA | NM_006218.1 | 27490 | c.333_335delGAT | p.K111_I112>N | + | 3:178916946-178916948 |
| 1661 | PIK3CA | NM_006218.1 | 27497 | c.323G>A | p.R108H | + | 3:178916936-178916936 |
| 1662 | PIK3CA | NM_006218.1 | 27499 | c.321_323delCCG | p.R108del | + | 3:178916934-178916936 |
| 1663 | PIK3CA | NM_006218.1 | 39166 | c.223C>G | p.Q75E | + | 3:178916836-178916836 |
| 1664 | PIK3CA | NM_006218.1 | 41785 | c.1371C>G | p.N457K | + | 3:178928093-178928093 |
| 1665 | PIK3CA | NM_006218.1 | 51258 | c.1221C>G | p.C407W | + | 3:178927458-178927458 |
| 1666 | PIK3CA | NM_006218.1 | 51259 | c.341_342insCCTCAA | p.N114_R115insLN | + | 3:178916954-178916955 |
| 1667 | PIK3CA | NM_006218.1 | 6145 | c.328G>A | p.E110K | + | 3:178916941-178916941 |
| 1668 | PIK3CA | NM_006218.1 | 6149 | c.1214C>T | p.S405F | + | 3:178927451-178927451 |
| 1669 | PIK3CA | NM_006218.1 | 6150 | c.1352_1366del15 | p.G451_L456>V | + | 3:178928074-178928088 |
| 1670 | PIK3CA | NM_006218.1 | 749 | c.323G>C | p.R108P | + | 3:178916936-178916936 |
| 1671 | PIK3CA | NM_006218.1 | 750 | c.332_334delAGA | p.K111del | + | 3:178916945-178916947 |
| 1672 | PIK3CA | NM_006218.1 | 758 | c.1357G>C | p.E453Q | + | 3:178928079-178928079 |
| 1673 | PIK3CA | NM_006218.1 | 86045 | c.1358A>C | p.E453A | + | 3:178928080-178928080 |
| 1674 | PIK3CA | NM_006218.1 | 86949 | c.1404+10T>G | p.? | + | 3:178928136-178928136 |
| 1675 | PIK3CA | NM_006218.1 | 86950 | c.1404+2_1404+3insT | p.? | + | 3:178928128-178928129 |
| 1676 | PIK3CA | NM_006218.1 | 87210 | c.1252-14C>A | p.? | + | 3:178927960-178927960 |
| 1677 | PDGFRA | NM_006206 | 12396 | c.2524G>T | p.D842Y | + | 4:55152092-55152092 |
| 1678 | PDGFRA | NM_006206 | 12397 | c.2524_2526GAC>TAT | p.D842Y | + | 4:55152092-55152094 |
| 1679 | PDGFRA | NM_006206 | 12398 | c.2524_2525GA>AT | p.D842I | + | 4:55152092-55152093 |
| 1680 | PDGFRA | NM_006206 | 12399 | c.2536G>T | p.D846Y | + | 4:55152104-55152104 |
| 1681 | PDGFRA | NM_006206 | 12400 | c.2527_2538del12 | p.I843_D846del | + | 4:55152095-55152106 |
| 1682 | PDGFRA | NM_006206 | 12401 | c.2524_2532del9 | p.D842_M844del | + | 4:55152092-55152100 |
| 1683 | PDGFRA | NM_006206 | 12402 | c.2530_2541del12 | p.M844_S847del | + | 4:55152098-55152109 |
| 1684 | PDGFRA | NM_006206 | 12405 | c.2521_2526delAGAGAC | p.R841_D842del | + | 4:55152089-55152094 |
| 1685 | PDGFRA | NM_006206 | 12406 | c.2524_2526delGAC | p.D842del | + | 4:55152092-55152094 |
| 1686 | PDGFRA | NM_006206 | 12407 | c.2528_2539del12 | p.I843_S847>T | + | 4:55152096-55152107 |
| 1687 | PDGFRA | NM_006206 | 12408 | c.2526_2538>G | p.D842_D846>E | + | 4:55152094-55152106 |
| 1688 | PDGFRA | NM_006206 | 12411 | c.2524_2536>A | p.D842_D846>N | + | 4:55152092-55152104 |
| 1689 | PDGFRA | NM_006206 | 12417 | c.1697_1711del15 | p.S566_E571>K | + | 4:55141051-55141065 |
| 1690 | PDGFRA | NM_006206 | 12418 | c.1698_1712del15 | p.S566_E571>R | + | 4:55141052-55141066 |
| 1691 | PDGFRA | NM_006206 | 21973 | c.1659_1664delGAGGTA | p.R554_Y555del | + | 4:55141013-55141018 |
| 1692 | PDGFRA | NM_006206 | 22413 | c.2472C>T | p.V824V | + | 4:55152040-55152040 |
| 1693 | PDGFRA | NM_006206 | 22414 | c.1977C>G | p.N659K | + | 4:55144148-55144148 |
| 1694 | PDGFRA | NM_006206 | 22415 | c.1977C>A | p.N659K | + | 4:55144148-55144148 |
| 1695 | PDGFRA | NM_006206 | 22416 | c.1975A>T | p.N659Y | + | 4:55144146-55144146 |
| 1696 | PDGFRA | NM_006206 | 28053 | c.1694_1695insA | p.S566fs*6 | + | 4:55141048-55141049 |
| 1697 | PDGFRA | NM_006206 | 736 | c.2525A>T | p.D842V | + | 4:55152093-55152093 |
| 1698 | PDGFRA | NM_006206 | 737 | c.2524_2535del12 | p.D842_H845del | + | 4:55152092-55152103 |
| 1699 | PDGFRA | NM_006206 | 739 | c.1682T>A | p.V561D | + | 4:55141036-55141036 |
| 1700 | PDGFRA | NM_006206 | 741 | c.1678_1692del15 | p.R560_S564del | + | 4:55141032-55141046 |
| 1701 | PDGFRA | NM_006206 | 743 | c.2021C>T | p.T674I | + | 4:55144547-55144547 |
| 1702 | PDGFRA | NM_006206 | 96892 | c.2526_2537del12 | p.I843_D846del | + | 4:55152094-55152105 |
| 1703 | NTRK1 | NM_002529 |  | c.11>14 |  | + | 1:156844697-156846364 |
| 1704 | NRAS | NM_002524 | 12723 | c.34_35GG>AA | p.G12N | - | 1:115258747-115258748 |
| 1705 | NRAS | NM_002524 | 12725 | c.181_182CA>TT | p.Q61L | - | 1:115256529-115256530 |
| 1706 | NRAS | NM_002524 | 12730 | c.180_181AC>TA | p.Q61K | - | 1:115256530-115256531 |
| 1707 | NRAS | NM_002524 | 28673 | c.179G>A | p.G60E | - | 1:115256532-115256532 |
| 1708 | NRAS | NM_002524 | 30646 | c.182_183AA>TG | p.Q61L | - | 1:115256528-115256529 |
| 1709 | NRAS | NM_002524 | 33693 | c.182_183AA>GG | p.Q61R | - | 1:115256528-115256529 |
| 1710 | NRAS | NM_002524 | 53223 | c.181_183CAA>AAG | p.Q61K | - | 1:115256528-115256530 |
| 1711 | NRAS | NM_002524 | 558 | c.31G>A | p.A11T | - | 1:115258751-115258751 |
| 1712 | NRAS | NM_002524 | 561 | c.34G>C | p.G12R | - | 1:115258748-115258748 |
| 1713 | NRAS | NM_002524 | 562 | c.34G>T | p.G12C | - | 1:115258748-115258748 |
| 1714 | NRAS | NM_002524 | 563 | c.34G>A | p.G12S | - | 1:115258748-115258748 |
| 1715 | NRAS | NM_002524 | 564 | c.35G>A | p.G12D | - | 1:115258747-115258747 |
| 1716 | NRAS | NM_002524 | 565 | c.35G>C | p.G12A | - | 1:115258747-115258747 |
| 1717 | NRAS | NM_002524 | 566 | c.35G>T | p.G12V | - | 1:115258747-115258747 |
| 1718 | NRAS | NM_002524 | 567 | c.36T>C | p.G12G | - | 1:115258746-115258746 |
| 1719 | NRAS | NM_002524 | 569 | c.37G>C | p.G13R | - | 1:115258745-115258745 |
| 1720 | NRAS | NM_002524 | 570 | c.37G>T | p.G13C | - | 1:115258745-115258745 |
| 1721 | NRAS | NM_002524 | 571 | c.37G>A | p.G13S | - | 1:115258745-115258745 |
| 1722 | NRAS | NM_002524 | 572 | c.38_39GT>TC | p.G13V | - | 1:115258743-115258744 |
| 1723 | NRAS | NM_002524 | 573 | c.38G>A | p.G13D | - | 1:115258744-115258744 |
| 1724 | NRAS | NM_002524 | 574 | c.38G>T | p.G13V | - | 1:115258744-115258744 |
| 1725 | NRAS | NM_002524 | 575 | c.38G>C | p.G13A | - | 1:115258744-115258744 |
| 1726 | NRAS | NM_002524 | 576 | c.39T>C | p.G13G | - | 1:115258743-115258743 |
| 1727 | NRAS | NM_002524 | 577 | c.52G>A | p.A18T | - | 1:115258730-115258730 |
| 1728 | NRAS | NM_002524 | 579 | c.181_182CA>AG | p.Q61R | - | 1:115256529-115256530 |
| 1729 | NRAS | NM_002524 | 580 | c.181C>A | p.Q61K | - | 1:115256530-115256530 |
| 1730 | NRAS | NM_002524 | 581 | c.181C>G | p.Q61E | - | 1:115256530-115256530 |
| 1731 | NRAS | NM_002524 | 582 | c.182A>C | p.Q61P | - | 1:115256529-115256529 |
| 1732 | NRAS | NM_002524 | 583 | c.182A>T | p.Q61L | - | 1:115256529-115256529 |
| 1733 | NRAS | NM_002524 | 584 | c.182A>G | p.Q61R | - | 1:115256529-115256529 |
| 1734 | NRAS | NM_002524 | 585 | c.183A>T | p.Q61H | - | 1:115256528-115256528 |
| 1735 | NRAS | NM_002524 | 586 | c.183A>C | p.Q61H | - | 1:115256528-115256528 |
| 1736 | NRAS | NM_002524 | 587 | c.183A>G | p.Q61Q | - | 1:115256528-115256528 |
| 1737 | NRAS | NM_002524 | 589 | c.193A>T | p.S65C | - | 1:115256518-115256518 |
| 1738 | NRAS | NM_002524 | 27174 | c.436G>A | p.A146T | - | 1:115252204-115252204 |
| 1739 | NPM1 | NM_002520.4 | 158600 | c.861_862insTGCA | p.W288fs*>9 | + | 5:170837545-170837546 |
| 1740 | NPM1 | NM_002520.4 | 158604 | c.859_860insTCTG | p.W288fs*>9 | + | 5:170837543-170837544 |
| 1741 | NPM1 | NM_002520.4 | 17559 | c.863_864insTCTG | p.W288fs*12 | + | 5:170837547-170837548 |
| 1742 | NPM1 | NM_002520.4 | 17571 | c.863_864insCATG | p.W288fs*12 | + | 5:170837547-170837548 |
| 1743 | NPM1 | NM_002520.4 | 17572 | c.863_864insCGTG | p.W288fs*12 | + | 5:170837547-170837548 |
| 1744 | NPM1 | NM_002520.4 | 17573 | c.863_864insCCTG | p.W288fs*12 | + | 5:170837547-170837548 |
| 1745 | NPM1 | NM_002520.4 | 19317 | c.863_864insGCCA | p.Q289fs*11 | + | 5:170837547-170837548 |
| 1746 | NPM1 | NM_002520.4 | 19318 | c.885+11delT | p.? | + | 5:170837580-170837580 |
| 1747 | NPM1 | NM_002520.4 | 20806 | c.863_864insCCGG | p.W288fs*12 | + | 5:170837547-170837548 |
| 1748 | NPM1 | NM_002520.4 | 20809 | c.863_864insCCAG | p.W288fs*12 | + | 5:170837547-170837548 |
| 1749 | NPM1 | NM_002520.4 | 20810 | c.863_864insTTTG | p.W288fs*12 | + | 5:170837547-170837548 |
| 1750 | NPM1 | NM_002520.4 | 20811 | c.863_864insCTTG | p.W288fs*12 | + | 5:170837547-170837548 |
| 1751 | NPM1 | NM_002520.4 | 20813 | c.863_864insTCGG | p.W288fs*12 | + | 5:170837547-170837548 |
| 1752 | NPM1 | NM_002520.4 | 20814 | c.863_864insCAGA | p.W288fs*12 | + | 5:170837547-170837548 |
| 1753 | NPM1 | NM_002520.4 | 20815 | c.863_864insTATG | p.W288fs*12 | + | 5:170837547-170837548 |
| 1754 | NPM1 | NM_002520.4 | 20841 | c.863_864insTAAG | p.W288fs*12 | + | 5:170837547-170837548 |
| 1755 | NPM1 | NM_002520.4 | 20851 | c.867_868insAGAC | p.W290fs*10 | + | 5:170837551-170837552 |
| 1756 | NPM1 | NM_002520.4 | 20855 | c.863_864insTGTG | p.W288fs*12 | + | 5:170837547-170837548 |
| 1757 | NPM1 | NM_002520.4 | 20856 | c.863_864insTCAG | p.W288fs*12 | + | 5:170837547-170837548 |
| 1758 | NPM1 | NM_002520.4 | 20861 | c.861_862insTGCT | p.W288fs*12 | + | 5:170837545-170837546 |
| 1759 | NPM1 | NM_002520.4 | 25119 | c.863_864insTACG | p.W288fs*10 | + | 5:170837547-170837548 |
| 1760 | NPM1 | NM_002520.4 | 27065 | c.863_864insTAGG | p.W288fs*12 | + | 5:170837547-170837548 |
| 1761 | NPM1 | NM_002520.4 | 27084 | c.867_868insCGGC | p.W290fs*10 | + | 5:170837551-170837552 |
| 1762 | NPM1 | NM_002520.4 | 28072 | c.868T>G | p.W290G | + | 5:170837552-170837552 |
| 1763 | NPM1 | NM_002520.4 | 28937 | c.860_861insCTGC | p.W288fs*12 | + | 5:170837544-170837545 |
| 1764 | NPM1 | NM_002520.4 | 33980 | c.863_864insCCGA | p.W288fs*10 | + | 5:170837547-170837548 |
| 1765 | NPM1 | NM_002520.4 | 33981 | c.867_868insAGGC | p.W290fs*8 | + | 5:170837551-170837552 |
| 1766 | NPM1 | NM_002520.4 | 85977 | c.867_868insAGGA | p.W290fs*8 | + | 5:170837551-170837552 |
| 1767 | NOTCH1 | NM_017617.2 | 12771 | c.4802T>C | p.L1601P | - | 9:139399344-139399344 |
| 1768 | NOTCH1 | NM_017617.2 | 12772 | c.4724T>C | p.L1575P | - | 9:139399422-139399422 |
| 1769 | NOTCH1 | NM_017617.2 | 12776 | c.7378C>T | p.Q2460* | - | 9:139390816-139390816 |
| 1770 | NOTCH1 | NM_017617.2 | 13040 | c.5030T>A | p.V1677D | - | 9:139397774-139397774 |
| 1771 | NOTCH1 | NM_017617.2 | 13042 | c.4781T>C | p.L1594P | - | 9:139399365-139399365 |
| 1772 | NOTCH1 | NM_017617.2 | 13046 | c.4757T>C | p.L1586P | - | 9:139399389-139399389 |
| 1773 | NOTCH1 | NM_017617.2 | 13047 | c.4735_4737delGTG | p.V1579del | - | 9:139399409-139399411 |
| 1774 | NOTCH1 | NM_017617.2 | 13048 | c.5036T>C | p.L1679P | - | 9:139397768-139397768 |
| 1775 | NOTCH1 | NM_017617.2 | 13050 | c.4778T>C | p.F1593S | - | 9:139399368-139399368 |
| 1776 | NOTCH1 | NM_017617.2 | 13053 | c.4796G>C | p.R1599P | - | 9:139399350-139399350 |
| 1777 | NOTCH1 | NM_017617.2 | 13061 | c.7321C>T | p.Q2441* | - | 9:139390873-139390873 |
| 1778 | NOTCH1 | NM_017617.2 | 13070 | c.7389delC | p.P2463fs*15 | - | 9:139390805-139390805 |
| 1779 | NOTCH1 | NM_017617.2 | 13081 | c.7403C>A | p.S2468* | - | 9:139390791-139390791 |
| 1780 | NOTCH1 | NM_017617.2 | 24673 | c.4724T>C | p.L1575P | - | 9:139399422-139399422 |
| 1781 | NOTCH1 | NM_017617.2 | 24886 | c.7390delG | p.A2464fs*14 | - | 9:139390804-139390804 |
| 1782 | NOTCH1 | NM_017617.2 | 24888 | c.4790T>A | p.L1597H | - | 9:139399356-139399356 |
| 1783 | NOTCH1 | NM_017617.2 | 25836 | c.4730T>A | p.V1577E | - | 9:139399416-139399416 |
| 1784 | NOTCH1 | NM_017617.2 | 25839 | c.4757T>G | p.L1586R | - | 9:139399389-139399389 |
| 1785 | NOTCH1 | NM_017617.2 | 28524 | c.4802T>A | p.L1601Q | - | 9:139399344-139399344 |
| 1786 | NOTCH1 | NM_017617.2 | 28662 | c.7402_7403insGG | p.S2468fs*11 | - | 9:139390791-139390792 |
| 1787 | NFE2L2 | NM_006164 | 132858 | c.86A>G | p.D29G | - | 2:178098959-178098959 |
| 1788 | NFE2L2 | NM_006164 | 124736 | c.85G>C | p.D29H | - | 2:178098960-178098960 |
| 1789 | NFE2L2 | NM_006164 | 2894782 | c.86A>T | p.D29V | - | 2:178098959-178098959 |
| 1790 | NFE2L2 | NM_006164 | 132847 | c.100C>G | p.R34G | - | 2:178098945-178098945 |
| 1791 | NFE2L2 | NM_006164 | 132849 | c.101G>A | p.R34Q | - | 2:178098944-178098944 |
| 1792 | NFE2L2 | NM_006164 | 132848 | c.101G>C | p.R34P | - | 2:178098944-178098944 |
| 1793 | NFE2L2 | NM_006164 | 3961574 | c.101G>T | p.R34L | - | 2:178098944-178098944 |
| 1794 | NFE2L2 | NM_006164 | 1529470 | c.236A>T | p.E79V | - | 2:178098809-178098809 |
| 1795 | NFE2L2 | NM_006164 | 132960 | c.236A>G | p.E79G | - | 2:178098809-178098809 |
| 1796 | NFE2L2 | NM_006164 | 120958 | c.235G>C | p.E79Q | - | 2:178098810-178098810 |
| 1797 | NFE2L2 | NM_006164 | 132851 | c.235G>A | p.E79K | - | 2:178098810-178098810 |
| 1798 | MPRIP | NM_015134 |  | c.21>22 |  | + | 17:17080563-17083402 |
| 1799 | MPL | NM_005373.1 | 142839 | c.1544G>C | p.W515S | + | 1:43815009-43815009 |
| 1800 | MPL | NM_005373.1 | 18918 | c.1544G>T | p.W515L | + | 1:43815009-43815009 |
| 1801 | MPL | NM_005373.1 | 19193 | c.1543_1544TG>AA | p.W515K | + | 1:43815008-43815009 |
| 1802 | MPL | NM_005373.1 | 27286 | c.1514G>A | p.S505N | + | 1:43814979-43814979 |
| 1803 | MPL | NM_005373.1 | 27287 | c.1516G>A | p.A506T | + | 1:43814981-43814981 |
| 1804 | MPL | NM_005373.1 | 27289 | c.1543_1544TG>GC | p.W515A | + | 1:43815008-43815009 |
| 1805 | MPL | NM_005373.1 | 27290 | c.1555G>A | p.A519T | + | 1:43815020-43815020 |
| 1806 | MPL | NM_005373.1 | 28487 | c.1543_1545TGG>AAA | p.W515K | + | 1:43815008-43815010 |
| 1807 | MPL | NM_005373.1 | 29008 | c.1543T>A | p.W515R | + | 1:43815008-43815008 |
| 1808 | MPL | NM_005373.1 | 43212 | c.1543T>C | p.W515R | + | 1:43815008-43815008 |
| 1809 | MLH1 | NM_000249.2 | 26085 | c.1151T>A | p.V384D | + | 3:37067240-37067240 |
| 1810 | MET | NM_000245 | 690 | c.3742T>C | p.Y1248H | + | 7:116423413-116423413 |
| 1811 | MET | NM_000245 | 691 | c.3803T>C | p.M1268T | + | 7:116423474-116423474 |
| 1812 | MET | NM_000245 | 696 | c.3334C>T | p.H1112Y | + | 7:116417463-116417463 |
| 1813 | MET | NM_000245 | 699 | c.3743A>G | p.Y1248C | + | 7:116423414-116423414 |
| 1814 | MET | NM_000245 | 700 | c.3757T>G | p.Y1253D | + | 7:116423428-116423428 |
| 1815 | MET | NM_000245 | 703 | c.3335A>G | p.H1112R | + | 7:116417464-116417464 |
| 1816 | MET | NM_000245 | 706 | c.504G>T | p.E168D | + | 7:116339642-116339642 |
| 1817 | MET | NM_000245 | 707 | c.3029C>T | p.T1010I | + | 7:116411990-116411990 |
| 1818 | MET | NM_000245 | 710 | c.1124A>G | p.N375S | + | 7:116340262-116340262 |
| 1819 | MET | NM_000245 | 201908 | c.3350A>G | p.D1117G | + | 7:116417479-116417479 |
| 1820 | MET | NM_000245 | 29636 | c.2942_3082del141 | p.982_1028del47 | + | 7:116411885-116411895 |
| 1821 | MET | NM_000245 | 49015 | c.2942-20del22 | p.? | + | 7:116411883-116411904 |
| 1822 | MET | NM_000245 | 697 | c.3370C>G | p.H1124D | + | 7:116417499-116417499 |
| 1823 | MET | NM_000245 | 698 | c.3335A>T | p.H1112L | + | 7:116417464-116417464 |
| 1824 | MET | NM_000245 | 701 | c.3390G>A | p.L1130L | + | 7:116417519-116417519 |
| 1825 | MET | NM_000245 | 702 | c.3352A>T | p.N1118Y | + | 7:116417481-116417481 |
| 1826 | MET | NM_001127500 | NOCOSMIC848 | c.2543A>C | p.Y848S | + | 7:116403228-116403228 |
| 1827 | MET | NM_001127500 | NOCOSMIC988 | c.2962C>T | p.R988C | + | 7:116411923-116411923 |
| 1828 | MET | NM_000245 |  |  | X1010_splice | + | 7:116412045-116412045 |
| 1829 | MET | NM_000245 |  |  | X963_splice | + | 7:116411902-116411902 |
| 1830 | MET | NM_000245 |  |  | X1010_splice | + | 7:116412044-116412044 |
| 1831 | MAP2K1 | NM_002755 | 1235481 | c.167A>C | p.Q56P | + | 15:66727451-66727451 |
| 1832 | MAP2K1 | NM_002755 | 1235478 | c.171G>T | p.K57N | + | 15:66727455-66727455 |
| 1833 | MAP2K1 | NM_002755 | 1678546 | c.199G>A | p.D67N | + | 15:66727483-66727483 |
| 1834 | MAP2K1 | NM_002755 | 235614 | c.370C>T | p.P124S | + | 15:66729162-66729162 |
| 1835 | MAP2K1 | NM_002755 | 1315861 | c.371C>T | p.P124L | + | 15:66729163-66729163 |
| 1836 | KRAS | NM_004985 | 12654 | c.30_31insGGA | p.G10_A11insG | - | 12:25398288-25398289 |
| 1837 | KRAS | NM_004985 | 12655 | c.36_37insGGT | p.G12_G13insG | - | 12:25398282-25398283 |
| 1838 | KRAS | NM_004985 | 12703 | c.57G>C | p.L19F | - | 12:25398262-25398262 |
| 1839 | KRAS | NM_004985 | 12721 | c.38_39GC>TT | p.G13V | - | 12:25398280-25398281 |
| 1840 | KRAS | NM_004985 | 12722 | c.40G>A | p.V14I | - | 12:25398279-25398279 |
| 1841 | KRAS | NM_004985 | 12729 | c.180_181TC>CA | p.Q61K | - | 12:25380277-25380278 |
| 1842 | KRAS | NM_004985 | 14209 | c.35_36GT>AC | p.G12D | - | 12:25398283-25398284 |
| 1843 | KRAS | NM_004985 | 19404 | c.436G>A | p.A146T | - | 12:25378562-25378562 |
| 1844 | KRAS | NM_004985 | 19900 | c.437C>T | p.A146V | - | 12:25378561-25378561 |
| 1845 | KRAS | NM_004985 | 19905 | c.436G>C | p.A146P | - | 12:25378562-25378562 |
| 1846 | KRAS | NM_004985 | 19940 | c.351A>C | p.K117N | - | 12:25378647-25378647 |
| 1847 | KRAS | NM_004985 | 20818 | c.57G>T | p.L19F | - | 12:25398262-25398262 |
| 1848 | KRAS | NM_004985 | 219781 | c.39_40insGGC | p.G13_V14insG | - | 12:25398279-25398280 |
| 1849 | KRAS | NM_004985 | 25081 | c.34_35GG>TA | p.G12Y | - | 12:25398284-25398285 |
| 1850 | KRAS | NM_004985 | 28518 | c.176C>G | p.A59G | - | 12:25380282-25380282 |
| 1851 | KRAS | NM_004985 | 28519 | c.351A>T | p.K117N | - | 12:25378647-25378647 |
| 1852 | KRAS | NM_004985 | 34144 | c.34_35GG>AT | p.G12I | - | 12:25398284-25398285 |
| 1853 | KRAS | NM_004985 | 36281 | c.34_36GGT>TGG | p.G12W | - | 12:25398283-25398285 |
| 1854 | KRAS | NM_004985 | 507 | c.24A>G | p.V8V | - | 12:25398295-25398295 |
| 1855 | KRAS | NM_004985 | 510 | c.31G>C | p.A11P | - | 12:25398288-25398288 |
| 1856 | KRAS | NM_004985 | 511 | c.32C>T | p.A11V | - | 12:25398287-25398287 |
| 1857 | KRAS | NM_004985 | 512 | c.34_35GG>TT | p.G12F | - | 12:25398284-25398285 |
| 1858 | KRAS | NM_004985 | 513 | c.34_36GGT>TGC | p.G12C | - | 12:25398283-25398285 |
| 1859 | KRAS | NM_004985 | 514 | c.34_35GG>CT | p.G12L | - | 12:25398284-25398285 |
| 1860 | KRAS | NM_004985 | 515 | c.35_36GT>TC | p.G12V | - | 12:25398283-25398284 |
| 1861 | KRAS | NM_004985 | 516 | c.34G>T | p.G12C | - | 12:25398285-25398285 |
| 1862 | KRAS | NM_004985 | 517 | c.34G>A | p.G12S | - | 12:25398285-25398285 |
| 1863 | KRAS | NM_004985 | 518 | c.34G>C | p.G12R | - | 12:25398285-25398285 |
| 1864 | KRAS | NM_004985 | 519 | c.35_36GT>AA | p.G12E | - | 12:25398283-25398284 |
| 1865 | KRAS | NM_004985 | 520 | c.35G>T | p.G12V | - | 12:25398284-25398284 |
| 1866 | KRAS | NM_004985 | 521 | c.35G>A | p.G12D | - | 12:25398284-25398284 |
| 1867 | KRAS | NM_004985 | 522 | c.35G>C | p.G12A | - | 12:25398284-25398284 |
| 1868 | KRAS | NM_004985 | 523 | c.36T>C | p.G12G | - | 12:25398283-25398283 |
| 1869 | KRAS | NM_004985 | 524 | c.36T>A | p.G12G | - | 12:25398283-25398283 |
| 1870 | KRAS | NM_004985 | 526 | c.37_39GGC>CGT | p.G13R | - | 12:25398280-25398282 |
| 1871 | KRAS | NM_004985 | 527 | c.37G>T | p.G13C | - | 12:25398282-25398282 |
| 1872 | KRAS | NM_004985 | 528 | c.37G>A | p.G13S | - | 12:25398282-25398282 |
| 1873 | KRAS | NM_004985 | 529 | c.37G>C | p.G13R | - | 12:25398282-25398282 |
| 1874 | KRAS | NM_004985 | 530 | c.38_39GC>TG | p.G13V | - | 12:25398280-25398281 |
| 1875 | KRAS | NM_004985 | 531 | c.38_39GC>AT | p.G13D | - | 12:25398280-25398281 |
| 1876 | KRAS | NM_004985 | 532 | c.38G>A | p.G13D | - | 12:25398281-25398281 |
| 1877 | KRAS | NM_004985 | 533 | c.38G>C | p.G13A | - | 12:25398281-25398281 |
| 1878 | KRAS | NM_004985 | 534 | c.38G>T | p.G13V | - | 12:25398281-25398281 |
| 1879 | KRAS | NM_004985 | 535 | c.39C>G | p.G13G | - | 12:25398280-25398280 |
| 1880 | KRAS | NM_004985 | 536 | c.39C>T | p.G13G | - | 12:25398280-25398280 |
| 1881 | KRAS | NM_004985 | 537 | c.39C>A | p.G13G | - | 12:25398280-25398280 |
| 1882 | KRAS | NM_004985 | 538 | c.43G>A | p.G15S | - | 12:25398276-25398276 |
| 1883 | KRAS | NM_004985 | 542 | c.53C>A | p.A18D | - | 12:25398266-25398266 |
| 1884 | KRAS | NM_004985 | 543 | c.64C>A | p.Q22K | - | 12:25398255-25398255 |
| 1885 | KRAS | NM_004985 | 546 | c.175G>A | p.A59T | - | 12:25380283-25380283 |
| 1886 | KRAS | NM_004985 | 547 | c.176C>A | p.A59E | - | 12:25380282-25380282 |
| 1887 | KRAS | NM_004985 | 549 | c.181C>A | p.Q61K | - | 12:25380277-25380277 |
| 1888 | KRAS | NM_004985 | 550 | c.181C>G | p.Q61E | - | 12:25380277-25380277 |
| 1889 | KRAS | NM_004985 | 551 | c.182A>C | p.Q61P | - | 12:25380276-25380276 |
| 1890 | KRAS | NM_004985 | 552 | c.182A>G | p.Q61R | - | 12:25380276-25380276 |
| 1891 | KRAS | NM_004985 | 553 | c.182A>T | p.Q61L | - | 12:25380276-25380276 |
| 1892 | KRAS | NM_004985 | 554 | c.183A>C | p.Q61H | - | 12:25380275-25380275 |
| 1893 | KRAS | NM_004985 | 555 | c.183A>T | p.Q61H | - | 12:25380275-25380275 |
| 1894 | KRAS | NM_004985 | 87280 | c.38_39GC>AA | p.G13E | - | 12:25398280-25398281 |
| 1895 | KRAS | NM_004985 | 87281 | c.36_37TG>AT | p.G13C | - | 12:25398282-25398283 |
| 1896 | KRAS | NM_004985 | 87288 | c.173C>T | p.T58I | - | 12:25380285-25380285 |
| 1897 | KRAS | NM_004985 | 87298 | c.180_181TC>AA | p.Q61K | - | 12:25380277-25380278 |
| 1898 | KRAS | NM_004985 | 87301 | c.33_34insGGAGCT | p.A11_G12insGA | - | 12:25398285-25398286 |
| 1899 | KIT | NM_000222 | 1145 | c.153C>G | p.G51G | + | 4:55561763-55561763 |
| 1900 | KIT | NM_000222 | 1146 | c.154G>A | p.D52N | + | 4:55561764-55561764 |
| 1901 | KIT | NM_000222 | 1155 | c.1588G>A | p.V530I | + | 4:55593431-55593431 |
| 1902 | KIT | NM_000222 | 1169 | c.1651_1665del15 | p.P551_V555del | + | 4:55593585-55593599 |
| 1903 | KIT | NM_000222 | 1177 | c.1653_1670del18 | p.M552_W557del | + | 4:55593587-55593604 |
| 1904 | KIT | NM_000222 | 1179 | c.1654_1659delATGTAT | p.M552_Y553del | + | 4:55593588-55593593 |
| 1905 | KIT | NM_000222 | 1180 | c.1654_1671del18 | p.M552_W557del | + | 4:55593588-55593605 |
| 1906 | KIT | NM_000222 | 1181 | c.1654_1662del9 | p.M552_E554del | + | 4:55593588-55593596 |
| 1907 | KIT | NM_000222 | 1183 | c.1654A>C | p.M552L | + | 4:55593588-55593588 |
| 1908 | KIT | NM_000222 | 1187 | c.1656_1670del15 | p.Y553_W557del | + | 4:55593590-55593604 |
| 1909 | KIT | NM_000222 | 1189 | c.1657_1668del12 | p.Y553_Q556del | + | 4:55593591-55593602 |
| 1910 | KIT | NM_000222 | 1190 | c.1657_1674del18 | p.Y553_K558del | + | 4:55593591-55593608 |
| 1911 | KIT | NM_000222 | 1192 | c.1660_1674del15 | p.E554_K558del | + | 4:55593594-55593608 |
| 1912 | KIT | NM_000222 | 1194 | c.1662_1685del24 | p.V555_E562del | + | 4:55593596-55593619 |
| 1913 | KIT | NM_000222 | 1198 | c.1663_1677del15 | p.V555_V559del | + | 4:55593597-55593611 |
| 1914 | KIT | NM_000222 | 1199 | c.1663_1674del12 | p.V555_K558del | + | 4:55593597-55593608 |
| 1915 | KIT | NM_000222 | 1200 | c.1663_1713del51 | p.V555_I571del | + | 4:55593597-55593647 |
| 1916 | KIT | NM_000222 | 1201 | c.1663_1668delGTACAG | p.V555_Q556del | + | 4:55593597-55593602 |
| 1917 | KIT | NM_000222 | 1202 | c.1663_1680del18 | p.V555_V560del | + | 4:55593597-55593614 |
| 1918 | KIT | NM_000222 | 1203 | c.1663_1719del57 | p.V555_P573del | + | 4:55593597-55593653 |
| 1919 | KIT | NM_000222 | 1204 | c.1666_1680del15 | p.Q556_V560del | + | 4:55593600-55593614 |
| 1920 | KIT | NM_000222 | 1205 | c.1666_1728del63 | p.Q556_L576del | + | 4:55593600-55593662 |
| 1921 | KIT | NM_000222 | 1210 | c.1667_1672delAGTGGA | p.W557_K558del | + | 4:55593601-55593606 |
| 1922 | KIT | NM_000222 | 1211 | c.1668_1673delGTGGAA | p.W557_K558del | + | 4:55593602-55593607 |
| 1923 | KIT | NM_000222 | 1213 | c.1668_1679del12 | p.Q556_V560>H | + | 4:55593602-55593613 |
| 1924 | KIT | NM_000222 | 1216 | c.1669T>A | p.W557R | + | 4:55593603-55593603 |
| 1925 | KIT | NM_000222 | 1217 | c.1669_1674delTGGAAG | p.W557_K558del | + | 4:55593603-55593608 |
| 1926 | KIT | NM_000222 | 1218 | c.1669_1677del9 | p.W557_V559del | + | 4:55593603-55593611 |
| 1927 | KIT | NM_000222 | 1219 | c.1669T>C | p.W557R | + | 4:55593603-55593603 |
| 1928 | KIT | NM_000222 | 1220 | c.1669_1671delTGG | p.W557del | + | 4:55593603-55593605 |
| 1929 | KIT | NM_000222 | 1221 | c.1669T>G | p.W557G | + | 4:55593603-55593603 |
| 1930 | KIT | NM_000222 | 1223 | c.1669_1680del12 | p.W557_V560del | + | 4:55593603-55593614 |
| 1931 | KIT | NM_000222 | 1226 | c.1670_1675delGGAAGG | p.W557_V559>F | + | 4:55593604-55593609 |
| 1932 | KIT | NM_000222 | 1227 | c.1670G>C | p.W557S | + | 4:55593604-55593604 |
| 1933 | KIT | NM_000222 | 1229 | c.1670_1717del48 | p.W557_P573>S | + | 4:55593604-55593651 |
| 1934 | KIT | NM_000222 | 1232 | c.1671_1679del9 | p.W557_V560>C | + | 4:55593605-55593613 |
| 1935 | KIT | NM_000222 | 1233 | c.1671_1676delGAAGGT | p.W557_V559>C | + | 4:55593605-55593610 |
| 1936 | KIT | NM_000222 | 1234 | c.1672_1680del9 | p.K558_V560del | + | 4:55593606-55593614 |
| 1937 | KIT | NM_000222 | 1235 | c.1672_1677delAAGGTT | p.K558_V559del | + | 4:55593606-55593611 |
| 1938 | KIT | NM_000222 | 1238 | c.1672_1692del21 | p.K558_N564del | + | 4:55593606-55593626 |
| 1939 | KIT | NM_000222 | 1239 | c.1672_1686del15 | p.K558_E562del | + | 4:55593606-55593620 |
| 1940 | KIT | NM_000222 | 1241 | c.1673_1678delAGGTTG | p.K558_V560>I | + | 4:55593607-55593612 |
| 1941 | KIT | NM_000222 | 1243 | c.1674G>A | p.K558K | + | 4:55593608-55593608 |
| 1942 | KIT | NM_000222 | 1245 | c.1674_1674G>TCCT | p.K558>NP | + | 4:55593608-55593608 |
| 1943 | KIT | NM_000222 | 1247 | c.1675_1677delGTT | p.V559del | + | 4:55593609-55593611 |
| 1944 | KIT | NM_000222 | 1248 | c.1675_1680delGTTGTT | p.V559_V560del | + | 4:55593609-55593614 |
| 1945 | KIT | NM_000222 | 1249 | c.1675_1695del21 | p.V559_G565del | + | 4:55593609-55593629 |
| 1946 | KIT | NM_000222 | 1250 | c.1675_1683del9 | p.V559_E561del | + | 4:55593609-55593617 |
| 1947 | KIT | NM_000222 | 1251 | c.1675G>A | p.V559I | + | 4:55593609-55593609 |
| 1948 | KIT | NM_000222 | 1252 | c.1676T>A | p.V559D | + | 4:55593610-55593610 |
| 1949 | KIT | NM_000222 | 1253 | c.1676T>G | p.V559G | + | 4:55593610-55593610 |
| 1950 | KIT | NM_000222 | 1254 | c.1676_1684del9 | p.V559_E561del | + | 4:55593610-55593618 |
| 1951 | KIT | NM_000222 | 1255 | c.1676T>C | p.V559A | + | 4:55593610-55593610 |
| 1952 | KIT | NM_000222 | 1256 | c.1678_1680delGTT | p.V560del | + | 4:55593612-55593614 |
| 1953 | KIT | NM_000222 | 1257 | c.1679T>A | p.V560D | + | 4:55593613-55593613 |
| 1954 | KIT | NM_000222 | 1258 | c.1679_1681delTTG | p.V560del | + | 4:55593613-55593615 |
| 1955 | KIT | NM_000222 | 1260 | c.1679T>G | p.V560G | + | 4:55593613-55593613 |
| 1956 | KIT | NM_000222 | 1264 | c.1681G>A | p.E561K | + | 4:55593615-55593615 |
| 1957 | KIT | NM_000222 | 1265 | c.1683G>A | p.E561E | + | 4:55593617-55593617 |
| 1958 | KIT | NM_000222 | 12706 | c.1961T>C | p.V654A | + | 4:55594258-55594258 |
| 1959 | KIT | NM_000222 | 12708 | c.2009C>T | p.T670I | + | 4:55595519-55595519 |
| 1960 | KIT | NM_000222 | 12709 | c.2460T>A | p.D820E | + | 4:55599334-55599334 |
| 1961 | KIT | NM_000222 | 1270 | c.1690_1728del39 | p.N564_L576del | + | 4:55593624-55593662 |
| 1962 | KIT | NM_000222 | 12710 | c.2458G>T | p.D820Y | + | 4:55599332-55599332 |
| 1963 | KIT | NM_000222 | 12711 | c.2447A>G | p.D816G | + | 4:55599321-55599321 |
| 1964 | KIT | NM_000222 | 1273 | c.1696A>G | p.N566D | + | 4:55593630-55593630 |
| 1965 | KIT | NM_000222 | 1275 | c.1698C>T | p.N566N | + | 4:55593632-55593632 |
| 1966 | KIT | NM_000222 | 1277 | c.1702T>G | p.Y568D | + | 4:55593636-55593636 |
| 1967 | KIT | NM_000222 | 1285 | c.1708_1728del21 | p.Y570_L576del | + | 4:55593642-55593662 |
| 1968 | KIT | NM_000222 | 1289 | c.1726_1728delCTT | p.L576del | + | 4:55593660-55593662 |
| 1969 | KIT | NM_000222 | 1290 | c.1727T>C | p.L576P | + | 4:55593661-55593661 |
| 1970 | KIT | NM_000222 | 1293 | c.1729C>T | p.P577S | + | 4:55593663-55593663 |
| 1971 | KIT | NM_000222 | 1294 | c.1735_1737delGAT | p.D579del | + | 4:55593669-55593671 |
| 1972 | KIT | NM_000222 | 1297 | c.1751T>C | p.F584S | + | 4:55593685-55593685 |
| 1973 | KIT | NM_000222 | 1299 | c.1755C>T | p.P585P | + | 4:55593689-55593689 |
| 1974 | KIT | NM_000222 | 1304 | c.1924A>G | p.K642E | + | 4:55594221-55594221 |
| 1975 | KIT | NM_000222 | 1306 | c.2143_2145delAGC | p.S715del | + | 4:55597495-55597497 |
| 1976 | KIT | NM_000222 | 1310 | c.2446G>T | p.D816Y | + | 4:55599320-55599320 |
| 1977 | KIT | NM_000222 | 1311 | c.2446G>C | p.D816H | + | 4:55599320-55599320 |
| 1978 | KIT | NM_000222 | 1312 | c.2446_2447GA>TT | p.D816F | + | 4:55599320-55599321 |
| 1979 | KIT | NM_000222 | 1314 | c.2447A>T | p.D816V | + | 4:55599321-55599321 |
| 1980 | KIT | NM_000222 | 1315 | c.2453A>G | p.K818R | + | 4:55599327-55599327 |
| 1981 | KIT | NM_000222 | 1316 | c.2459A>G | p.D820G | + | 4:55599333-55599333 |
| 1982 | KIT | NM_000222 | 1317 | c.2459A>T | p.D820V | + | 4:55599333-55599333 |
| 1983 | KIT | NM_000222 | 1321 | c.2466T>A | p.N822K | + | 4:55599340-55599340 |
| 1984 | KIT | NM_000222 | 1322 | c.2466T>G | p.N822K | + | 4:55599340-55599340 |
| 1985 | KIT | NM_000222 | 1323 | c.2474T>C | p.V825A | + | 4:55599348-55599348 |
| 1986 | KIT | NM_000222 | 1324 | c.2515G>A | p.E839K | + | 4:55602694-55602694 |
| 1987 | KIT | NM_000222 | 1326 | c.1509_1510insGCCTAT | p.Y503_F504insAY | + | 4:55592185-55592186 |
| 1988 | KIT | NM_000222 | 1327 | c.1654_1668del15 | p.M552_Q556del | + | 4:55593588-55593602 |
| 1989 | KIT | NM_000222 | 1328 | c.1655_1672del18 | p.M552_W557del | + | 4:55593589-55593606 |
| 1990 | KIT | NM_000222 | 1329 | c.1661_1675del15 | p.E554_K558del | + | 4:55593595-55593609 |
| 1991 | KIT | NM_000222 | 1330 | c.1667_1681del15 | p.W557_E561del | + | 4:55593601-55593615 |
| 1992 | KIT | NM_000222 | 1332 | c.1669_1683del15 | p.W557_E561del | + | 4:55593603-55593617 |
| 1993 | KIT | NM_000222 | 1333 | c.1679_1680TT>AG | p.V560E | + | 4:55593613-55593614 |
| 1994 | KIT | NM_000222 | 1334 | c.1702_1722del21 | p.Y568_T574del | + | 4:55593636-55593656 |
| 1995 | KIT | NM_000222 | 133754 | c.1711_1728del18 | p.I571_L576del | + | 4:55593645-55593662 |
| 1996 | KIT | NM_000222 | 133763 | c.1657T>A | p.Y553N | + | 4:55593591-55593591 |
| 1997 | KIT | NM_000222 | 133764 | c.1663G>A | p.V555I | + | 4:55593597-55593597 |
| 1998 | KIT | NM_000222 | 133767 | c.2558G>A | p.W853* | + | 4:55602737-55602737 |
| 1999 | KIT | NM_000222 | 17944 | c.1660G>A | p.E554K | + | 4:55593594-55593594 |
| 2000 | KIT | NM_000222 | 17946 | c.1684G>A | p.E562K | + | 4:55593618-55593618 |
| 2001 | KIT | NM_000222 | 18681 | c.2467T>G | p.Y823D | + | 4:55599341-55599341 |
| 2002 | KIT | NM_000222 | 18682 | c.2468A>G | p.Y823C | + | 4:55599342-55599342 |
| 2003 | KIT | NM_000222 | 18896 | c.1673_1687del15 | p.K558_E562del | + | 4:55593607-55593621 |
| 2004 | KIT | NM_000222 | 19029 | c.1701T>A | p.N567K | + | 4:55593635-55593635 |
| 2005 | KIT | NM_000222 | 19109 | c.2464A>T | p.N822Y | + | 4:55599338-55599338 |
| 2006 | KIT | NM_000222 | 19110 | c.2473G>A | p.V825I | + | 4:55599347-55599347 |
| 2007 | KIT | NM_000222 | 19285 | c.2448C>G | p.D816E | + | 4:55599322-55599322 |
| 2008 | KIT | NM_000222 | 19293 | c.2008_2009AC>GA | p.T670E | + | 4:55595518-55595519 |
| 2009 | KIT | NM_000222 | 19310 | c.1669_1674delTGGAAG | p.W557_K558del | + | 4:55593603-55593608 |
| 2010 | KIT | NM_000222 | 21976 | c.1673_1674insTCC | p.K558>NP | + | 4:55593607-55593608 |
| 2011 | KIT | NM_000222 | 21978 | c.1671_1673GAA>TCC | p.W557_K558>CP | + | 4:55593605-55593607 |
| 2012 | KIT | NM_000222 | 21979 | c.2446_2447GA>AT | p.D816I | + | 4:55599320-55599321 |
| 2013 | KIT | NM_000222 | 21983 | c.1638A>G | p.K546K | + | 4:55593481-55593481 |
| 2014 | KIT | NM_000222 | 22275 | c.1658_1720del63 | p.Y553_T574>S | + | 4:55593592-55593654 |
| 2015 | KIT | NM_000222 | 22379 | c.2458G>C | p.D820H | + | 4:55599332-55599332 |
| 2016 | KIT | NM_000222 | 23418 | c.?_?del? | p.Q556_K558del | + | 4:55593600-55593608 |
| 2017 | KIT | NM_000222 | 23560 | c.1690_1734del45 | p.N564_Y578del | + | 4:55593624-55593668 |
| 2018 | KIT | NM_000222 | 24748 | c.1669_1672TGGA>G | p.W557_K558>E | + | 4:55593603-55593606 |
| 2019 | KIT | NM_000222 | 25064 | c.1928T>C | p.V643A | + | 4:55594225-55594225 |
| 2020 | KIT | NM_000222 | 27069 | c.1674_1679delGGTTGT | p.K558_V560>N | + | 4:55593608-55593613 |
| 2021 | KIT | NM_000222 | 27909 | c.1656_1673del18 | p.Y553_K558> | + | 4:55593590-55593607 |
| 2022 | KIT | NM_000222 | 27910 | c.2433T>C | p.F811F | + | 4:55599307-55599307 |
| 2023 | KIT | NM_000222 | 28026 | c.1621A>C | p.M541L | + | 4:55593464-55593464 |
| 2024 | KIT | NM_000222 | 28637 | c.1672_1676AAGGT>TCTTC | p.K558_V559>SS | + | 4:55593606-55593610 |
| 2025 | KIT | NM_000222 | 29015 | c.1673A>G | p.K558R | + | 4:55593607-55593607 |
| 2026 | KIT | NM_000222 | 29442 | c.1687_1728del42 | p.I563_L576del | + | 4:55593621-55593662 |
| 2027 | KIT | NM_000222 | 30551 | c.1672A>G | p.K558E | + | 4:55593606-55593606 |
| 2028 | KIT | NM_000222 | 33965 | c.1652C>T | p.P551L | + | 4:55593586-55593586 |
| 2029 | KIT | NM_000222 | 33966 | c.1726C>T | p.L576F | + | 4:55593660-55593660 |
| 2030 | KIT | NM_000222 | 36293 | c.1678_1728del51 | p.V560_L576del | + | 4:55593612-55593662 |
| 2031 | KIT | NM_000222 | 36305 | c.1745G>A | p.W582* | + | 4:55593679-55593679 |
| 2032 | KIT | NM_000222 | 36311 | c.1655_1660delTGTATG | p.M552_E554>K | + | 4:55593589-55593594 |
| 2033 | KIT | NM_000222 | 36313 | c.1746G>A | p.W582* | + | 4:55593680-55593680 |
| 2034 | KIT | NM_000222 | 96868 | c.1655_1666del12 | p.M552_Q556>K | + | 4:55593589-55593600 |
| 2035 | KIT | NM_000222 | 96883 | c.1718C>T | p.P573L | + | 4:55593652-55593652 |
| 2036 | KIT | NM_000222 | 96885 | c.1526A>T | p.K509I | + | 4:55592202-55592202 |
| 2037 | KIT | NM_000222 | 96888 | c.1714G>A | p.D572N | + | 4:55593648-55593648 |
| 2038 | KIF5B | NM_004521 |  | c.15>17 |  | - | 10:32322772-32324595 |
| 2039 | KIF5B | NM_004521 |  | c.22>25 |  | - | 10:32327705-32337479 |
| 2040 | KIAA1468 | NM_020854 |  | c.10>11 |  | + | 18:59899564-59912109 |
| 2041 | KDR | NM_002253 | 21091 | c.743C>G | p.A248G | - | 4:55980348-55980348 |
| 2042 | KDR | NM_002253 | 32294 | c.2617G>A | p.G873R | - | 4:55962507-55962507 |
| 2043 | KDR | NM_002253 | 32339 | c.824G>T | p.R275L | - | 4:55979623-55979623 |
| 2044 | KDR | NM_002253 | 48460 | c.3629C>T | p.P1210L | - | 4:55953807-55953807 |
| 2045 | KDR | NM_002253 | 48461 | c.3434G>A | p.G1145E | - | 4:55955111-55955111 |
| 2046 | KDR | NM_002253 | 48462 | c.3418C>A | p.L1140M | - | 4:55955127-55955127 |
| 2047 | KDR | NM_002253 | 48463 | c.2951G>C | p.S984T | - | 4:55960989-55960989 |
| 2048 | KDR | NM_002253 | 48464 | c.2917G>T | p.A973S | - | 4:55961023-55961023 |
| 2049 | KDR | NM_002253 | 48465 | c.1426G>T | p.V476L | - | 4:55972964-55972964 |
| 2050 | KDR | NM_002253 | 48875 | c.3922G>T | p.G1308* | - | 4:55946257-55946257 |
| 2051 | KDR | NM_002253 | 48977 | c.4063_4065delCCT | p.P1355del | - | 4:55946114-55946116 |
| 2052 | JAK3 | NM_000215 | 34196 | c.420+28G>A | p.? | - | 19:17954161-17954161 |
| 2053 | JAK3 | NM_000215 | 34197 | c.420+5G>A | p.? | - | 19:17954184-17954184 |
| 2054 | JAK3 | NM_000215 | 34213 | c.2164G>A | p.V722I | - | 19:17945696-17945696 |
| 2055 | JAK3 | NM_000215 | 34214 | c.1715C>T | p.A572V | - | 19:17948009-17948009 |
| 2056 | JAK3 | NM_000215 | 34215 | c.1718C>T | p.A573V | - | 19:17948006-17948006 |
| 2057 | JAK3 | NM_000215 | 34216 | c.394C>A | p.P132T | - | 19:17954215-17954215 |
| 2058 | JAK2 | ENST00000381652 | 12600 | c.1849G>T | p.V617F | + | 9:5073770-5073770 |
| 2059 | JAK2 | ENST00000381652 | 25834 | c.1848_1849TG>CT | p.V617F | + | 9:5073769-5073770 |
| 2060 | JAK2 | ENST00000381652 | 27063 | c.1860C>A | p.D620E | + | 9:5073781-5073781 |
| 2061 | JAK2 | ENST00000381652 | 29118 | c.1852T>C | p.C618R | + | 9:5073773-5073773 |
| 2062 | JAK2 | ENST00000381652 | 51411 | c.1831T>G | p.L611V | + | 9:5073752-5073752 |
| 2063 | IDH2 | NM_002168.2 | 133672 | c.516G>C | p.R172S | - | 15:90631837-90631837 |
| 2064 | IDH2 | NM_002168.2 | 33731 | c.514A>G | p.R172G | - | 15:90631839-90631839 |
| 2065 | IDH2 | NM_002168.2 | 33732 | c.515G>T | p.R172M | - | 15:90631838-90631838 |
| 2066 | IDH2 | NM_002168.2 | 33733 | c.515G>A | p.R172K | - | 15:90631838-90631838 |
| 2067 | IDH2 | NM_002168.2 | 34039 | c.514A>T | p.R172W | - | 15:90631839-90631839 |
| 2068 | IDH2 | NM_002168.2 | 34090 | c.516G>T | p.R172S | - | 15:90631837-90631837 |
| 2069 | IDH2 | NM_002168.2 | 41590 | c.419G>A | p.R140Q | - | 15:90631934-90631934 |
| 2070 | IDH2 | NM_002168.2 | 41875 | c.419G>T | p.R140L | - | 15:90631934-90631934 |
| 2071 | IDH2 | NM_002168.2 | 41877 | c.418C>T | p.R140W | - | 15:90631935-90631935 |
| 2072 | IDH2 | NM_002168.2 | 227366 | c.415A>T | p.I139F | - | 15:90631938-90631938 |
| 2073 | IDH2 | NM_002168.2 | 86959 | c.472C>A | p.P158T | - | 15:90631881-90631881 |
| 2074 | IDH2 | NM_002168.2 | 86960 | c.512G>A | p.G171D | - | 15:90631841-90631841 |
| 2075 | IDH1 | NM_005896.2 | 28746 | c.395G>A | p.R132H | - | 2:209113112-209113112 |
| 2076 | IDH1 | NM_005896.2 | 28747 | c.394C>T | p.R132C | - | 2:209113113-209113113 |
| 2077 | IDH1 | NM_005896.2 | 28748 | c.394C>A | p.R132S | - | 2:209113113-209113113 |
| 2078 | IDH1 | NM_005896.2 | 28749 | c.394C>G | p.R132G | - | 2:209113113-209113113 |
| 2079 | IDH1 | NM_005896.2 | 28750 | c.395G>T | p.R132L | - | 2:209113112-209113112 |
| 2080 | IDH1 | NM_005896.2 | 86993 | c.395_396GT>AC | p.R132H | - | 2:209113111-209113112 |
| 2081 | IDH1 | NM_005896.2 | 242544 | c.356G>A | p.R119Q | - | 2:209113151-209113151 |
| 2082 | IDH1 | NM_005896.2 | 28751 | c.394_395CG>GT | p.R132V | - | 2:209113112-209113113 |
| 2083 | IDH1 | NM_005896.2 | 51542 | c.368G>A | p.G123E | - | 2:209113139-209113139 |
| 2084 | IDH1 | NM_005896.2 | 96532 | c.390A>G | p.I130M | - | 2:209113117-209113117 |
| 2085 | IDH1 | NM_005896.2 | 96533 | c.399T>A | p.H133Q | - | 2:209113108-209113108 |
| 2086 | IDH1 | NM_005896.2 | 96534 | c.401C>A | p.A134D | - | 2:209113106-209113106 |
| 2087 | IDH1 | NM_005896.2 | 96922 | c.367G>A | p.G123R | - | 2:209113140-209113140 |
| 2088 | IDH1 | NM_005896.2 | 97049 | c.347A>G | p.N116S | - | 2:209113160-209113160 |
| 2089 | IDH1 | NM_005896 | NOCOSMIC105 | c.315C>T | p.G105G | - | 2:209113192-209113192 |
| 2090 | HRAS | ENST00000397594 | 123649 | c.181C>A | p.Q61K | - | 11:533875-533875 |
| 2091 | HRAS | ENST00000397594 | 99664 | c.182A>T | p.Q61L | - | 11:533874-533874 |
| 2092 | HRAS | ENST00000397594 | 99915 | c.35G>A | p.G12D | - | 11:534288-534288 |
| 2093 | HRAS | NM_005343 | 249860 | c.81T>C | p.H27H | - | 11:534242-534242 |
| 2094 | HRAS | NM_005343 | 33692 | c.185A>G | p.E62G | - | 11:533871-533871 |
| 2095 | HRAS | NM_005343 | 33695 | c.182_183AG>GA | p.Q61R | - | 11:533873-533874 |
| 2096 | HRAS | NM_005343 | 479 | c.33C>T | p.A11A | - | 11:534290-534290 |
| 2097 | HRAS | NM_005343 | 480 | c.34G>A | p.G12S | - | 11:534289-534289 |
| 2098 | HRAS | NM_005343 | 481 | c.34G>T | p.G12C | - | 11:534289-534289 |
| 2099 | HRAS | NM_005343 | 482 | c.34G>C | p.G12R | - | 11:534289-534289 |
| 2100 | HRAS | NM_005343 | 483 | c.35G>T | p.G12V | - | 11:534288-534288 |
| 2101 | HRAS | NM_005343 | 484 | c.35G>A | p.G12D | - | 11:534288-534288 |
| 2102 | HRAS | NM_005343 | 485 | c.35G>C | p.G12A | - | 11:534288-534288 |
| 2103 | HRAS | NM_005343 | 486 | c.37G>C | p.G13R | - | 11:534286-534286 |
| 2104 | HRAS | NM_005343 | 487 | c.37G>A | p.G13S | - | 11:534286-534286 |
| 2105 | HRAS | NM_005343 | 488 | c.37G>T | p.G13C | - | 11:534286-534286 |
| 2106 | HRAS | NM_005343 | 489 | c.38G>T | p.G13V | - | 11:534285-534285 |
| 2107 | HRAS | NM_005343 | 490 | c.38G>A | p.G13D | - | 11:534285-534285 |
| 2108 | HRAS | NM_005343 | 496 | c.181C>A | p.Q61K | - | 11:533875-533875 |
| 2109 | HRAS | NM_005343 | 498 | c.182A>T | p.Q61L | - | 11:533874-533874 |
| 2110 | HRAS | NM_005343 | 499 | c.182A>G | p.Q61R | - | 11:533874-533874 |
| 2111 | HRAS | NM_005343 | 500 | c.182A>C | p.Q61P | - | 11:533874-533874 |
| 2112 | HRAS | NM_005343 | 501 | c.182_183AG>GT | p.Q61R | - | 11:533873-533874 |
| 2113 | HRAS | NM_005343 | 502 | c.183G>T | p.Q61H | - | 11:533873-533873 |
| 2114 | HRAS | NM_005343 | 503 | c.183G>C | p.Q61H | - | 11:533873-533873 |
| 2115 | HRAS | NM_005343 | 52978 | c.182_183AG>TA | p.Q61L | - | 11:533873-533874 |
| 2116 | HRAS | NM_005343 | 52979 | c.181_182CA>AG | p.Q61R | - | 11:533874-533875 |
| 2117 | HNF1A | NM_000545.3 | 21471 | c.617G>T | p.W206L | + | 12:121431413-121431413 |
| 2118 | HNF1A | NM_000545.3 | 21477 | c.817A>G | p.K273E | + | 12:121432070-121432070 |
| 2119 | HNF1A | NM_000545.3 | 21478 | c.618G>T | p.W206C | + | 12:121431414-121431414 |
| 2120 | HNF1A | NM_000545.3 | 24692 | c.787C>T | p.R263C | + | 12:121432040-121432040 |
| 2121 | HNF1A | NM_000545.3 | 24900 | c.632A>C | p.Q211P | + | 12:121431428-121431428 |
| 2122 | HNF1A | NM_000545.3 | 24915 | c.607C>T | p.R203C | + | 12:121431403-121431403 |
| 2123 | HNF1A | NM_000545.3 | 24918 | c.618G>C | p.W206C | + | 12:121431414-121431414 |
| 2124 | HNF1A | NM_000545.3 | 24923 | c.779C>T | p.T260M | + | 12:121432032-121432032 |
| 2125 | HNF1A | NM_000545.3 | 24931 | c.620G>A | p.G207D | + | 12:121431416-121431416 |
| 2126 | HNF1A | NM_000545.3 | 24933 | c.815G>A | p.R272H | + | 12:121432068-121432068 |
| 2127 | GNAS | ENST00000371100 | 123397 | c.2530C>T | p.R844C | + | 20:57484420-57484420 |
| 2128 | GNAS | ENST00000371100 | 94388 | c.2531G>A | p.R844H | + | 20:57484421-57484421 |
| 2129 | GNAS | ENST00000371100 | 192558 | c.2628G>T | p.K876N | + | 20:57484615-57484615 |
| 2130 | GNAS | NM_000516.3 | 27887 | c.601C>T | p.R201C | + | 20:57484420-57484420 |
| 2131 | GNAS | NM_000516.3 | 27888 | c.680A>T | p.Q227L | + | 20:57484596-57484596 |
| 2132 | GNAS | NM_000516.3 | 27895 | c.602G>A | p.R201H | + | 20:57484421-57484421 |
| 2133 | GNAS | NM_000516.3 | 27896 | c.680A>G | p.Q227R | + | 20:57484596-57484596 |
| 2134 | GNAS | NM_000516.3 | 27899 | c.601C>A | p.R201S | + | 20:57484420-57484420 |
| 2135 | GNAS | NM_000516.3 | 27900 | c.681G>T | p.Q227H | + | 20:57484597-57484597 |
| 2136 | GNAS | NM_000516.3 | 192557 | c.699G>T | p.K233N | + | 20:57484615-57484615 |
| 2137 | GNAS | NM_000516.3 | 28618 | c.679C>A | p.Q227K | + | 20:57484595-57484595 |
| 2138 | GNAS | NM_000516 | NOCOSMIC227 | c.679C>G | p.Q227E | + | 20:57484595-57484595 |
| 2139 | GNAQ | NM_002072.2 | 28757 | c.626A>T | p.Q209L | - | 9:80409488-80409488 |
| 2140 | GNAQ | NM_002072.2 | 28758 | c.626A>C | p.Q209P | - | 9:80409488-80409488 |
| 2141 | GNAQ | NM_002072.2 | 28759 | c.625_626CA>TT | p.Q209L | - | 9:80409488-80409489 |
| 2142 | GNAQ | NM_002072.2 | 132932 | c.625C>A | p.Q209K | - | 9:80409489-80409489 |
| 2143 | GNAQ | NM_002072.2 | 28760 | c.626A>G | p.Q209R | - | 9:80409488-80409488 |
| 2144 | GNAQ | NM_002072.2 | 28770 | c.627A>T | p.Q209H | - | 9:80409487-80409487 |
| 2145 | GNA11 | NM_002067.1 | 52969 | c.626A>T | p.Q209L | + | 19:3118942-3118942 |
| 2146 | GNA11 | NM_002067.1 | 52970 | c.626A>C | p.Q209P | + | 19:3118942-3118942 |
| 2147 | GNA11 | NM_002067.1 | 238583 | c.625C>A | p.Q209K | + | 19:3118941-3118941 |
| 2148 | GNA11 | NM_002067.1 | 52971 | c.626_627AG>TA | p.Q209L | + | 19:3118942-3118943 |
| 2149 | GNA11 | NM_002067.1 | 52972 | c.626_627AG>TT | p.Q209L | + | 19:3118942-3118943 |
| 2150 | FLT3 | Z26652 | 158601 | c.1782_1783ins TCAGATAATGAGTACTTCTACGTTGATTTC | p.F594_R595ins SDNEYFYVDF | - | 13:28608273-28608274 |
| 2151 | FLT3 | Z26652 | 158603 | c.1797_1798ins CCGGCTCCTCAGATAATGAGTACTTCTACGTTGATTTCAGAGAATATGAATATCGCC | p.Y599_D600ins PAPQIMSTSTLISENMNIA | - | 13:28608258-28608259 |
| 2152 | FLT3 | Z26652 | 158605 | c.1799_1800ins ATATGAATATGAATATGAATATGA | p.Y599_D600ins EYEYEYEY | - | 13:28608256-28608257 |
| 2153 | FLT3 | Z26652 | 19522 | c.1775T>C | p.V592A | - | 13:28608281-28608281 |
| 2154 | FLT3 | Z26652 | 19686 | c.2508C>G | p.I836M | - | 13:28592637-28592637 |
| 2155 | FLT3 | Z26652 | 19692 | c.2525A>G | p.Y842C | - | 13:28592620-28592620 |
| 2156 | FLT3 | Z26652 | 19836 | c.2508_2510delCAT | p.I836del | - | 13:28592635-28592637 |
| 2157 | FLT3 | Z26652 | 24530 | c.2506_2508ATC>TTT | p.I836F | - | 13:28592637-28592639 |
| 2158 | FLT3 | Z26652 | 24531 | c.2509_2510AT>CC | p.M837P | - | 13:28592635-28592636 |
| 2159 | FLT3 | Z26652 | 25248 | c.2492G>A | p.G831E | - | 13:28592653-28592653 |
| 2160 | FLT3 | Z26652 | 27650 | c.2504A>C | p.D835A | - | 13:28592641-28592641 |
| 2161 | FLT3 | Z26652 | 27906 | c.1796A>T | p.Y599F | - | 13:28608260-28608260 |
| 2162 | FLT3 | Z26652 | 28042 | c.1352C>T | p.S451F | - | 13:28610138-28610138 |
| 2163 | FLT3 | Z26652 | 28044 | c.1715A>G | p.Y572C | - | 13:28608341-28608341 |
| 2164 | FLT3 | Z26652 | 28047 | c.2501G>A | p.R834Q | - | 13:28592644-28592644 |
| 2165 | FLT3 | Z26652 | 783 | c.2503G>T | p.D835Y | - | 13:28592642-28592642 |
| 2166 | FLT3 | Z26652 | 784 | c.2504A>T | p.D835V | - | 13:28592641-28592641 |
| 2167 | FLT3 | Z26652 | 785 | c.2503G>C | p.D835H | - | 13:28592642-28592642 |
| 2168 | FLT3 | Z26652 | 786 | c.2039C>T | p.A680V | - | 13:28602329-28602329 |
| 2169 | FLT3 | Z26652 | 787 | c.2505T>A | p.D835E | - | 13:28592640-28592640 |
| 2170 | FLT3 | Z26652 | 788 | c.2505T>G | p.D835E | - | 13:28592640-28592640 |
| 2171 | FLT3 | Z26652 | 789 | c.2503G>A | p.D835N | - | 13:28592642-28592642 |
| 2172 | FLT3 | Z26652 | 796 | c.2503_2505delGAT | p.D835del | - | 13:28592640-28592642 |
| 2173 | FLT3 | Z26652 | 797 | c.2506_2508delATC | p.I836del | - | 13:28592637-28592639 |
| 2174 | FLT3 | Z26652 | 850 | c.2520_2521insGGATCC | p.S840_N841insGS | - | 13:28592624-28592625 |
| 2175 | FLT3 | Z26652 | 19737 | c.1803_1804ins81 | p.L601_K602ins27 | - | 13:28608252-28608253 |
| 2176 | FLT3 | Z26652 | 19790 | c.1807_1808ins18 | p.K602_W603insYEYDLK | - | 13:28608248-28608249 |
| 2177 | FLT3 | Z26652 | 27979 | c.1788_1789ins36 | p.E596_Y597ins12 | - | 13:28608267-28608268 |
| 2178 | FLT3 | Z26652 | 28771 | c.1811_1812ins30 | p.W603_E604insDREYEYDLKW | - | 13:28608244-28608245 |
| 2179 | FLT3 | Z26652 | 28921 | c.1798_1799ins45 | p.Y599_D600ins15 | - | 13:28608257-28608258 |
| 2180 | FGFR3 | NM_000142 | 17461 | c.1111A>T | p.S371C | + | 4:1806092-1806092 |
| 2181 | FGFR3 | NM_000142 | 24802 | c.2089G>T | p.G697C | + | 4:1808331-1808331 |
| 2182 | FGFR3 | NM_000142 | 24842 | c.1138G>A | p.G380R | + | 4:1806119-1806119 |
| 2183 | FGFR3 | NM_000142 | 29438 | c.1921G>A | p.D641N | + | 4:1807862-1807862 |
| 2184 | FGFR3 | NM_000142 | 29446 | c.753C>T | p.H251H | + | 4:1803575-1803575 |
| 2185 | FGFR3 | NM_000142 | 714 | c.742C>T | p.R248C | + | 4:1803564-1803564 |
| 2186 | FGFR3 | NM_000142 | 715 | c.746C>G | p.S249C | + | 4:1803568-1803568 |
| 2187 | FGFR3 | NM_000142 | 716 | c.1108G>T | p.G370C | + | 4:1806089-1806089 |
| 2188 | FGFR3 | NM_000142 | 718 | c.1118A>G | p.Y373C | + | 4:1806099-1806099 |
| 2189 | FGFR3 | NM_000142 | 719 | c.1948A>G | p.K650E | + | 4:1807889-1807889 |
| 2190 | FGFR3 | NM_000142 | 720 | c.1949A>T | p.K650M | + | 4:1807890-1807890 |
| 2191 | FGFR3 | NM_000142 | 721 | c.1172C>A | p.A391E | + | 4:1806153-1806153 |
| 2192 | FGFR3 | NM_000142 | 722 | c.1107G>T | p.A369A | + | 4:1806088-1806088 |
| 2193 | FGFR3 | NM_000142 | 724 | c.1150T>C | p.F384L | + | 4:1806131-1806131 |
| 2194 | FGFR3 | NM_000142 | 726 | c.1948A>C | p.K650Q | + | 4:1807889-1807889 |
| 2195 | FGFR3 | NM_000142 | 729 | c.2381_2381T>GA | p.L794fs*23 | + | 4:1808949-1808949 |
| 2196 | FGFR3 | NM_000142 | 731 | c.1949A>C | p.K650T | + | 4:1807890-1807890 |
| 2197 | FGFR3 | NM_000142 |  | c.16>17 |  | + | 4:1808272-1808661 |
| 2198 | FGFR2 | NM_000141.2 | 36901 | c.929A>G | p.K310R | - | 10:123279503-123279503 |
| 2199 | FGFR2 | NM_000141.2 | 36902 | c.1647T>G | p.N549K | - | 10:123258034-123258034 |
| 2200 | FGFR2 | NM_000141.2 | 36903 | c.755C>G | p.S252W | - | 10:123279677-123279677 |
| 2201 | FGFR2 | NM_000141.2 | 36904 | c.1124A>G | p.Y375C | - | 10:123274794-123274794 |
| 2202 | FGFR2 | NM_000141.2 | 36905 | c.1115C>G | p.S372C | - | 10:123274803-123274803 |
| 2203 | FGFR2 | NM_000141.2 | 36906 | c.1144T>C | p.C382R | - | 10:123274774-123274774 |
| 2204 | FGFR2 | NM_000141.2 | 36912 | c.1647T>A | p.N549K | - | 10:123258034-123258034 |
| 2205 | FGFR2 | NM_000141.2 | 49170 | c.758C>G | p.P253R | - | 10:123279674-123279674 |
| 2206 | FGFR2 | NM_000141 |  | c.17>18 |  | - | 10:123353222-123357972 |
| 2207 | FGFR1 | NM_000604 | 12834 | c.754C>A | p.P252T | - | 8:38282209-38282209 |
| 2208 | FGFR1 | NM_000604 | 601 | c.374C>T | p.S125L | - | 8:38285938-38285938 |
| 2209 | FBXW7 | NM_018315.2 | 117308 | c.1154G>A | p.R385H | - | 4:153249384-153249384 |
| 2210 | FBXW7 | NM_018315.2 | 170725 | c.1153C>T | p.R385C | - | 4:153249385-153249385 |
| 2211 | FBXW7 | NM_018315.2 | 74637 | c.1273C>T | p.R425C | - | 4:153247289-153247289 |
| 2212 | FBXW7 | NM_018315.2 | 99603 | c.1273C>G | p.R425G | - | 4:153247289-153247289 |
| 2213 | FBXW7 | ENST00000534231 | 108571 | c.796C>T | p.R266C | - | 4:153247289-153247289 |
| 2214 | FBXW7 | ENST00000534231 | 117309 | c.677G>A | p.R226H | - | 4:153249384-153249384 |
| 2215 | FBXW7 | ENST00000534231 | 170726 | c.676C>T | p.R226C | - | 4:153249385-153249385 |
| 2216 | FBXW7 | ENST00000534231 | 99605 | c.796C>G | p.R266G | - | 4:153247289-153247289 |
| 2217 | FBXW7 | ENST00000281708 | 108572 | c.1513C>T | p.R505C | - | 4:153247289-153247289 |
| 2218 | FBXW7 | ENST00000281708 | 117310 | c.1394G>A | p.R465H | - | 4:153249384-153249384 |
| 2219 | FBXW7 | ENST00000281708 | 170727 | c.1393C>T | p.R465C | - | 4:153249385-153249385 |
| 2220 | FBXW7 | ENST00000281708 | 99606 | c.1513C>G | p.R505G | - | 4:153247289-153247289 |
| 2221 | FBXW7 | NM_033632.1 | 133115 | c.1393_1394CG>TA | p.R465Y | - | 4:153249384-153249385 |
| 2222 | FBXW7 | NM_033632.1 | 22932 | c.1393C>T | p.R465C | - | 4:153249385-153249385 |
| 2223 | FBXW7 | NM_033632.1 | 22965 | c.1394G>A | p.R465H | - | 4:153249384-153249384 |
| 2224 | FBXW7 | NM_033632.1 | 22971 | c.832C>T | p.R278* | - | 4:153258983-153258983 |
| 2225 | FBXW7 | NM_033632.1 | 22973 | c.1177C>T | p.R393* | - | 4:153250883-153250883 |
| 2226 | FBXW7 | NM_033632.1 | 22974 | c.1436G>A | p.R479Q | - | 4:153247366-153247366 |
| 2227 | FBXW7 | NM_033632.1 | 22975 | c.1513C>T | p.R505C | - | 4:153247289-153247289 |
| 2228 | FBXW7 | NM_033632.1 | 22979 | c.1745C>T | p.S582L | - | 4:153245446-153245446 |
| 2229 | FBXW7 | NM_033632.1 | 23000 | c.1514G>T | p.R505L | - | 4:153247288-153247288 |
| 2230 | FBXW7 | NM_033632.1 | 25812 | c.1514G>A | p.R505H | - | 4:153247288-153247288 |
| 2231 | FBXW7 | NM_033632.1 | 27055 | c.1510G>A | p.V504I | - | 4:153247292-153247292 |
| 2232 | FBXW7 | NM_033632.1 | 33762 | c.1394G>T | p.R465L | - | 4:153249384-153249384 |
| 2233 | FBXW7 | NM_033632.1 | 99604 | c.1513C>G | p.R505G | - | 4:153247289-153247289 |
| 2234 | EZR | NM_003379 |  | c.10>11 |  | - | 6:159206340-159210403 |
| 2235 | EZH2 | ENST00000350995 | 139744 | c.1804T>A | p.Y602N | - | 7:148508728-148508728 |
| 2236 | EZH2 | ENST00000350995 | 220730 | c.1805A>C | p.Y602S | - | 7:148508727-148508727 |
| 2237 | EZH2 | ENST00000350995 | 220731 | c.1805A>T | p.Y602F | - | 7:148508727-148508727 |
| 2238 | EZH2 | ENST00000350995 | 220732 | c.1804T>C | p.Y602H | - | 7:148508728-148508728 |
| 2239 | EZH2 | NM_004456.3 | 37028 | c.1937A>T | p.Y646F | - | 7:148508727-148508727 |
| 2240 | EZH2 | NM_004456.3 | 37029 | c.1937A>C | p.Y646S | - | 7:148508727-148508727 |
| 2241 | EZH2 | NM_004456.3 | 37030 | c.1936T>C | p.Y646H | - | 7:148508728-148508728 |
| 2242 | EZH2 | NM_004456.3 | 37031 | c.1936T>A | p.Y646N | - | 7:148508728-148508728 |
| 2243 | EZH2 | NM_004456.3 | 37032 | c.1937A>G | p.Y646C | - | 7:148508727-148508727 |
| 2244 | EZH2 | NM_004456.3 | 37033 | c.1920T>A | p.N640K | - | 7:148508744-148508744 |
| 2245 | EZH2 | NM_004456.3 | 88179 | c.1907C>T | p.P636L | - | 7:148508757-148508757 |
| 2246 | ERBB4 | NM_005235 | 108015 | c.2806G>A | p.G936R | - | 2:212288940-212288940 |
| 2247 | ERBB4 | NM_005235 | 110095 | c.1022C>T | p.S341L | - | 2:212576877-212576877 |
| 2248 | ERBB4 | NM_005235 | 12833 | c.908C>A | p.S303Y | - | 2:212578349-212578349 |
| 2249 | ERBB4 | NM_005235 | 20392 | c.419C>T | p.T140I | - | 2:212812157-212812157 |
| 2250 | ERBB4 | NM_005235 | 48361 | c.2804A>T | p.K935I | - | 2:212288942-212288942 |
| 2251 | ERBB4 | NM_005235 | 48362 | c.2791G>T | p.D931Y | - | 2:212288955-212288955 |
| 2252 | ERBB4 | NM_005235 | 48363 | c.1853A>C | p.H618P | - | 2:212530066-212530066 |
| 2253 | ERBB4 | NM_005235 | 48364 | c.1784A>T | p.D595V | - | 2:212530135-212530135 |
| 2254 | ERBB4 | NM_005235 | 48365 | c.1042G>T | p.V348L | - | 2:212576857-212576857 |
| 2255 | ERBB4 | NM_005235 | 48366 | c.916C>A | p.R306S | - | 2:212578341-212578341 |
| 2256 | ERBB4 | NM_005235 | 48367 | c.854A>G | p.Y285C | - | 2:212587147-212587147 |
| 2257 | ERBB4 | NM_005235 | 48368 | c.731C>G | p.T244R | - | 2:212589811-212589811 |
| 2258 | ERBB4 | NM_005235 | 48369 | c.542A>G | p.N181S | - | 2:212652764-212652764 |
| 2259 | ERBB4 | NM_005235 |  | c.17>18 |  |  | 2:212566691-212568919 |
| 2260 | ERBB2 | NM_004448 | 12552 | c.2326_2327insTTT | p.G776>VC | + | 17:37880997-37880998 |
| 2261 | ERBB2 | NM_004448 | 12553 | c.2326_2327insTGT | p.G776>VC | + | 17:37880997-37880998 |
| 2262 | ERBB2 | NM_004448 | 13170 | c.2305G>C | p.D769H | + | 17:37880261-37880261 |
| 2263 | ERBB2 | NM_004448 | 14060 | c.2264T>C | p.L755S | + | 17:37880220-37880220 |
| 2264 | ERBB2 | NM_004448 | 14062 | c.2329G>T | p.V777L | + | 17:37881000-37881000 |
| 2265 | ERBB2 | NM_004448 | 14064 | c.2329G>A | p.V777M | + | 17:37881000-37881000 |
| 2266 | ERBB2 | NM_004448 | 14065 | c.2524G>A | p.V842I | + | 17:37881332-37881332 |
| 2267 | ERBB2 | NM_004448 | 18609 | c.2327G>T | p.G776V | + | 17:37880998-37880998 |
| 2268 | ERBB2 | NM_004448 | 21985 | c.2632C>T | p.H878Y | + | 17:37881440-37881440 |
| 2269 | ERBB2 | NM_004448 | 26681 | c.2333_2334insGGG | p.G778_S779insG | + | 17:37881004-37881005 |
| 2270 | ERBB2 | NM_004448 | 35496 | c.2330T>C | p.V777A | + | 17:37881001-37881001 |
| 2271 | ERBB2 | NM_004448 | 51317 | c.2301C>G | p.I767M | + | 17:37880257-37880257 |
| 2272 | ERBB2 | NM_004448 | 683 | c.2263_2264TT>CC | p.L755P | + | 17:37880219-37880220 |
| 2273 | ERBB2 | NM_004448 | 685 | c.2326G>A | p.G776S | + | 17:37880997-37880997 |
| 2274 | ERBB2 | NM_004448 | 681 | c.2335_2336ins9 | p.S779_P780insVGS | + | 17:37881006-37881007 |
| 2275 | ERBB2 | NM_004448 | 682 | c.2322_2323ins12 | p.M774_A775insAYVM | + | 17:37880993-37880994 |
| 2276 | ERBB2 | NM_004448 | 12556 | c.2340_2341ins9 | p.P780_Y781insGSP | + | 17:37881011-37881012 |
| 2277 | ERBB2 | NM_004448 | 12558 | c.2325_2326ins12 | p.A775_G776insYVMA | + | 17:37880996-37880997 |
| 2278 | ERBB2 | NM_004448 | 20959 | c.2324_2325ins12 | p.A775_G776insYVMA | + | 17:37880995-37880996 |
| 2279 | ERBB2 | NM_004448 |  | c.2310_2311ins12 | p.Y772_A775ins* | + | 17:37880981-37880982 |
| 2280 | EML4 | NM_019063 |  | c.2>3 |  | + | 2:42472644-42483770 |
| 2281 | EML4 | NM_019063 |  | c.6>7 |  | + | 2:42491845-42508113 |
| 2282 | EML4 | NM_019063 |  | c.13>16 |  | + | 2:42522520-42530586 |
| 2283 | EML4 | NM_019063 |  | c.18>19 |  | + | 2:42543101-42544664 |
| 2284 | EML4 | NM_019063 |  | c.20>21 |  | + | 2:42552606-42553392 |
| 2285 | EGFR | NM_005228 | 12366 | c.2572C>A | p.L858M | + | 7:55259514-55259514 |
| 2286 | EGFR | NM_005228 | 12367 | c.2237_2254del18 | p.E746_S752>A | + | 7:55242467-55242484 |
| 2287 | EGFR | NM_005228 | 12369 | c.2240_2254del15 | p.L747_T751del | + | 7:55242470-55242484 |
| 2288 | EGFR | NM_005228 | 12370 | c.2240_2257del18 | p.L747_P753>S | + | 7:55242470-55242487 |
| 2289 | EGFR | NM_005228 | 12371 | c.2126A>T | p.E709V | + | 7:55241678-55241678 |
| 2290 | EGFR | NM_005228 | 12373 | c.2159C>T | p.S720F | + | 7:55241711-55241711 |
| 2291 | EGFR | NM_005228 | 12374 | c.2582T>G | p.L861R | + | 7:55259524-55259524 |
| 2292 | EGFR | NM_005228 | 12377 | c.2319_2320insCAC | p.H773_V774insH | + | 7:55249021-55249022 |
| 2293 | EGFR | NM_005228 | 12378 | c.2310_2311insGGT | p.D770_N771insG | + | 7:55249012-55249013 |
| 2294 | EGFR | NM_005228 | 12382 | c.2239_2248TTAAGAGAAG>C | p.L747_A750>P | + | 7:55242469-55242478 |
| 2295 | EGFR | NM_005228 | 12383 | c.2239_2251>C | p.L747_T751>P | + | 7:55242469-55242481 |
| 2296 | EGFR | NM_005228 | 12384 | c.2237_2255>T | p.E746_S752>V | + | 7:55242467-55242485 |
| 2297 | EGFR | NM_005228 | 12386 | c.2237_2252>T | p.E746_T751>V | + | 7:55242467-55242482 |
| 2298 | EGFR | NM_005228 | 12427 | c.2308_2309insGTT | p.D770>GY | + | 7:55249010-55249011 |
| 2299 | EGFR | NM_005228 | 12428 | c.2125_2127GAA>CAT | p.E709H | + | 7:55241677-55241679 |
| 2300 | EGFR | NM_005228 | 12429 | c.2573_2574TG>GT | p.L858R | + | 7:55259515-55259516 |
| 2301 | EGFR | NM_005228 | 12675 | c.2575G>A | p.A859T | + | 7:55259517-55259517 |
| 2302 | EGFR | NM_005228 | 12678 | c.2237_2251del15 | p.E746_T751>A | + | 7:55242467-55242481 |
| 2303 | EGFR | NM_005228 | 12728 | c.2236_2253del18 | p.E746_T751del | + | 7:55242466-55242483 |
| 2304 | EGFR | NM_005228 | 12986 | c.2429G>A | p.G810D | + | 7:55249131-55249131 |
| 2305 | EGFR | NM_005228 | 12988 | c.2125G>A | p.E709K | + | 7:55241677-55241677 |
| 2306 | EGFR | NM_005228 | 13003 | c.2310_2311insAAC | p.D770_N771insN | + | 7:55249012-55249013 |
| 2307 | EGFR | NM_005228 | 13004 | c.2310_2311insGGC | p.D770_N771insG | + | 7:55249012-55249013 |
| 2308 | EGFR | NM_005228 | 13005 | c.2318A>T | p.H773L | + | 7:55249020-55249020 |
| 2309 | EGFR | NM_005228 | 13006 | c.2320G>A | p.V774M | + | 7:55249022-55249022 |
| 2310 | EGFR | NM_005228 | 13007 | c.2335_2336GG>TT | p.G779F | + | 7:55249037-55249038 |
| 2311 | EGFR | NM_005228 | 13008 | c.2612C>G | p.A871G | + | 7:55259554-55259554 |
| 2312 | EGFR | NM_005228 | 13009 | c.2126A>G | p.E709G | + | 7:55241678-55241678 |
| 2313 | EGFR | NM_005228 | 13180 | c.2188C>T | p.L730F | + | 7:55242418-55242418 |
| 2314 | EGFR | NM_005228 | 13181 | c.2198C>T | p.P733L | + | 7:55242428-55242428 |
| 2315 | EGFR | NM_005228 | 13182 | c.2203G>A | p.G735S | + | 7:55242433-55242433 |
| 2316 | EGFR | NM_005228 | 13183 | c.2225T>C | p.V742A | + | 7:55242455-55242455 |
| 2317 | EGFR | NM_005228 | 13184 | c.2236G>A | p.E746K | + | 7:55242466-55242466 |
| 2318 | EGFR | NM_005228 | 13185 | c.2252C>T | p.T751I | + | 7:55242482-55242482 |
| 2319 | EGFR | NM_005228 | 13186 | c.2255C>A | p.S752Y | + | 7:55242485-55242485 |
| 2320 | EGFR | NM_005228 | 13188 | c.2281G>A | p.D761N | + | 7:55242511-55242511 |
| 2321 | EGFR | NM_005228 | 13189 | c.2351C>T | p.S784F | + | 7:55249053-55249053 |
| 2322 | EGFR | NM_005228 | 13190 | c.2375T>C | p.L792P | + | 7:55249077-55249077 |
| 2323 | EGFR | NM_005228 | 13192 | c.2428G>A | p.G810S | + | 7:55249130-55249130 |
| 2324 | EGFR | NM_005228 | 13197 | c.2590G>A | p.A864T | + | 7:55259532-55259532 |
| 2325 | EGFR | NM_005228 | 13199 | c.2618G>A | p.G873E | + | 7:55259560-55259560 |
| 2326 | EGFR | NM_005228 | 133189 | c.2236_2256del21 | p.E746_S752del | + | 7:55242466-55242486 |
| 2327 | EGFR | NM_005228 | 133197 | c.2239_2257>T | p.L747_P753>S | + | 7:55242469-55242487 |
| 2328 | EGFR | NM_005228 | 133207 | c.2252_2275del24 | p.T751_I759del | + | 7:55242482-55242505 |
| 2329 | EGFR | NM_005228 | 13400 | c.2457G>A | p.V819V | + | 7:55249159-55249159 |
| 2330 | EGFR | NM_005228 | 13427 | c.2126A>C | p.E709A | + | 7:55241678-55241678 |
| 2331 | EGFR | NM_005228 | 13432 | c.2193G>A | p.W731* | + | 7:55242423-55242423 |
| 2332 | EGFR | NM_005228 | 13433 | c.2318A>G | p.H773R | + | 7:55249020-55249020 |
| 2333 | EGFR | NM_005228 | 13553 | c.2572_2573CT>AG | p.L858R | + | 7:55259514-55259515 |
| 2334 | EGFR | NM_005228 | 13556 | c.2253_2276del24 | p.S752_I759del | + | 7:55242483-55242506 |
| 2335 | EGFR | NM_005228 | 13979 | c.2170G>A | p.G724S | + | 7:55241722-55241722 |
| 2336 | EGFR | NM_005228 | 14068 | c.2308G>A | p.D770N | + | 7:55249010-55249010 |
| 2337 | EGFR | NM_005228 | 14070 | c.2588G>A | p.G863D | + | 7:55259530-55259530 |
| 2338 | EGFR | NM_005228 | 14243 | c.2234A>G | p.K745R | + | 7:55242464-55242464 |
| 2339 | EGFR | NM_005228 | 17570 | c.2222C>T | p.P741L | + | 7:55242452-55242452 |
| 2340 | EGFR | NM_005228 | 18419 | c.2200G>A | p.E734K | + | 7:55242430-55242430 |
| 2341 | EGFR | NM_005228 | 18425 | c.2156G>A | p.G719D | + | 7:55241708-55241708 |
| 2342 | EGFR | NM_005228 | 18441 | c.2154_2155GG>TT | p.G719C | + | 7:55241706-55241707 |
| 2343 | EGFR | NM_005228 | 18442 | c.2241_2244AAGA>CCCG | p.L747_R748>FP | + | 7:55242471-55242474 |
| 2344 | EGFR | NM_005228 | 21683 | c.323G>A | p.R108K | + | 7:55211080-55211080 |
| 2345 | EGFR | NM_005228 | 21685 | c.866C>A | p.A289D | + | 7:55221822-55221822 |
| 2346 | EGFR | NM_005228 | 21686 | c.865G>A | p.A289T | + | 7:55221821-55221821 |
| 2347 | EGFR | NM_005228 | 21687 | c.866C>T | p.A289V | + | 7:55221822-55221822 |
| 2348 | EGFR | NM_005228 | 21689 | c.1787C>T | p.P596L | + | 7:55233037-55233037 |
| 2349 | EGFR | NM_005228 | 21690 | c.1793G>T | p.G598V | + | 7:55233043-55233043 |
| 2350 | EGFR | NM_005228 | 21984 | c.2281G>T | p.D761Y | + | 7:55242511-55242511 |
| 2351 | EGFR | NM_005228 | 22940 | c.2327G>A | p.R776H | + | 7:55249029-55249029 |
| 2352 | EGFR | NM_005228 | 22954 | c.2324G>A | p.C775Y | + | 7:55249026-55249026 |
| 2353 | EGFR | NM_005228 | 22992 | c.2161G>A | p.G721S | + | 7:55241713-55241713 |
| 2354 | EGFR | NM_005228 | 23571 | c.2238_2252del15 | p.L747_T751del | + | 7:55242468-55242482 |
| 2355 | EGFR | NM_005228 | 24267 | c.2239_2240TT>CC | p.L747P | + | 7:55242469-55242470 |
| 2356 | EGFR | NM_005228 | 24869 | c.2235_2252del18 | p.E746_T751del | + | 7:55242465-55242482 |
| 2357 | EGFR | NM_005228 | 26038 | c.2233_2247del15 | p.K745_E749del | + | 7:55242463-55242477 |
| 2358 | EGFR | NM_005228 | 26129 | c.2572C>T | p.L858L | + | 7:55259514-55259514 |
| 2359 | EGFR | NM_005228 | 26438 | c.2620G>A | p.G874S | + | 7:55259562-55259562 |
| 2360 | EGFR | NM_005228 | 26445 | c.2300C>T | p.A767V | + | 7:55249002-55249002 |
| 2361 | EGFR | NM_005228 | 26509 | c.2227G>A | p.A743T | + | 7:55242457-55242457 |
| 2362 | EGFR | NM_005228 | 26704 | c.2240T>C | p.L747S | + | 7:55242470-55242470 |
| 2363 | EGFR | NM_005228 | 27041 | c.2213T>G | p.V738G | + | 7:55242443-55242443 |
| 2364 | EGFR | NM_005228 | 27042 | c.2282A>G | p.D761G | + | 7:55242512-55242512 |
| 2365 | EGFR | NM_005228 | 27110 | c.2356G>A | p.V786M | + | 7:55249058-55249058 |
| 2366 | EGFR | NM_005228 | 28508 | c.2104G>T | p.A702S | + | 7:55241656-55241656 |
| 2367 | EGFR | NM_005228 | 28510 | c.2162G>C | p.G721A | + | 7:55241714-55241714 |
| 2368 | EGFR | NM_005228 | 28511 | c.2108T>C | p.L703P | + | 7:55241660-55241660 |
| 2369 | EGFR | NM_005228 | 28513 | c.2350T>C | p.S784P | + | 7:55249052-55249052 |
| 2370 | EGFR | NM_005228 | 28517 | c.2235_2246del12 | p.E746_E749del | + | 7:55242465-55242476 |
| 2371 | EGFR | NM_005228 | 28601 | c.2135T>C | p.F712S | + | 7:55241687-55241687 |
| 2372 | EGFR | NM_005228 | 28603 | c.2293G>A | p.V765M | + | 7:55248995-55248995 |
| 2373 | EGFR | NM_005228 | 28605 | c.2611G>A | p.A871T | + | 7:55259553-55259553 |
| 2374 | EGFR | NM_005228 | 28607 | c.2603A>G | p.E868G | + | 7:55259545-55259545 |
| 2375 | EGFR | NM_005228 | 28610 | c.2441T>C | p.L814P | + | 7:55249143-55249143 |
| 2376 | EGFR | NM_005228 | 29274 | c.2254T>C | p.S752P | + | 7:55242484-55242484 |
| 2377 | EGFR | NM_005228 | 33725 | c.2609A>G | p.H870R | + | 7:55259551-55259551 |
| 2378 | EGFR | NM_005228 | 41603 | c.2134T>C | p.F712L | + | 7:55241686-55241686 |
| 2379 | EGFR | NM_005228 | 41663 | c.2462T>C | p.I821T | + | 7:55249164-55249164 |
| 2380 | EGFR | NM_005228 | 41905 | c.2092G>A | p.A698T | + | 7:55241644-55241644 |
| 2381 | EGFR | NM_005228 | 48922 | c.2311_2312insGCGTGGACA | p.D770_N771insSVD | + | 7:55249013-55249014 |
| 2382 | EGFR | NM_005228 | 53194 | c.2197C>T | p.P733S | + | 7:55242427-55242427 |
| 2383 | EGFR | NM_005228 | 53292 | c.2608C>T | p.H870Y | + | 7:55259550-55259550 |
| 2384 | EGFR | NM_005228 | 6210 | c.2240_2251del12 | p.L747_T751>S | + | 7:55242470-55242481 |
| 2385 | EGFR | NM_005228 | 6213 | c.2582T>A | p.L861Q | + | 7:55259524-55259524 |
| 2386 | EGFR | NM_005228 | 6218 | c.2239_2247del9 | p.L747_E749del | + | 7:55242469-55242477 |
| 2387 | EGFR | NM_005228 | 6220 | c.2238_2255del18 | p.E746_S752>D | + | 7:55242468-55242485 |
| 2388 | EGFR | NM_005228 | 6223 | c.2235_2249del15 | p.E746_A750del | + | 7:55242465-55242479 |
| 2389 | EGFR | NM_005228 | 6224 | c.2573T>G | p.L858R | + | 7:55259515-55259515 |
| 2390 | EGFR | NM_005228 | 6225 | c.2236_2250del15 | p.E746_A750del | + | 7:55242466-55242480 |
| 2391 | EGFR | NM_005228 | 6226 | c.2326C>T | p.R776C | + | 7:55249028-55249028 |
| 2392 | EGFR | NM_005228 | 6239 | c.2156G>C | p.G719A | + | 7:55241708-55241708 |
| 2393 | EGFR | NM_005228 | 6240 | c.2369C>T | p.T790M | + | 7:55249071-55249071 |
| 2394 | EGFR | NM_005228 | 6241 | c.2303G>T | p.S768I | + | 7:55249005-55249005 |
| 2395 | EGFR | NM_005228 | 6242 | c.2305G>T | p.V769L | + | 7:55249007-55249007 |
| 2396 | EGFR | NM_005228 | 6252 | c.2155G>A | p.G719S | + | 7:55241707-55241707 |
| 2397 | EGFR | NM_005228 | 6253 | c.2155G>T | p.G719C | + | 7:55241707-55241707 |
| 2398 | EGFR | NM_005228 | 6254 | c.2239_2253del15 | p.L747_T751del | + | 7:55242469-55242483 |
| 2399 | EGFR | NM_005228 | 6255 | c.2239_2256del18 | p.L747_S752del | + | 7:55242469-55242486 |
| 2400 | EGFR | NM_005228 | 6256 | c.2254_2277del24 | p.S752_I759del | + | 7:55242484-55242507 |
| 2401 | EGFR | NM_005228 | 6268 | c.2257C>T | p.P753S | + | 7:55242487-55242487 |
| 2402 | EGFR | NM_005228 | 85993 | c.2260A>G | p.K754E | + | 7:55242490-55242490 |
| 2403 | EGFR | NM_005228 | 96856 | c.2252_2276>A | p.T751_I759>N | + | 7:55242482-55242506 |
| 2404 | EGFR | NM_005228 | 6219 | c.2248G>C | p.A750P | + | 7:55242478-55242478 |
| 2405 | EGFR | NM_005228 | 12376 | c.2307_2308ins9 | p.V769_D770insASV | + | 7:55249009-55249010 |
| 2406 | EGFR | NM_005228 | 12381 | c.2319_2320ins9 | p.H773_V774insNPH | + | 7:55249021-55249022 |
| 2407 | EGFR | NM_005228 | 13428 | c.2311_2312ins9 | p.D770_N771insSVD | + | 7:55249013-55249014 |
| 2408 | EGFR | NM_005228 | 26720 | c.2290_2291ins12 | p.A763_Y764insFQEA | + | 7:55248992-55248993 |
| 2409 | EGFR | NM_005228 |  | c.25>26 |  | + | 7:55268880-55269475 |
| 2410 | EGFR | NM_005228 |  | c.17>18 |  | + | 7:55240675-55241736 |
| 2411 | CUX1 | NM_001913 |  | c.10>11 |  | + | 7:101813725-101821937 |
| 2412 | CTNNB1 | NM_001904 | 13168 | c.104T>C | p.I35T | + | 3:41266107-41266107 |
| 2413 | CTNNB1 | NM_001904 | 13175 | c.138G>A | p.L46L | + | 3:41266141-41266141 |
| 2414 | CTNNB1 | NM_001904 | 14256 | c.73_96del24 | p.WQQQSYLD25? | + | 3:41266076-41266099 |
| 2415 | CTNNB1 | NM_001904 | 17661 | c.130C>G | p.P44A | + | 3:41266133-41266133 |
| 2416 | CTNNB1 | NM_001904 | 17941 | c.67A>G | p.S23G | + | 3:41266070-41266070 |
| 2417 | CTNNB1 | NM_001904 | 22566 | c.64G>A | p.V22I | + | 3:41266067-41266067 |
| 2418 | CTNNB1 | NM_001904 | 24746 | c.112_114GGT>CCC | p.G38P | + | 3:41266115-41266117 |
| 2419 | CTNNB1 | NM_001904 | 27378 | c.107A>G | p.H36R | + | 3:41266110-41266110 |
| 2420 | CTNNB1 | NM_001904 | 29289 | c.125_126delCA | p.T42fs*7 | + | 3:41266128-41266129 |
| 2421 | CTNNB1 | NM_001904 | 34125 | c.90C>G | p.Y30* | + | 3:41266093-41266093 |
| 2422 | CTNNB1 | NM_001904 | 49161 | c.43G>A | p.E15K | + | 3:41266046-41266046 |
| 2423 | CTNNB1 | NM_001904 | 5661 | c.94G>T | p.D32Y | + | 3:41266097-41266097 |
| 2424 | CTNNB1 | NM_001904 | 5662 | c.110C>T | p.S37F | + | 3:41266113-41266113 |
| 2425 | CTNNB1 | NM_001904 | 5663 | c.133T>C | p.S45P | + | 3:41266136-41266136 |
| 2426 | CTNNB1 | NM_001904 | 5664 | c.121A>G | p.T41A | + | 3:41266124-41266124 |
| 2427 | CTNNB1 | NM_001904 | 5666 | c.110C>A | p.S37Y | + | 3:41266113-41266113 |
| 2428 | CTNNB1 | NM_001904 | 5667 | c.134C>T | p.S45F | + | 3:41266137-41266137 |
| 2429 | CTNNB1 | NM_001904 | 5668 | c.94G>C | p.D32H | + | 3:41266097-41266097 |
| 2430 | CTNNB1 | NM_001904 | 5669 | c.98C>T | p.S33F | + | 3:41266101-41266101 |
| 2431 | CTNNB1 | NM_001904 | 5670 | c.101G>T | p.G34V | + | 3:41266104-41266104 |
| 2432 | CTNNB1 | NM_001904 | 5671 | c.101G>A | p.G34E | + | 3:41266104-41266104 |
| 2433 | CTNNB1 | NM_001904 | 5672 | c.94G>A | p.D32N | + | 3:41266097-41266097 |
| 2434 | CTNNB1 | NM_001904 | 5673 | c.98C>A | p.S33Y | + | 3:41266101-41266101 |
| 2435 | CTNNB1 | NM_001904 | 5674 | c.104T>G | p.I35S | + | 3:41266107-41266107 |
| 2436 | CTNNB1 | NM_001904 | 5675 | c.109T>G | p.S37A | + | 3:41266112-41266112 |
| 2437 | CTNNB1 | NM_001904 | 5676 | c.122C>T | p.T41I | + | 3:41266125-41266125 |
| 2438 | CTNNB1 | NM_001904 | 5677 | c.98C>G | p.S33C | + | 3:41266101-41266101 |
| 2439 | CTNNB1 | NM_001904 | 5678 | c.107A>C | p.H36P | + | 3:41266110-41266110 |
| 2440 | CTNNB1 | NM_001904 | 5679 | c.110C>G | p.S37C | + | 3:41266113-41266113 |
| 2441 | CTNNB1 | NM_001904 | 5681 | c.95A>G | p.D32G | + | 3:41266098-41266098 |
| 2442 | CTNNB1 | NM_001904 | 5682 | c.97T>C | p.S33P | + | 3:41266100-41266100 |
| 2443 | CTNNB1 | NM_001904 | 5683 | c.97T>G | p.S33A | + | 3:41266100-41266100 |
| 2444 | CTNNB1 | NM_001904 | 5684 | c.100G>C | p.G34R | + | 3:41266103-41266103 |
| 2445 | CTNNB1 | NM_001904 | 5685 | c.133T>G | p.S45A | + | 3:41266136-41266136 |
| 2446 | CTNNB1 | NM_001904 | 5686 | c.100G>A | p.G34R | + | 3:41266103-41266103 |
| 2447 | CTNNB1 | NM_001904 | 5687 | c.109T>C | p.S37P | + | 3:41266112-41266112 |
| 2448 | CTNNB1 | NM_001904 | 5688 | c.121A>C | p.T41P | + | 3:41266124-41266124 |
| 2449 | CTNNB1 | NM_001904 | 5689 | c.134C>G | p.S45C | + | 3:41266137-41266137 |
| 2450 | CTNNB1 | NM_001904 | 5690 | c.95A>C | p.D32A | + | 3:41266098-41266098 |
| 2451 | CTNNB1 | NM_001904 | 5691 | c.95A>T | p.D32V | + | 3:41266098-41266098 |
| 2452 | CTNNB1 | NM_001904 | 5692 | c.134C>A | p.S45Y | + | 3:41266137-41266137 |
| 2453 | CTNNB1 | NM_001904 | 5694 | c.86C>T | p.S29F | + | 3:41266089-41266089 |
| 2454 | CTNNB1 | NM_001904 | 5696 | c.125C>T | p.T42I | + | 3:41266128-41266128 |
| 2455 | CTNNB1 | NM_001904 | 5699 | c.128C>T | p.A43V | + | 3:41266131-41266131 |
| 2456 | CTNNB1 | NM_001904 | 5701 | c.122C>G | p.T41S | + | 3:41266125-41266125 |
| 2457 | CTNNB1 | NM_001904 | 5702 | c.59C>T | p.A20V | + | 3:41266062-41266062 |
| 2458 | CTNNB1 | NM_001904 | 5703 | c.106C>T | p.H36Y | + | 3:41266109-41266109 |
| 2459 | CTNNB1 | NM_001904 | 5704 | c.130C>T | p.P44S | + | 3:41266133-41266133 |
| 2460 | CTNNB1 | NM_001904 | 5706 | c.65T>C | p.V22A | + | 3:41266068-41266068 |
| 2461 | CTNNB1 | NM_001904 | 5708 | c.119C>T | p.T40I | + | 3:41266122-41266122 |
| 2462 | CTNNB1 | NM_001904 | 5713 | c.113G>A | p.G38D | + | 3:41266116-41266116 |
| 2463 | CTNNB1 | NM_001904 | 5714 | c.67A>C | p.S23R | + | 3:41266070-41266070 |
| 2464 | CTNNB1 | NM_001904 | 5716 | c.121A>T | p.T41S | + | 3:41266124-41266124 |
| 2465 | CTNNB1 | NM_001904 | 5717 | c.123C>T | p.T41T | + | 3:41266126-41266126 |
| 2466 | CTNNB1 | NM_001904 | 5721 | c.91C>T | p.L31L | + | 3:41266094-41266094 |
| 2467 | CTNNB1 | NM_001904 | 5730 | c.122C>A | p.T41N | + | 3:41266125-41266125 |
| 2468 | CTNNB1 | NM_001904 | 5732 | c.125C>G | p.T42R | + | 3:41266128-41266128 |
| 2469 | CTNNB1 | NM_001904 | 5738 | c.61G>A | p.A21T | + | 3:41266064-41266064 |
| 2470 | CTNNB1 | NM_001904 | 5744 | c.127G>C | p.A43P | + | 3:41266130-41266130 |
| 2471 | CTNNB1 | NM_001904 | 5747 | c.37G>A | p.A13T | + | 3:41266040-41266040 |
| 2472 | CTNNB1 | NM_001904 | 5749 | c.74G>T | p.W25L | + | 3:41266077-41266077 |
| 2473 | CTNNB1 | NM_001904 | 5753 | c.116C>G | p.A39G | + | 3:41266119-41266119 |
| 2474 | CTNNB1 | NM_001904 | 5758 | c.127G>A | p.A43T | + | 3:41266130-41266130 |
| 2475 | CTNNB1 | NM_001904 | 5761 | c.131C>T | p.P44L | + | 3:41266134-41266134 |
| 2476 | CTNNB1 | NM_001904 | 5762 | c.115G>A | p.A39T | + | 3:41266118-41266118 |
| 2477 | CTNNB1 | NM_001904 | 6050 | c.64_114del51 | p.V22_G38del | + | 3:41266067-41266117 |
| 2478 | CTNNB1 | NM_001904 | 6052 | c.64_99del36 | p.V22_S33del | + | 3:41266067-41266102 |
| 2479 | CTNNB1 | NM_001904 | 6057 | c.67_99del33 | p.S23_S33del | + | 3:41266070-41266102 |
| 2480 | CTNNB1 | NM_001904 | 6064 | c.74_97del24 | p.W25_D32del | + | 3:41266077-41266100 |
| 2481 | CTNNB1 | NM_001904 | 6076 | c.88_99del12 | p.Y30_S33del | + | 3:41266091-41266102 |
| 2482 | CTNNB1 | NM_001904 | 6098 | c.97_98TC>CT | p.S33L | + | 3:41266100-41266101 |
| 2483 | CTNNB1 | NM_001904 | 6099 | c.97_98TC>AA | p.S33N | + | 3:41266100-41266101 |
| 2484 | CTNNB1 | NM_001904 | 6140 | c.120T>C | p.T40T | + | 3:41266123-41266123 |
| 2485 | CTNNB1 | NM_001904 | 17797 | c.1004A>T | p.K335I | + | 3:41268766-41268766 |
| 2486 | CSF1R | NM_005211 | 946 | c.902T>A | p.L301* | - | 5:149453044-149453044 |
| 2487 | CSF1R | NM_005211 | 947 | c.2906A>G | p.Y969C | - | 5:149433645-149433645 |
| 2488 | CSF1R | NM_005211 | 948 | c.2906A>T | p.Y969F | - | 5:149433645-149433645 |
| 2489 | CSF1R | NM_005211 | 949 | c.2907T>G | p.Y969* | - | 5:149433644-149433644 |
| 2490 | CSF1R | NM_005211 | 951 | c.2905T>A | p.Y969N | - | 5:149433646-149433646 |
| 2491 | CSF1R | NM_005211 | 952 | c.2905T>C | p.Y969H | - | 5:149433646-149433646 |
| 2492 | CSF1R | NM_005211 | 954 | c.902T>C | p.L301S | - | 5:149453044-149453044 |
| 2493 | CSF1R | NM_005211 | 955 | c.2907T>A | p.Y969* | - | 5:149433644-149433644 |
| 2494 | CIT | NM_007174 |  | c.22>23 |  | - | 12:120168299-120172162 |
| 2495 | CDKN2A | ENST00000446177 | 99191 | c.238C>T | p.R80* | - | 9:21971120-21971120 |
| 2496 | CDKN2A | ENST00000361570_v62 | 99935 | c.404C>T | p.P135L | - | 9:21971120-21971120 |
| 2497 | CDKN2A | NM_000077 | 12468 | c.236C>T | p.T79I | - | 9:21971122-21971122 |
| 2498 | CDKN2A | NM_000077 | 12469 | c.244G>A | p.V82M | - | 9:21971114-21971114 |
| 2499 | CDKN2A | NM_000077 | 12473 | c.172C>T | p.R58* | - | 9:21971186-21971186 |
| 2500 | CDKN2A | NM_000077 | 12475 | c.238C>T | p.R80* | - | 9:21971120-21971120 |
| 2501 | CDKN2A | NM_000077 | 12476 | c.341C>T | p.P114L | - | 9:21971017-21971017 |
| 2502 | CDKN2A | NM_000077 | 12479 | c.358G>T | p.E120* | - | 9:21971000-21971000 |
| 2503 | CDKN2A | NM_000077 | 12480 | c.239G>A | p.R80Q | - | 9:21971119-21971119 |
| 2504 | CDKN2A | NM_000077 | 12481 | c.329G>A | p.W110* | - | 9:21971029-21971029 |
| 2505 | CDKN2A | NM_000077 | 12484 | c.322G>A | p.D108N | - | 9:21971036-21971036 |
| 2506 | CDKN2A | NM_000077 | 12490 | c.224C>T | p.P75L | - | 9:21971134-21971134 |
| 2507 | CDKN2A | NM_000077 | 12491 | c.318G>A | p.V106V | - | 9:21971040-21971040 |
| 2508 | CDKN2A | NM_000077 | 12493 | c.303G>A | p.G101G | - | 9:21971055-21971055 |
| 2509 | CDKN2A | NM_000077 | 12494 | c.248A>C | p.H83P | - | 9:21971110-21971110 |
| 2510 | CDKN2A | NM_000077 | 12501 | c.365G>A | p.G122D | - | 9:21970993-21970993 |
| 2511 | CDKN2A | NM_000077 | 12502 | c.305C>A | p.A102E | - | 9:21971053-21971053 |
| 2512 | CDKN2A | NM_000077 | 12503 | c.334C>G | p.R112G | - | 9:21971024-21971024 |
| 2513 | CDKN2A | NM_000077 | 12504 | c.247C>T | p.H83Y | - | 9:21971111-21971111 |
| 2514 | CDKN2A | NM_000077 | 12509 | c.220G>T | p.D74Y | - | 9:21971138-21971138 |
| 2515 | CDKN2A | NM_000077 | 12511 | c.242C>A | p.P81H | - | 9:21971116-21971116 |
| 2516 | CDKN2A | NM_000077 | 12513 | c.298G>T | p.A100S | - | 9:21971060-21971060 |
| 2517 | CDKN2A | NM_000077 | 12518 | c.233_234delTC | p.L78fs*41 | - | 9:21971124-21971125 |
| 2518 | CDKN2A | NM_000077 | 12537 | c.151-1G>T | p.? | - | 9:21971208-21971208 |
| 2519 | CDKN2A | NM_000077 | 12539 | c.151-2A>C | p.? | - | 9:21971209-21971209 |
| 2520 | CDKN2A | NM_000077 | 12544 | c.243_244insT | p.P81fs*38 | - | 9:21971114-21971115 |
| 2521 | CDKN2A | NM_000077 | 12547 | c.330G>A | p.W110* | - | 9:21971028-21971028 |
| 2522 | CDKN2A | NM_000077 | 12731 | c.171_172CC>TT | p.R58* | - | 9:21971186-21971187 |
| 2523 | CDKN2A | NM_000077 | 12739 | c.222C>T | p.D74D | - | 9:21971136-21971136 |
| 2524 | CDKN2A | NM_000077 | 12741 | c.392G>A | p.R131H | - | 9:21970966-21970966 |
| 2525 | CDKN2A | NM_000077 | 12746 | c.199G>A | p.G67S | - | 9:21971159-21971159 |
| 2526 | CDKN2A | NM_000077 | 12748 | c.382C>T | p.R128W | - | 9:21970976-21970976 |
| 2527 | CDKN2A | NM_000077 | 12749 | c.204G>A | p.A68A | - | 9:21971154-21971154 |
| 2528 | CDKN2A | NM_000077 | 12758 | c.192G>A | p.L64L | - | 9:21971166-21971166 |
| 2529 | CDKN2A | NM_000077 | 13221 | c.387C>G | p.Y129* | - | 9:21970971-21970971 |
| 2530 | CDKN2A | NM_000077 | 13222 | c.151-2A>T | p.? | - | 9:21971209-21971209 |
| 2531 | CDKN2A | NM_000077 | 13223 | c.151-1G>A | p.? | - | 9:21971208-21971208 |
| 2532 | CDKN2A | NM_000077 | 13224 | c.242C>T | p.P81L | - | 9:21971116-21971116 |
| 2533 | CDKN2A | NM_000077 | 13227 | c.343G>T | p.V115L | - | 9:21971015-21971015 |
| 2534 | CDKN2A | NM_000077 | 13252 | c.170C>T | p.A57V | - | 9:21971188-21971188 |
| 2535 | CDKN2A | NM_000077 | 13254 | c.313delG | p.D105fs*41 | - | 9:21971045-21971045 |
| 2536 | CDKN2A | NM_000077 | 13274 | c.251A>G | p.D84G | - | 9:21971107-21971107 |
| 2537 | CDKN2A | NM_000077 | 13276 | c.152T>C | p.V51A | - | 9:21971206-21971206 |
| 2538 | CDKN2A | NM_000077 | 13280 | c.200G>T | p.G67V | - | 9:21971158-21971158 |
| 2539 | CDKN2A | NM_000077 | 13281 | c.205G>T | p.E69* | - | 9:21971153-21971153 |
| 2540 | CDKN2A | NM_000077 | 13289 | c.231_232delTC | p.L78fs*41 | - | 9:21971126-21971127 |
| 2541 | CDKN2A | NM_000077 | 13294 | c.376G>T | p.V126F | - | 9:21970982-21970982 |
| 2542 | CDKN2A | NM_000077 | 13295 | c.251A>T | p.D84V | - | 9:21971107-21971107 |
| 2543 | CDKN2A | NM_000077 | 13296 | c.358G>A | p.E120K | - | 9:21971000-21971000 |
| 2544 | CDKN2A | NM_000077 | 13297 | c.249_250insTT | p.D84fs*63 | - | 9:21971108-21971109 |
| 2545 | CDKN2A | NM_000077 | 13298 | c.394G>C | p.A132P | - | 9:21970964-21970964 |
| 2546 | CDKN2A | NM_000077 | 13299 | c.250G>T | p.D84Y | - | 9:21971108-21971108 |
| 2547 | CDKN2A | NM_000077 | 13300 | c.355G>T | p.E119* | - | 9:21971003-21971003 |
| 2548 | CDKN2A | NM_000077 | 13436 | c.155T>A | p.M52K | - | 9:21971203-21971203 |
| 2549 | CDKN2A | NM_000077 | 13440 | c.203C>T | p.A68V | - | 9:21971155-21971155 |
| 2550 | CDKN2A | NM_000077 | 13463 | c.369T>A | p.H123Q | - | 9:21970989-21970989 |
| 2551 | CDKN2A | NM_000077 | 13474 | c.220G>A | p.D74N | - | 9:21971138-21971138 |
| 2552 | CDKN2A | NM_000077 | 13486 | c.181G>T | p.E61* | - | 9:21971177-21971177 |
| 2553 | CDKN2A | NM_000077 | 13488 | c.250G>A | p.D84N | - | 9:21971108-21971108 |
| 2554 | CDKN2A | NM_000077 | 13489 | c.322G>T | p.D108Y | - | 9:21971036-21971036 |
| 2555 | CDKN2A | NM_000077 | 13491 | c.232_233delCT | p.L78fs*41 | - | 9:21971125-21971126 |
| 2556 | CDKN2A | NM_000077 | 13493 | c.174_189del16 | p.V59fs*82 | - | 9:21971169-21971184 |
| 2557 | CDKN2A | NM_000077 | 13494 | c.160_173del14 | p.M54fs*61 | - | 9:21971185-21971198 |
| 2558 | CDKN2A | NM_000077 | 13496 | c.151G>A | p.V51I | - | 9:21971207-21971207 |
| 2559 | CDKN2A | NM_000077 | 13504 | c.188T>C | p.L63P | - | 9:21971170-21971170 |
| 2560 | CDKN2A | NM_000077 | 13505 | c.316G>A | p.V106M | - | 9:21971042-21971042 |
| 2561 | CDKN2A | NM_000077 | 13520 | c.322G>C | p.D108H | - | 9:21971036-21971036 |
| 2562 | CDKN2A | NM_000077 | 13524 | c.237_238CC>TT | p.R80* | - | 9:21971120-21971121 |
| 2563 | CDKN2A | NM_000077 | 13531 | c.151-1G>A | p.? | - | 9:21971208-21971208 |
| 2564 | CDKN2A | NM_000077 | 13534 | c.373G>A | p.D125N | - | 9:21970985-21970985 |
| 2565 | CDKN2A | NM_000077 | 13540 | c.355G>C | p.E119Q | - | 9:21971003-21971003 |
| 2566 | CDKN2A | NM_000077 | 13546 | c.221A>T | p.D74V | - | 9:21971137-21971137 |
| 2567 | CDKN2A | NM_000077 | 13548 | c.364G>A | p.G122S | - | 9:21970994-21970994 |
| 2568 | CDKN2A | NM_000077 | 13567 | c.216C>A | p.C72* | - | 9:21971142-21971142 |
| 2569 | CDKN2A | NM_000077 | 13568 | c.225delC | p.A76fs*70 | - | 9:21971133-21971133 |
| 2570 | CDKN2A | NM_000077 | 13604 | c.202G>A | p.A68T | - | 9:21971156-21971156 |
| 2571 | CDKN2A | NM_000077 | 13607 | c.310delC | p.L104fs*42 | - | 9:21971048-21971048 |
| 2572 | CDKN2A | NM_000077 | 13608 | c.371G>A | p.R124H | - | 9:21970987-21970987 |
| 2573 | CDKN2A | NM_000077 | 13613 | c.250G>C | p.D84H | - | 9:21971108-21971108 |
| 2574 | CDKN2A | NM_000077 | 13619 | c.363delG | p.G122fs*24 | - | 9:21970995-21970995 |
| 2575 | CDKN2A | NM_000077 | 13633 | c.386A>G | p.Y129C | - | 9:21970972-21970972 |
| 2576 | CDKN2A | NM_000077 | 13642 | c.228_229insT | p.T77fs*43 | - | 9:21971129-21971130 |
| 2577 | CDKN2A | NM_000077 | 13645 | c.366C>A | p.G122G | - | 9:21970992-21970992 |
| 2578 | CDKN2A | NM_000077 | 13675 | c.305C>T | p.A102V | - | 9:21971053-21971053 |
| 2579 | CDKN2A | NM_000077 | 13713 | c.340C>T | p.P114S | - | 9:21971018-21971018 |
| 2580 | CDKN2A | NM_000077 | 13717 | c.206A>T | p.E69V | - | 9:21971152-21971152 |
| 2581 | CDKN2A | NM_000077 | 13754 | c.157_174del18 | p.M53_R58del | - | 9:21971184-21971201 |
| 2582 | CDKN2A | NM_000077 | 13766 | c.227C>T | p.A76V | - | 9:21971131-21971131 |
| 2583 | CDKN2A | NM_000077 | 13779 | c.160_161insA | p.M54fs*66 | - | 9:21971197-21971198 |
| 2584 | CDKN2A | NM_000077 | 13782 | c.187C>G | p.L63V | - | 9:21971171-21971171 |
| 2585 | CDKN2A | NM_000077 | 13803 | c.230_231delCT | p.L78fs*41 | - | 9:21971127-21971128 |
| 2586 | CDKN2A | NM_000077 | 13807 | c.346G>T | p.D116Y | - | 9:21971012-21971012 |
| 2587 | CDKN2A | NM_000077 | 13815 | c.209C>T | p.P70L | - | 9:21971149-21971149 |
| 2588 | CDKN2A | NM_000077 | 13827 | c.378C>T | p.V126V | - | 9:21970980-21970980 |
| 2589 | CDKN2A | NM_000077 | 13830 | c.341C>A | p.P114H | - | 9:21971017-21971017 |
| 2590 | CDKN2A | NM_000077 | 13965 | c.156G>C | p.M52I | - | 9:21971202-21971202 |
| 2591 | CDKN2A | NM_000077 | 13972 | c.194T>C | p.L65P | - | 9:21971164-21971164 |
| 2592 | CDKN2A | NM_000077 | 14253 | c.197A>G | p.H66R | - | 9:21971161-21971161 |
| 2593 | CDKN2A | NM_000077 | 14254 | c.151-4G>C | p.? | - | 9:21971211-21971211 |
| 2594 | CDKN2A | NM_000077 | 22559 | c.169G>C | p.A57P | - | 9:21971189-21971189 |
| 2595 | CDKN2A | NM_000077 | 22560 | c.179C>T | p.A60V | - | 9:21971179-21971179 |
| 2596 | CDKN2A | NM_000077 | 22561 | c.213C>A | p.N71K | - | 9:21971145-21971145 |
| 2597 | CDKN2A | NM_000077 | 28562 | c.387C>A | p.Y129* | - | 9:21970971-21970971 |
| 2598 | CDKN2A | NM_000077 | 28675 | c.389T>C | p.L130P | - | 9:21970969-21970969 |
| 2599 | CDKN2A | NM_000077 | 33797 | c.370delC | p.R124fs*22 | - | 9:21970988-21970988 |
| 2600 | CDKN2A | NM_000077 | 33799 | c.389T>G | p.L130R | - | 9:21970969-21970969 |
| 2601 | CDKN2A | NM_000077 | 33800 | c.390G>A | p.L130L | - | 9:21970968-21970968 |
| 2602 | CDKN2A | NM_000077 | 33802 | c.396G>A | p.A132A | - | 9:21970962-21970962 |
| 2603 | CDH1 | NM_004360.2 | 19747 | c.1057G>T | p.E353* | + | 16:68846086-68846086 |
| 2604 | CDH1 | NM_004360.2 | 19748 | c.1108G>C | p.D370H | + | 16:68846137-68846137 |
| 2605 | CDH1 | NM_004360.2 | 19750 | c.1204G>A | p.D402N | + | 16:68847282-68847282 |
| 2606 | CDH1 | NM_004360.2 | 19751 | c.1027delC | p.L343fs*13 | + | 16:68846056-68846056 |
| 2607 | CDH1 | NM_004360.2 | 19753 | c.1009-1G>A | p.? | + | 16:68846037-68846037 |
| 2608 | CDH1 | NM_004360.2 | 19761 | c.1196_1199delCTGA | p.T399fs*17 | + | 16:68847274-68847277 |
| 2609 | CDH1 | NM_004360.2 | 28934 | c.240_241insGGTG | p.V82fs*13 | + | 16:68835649-68835650 |
| 2610 | CD74 | NM_004355 |  | c.6>7 |  | - | 5:149786443-149786887 |
| 2611 | CCDC6 | NM_005436 |  | c.1>2 |  | - | 10:61548505-61554355 |
| 2612 | CCDC6 | NM_005436 |  | c.6>7 |  | - | 10:61574409-61592282 |
| 2613 | BRAF | NM_004333 | 1111 | c.1390G>C | p.G464R | - | 7:140481418-140481418 |
| 2614 | BRAF | NM_004333 | 1112 | c.1396G>C | p.G466R | - | 7:140481412-140481412 |
| 2615 | BRAF | NM_004333 | 1113 | c.1405_1407GGA>AGC | p.G469S | - | 7:140481401-140481403 |
| 2616 | BRAF | NM_004333 | 1115 | c.1746A>G | p.I582M | - | 7:140453189-140453189 |
| 2617 | BRAF | NM_004333 | 1116 | c.1749T>C | p.F583F | - | 7:140453186-140453186 |
| 2618 | BRAF | NM_004333 | 1117 | c.1752T>C | p.L584L | - | 7:140453183-140453183 |
| 2619 | BRAF | NM_004333 | 1118 | c.1758A>G | p.E586E | - | 7:140453177-140453177 |
| 2620 | BRAF | NM_004333 | 1119 | c.1776A>G | p.I592M | - | 7:140453159-140453159 |
| 2621 | BRAF | NM_004333 | 1120 | c.1774A>G | p.I592V | - | 7:140453161-140453161 |
| 2622 | BRAF | NM_004333 | 1121 | c.1782T>A | p.D594E | - | 7:140453153-140453153 |
| 2623 | BRAF | NM_004333 | 1123 | c.1784T>C | p.F595S | - | 7:140453151-140453151 |
| 2624 | BRAF | NM_004333 | 1124 | c.1791A>G | p.L597L | - | 7:140453144-140453144 |
| 2625 | BRAF | NM_004333 | 1125 | c.1790T>A | p.L597Q | - | 7:140453145-140453145 |
| 2626 | BRAF | NM_004333 | 1126 | c.1789_1790CT>TC | p.L597S | - | 7:140453145-140453146 |
| 2627 | BRAF | NM_004333 | 1127 | c.1797_1799AGT>GAG | p.V600R | - | 7:140453136-140453138 |
| 2628 | BRAF | NM_004333 | 1128 | c.1797_1797A>TACTACG | p.T599_V600insTT | - | 7:140453138-140453138 |
| 2629 | BRAF | NM_004333 | 1130 | c.1798G>A | p.V600M | - | 7:140453137-140453137 |
| 2630 | BRAF | NM_004333 | 1132 | c.1803A>C | p.K601N | - | 7:140453132-140453132 |
| 2631 | BRAF | NM_004333 | 1133 | c.1799_1801delTGA | p.V600_K601>E | - | 7:140453134-140453136 |
| 2632 | BRAF | NM_004333 | 1134 | c.1810T>G | p.W604G | - | 7:140453125-140453125 |
| 2633 | BRAF | NM_004333 | 1135 | c.1813_1814AG>TT | p.S605F | - | 7:140453121-140453122 |
| 2634 | BRAF | NM_004333 | 1136 | c.1814G>A | p.S605N | - | 7:140453121-140453121 |
| 2635 | BRAF | NM_004333 | 1137 | c.1817G>A | p.G606E | - | 7:140453118-140453118 |
| 2636 | BRAF | NM_004333 | 1138 | c.1823A>G | p.H608R | - | 7:140453112-140453112 |
| 2637 | BRAF | NM_004333 | 144982 | c.1797_1798insACA | p.T599_V600insT | - | 7:140453137-140453138 |
| 2638 | BRAF | NM_004333 | 18443 | c.1799T>C | p.V600A | - | 7:140453136-140453136 |
| 2639 | BRAF | NM_004333 | 21492 | c.1357C>A | p.P453T | - | 7:140481451-140481451 |
| 2640 | BRAF | NM_004333 | 21542 | c.1813A>G | p.S605G | - | 7:140453122-140453122 |
| 2641 | BRAF | NM_004333 | 21549 | c.1793C>T | p.A598V | - | 7:140453142-140453142 |
| 2642 | BRAF | NM_004333 | 21609 | c.1761C>A | p.D587E | - | 7:140453174-140453174 |
| 2643 | BRAF | NM_004333 | 21612 | c.1783T>C | p.F595L | - | 7:140453152-140453152 |
| 2644 | BRAF | NM_004333 | 219798 | c.1798G>C | p.V600L | - | 7:140453137-140453137 |
| 2645 | BRAF | NM_004333 | 24642 | c.1411G>T | p.V471F | - | 7:140481397-140481397 |
| 2646 | BRAF | NM_004333 | 249889 | c.1798_1799GT>CA | p.V600Q | - | 7:140453136-140453137 |
| 2647 | BRAF | NM_004333 | 26506 | c.1787G>A | p.G596D | - | 7:140453148-140453148 |
| 2648 | BRAF | NM_004333 | 26625 | c.1794_1795insGTT | p.A598_T599insV | - | 7:140453140-140453141 |
| 2649 | BRAF | NM_004333 | 27639 | c.1780G>A | p.D594N | - | 7:140453155-140453155 |
| 2650 | BRAF | NM_004333 | 27912 | c.1405_1407GGA>AGT | p.G469S | - | 7:140481401-140481403 |
| 2651 | BRAF | NM_004333 | 28010 | c.1750C>T | p.L584F | - | 7:140453185-140453185 |
| 2652 | BRAF | NM_004333 | 30594 | c.1801_1803delAAA | p.K601del | - | 7:140453132-140453134 |
| 2653 | BRAF | NM_004333 | 30730 | c.1796_1797insTAC | p.T599_V600insT | - | 7:140453138-140453139 |
| 2654 | BRAF | NM_004333 | 33729 | c.1807C>T | p.R603* | - | 7:140453128-140453128 |
| 2655 | BRAF | NM_004333 | 33808 | c.1798G>T | p.V600L | - | 7:140453137-140453137 |
| 2656 | BRAF | NM_004333 | 447 | c.1385G>T | p.R462I | - | 7:140481423-140481423 |
| 2657 | BRAF | NM_004333 | 449 | c.1391G>A | p.G464E | - | 7:140481417-140481417 |
| 2658 | BRAF | NM_004333 | 450 | c.1391G>T | p.G464V | - | 7:140481417-140481417 |
| 2659 | BRAF | NM_004333 | 451 | c.1397G>T | p.G466V | - | 7:140481411-140481411 |
| 2660 | BRAF | NM_004333 | 452 | c.1397G>C | p.G466A | - | 7:140481411-140481411 |
| 2661 | BRAF | NM_004333 | 453 | c.1397G>A | p.G466E | - | 7:140481411-140481411 |
| 2662 | BRAF | NM_004333 | 455 | c.1405G>C | p.G469R | - | 7:140481403-140481403 |
| 2663 | BRAF | NM_004333 | 457 | c.1405G>A | p.G469R | - | 7:140481403-140481403 |
| 2664 | BRAF | NM_004333 | 458 | c.1405_1406GG>TC | p.G469S | - | 7:140481402-140481403 |
| 2665 | BRAF | NM_004333 | 459 | c.1406G>T | p.G469V | - | 7:140481402-140481402 |
| 2666 | BRAF | NM_004333 | 460 | c.1406G>C | p.G469A | - | 7:140481402-140481402 |
| 2667 | BRAF | NM_004333 | 461 | c.1406G>A | p.G469E | - | 7:140481402-140481402 |
| 2668 | BRAF | NM_004333 | 462 | c.1742A>G | p.N581S | - | 7:140453193-140453193 |
| 2669 | BRAF | NM_004333 | 463 | c.1756G>A | p.E586K | - | 7:140453179-140453179 |
| 2670 | BRAF | NM_004333 | 464 | c.1760A>C | p.D587A | - | 7:140453175-140453175 |
| 2671 | BRAF | NM_004333 | 465 | c.1761C>G | p.D587E | - | 7:140453174-140453174 |
| 2672 | BRAF | NM_004333 | 466 | c.1781A>T | p.D594V | - | 7:140453154-140453154 |
| 2673 | BRAF | NM_004333 | 467 | c.1781A>G | p.D594G | - | 7:140453154-140453154 |
| 2674 | BRAF | NM_004333 | 468 | c.1785T>G | p.F595L | - | 7:140453150-140453150 |
| 2675 | BRAF | NM_004333 | 469 | c.1786G>C | p.G596R | - | 7:140453149-140453149 |
| 2676 | BRAF | NM_004333 | 470 | c.1789C>G | p.L597V | - | 7:140453146-140453146 |
| 2677 | BRAF | NM_004333 | 471 | c.1790T>G | p.L597R | - | 7:140453145-140453145 |
| 2678 | BRAF | NM_004333 | 472 | c.1796C>T | p.T599I | - | 7:140453139-140453139 |
| 2679 | BRAF | NM_004333 | 473 | c.1798_1799GT>AA | p.V600K | - | 7:140453136-140453137 |
| 2680 | BRAF | NM_004333 | 474 | c.1798_1799GT>AG | p.V600R | - | 7:140453136-140453137 |
| 2681 | BRAF | NM_004333 | 475 | c.1799_1800TG>AA | p.V600E | - | 7:140453135-140453136 |
| 2682 | BRAF | NM_004333 | 476 | c.1799T>A | p.V600E | - | 7:140453136-140453136 |
| 2683 | BRAF | NM_004333 | 477 | c.1799_1800TG>AT | p.V600D | - | 7:140453135-140453136 |
| 2684 | BRAF | NM_004333 | 478 | c.1801A>G | p.K601E | - | 7:140453134-140453134 |
| 2685 | BRAF | NM_004333 | 53198 | c.1785T>A | p.F595L | - | 7:140453150-140453150 |
| 2686 | BRAF | NM_004333 | 6137 | c.1799T>G | p.V600G | - | 7:140453136-140453136 |
| 2687 | BRAF | NM_004333 | 6262 | c.1330C>T | p.R444W | - | 7:140481478-140481478 |
| 2688 | BRAF | NM_004333 | 6265 | c.1803A>T | p.K601N | - | 7:140453132-140453132 |
| 2689 | BRAF | NM_004333 | 6267 | c.1808_1810delGAT | p.W604del | - | 7:140453125-140453127 |
| 2690 | BRAF | NM_004333 |  | c.1405_1406GG>TT | p.G469L | - | 7:140481402-140481403 |
| 2691 | BRAF | NM_004333 | 1133046 | c.1415A>G | p.Y472C | - | 7:140481393-140481393 |
| 2692 | BAG4 | NM_004874 |  | c.2>3 |  | + | 8:38050205-38065284 |
| 2693 | ATM | NM_000051 | 12791 | c.7996A>G | p.T2666A | + | 11:108204681-108204681 |
| 2694 | ATM | NM_000051 | 12792 | c.5380C>T | p.L1794L | + | 11:108173640-108173640 |
| 2695 | ATM | NM_000051 | 12793 | c.2542G>C | p.E848Q | + | 11:108137973-108137973 |
| 2696 | ATM | NM_000051 | 12951 | c.7325A>C | p.Q2442P | + | 11:108200958-108200958 |
| 2697 | ATM | NM_000051 | 20404 | c.7328G>A | p.R2443Q | + | 11:108200961-108200961 |
| 2698 | ATM | NM_000051 | 21323 | c.1009C>T | p.R337C | + | 11:108117798-108117798 |
| 2699 | ATM | NM_000051 | 21624 | c.9139C>T | p.R3047* | + | 11:108236203-108236203 |
| 2700 | ATM | NM_000051 | 21626 | c.9023G>A | p.R3008H | + | 11:108236087-108236087 |
| 2701 | ATM | NM_000051 | 21636 | c.8084G>C | p.G2695A | + | 11:108205769-108205769 |
| 2702 | ATM | NM_000051 | 21642 | c.9022C>T | p.R3008C | + | 11:108236086-108236086 |
| 2703 | ATM | NM_000051 | 21679 | c.8663T>C | p.I2888T | + | 11:108218084-108218084 |
| 2704 | ATM | NM_000051 | 21825 | c.1229T>C | p.V410A | + | 11:108119823-108119823 |
| 2705 | ATM | NM_000051 | 21826 | c.2572T>C | p.F858L | + | 11:108138003-108138003 |
| 2706 | ATM | NM_000051 | 21918 | c.5224G>C | p.A1742P | + | 11:108172421-108172421 |
| 2707 | ATM | NM_000051 | 21919 | c.5041A>G | p.I1681V | + | 11:108170476-108170476 |
| 2708 | ATM | NM_000051 | 21920 | c.5044G>T | p.D1682Y | + | 11:108170479-108170479 |
| 2709 | ATM | NM_000051 | 21922 | c.5821G>C | p.V1941L | + | 11:108180945-108180945 |
| 2710 | ATM | NM_000051 | 21924 | c.1058_1059delGT | p.C353fs*5 | + | 11:108117847-108117848 |
| 2711 | ATM | NM_000051 | 21930 | c.8839A>T | p.T2947S | + | 11:108225590-108225590 |
| 2712 | ATM | NM_000051 | 21931 | c.1009C>A | p.R337S | + | 11:108117798-108117798 |
| 2713 | ATM | NM_000051 | 22481 | c.8174A>T | p.D2725V | + | 11:108206594-108206594 |
| 2714 | ATM | NM_000051 | 22485 | c.8668C>G | p.L2890V | + | 11:108218089-108218089 |
| 2715 | ATM | NM_000051 | 22499 | c.1810C>T | p.P604S | + | 11:108123551-108123551 |
| 2716 | ATM | NM_000051 | 22507 | c.3925G>A | p.A1309T | + | 11:108155132-108155132 |
| 2717 | APC | NM_000038 | 13113 | c.3927_3931delAAAGA | p.E1309fs*4 | + | 5:112175218-112175222 |
| 2718 | APC | NM_000038 | 13121 | c.4099C>T | p.Q1367* | + | 5:112175390-112175390 |
| 2719 | APC | NM_000038 | 13123 | c.4729G>T | p.E1577* | + | 5:112176020-112176020 |
| 2720 | APC | NM_000038 | 13125 | c.3340C>T | p.R1114* | + | 5:112174631-112174631 |
| 2721 | APC | NM_000038 | 13127 | c.4348C>T | p.R1450* | + | 5:112175639-112175639 |
| 2722 | APC | NM_000038 | 13727 | c.3922A>T | p.K1308* | + | 5:112175213-112175213 |
| 2723 | APC | NM_000038 | 13728 | c.3907C>T | p.Q1303* | + | 5:112175198-112175198 |
| 2724 | APC | NM_000038 | 13864 | c.4393_4394delAG | p.S1465fs*3 | + | 5:112175684-112175685 |
| 2725 | APC | NM_000038 | 13872 | c.3286C>T | p.Q1096* | + | 5:112174577-112174577 |
| 2726 | APC | NM_000038 | 13879 | c.4639G>T | p.E1547* | + | 5:112175930-112175930 |
| 2727 | APC | NM_000038 | 181848 | c.4127_4128delAT | p.Y1376fs*9 | + | 5:112175418-112175419 |
| 2728 | APC | NM_000038 | 18561 | c.4666_4667insA | p.T1556fs*3 | + | 5:112175957-112175958 |
| 2729 | APC | NM_000038 | 18576 | c.4666delA | p.T1556fs*9 | + | 5:112175957-112175957 |
| 2730 | APC | NM_000038 | 18698 | c.4126_4127delTA | p.Y1376fs*9 | + | 5:112175417-112175418 |
| 2731 | APC | NM_000038 | 18699 | c.4461_4462insT | p.L1488fs*26 | + | 5:112175752-112175753 |
| 2732 | APC | NM_000038 | 18700 | c.3956delC | p.P1319fs*2 | + | 5:112175247-112175247 |
| 2733 | APC | NM_000038 | 18701 | c.3926_3930delAAAAG | p.E1309fs*4 | + | 5:112175217-112175221 |
| 2734 | APC | NM_000038 | 18702 | c.3964G>T | p.E1322* | + | 5:112175255-112175255 |
| 2735 | APC | NM_000038 | 18704 | c.4312delA | p.T1438fs*35 | + | 5:112175603-112175603 |
| 2736 | APC | NM_000038 | 18719 | c.3923_3924insA | p.E1309fs*6 | + | 5:112175214-112175215 |
| 2737 | APC | NM_000038 | 18729 | c.4405C>T | p.Q1469* | + | 5:112175696-112175696 |
| 2738 | APC | NM_000038 | 18734 | c.4662_4663insA | p.T1556fs*3 | + | 5:112175953-112175954 |
| 2739 | APC | NM_000038 | 18735 | c.3916G>A | p.E1306K | + | 5:112175207-112175207 |
| 2740 | APC | NM_000038 | 18737 | c.4120G>A | p.E1374K | + | 5:112175411-112175411 |
| 2741 | APC | NM_000038 | 18738 | c.4495G>A | p.G1499R | + | 5:112175786-112175786 |
| 2742 | APC | NM_000038 | 18755 | c.3928_3929delAA | p.K1310fs*4 | + | 5:112175219-112175220 |
| 2743 | APC | NM_000038 | 18758 | c.4108A>T | p.K1370* | + | 5:112175399-112175399 |
| 2744 | APC | NM_000038 | 18759 | c.4033G>T | p.E1345* | + | 5:112175324-112175324 |
| 2745 | APC | NM_000038 | 18760 | c.3916G>T | p.E1306* | + | 5:112175207-112175207 |
| 2746 | APC | NM_000038 | 18763 | c.4065_4066delTT | p.S1356fs*18 | + | 5:112175356-112175357 |
| 2747 | APC | NM_000038 | 18764 | c.3921_3925delAAAAG | p.E1309fs*4 | + | 5:112175212-112175216 |
| 2748 | APC | NM_000038 | 18765 | c.4060_4064delTTTTC | p.S1355fs*18 | + | 5:112175351-112175355 |
| 2749 | APC | NM_000038 | 18767 | c.3920_3921delTA | p.I1307fs*7 | + | 5:112175211-112175212 |
| 2750 | APC | NM_000038 | 18775 | c.3925G>T | p.E1309* | + | 5:112175216-112175216 |
| 2751 | APC | NM_000038 | 18777 | c.3944C>A | p.S1315* | + | 5:112175235-112175235 |
| 2752 | APC | NM_000038 | 18779 | c.4067C>G | p.S1356* | + | 5:112175358-112175358 |
| 2753 | APC | NM_000038 | 18783 | c.4316delC | p.P1439fs*34 | + | 5:112175607-112175607 |
| 2754 | APC | NM_000038 | 18785 | c.4469delA | p.H1490fs*17 | + | 5:112175760-112175760 |
| 2755 | APC | NM_000038 | 18786 | c.4476delC | p.T1493fs*14 | + | 5:112175767-112175767 |
| 2756 | APC | NM_000038 | 18796 | c.3935delG | p.G1312fs*9 | + | 5:112175226-112175226 |
| 2757 | APC | NM_000038 | 18804 | c.3943_3944insA | p.S1315fs*3 | + | 5:112175234-112175235 |
| 2758 | APC | NM_000038 | 18809 | c.3920delT | p.I1307fs*14 | + | 5:112175211-112175211 |
| 2759 | APC | NM_000038 | 18817 | c.3934G>T | p.G1312* | + | 5:112175225-112175225 |
| 2760 | APC | NM_000038 | 18823 | c.4328delC | p.P1443fs*30 | + | 5:112175619-112175619 |
| 2761 | APC | NM_000038 | 18825 | c.4364delA | p.N1455fs*18 | + | 5:112175655-112175655 |
| 2762 | APC | NM_000038 | 18834 | c.4135G>T | p.E1379* | + | 5:112175426-112175426 |
| 2763 | APC | NM_000038 | 18836 | c.4285C>T | p.Q1429* | + | 5:112175576-112175576 |
| 2764 | APC | NM_000038 | 18838 | c.4391_4394delAGAG | p.E1464fs*8 | + | 5:112175682-112175685 |
| 2765 | APC | NM_000038 | 18852 | c.2626C>T | p.R876* | + | 5:112173917-112173917 |
| 2766 | APC | NM_000038 | 18855 | c.3898_3908del11 | p.T1301fs*10 | + | 5:112175189-112175199 |
| 2767 | APC | NM_000038 | 18856 | c.3930_3933delGATT | p.I1311fs*9 | + | 5:112175221-112175224 |
| 2768 | APC | NM_000038 | 18861 | c.4128T>A | p.Y1376* | + | 5:112175419-112175419 |
| 2769 | APC | NM_000038 | 18862 | c.4132C>T | p.Q1378* | + | 5:112175423-112175423 |
| 2770 | APC | NM_000038 | 18866 | c.4668_4669insA | p.I1557fs*2 | + | 5:112175959-112175960 |
| 2771 | APC | NM_000038 | 18869 | c.4308delT | p.S1436fs*37 | + | 5:112175599-112175599 |
| 2772 | APC | NM_000038 | 18873 | c.4385_4386delAG | p.S1465fs*3 | + | 5:112175676-112175677 |
| 2773 | APC | NM_000038 | 18883 | c.4390G>T | p.E1464* | + | 5:112175681-112175681 |
| 2774 | APC | NM_000038 | 18885 | c.4495G>T | p.G1499* | + | 5:112175786-112175786 |
| 2775 | APC | NM_000038 | 18910 | c.4126_4127insT | p.Y1376fs*10 | + | 5:112175417-112175418 |
| 2776 | APC | NM_000038 | 18927 | c.4329T>A | p.P1443P | + | 5:112175620-112175620 |
| 2777 | APC | NM_000038 | 18931 | c.4392_4393delGA | p.S1465fs*3 | + | 5:112175683-112175684 |
| 2778 | APC | NM_000038 | 18935 | c.3870_3871insCAGACGA | p.Q1294fs*9 | + | 5:112175161-112175162 |
| 2779 | APC | NM_000038 | 18937 | c.3904_3905insT | p.Q1303fs*12 | + | 5:112175195-112175196 |
| 2780 | APC | NM_000038 | 18938 | c.4040delC | p.R1348fs*67 | + | 5:112175331-112175331 |
| 2781 | APC | NM_000038 | 18942 | c.3928A>T | p.K1310* | + | 5:112175219-112175219 |
| 2782 | APC | NM_000038 | 18949 | c.3942delG | p.R1314fs*7 | + | 5:112175233-112175233 |
| 2783 | APC | NM_000038 | 18950 | c.3920_3924delTAAAA | p.I1307fs*6 | + | 5:112175211-112175215 |
| 2784 | APC | NM_000038 | 18960 | c.3880C>T | p.Q1294* | + | 5:112175171-112175171 |
| 2785 | APC | NM_000038 | 18961 | c.3883G>T | p.E1295* | + | 5:112175174-112175174 |
| 2786 | APC | NM_000038 | 18965 | c.4477_4478insA | p.T1493fs*21 | + | 5:112175768-112175769 |
| 2787 | APC | NM_000038 | 18978 | c.4667_4668insC | p.I1557fs*2 | + | 5:112175958-112175959 |
| 2788 | APC | NM_000038 | 18986 | c.4066_4079del14 | p.G1357fs*13 | + | 5:112175357-112175370 |
| 2789 | APC | NM_000038 | 18990 | c.4122_4123delAC | p.H1375fs*10 | + | 5:112175413-112175414 |
| 2790 | APC | NM_000038 | 18997 | c.4372delC | p.P1458fs*15 | + | 5:112175663-112175663 |
| 2791 | APC | NM_000038 | 18999 | c.4461delT | p.L1488fs*19 | + | 5:112175752-112175752 |
| 2792 | APC | NM_000038 | 19005 | c.3893_3894insTGCTAATA | p.T1301fs*7 | + | 5:112175184-112175185 |
| 2793 | APC | NM_000038 | 19020 | c.4665_4666insA | p.T1556fs*3 | + | 5:112175956-112175957 |
| 2794 | APC | NM_000038 | 19021 | c.4330C>T | p.Q1444* | + | 5:112175621-112175621 |
| 2795 | APC | NM_000038 | 19033 | c.4110_4111delAA | p.P1372fs*2 | + | 5:112175401-112175402 |
| 2796 | APC | NM_000038 | 19047 | c.4050delA | p.A1351fs*64 | + | 5:112175341-112175341 |
| 2797 | APC | NM_000038 | 19048 | c.4057G>T | p.E1353* | + | 5:112175348-112175348 |
| 2798 | APC | NM_000038 | 19049 | c.4326T>A | p.P1442P | + | 5:112175617-112175617 |
| 2799 | APC | NM_000038 | 19052 | c.4290delC | p.M1431fs*42 | + | 5:112175581-112175581 |
| 2800 | APC | NM_000038 | 19053 | c.4314_4315delAC | p.P1439fs*15 | + | 5:112175605-112175606 |
| 2801 | APC | NM_000038 | 19054 | c.4473delT | p.F1491fs*16 | + | 5:112175764-112175764 |
| 2802 | APC | NM_000038 | 19055 | c.4537G>T | p.E1513* | + | 5:112175828-112175828 |
| 2803 | APC | NM_000038 | 19062 | c.2606delA | p.N869fs*47 | + | 5:112173897-112173897 |
| 2804 | APC | NM_000038 | 19072 | c.3871C>T | p.Q1291* | + | 5:112175162-112175162 |
| 2805 | APC | NM_000038 | 19084 | c.4037C>A | p.S1346* | + | 5:112175328-112175328 |
| 2806 | APC | NM_000038 | 19085 | c.4120G>T | p.E1374* | + | 5:112175411-112175411 |
| 2807 | APC | NM_000038 | 19093 | c.4304delG | p.R1435fs*38 | + | 5:112175595-112175595 |
| 2808 | APC | NM_000038 | 19094 | c.4332_4335delAACA | p.T1445fs*27 | + | 5:112175623-112175626 |
| 2809 | APC | NM_000038 | 19095 | c.4351G>T | p.E1451* | + | 5:112175642-112175642 |
| 2810 | APC | NM_000038 | 19098 | c.4463delT | p.L1488fs*19 | + | 5:112175754-112175754 |
| 2811 | APC | NM_000038 | 19099 | c.3949G>C | p.E1317Q | + | 5:112175240-112175240 |
| 2812 | APC | NM_000038 | 19105 | c.4466delT | p.L1489fs*18 | + | 5:112175757-112175757 |
| 2813 | APC | NM_000038 | 19119 | c.4364_4365insA | p.N1455fs*2 | + | 5:112175655-112175656 |
| 2814 | APC | NM_000038 | 19127 | c.4394_4395delGT | p.S1465fs*3 | + | 5:112175685-112175686 |
| 2815 | APC | NM_000038 | 19129 | c.4339C>T | p.Q1447* | + | 5:112175630-112175630 |
| 2816 | APC | NM_000038 | 19141 | c.4393delA | p.S1465fs*8 | + | 5:112175684-112175684 |
| 2817 | APC | NM_000038 | 19145 | c.3925_3929delGAAAA | p.E1309fs*4 | + | 5:112175216-112175220 |
| 2818 | APC | NM_000038 | 19148 | c.4358delC | p.P1453fs*20 | + | 5:112175649-112175649 |
| 2819 | APC | NM_000038 | 19201 | c.4384_4385delAA | p.K1462fs*6 | + | 5:112175675-112175676 |
| 2820 | APC | NM_000038 | 19203 | c.3919_3920insA | p.I1307fs*8 | + | 5:112175210-112175211 |
| 2821 | APC | NM_000038 | 19218 | c.3902C>G | p.T1301S | + | 5:112175193-112175193 |
| 2822 | APC | NM_000038 | 19225 | c.4468_4469delCA | p.H1490fs*23 | + | 5:112175759-112175760 |
| 2823 | APC | NM_000038 | 19230 | c.2639T>C | p.I880T | + | 5:112173930-112173930 |
| 2824 | APC | NM_000038 | 19236 | c.4333delA | p.T1445fs*28 | + | 5:112175624-112175624 |
| 2825 | APC | NM_000038 | 19241 | c.4067C>A | p.S1356* | + | 5:112175358-112175358 |
| 2826 | APC | NM_000038 | 19253 | c.3949G>T | p.E1317* | + | 5:112175240-112175240 |
| 2827 | APC | NM_000038 | 19263 | c.3922_3926delAAAGA | p.E1309fs*4 | + | 5:112175213-112175217 |
| 2828 | APC | NM_000038 | 19268 | c.4132_4133delCA | p.Q1378fs*7 | + | 5:112175423-112175424 |
| 2829 | APC | NM_000038 | 19299 | c.4473_4474insT | p.A1492fs*22 | + | 5:112175764-112175765 |
| 2830 | APC | NM_000038 | 19329 | c.3359G>A | p.G1120E | + | 5:112174650-112174650 |
| 2831 | APC | NM_000038 | 19330 | c.2656C>T | p.Q886* | + | 5:112173947-112173947 |
| 2832 | APC | NM_000038 | 19332 | c.4391_4392delAG | p.S1465fs*3 | + | 5:112175682-112175683 |
| 2833 | APC | NM_000038 | 19340 | c.4474delG | p.A1492fs*15 | + | 5:112175765-112175765 |
| 2834 | APC | NM_000038 | 19349 | c.4479_4480delGG | p.E1494fs*19 | + | 5:112175770-112175771 |
| 2835 | APC | NM_000038 | 19478 | c.4691T>G | p.L1564* | + | 5:112175982-112175982 |
| 2836 | APC | NM_000038 | 19582 | c.3944C>G | p.S1315* | + | 5:112175235-112175235 |
| 2837 | APC | NM_000038 | 19594 | c.4318delC | p.P1440fs*33 | + | 5:112175609-112175609 |
| 2838 | APC | NM_000038 | 19615 | c.4462_4463insTA | p.L1489fs*19 | + | 5:112175753-112175754 |
| 2839 | APC | NM_000038 | 19616 | c.4463T>G | p.L1488* | + | 5:112175754-112175754 |
| 2840 | APC | NM_000038 | 19617 | c.4464delA | p.L1488fs*19 | + | 5:112175755-112175755 |
| 2841 | APC | NM_000038 | 19626 | c.4480delG | p.E1494fs*13 | + | 5:112175771-112175771 |
| 2842 | APC | NM_000038 | 19629 | c.4489delC | p.P1497fs*10 | + | 5:112175780-112175780 |
| 2843 | APC | NM_000038 | 19652 | c.4063T>C | p.S1355P | + | 5:112175354-112175354 |
| 2844 | APC | NM_000038 | 19664 | c.3919delA | p.I1307fs*1 | + | 5:112175210-112175210 |
| 2845 | APC | NM_000038 | 19667 | c.4465_4466insAC | p.L1489fs*19 | + | 5:112175756-112175757 |
| 2846 | APC | NM_000038 | 19674 | c.4483_4484insA | p.S1495fs*19 | + | 5:112175774-112175775 |
| 2847 | APC | NM_000038 | 19688 | c.4386_4387delGA | p.S1465fs*3 | + | 5:112175677-112175678 |
| 2848 | APC | NM_000038 | 19694 | c.4386_4389delGAGA | p.E1464fs*8 | + | 5:112175677-112175680 |
| 2849 | APC | NM_000038 | 19695 | c.4660_4661insA | p.T1556fs*3 | + | 5:112175951-112175952 |
| 2850 | APC | NM_000038 | 19696 | c.4117delC | p.P1373fs*42 | + | 5:112175408-112175408 |
| 2851 | APC | NM_000038 | 19701 | c.4061_4062delTT | p.S1355fs*19 | + | 5:112175352-112175353 |
| 2852 | APC | NM_000038 | 19705 | c.4303A>T | p.R1435* | + | 5:112175594-112175594 |
| 2853 | APC | NM_000038 | 19714 | c.4479delG | p.E1494fs*13 | + | 5:112175770-112175770 |
| 2854 | APC | NM_000038 | 19718 | c.4660_4661insG | p.E1554fs*5 | + | 5:112175951-112175952 |
| 2855 | APC | NM_000038 | 210755 | c.4343_4343delC | p.K1449fs*24 | + | 5:112175634-112175634 |
| 2856 | APC | NM_000038 | 23587 | c.4097C>T | p.A1366V | + | 5:112175388-112175388 |
| 2857 | APC | NM_000038 | 23598 | c.4483delA | p.S1495fs*12 | + | 5:112175774-112175774 |
| 2858 | APC | NM_000038 | 24941 | c.4477delA | p.T1493fs*14 | + | 5:112175768-112175768 |
| 2859 | APC | NM_000038 | 24946 | c.4291delA | p.M1431fs*42 | + | 5:112175582-112175582 |
| 2860 | APC | NM_000038 | 24948 | c.4394delG | p.S1465fs*8 | + | 5:112175685-112175685 |
| 2861 | APC | NM_000038 | 25815 | c.4295_4296CA>AC | p.P1432H | + | 5:112175586-112175587 |
| 2862 | APC | NM_000038 | 25826 | c.4037C>G | p.S1346* | + | 5:112175328-112175328 |
| 2863 | APC | NM_000038 | 25827 | c.4048A>T | p.K1350* | + | 5:112175339-112175339 |
| 2864 | APC | NM_000038 | 27993 | c.4360A>G | p.K1454E | + | 5:112175651-112175651 |
| 2865 | APC | NM_000038 | 29330 | c.3897delT | p.N1300fs*5 | + | 5:112175188-112175188 |
| 2866 | APC | NM_000038 | 29331 | c.4063delT | p.S1355fs*60 | + | 5:112175354-112175354 |
| 2867 | APC | NM_000038 | 30779 | c.4381G>T | p.E1461* | + | 5:112175672-112175672 |
| 2868 | APC | NM_000038 | 32201 | c.3956delC | p.P1319fs*2 | + | 5:112175247-112175247 |
| 2869 | APC | NM_000038 | 32442 | c.4473delT | p.F1491fs*16 | + | 5:112175764-112175764 |
| 2870 | APC | NM_000038 | 41607 | c.4090_4091insA | p.S1364fs*11 | + | 5:112175381-112175382 |
| 2871 | APC | NM_000038 | 41608 | c.4063_4064insT | p.S1355fs*20 | + | 5:112175354-112175355 |
| 2872 | APC | NM_000038 | 41613 | c.3925_3926insA | p.I1311fs*4 | + | 5:112175216-112175217 |
| 2873 | APC | NM_000038 | 41614 | c.4373_4374insC | p.T1459fs*3 | + | 5:112175664-112175665 |
| 2874 | APC | NM_000038 | 41616 | c.4654G>T | p.E1552* | + | 5:112175945-112175945 |
| 2875 | APC | NM_000038 | 41618 | c.4463_4466delTATT | p.L1488fs*18 | + | 5:112175754-112175757 |
| 2876 | APC | NM_000038 | 41619 | c.4118_4118delC | p.P1373fs*42 | + | 5:112175409-112175409 |
| 2877 | APC | NM_000038 | 41621 | c.4665_4666delAA | p.K1555fs*3 | + | 5:112175956-112175957 |
| 2878 | APC | NM_000038 | 41622 | c.4388_4391delGAGA | p.E1464fs*8 | + | 5:112175679-112175682 |
| 2879 | APC | NM_000038 | 41623 | c.4081_4082delCC | p.P1361fs*13 | + | 5:112175372-112175373 |
| 2880 | APC | NM_000038 | 99778 | c.4484G>T | p.S1495I | + | 5:112175775-112175775 |
| 2881 | ALK | NM_004304 | 28054 | c.3520T>G | p.F1174V | - | 2:29443697-29443697 |
| 2882 | ALK | NM_004304 | 28055 | c.3522C>A | p.F1174L | - | 2:29443695-29443695 |
| 2883 | ALK | NM_004304 | 28056 | c.3824G>A | p.R1275Q | - | 2:29432664-29432664 |
| 2884 | ALK | NM_004304 | 28057 | c.3520T>C | p.F1174L | - | 2:29443697-29443697 |
| 2885 | ALK | NM_004304 | 28059 | c.3521T>G | p.F1174C | - | 2:29443696-29443696 |
| 2886 | ALK | NM_004304 | 28061 | c.3522C>G | p.F1174L | - | 2:29443695-29443695 |
| 2887 | ALK | NM_004304 | 28491 | c.3520T>A | p.F1174I | - | 2:29443697-29443697 |
| 2888 | ALK | NM_004304 | 99137 | c.3586C>A | p.L1196M | - | 2:29443631-29443631 |
| 2889 | ALK | NM_004304 | 148825 | c.2535T>C | p.G845G | - | 2:29455267-29455267 |
| 2890 | ALK | NM_004304 | 97185 | c.3455T>G | p.L1152R | - | 2:29445270-29445270 |
| 2891 | ALK | NM_004304 | 99136 | c.3467G>A | p.C1156Y | - | 2:29445258-29445258 |
| 2892 | ALK | NM_004304 | 97028 | c.3592C>T | p.L1198F | - | 2:29443625-29443625 |
| 2893 | ALK | NM_004304 | 1169707 | c.3806G>C | p.G1269A | - | 2:29432682-29432682 |
| 2894 | ALK | NM_004304 | 148825 | c.2535T>C | p.G845G | - | 2:29455267-29455267 |
| 2895 | ALK | NM_004304 | 97185 | c.3455T>G | p.L1152R | - | 2:29445270-29445270 |
| 2896 | ALK | NM_004304 | 99136 | c.3467G>A | p.C1156Y | - | 2:29445258-29445258 |
| 2897 | ALK | NM_004304 | 97028 | c.3592C>T | p.L1198F | - | 2:29443625-29443625 |
| 2898 | ALK | NM_004304 | 1169707 | c.3806G>C | p.G1269A | - | 2:29432682-29432682 |
| 2899 | ALK | NM_004304 |  | c.18end>20 |  | - | 2:29474133-29498362 |
| 2900 | AKT1 | ENST00000349310 | 33765 | c.49G>A | p.E17K | - | 14:105246551-105246551 |
| 2901 | AKT1 | ENST00000349310 | 36918 | c.145G>A | p.E49K | - | 14:105246455-105246455 |
| 2902 | AKT1 | ENST00000349310 | 41225 | c.47-1G>T | p.? | - | 14:105246554-105246554 |
| 2903 | AKT1 | ENST00000349310 | 48226 | c.103T>C | p.F35L | - | 14:105246497-105246497 |
| 2904 | AKT1 | NM_005163 | NOCOSMIC173 | c.517G>C | p.G173R | - | 14:105241463-105241463 |
| 2905 | AKT1 | NM_005163 | NOCOSMIC179 | c.536A>T | p.K179M | - | 14:105241444-105241444 |
| 2906 | ABL1 | X16416 | 12560 | c.944C>T | p.T315I | + | 9:133748283-133748283 |
| 2907 | ABL1 | X16416 | 12573 | c.763G>A | p.E255K | + | 9:133738363-133738363 |
| 2908 | ABL1 | X16416 | 12574 | c.764A>T | p.E255V | + | 9:133738364-133738364 |
| 2909 | ABL1 | X16416 | 12575 | c.951C>G | p.F317L | + | 9:133748290-133748290 |
| 2910 | ABL1 | X16416 | 12576 | c.757T>C | p.Y253H | + | 9:133738357-133738357 |
| 2911 | ABL1 | X16416 | 12577 | c.749G>A | p.G250E | + | 9:133738349-133738349 |
| 2912 | ABL1 | X16416 | 12578 | c.1052T>C | p.M351T | + | 9:133748391-133748391 |
| 2913 | ABL1 | X16416 | 12602 | c.827A>G | p.D276G | + | 9:133747520-133747520 |
| 2914 | ABL1 | X16416 | 12604 | c.1187A>G | p.H396R | + | 9:133750356-133750356 |
| 2915 | ABL1 | X16416 | 12605 | c.1075T>G | p.F359V | + | 9:133748414-133748414 |
| 2916 | ABL1 | X16416 | 12608 | c.730A>G | p.M244V | + | 9:133738330-133738330 |
| 2917 | ABL1 | X16416 | 12609 | c.756G>C | p.Q252H | + | 9:133738356-133738356 |
| 2918 | ABL1 | X16416 | 12610 | c.758A>T | p.Y253F | + | 9:133738358-133738358 |
| 2919 | ABL1 | X16416 | 12611 | c.1064A>G | p.E355G | + | 9:133748403-133748403 |
| 2920 | ABL1 | X16416 | 12631 | c.742C>G | p.L248V | + | 9:133738342-133738342 |
| 2921 | ABL1 | X16416 | 12632 | c.756G>T | p.Q252H | + | 9:133738356-133738356 |
| 2922 | ABL1 | X16416 | 131574 | c.1159T>A | p.L387M | + | 9:133750328-133750328 |
| 2923 | ABL1 | X16416 | 49071 | c.1150C>A | p.L384M | + | 9:133750319-133750319 |
| 2924 | ABL1 | X16416 | 49074 | c.949T>C | p.F317L | + | 9:133748288-133748288 |

**Supplementary Figure S1.** Overall Survival Curves With SNVs of 12 ctDNA Genes in HCC Patients (n = 59). ctDNA, circulating tumor DNA; HCC, hepatocellular carcinoma; SNV, single nucleotide variant. ** P* < 0.05; Log-rank test.


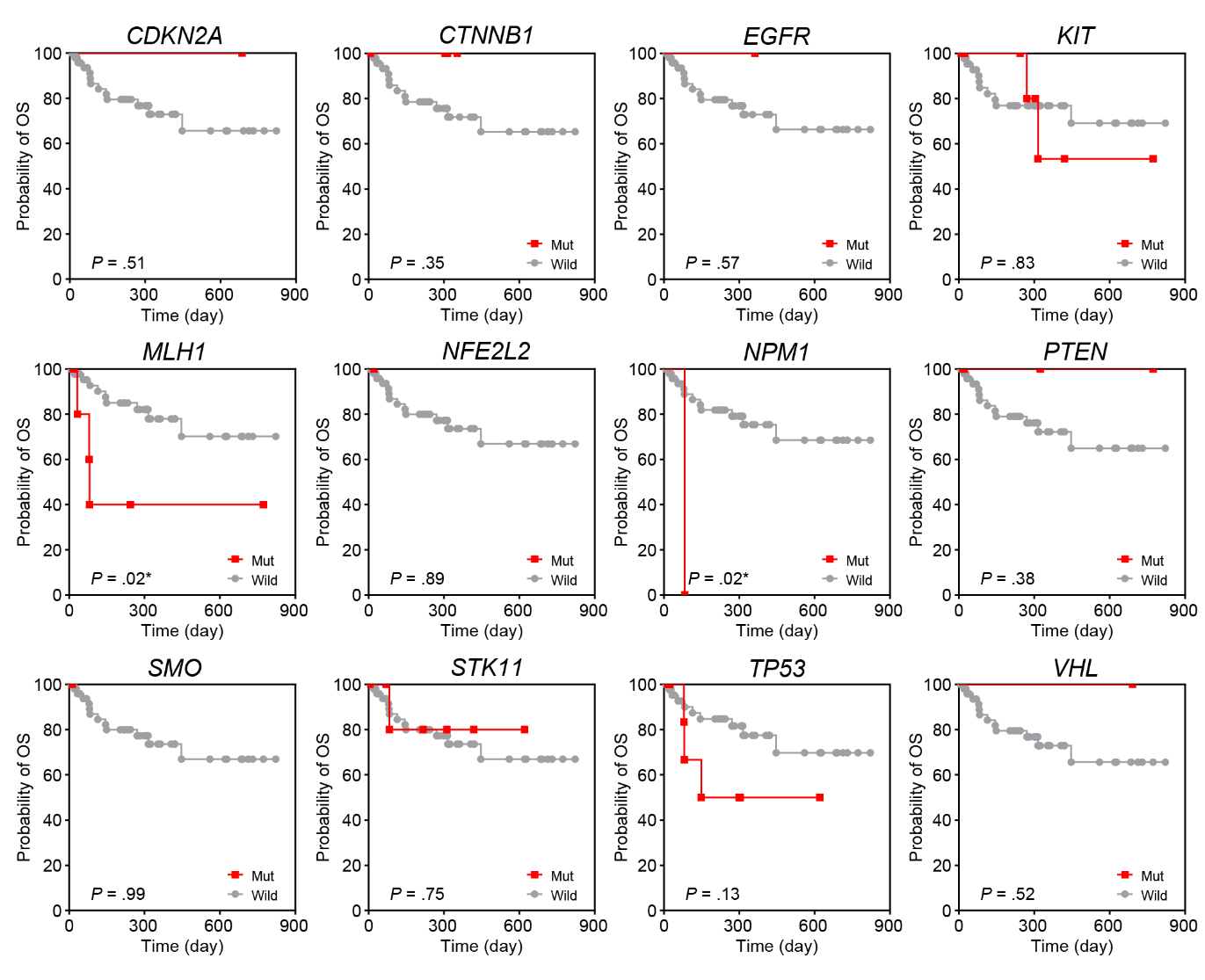


**Supplementary Figure S2.** 2D–Plots and Amount of Positive Droplets of ddPCR Results of 4 ctDNA Genes in HCC Patients. All diagrams represent merged ddPCR results, showing SNV-positive droplet clusters (*blue dots*), negative droplet clusters (*dark grey dots*). The *green dots* represent WT-positive droplets, proving existence of cfDNA in the samples and satisfactory ddPCR conditions. ctDNA, circulating tumor DNA; ddPCR, droplet digital PCR; HCC, hepatocellular carcinoma


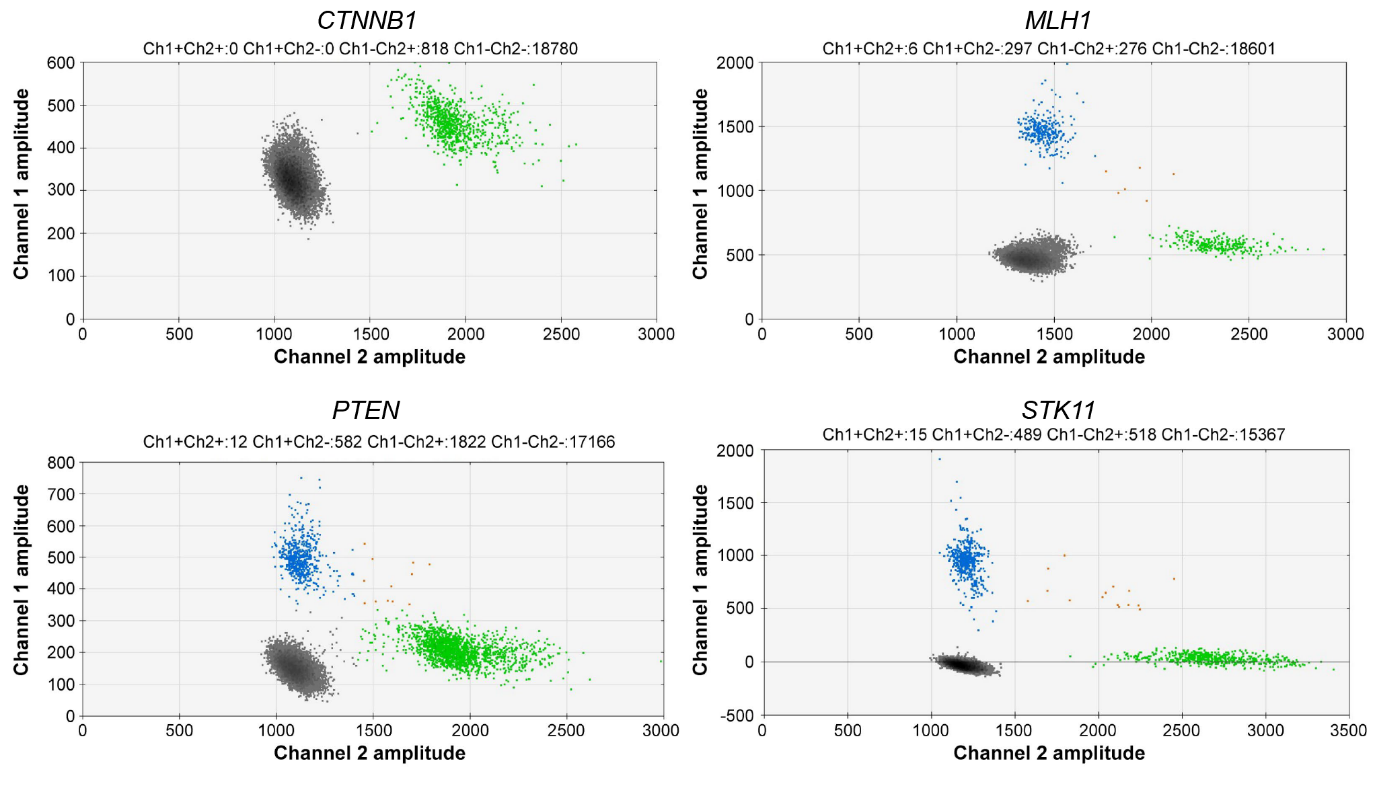


**Supplementary Figure S3.** Prognostic Potential of the MLH1 SNV for HCC. A, Overall survival curves of HCC patients with each Barcelona-Clinic Liver Cancer Group stage. B, Overall survival curves of HCC patients with each modified Union for International Cancer Control IV. HCC, hepatocellular carcinoma; SNV, single nucleotide variant


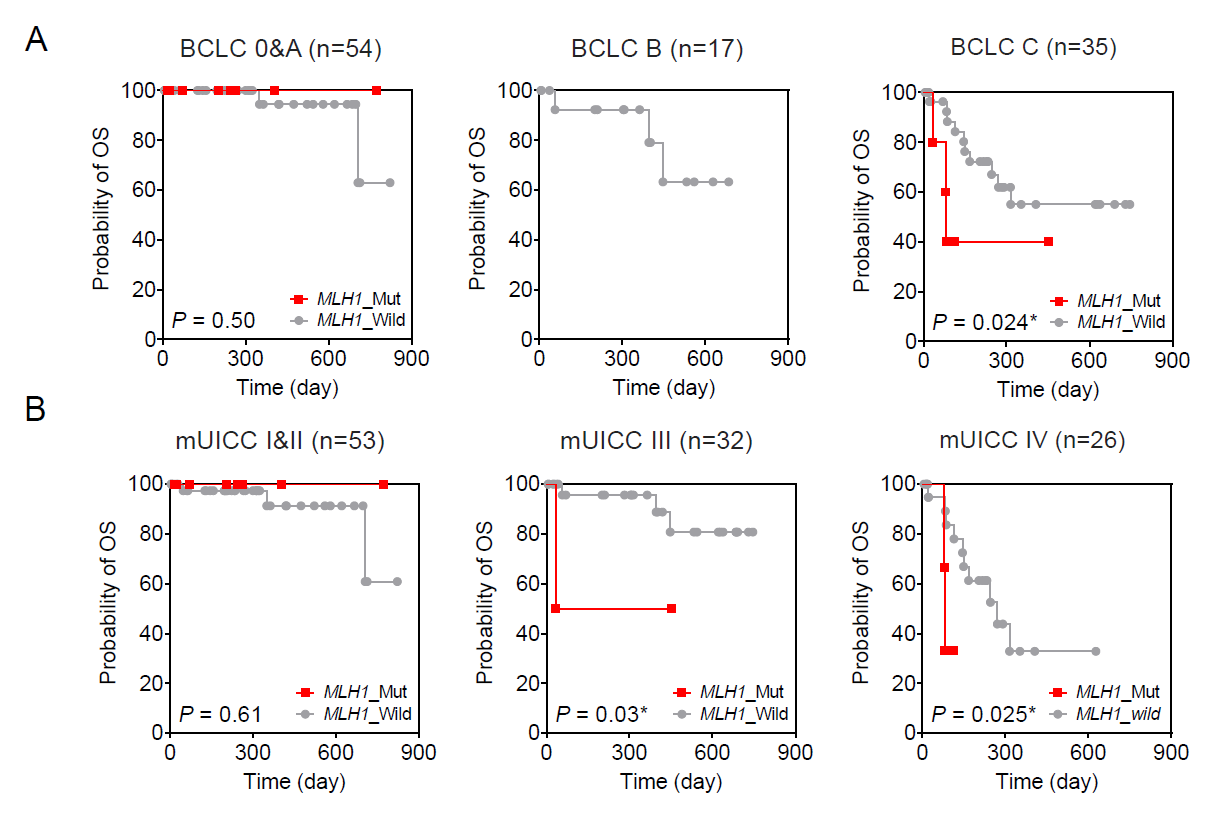


**Supplementary Figure S4.** Analysis of Overall Survival of Patient Outcome Predicted by the SNV Status of ctDNA Genes in HCC. A, Overall survival curves of HCC patients with Barcelona-Clinic Liver Cancer Group stage C . B, Overall survival curves of HCC patients with modified Union for International Cancer Control IV.


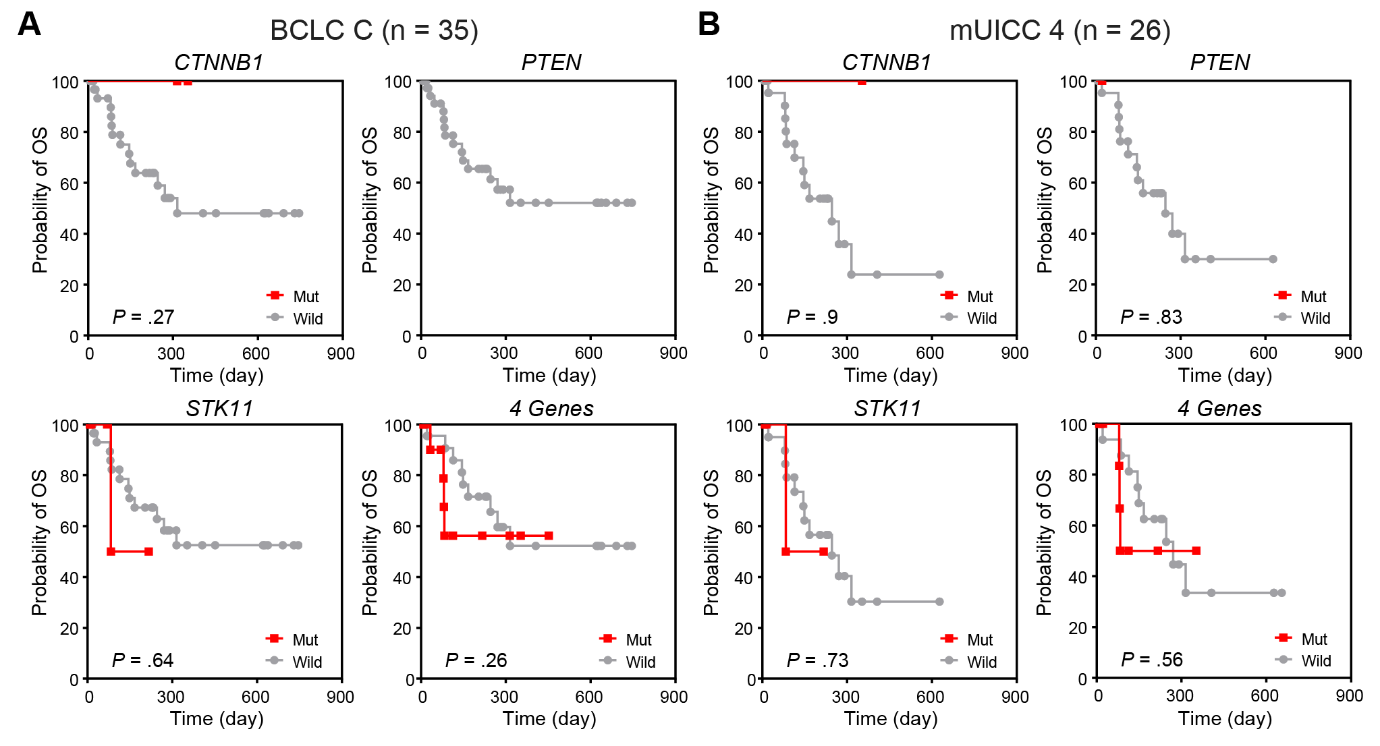


**Supplementary Figure S5.** Association of the AFP level and *MLH1* SNV with overall survival of patients with HCC. AFP positivity was defined as ≥20 ng/mL. AFP, alpha-fetoprotein; HCC, hepatocellular carcinoma; OS, overall survival; SNV, single-nucleotide variant.


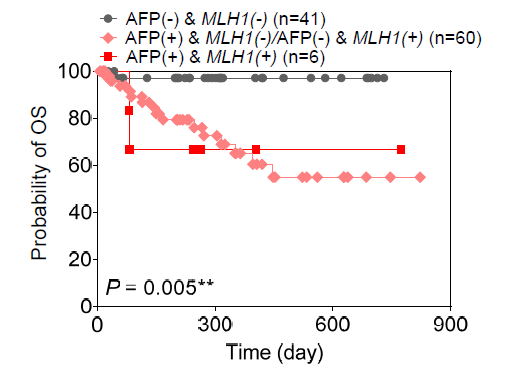


**Supplementary Figure S6.** Strategy Used to Identify Novel Single Nucleotide Variants of ctDNA for HCC. ctDNA, circulating tumor DNA ; ddPCR, droplet digital PCR; HCC, hepatocellular carcinoma; tDNA, tissue genomic DNA .


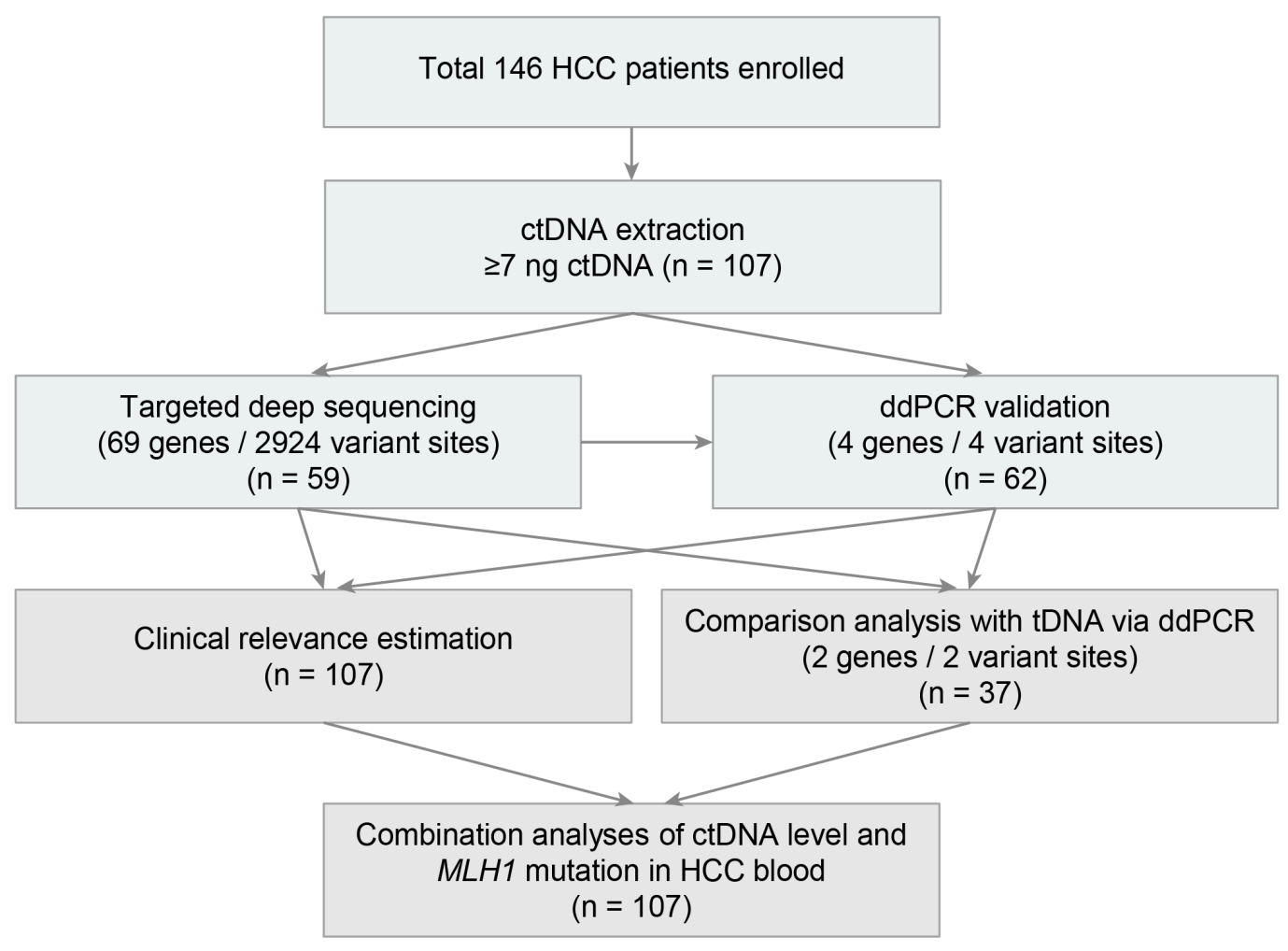


**Supplementary Figure S7.** The Schematic of Data Analyses of Targeted Deep Sequencing


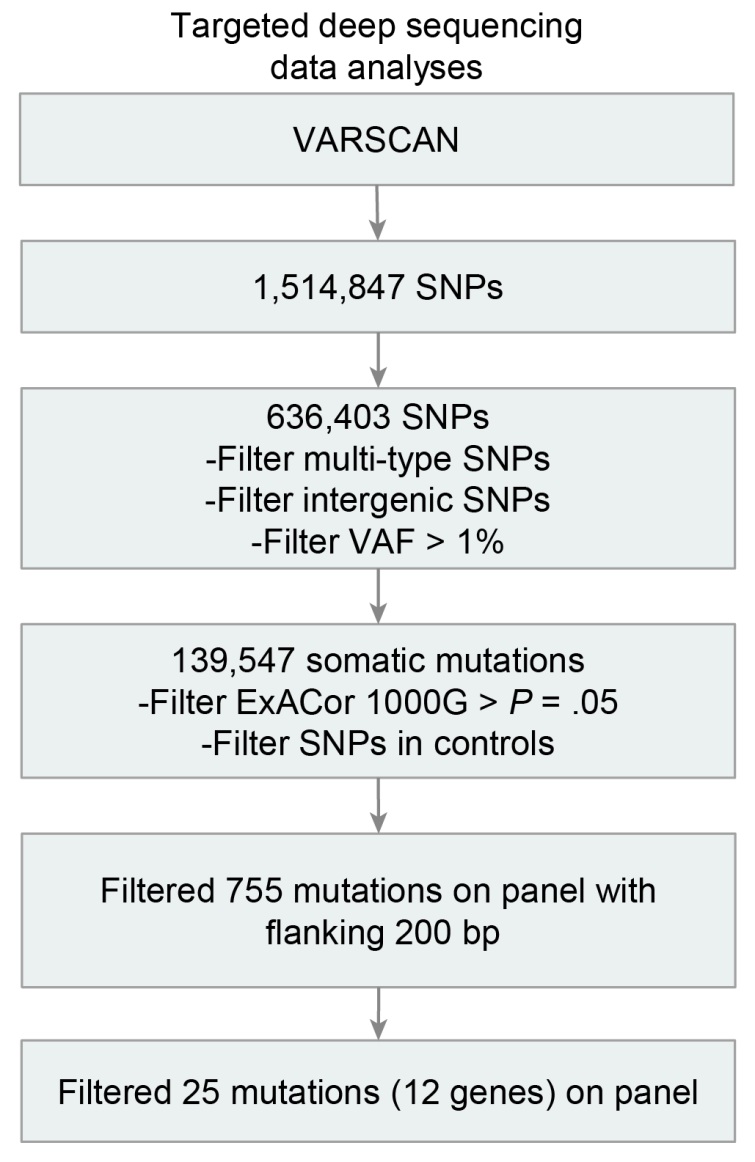

Supplement: Supplementary file 1 — Supplementary information. [file 41598_2020_74494_MOESM1_ESM.docx]
